# Temporal Dynamics, Discovery, and Emergence of Human-Transmissible RNA Viruses

**DOI:** 10.1093/molbev/msad272

**Published:** 2024-01-18

**Authors:** Lu Lu, Feifei Zhang, Liam Brierley, Gail Robertson, Margo Chase-Topping, Samantha Lycett, Mark Woolhouse

**Affiliations:** Roslin Institute, University of Edinburgh, Edinburgh, United Kingdom; Usher Institute, University of Edinburgh, Edinburgh, United Kingdom; Usher Institute, University of Edinburgh, Edinburgh, United Kingdom; National Institute of Health Data Science at Peking University, Beijing, China; Institute of Population Health, University of Liverpool, Liverpool, Unitied Kingdom; Biomathematics and Statistics Scotland, Edinburgh, United Kingdom; Roslin Institute, University of Edinburgh, Edinburgh, United Kingdom; Roslin Institute, University of Edinburgh, Edinburgh, United Kingdom; Usher Institute, University of Edinburgh, Edinburgh, United Kingdom

**Keywords:** RNA viruses, epidemic potential, phylogenetics, emergences, temporal dynamics

## Abstract

Transmissibility, the ability to spread within host populations, is a prerequisite for a pathogen to have epidemic or pandemic potential. Here, we estimate the phylogenies of human infectivity and transmissibility using 1,408 genome sequences from 743 distinct RNA virus species/types in 59 genera. By repeating this analysis using data sets censored by virus discovery date, we explore how temporal changes in the known diversity of RNA viruses—especially recent increases in recognized nonhuman viruses—have altered these phylogenies. Over time, we find significant increases in the proportion of RNA virus genera estimated to have a nonhuman-infective ancestral state, in the fraction of distinct human virus lineages that are purely human-transmissible or strictly zoonotic (compared to mixed lineages), and in the number of human viruses with nearest relatives known not to infect humans. Our results are consistent with viruses that are capable of spreading in human populations commonly emerging from a nonhuman reservoir. This is more likely in lineages that already contain human-transmissible viruses but is rare in lineages that contain only strictly zoonotic viruses.

## Introduction

The coronavirus disease 2019 (COVID-19) pandemic has heightened interest in identifying pathogens most likely to emerge and spread in human populations (Albery et al. 2021; Carlson et al. 2021). One proposal, the Global Virome Project (Carroll et al. 2018), is to conduct comprehensive surveys of viral diversity in nonhuman reservoirs. Nonhuman mammals and birds are of greatest interest; human viruses are not widely shared with other taxa (Woolhouse et al. 2013). This idea has attracted the criticism that the immense scale of the challenge makes it unmanageable and too costly to implement (Holmes et al. 2018). Yet, discovery remains a fundamental part of all viable strategies to detect and identify high-risk pathogens before they spill over into humans (Bernstein et al. 2022).

A key step for making the challenge manageable is the development of accurate and robust methods of identifying the subset of viruses that are of greatest risk to humans and so should be the focus of surveillance efforts. There are currently 2 main approaches: first, systematic ecological risk modeling of the viruses catalogued (see, for example, the Spillover project; Grange et al. 2021), and second, using machine learning to predict phenotypic traits from virus sequence data (Mollentze et al. 2021). Both approaches, or a combination of the two, could help identify viruses with the potential to infect humans even in the absence of human cases.

A difficulty with both approaches is that the data inputs available for these analyses are “incomplete, biased and rapidly changing with ongoing virus discovery” (Wille et al. 2021). A specific concern is that virus discovery has historically prioritized viruses of humans over those of other animals and viruses of livestock (living in proximity to humans) over those of wildlife (Gibb et al. 2022).

This kind of ascertainment bias in ecological studies is a problem that has been recognized for over 200 yr (White 1789). There are several possible solutions. One is structured subsampling of the data to remove biases. Currently, this is only feasible for the small subset of viruses that have been extensively studied in nonhuman reservoirs, such as influenza A. An alternative is the discovery curve, a long-established tool for assessing the extent of missing diversity (Woolhouse et al. 2008). We describe below how we can extend this approach beyond simple species counts to capture temporal trends in other outputs of interest.

Virus phylogeny is 1 plausible predictor of zoonotic and epidemic risk, with novel viruses that are closely related to known human viruses being of greater concern (Grange et al. 2021). Here, we construct sequence-based phylogenetic trees for 52 out of all 59 genera containing human RNA viruses and use the trees to study the phylogenetics of 2 key traits, the ability to infect humans and the ability to transmit within human populations (whether directly or indirectly via a vector or through environmental contamination).

To do this, the viruses are classified by infection/transmission (IT) level (Woolhouse et al. 2013): level 1 (L1) viruses are not known to infect humans; L2 viruses can infect humans from a nonhuman reservoir but do not spread within human populations, i.e. are strictly zoonotic; L34 viruses can spread within human populations and therefore have the potential to cause self-limiting outbreaks (L3) or, if sufficiently transmissible, to cause epidemics (L4). We performed phylogenetic analyses on all virus genera containing human-infective viruses and mapped the IT level of each virus onto the phylogenies. To capture evolutionary changes of IT level, lineages containing only L2 or only L34 viruses are categorized as strictly zoonotic lineages and human-transmissible lineages, respectively, whereas lineages containing both L2 and L34 viruses are categorized as “mixed.” We use the trees to estimate 3 properties: the IT level of the most recent common ancestor of each genus; the number of distinct lineages containing human-infective and/or human-transmissible viruses; and the IT level of the nearest known relative of every human virus.

However, ascertainment bias also applies to phylogenetic trees and these may change as new, related viruses are discovered and included. To address this issue, we repeat the analysis using the data set censored by the year of discovery of each virus, going back to the early 20th century in 10-year steps. This ordered series of analyses indicates how estimates of the 3 properties of interest would have changed over time, particularly as the discovery of nonhuman viruses accelerated in the 21st century, and pinpoints current trends.

## Results

Our data set contained 1,408 representative genome sequences from 743 distinct RNA virus species/types, of which 260 (35%) can infect humans—these come from 59 genera across 24 families. A total of 162 species/types (22%) are strictly zoonotic (L2), providing 281 sequences, 87 from humans and 194 from nonhuman mammals or birds. A total of 98 species/types (13%) are human transmissible (L34), providing 314 sequences, 167 from humans and 147 from nonhuman mammals or birds. The remaining 813 sequences are of L1 viruses from, by definition, nonhuman hosts.

**Fig. 1.**
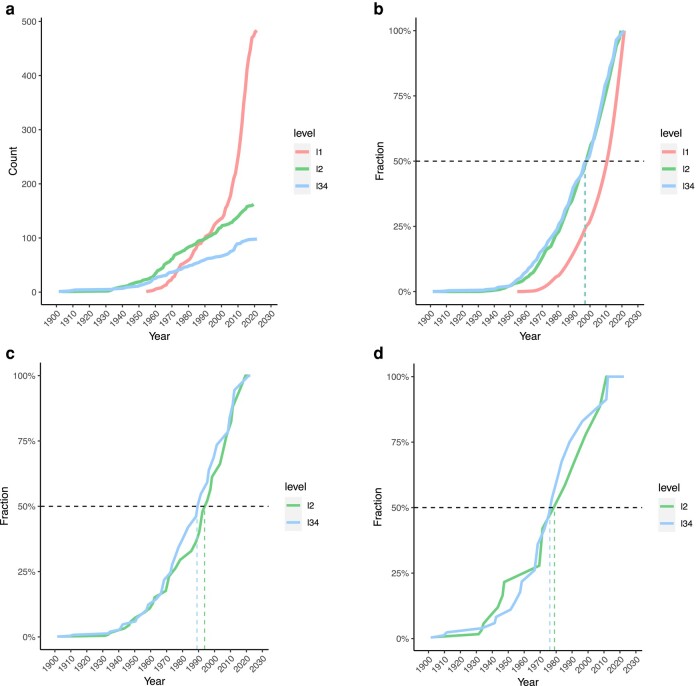
Discovery curves. a) Accumulated numbers of L1, L2, and L34 virus species/types over time. b) As a) showing cumulative fractions. c) Accumulated fractions of virus genera containing L2 or L34 virus species/types or both (note that some genera contribute to both curves). d) Accumulated fractions of virus families containing L2 or L34 virus species/types or both (note that some families contribute to both curves).

Known L2 and L34 species/types have accumulated at average rates of 1.9 and 1.2 yr^−1^, respectively, since 1938 (Fig. 1a). Numbers of nonhuman L1 viruses have increased much faster over the same period, particularly during the past 2 decades (noting that we only include L1 species/types from the 59 genera of interest here). There are minimal differences in the shape of the discovery curves for L2 and L34 viruses with 50% of the currently recognized viruses in both IT levels reported by 1998 (Fig. 1b).

Though the rate of discovery of new human virus species/types remains high, there is evidence of a comparative slowdown in the accumulation of higher-level taxonomic diversity (Fig. 1c and d). Of the 39 genera and 20 families containing L2 viruses, 50% were known to do so by 1994 and 1978, respectively. Of the 42 genera and 22 families containing L34 viruses, 50% were known to do so by 1989 and 1976, respectively.

**Fig. 2.**
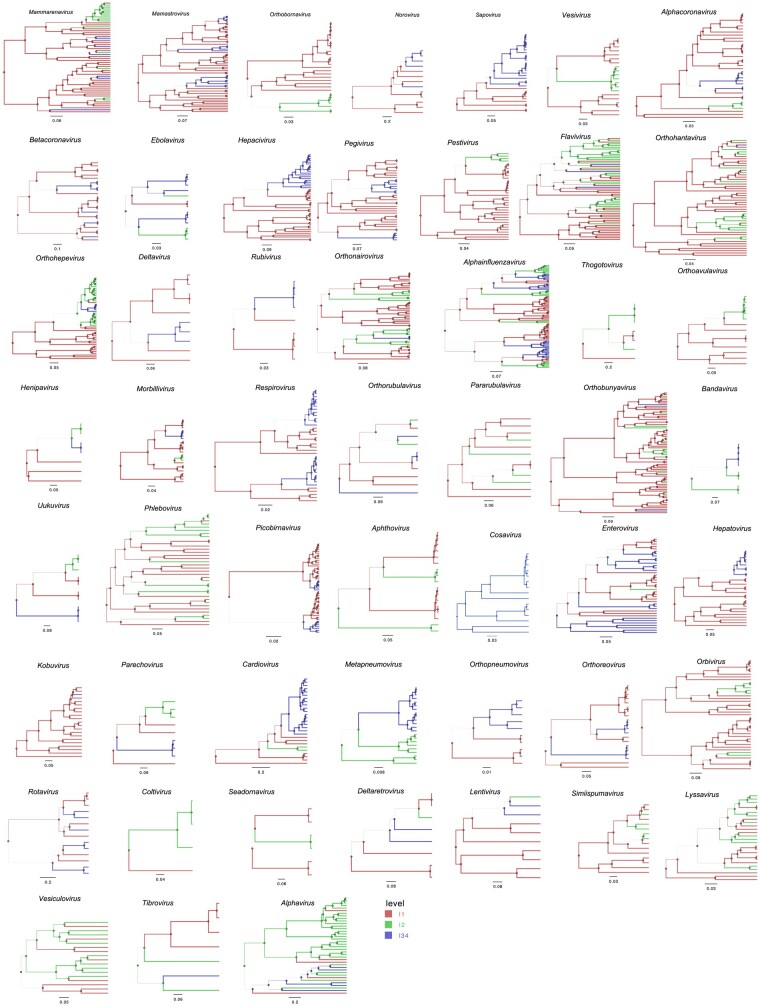
Bayesian maximum clade credibility (MCC) trees for members of 52 virus genera (excluding 7 single species genera) using polymerase protein sequences (listed in supplementary data file S4, Supplementary Material online). Phylogenies show the most probable transitions between nonhuman viruses (L1), viruses infective to humans (L2), and viruses transmissible in human populations (L34).

**Fig. 3.**
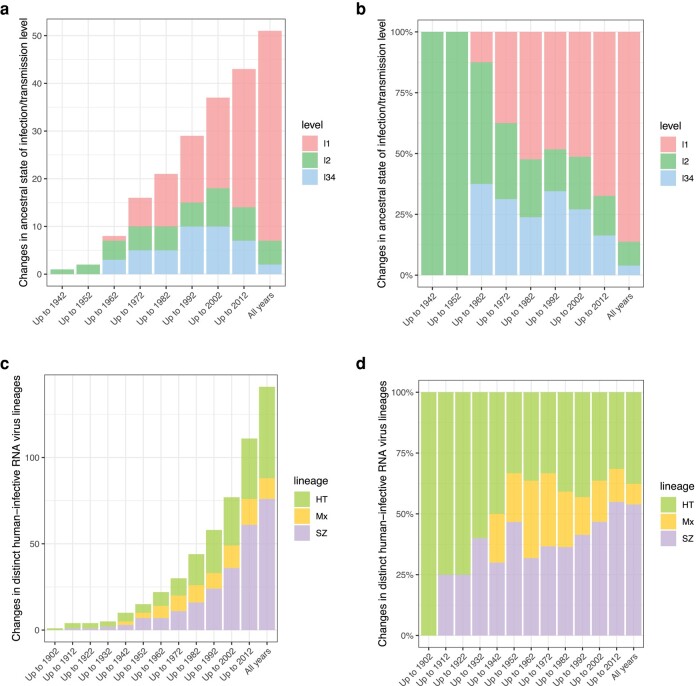
Temporal patterns. a) Changes in most probable ancestral state (L1, L2, or L34) of human RNA virus genera estimated using sequences from species/types discovered before cutoff date shown. b) As a) showing fractions. c) Changes in the numbers of distinct human-infective RNA virus lineages (human transmissible, strictly zoonotic, or mixed) estimated using sequences from species/types discovered before cutoff date shown. d) As c) showing fractions.

Phylogenetic trees for the 52 genera containing more than 1 virus species/type are shown in Fig. 2. Of the other 7 genera, 3 contain just a single L2 species/type and 4 a single L34 species/type. The most likely ancestral state is L1 for 44 (85%) of these 52 genera, though the expected value (the sum of estimated posterior support values) is lower at 36.2 (70%). Both the absolute number and proportion of genera with a most likely L1 ancestral state have increased markedly over time, and over the last 20 yr, the absolute number with most likely L2 or L34 ancestral states has declined (Fig. 3a and b).

From the phylogenies (plus the 7 single L2/L34 species/type genera), we can identify 149 distinct lineages containing human viruses (supplementary data file S3, Supplementary Material online). Of these, 79 are strictly zoonotic (containing only L2 species/types), 58 are human transmissible (containing only L34), but only 12 are mixed (containing both). Of the 12 mixed lineages, 11 (92%) contain enveloped viruses, and 4 (33%) contain vector-borne viruses. Of all lineages, 111 (74%) contain just a single human virus species/type.

**Fig. 4.**
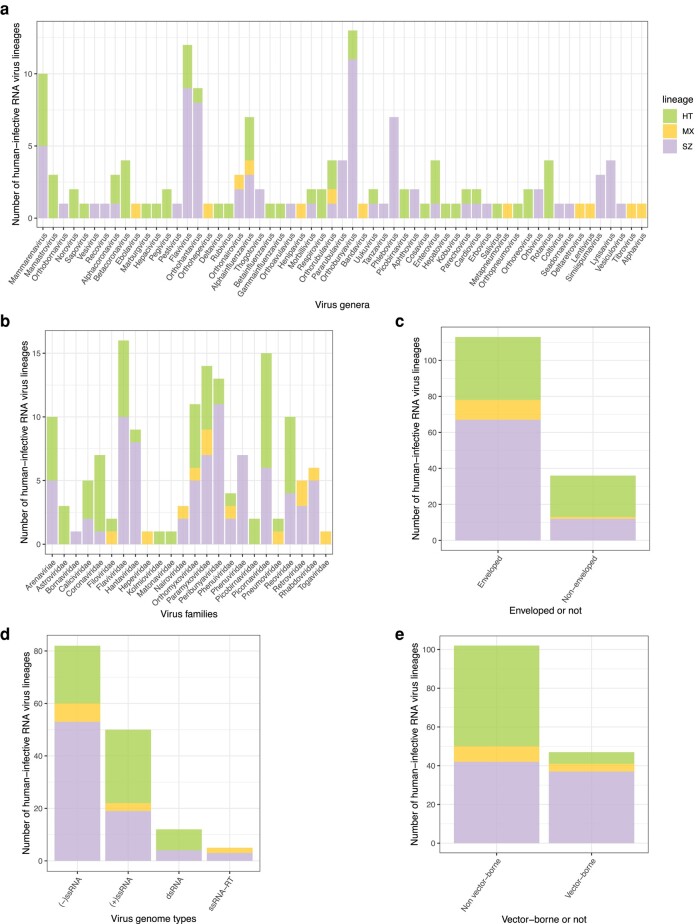
Numbers of distinct human-infective RNA virus lineages (*n* = 149) in different categories. Three lineage types are distinguished: only strictly zoonotic (SZ), only human transmissible (HT), and both (MX). a) Stacked bar chart of strictly zoonotic, human-transmissible, and mixed lineage counts by genus. b) By virus family. c) By enveloped/nonenveloped. d) By virus genome type. e) By vector borne/nonvector borne.

Overall, evidence from our study indicates that human transmissibility has independently evolved on at least 70 occasions across a wide range of taxa. This conclusion is drawn from the estimation of transitions from L1 or L2 to L34 in all phylogenies (Figs. 2 and 4). In at least 15 genera and 14 families, human transmissibility has evolved more than once (Fig. 4a and b). Lineages containing human-transmissible viruses (only L34 or L34 plus L2 viruses) are not disproportionately present across genome type or enveloped/nonenveloped (Table 1 and Fig. 4c and d). However, they are significantly underrepresented relative to strictly zoonotic lineages among vector-borne viruses (Table 1 and Fig. 4e).

**Table 1:** Ancestor node traits as predictors of lineage types

Variables	Coefficient	Lower 95% CI	Upper 95% CI	χ	df	*P* value
Genome type	-	-	-	4.22	3	0.24
Enveloped (+)ssRNA	1.69	−0.01	3.39	-	-	-
Non-enveloped (+)ssRNA	0.64	−0.81	2.08	-	-	-
dsRNA	0.77	−0.89	2.43	-	-	-
Vector borne	−2.05	−3.47	−0.63	8.01	1	0.005

Outputs are from a binomial GLMM with genus as a random factor. The model compares strictly zoonotic (*N* = 79) to human-transmissible and mixed lineages (*N* = 70), reporting log odds, 95% confidence intervals (CIs), and results of Wald tests in which each fixed effect is removed from the model and tested in turn. Genome type and enveloped/nonenveloped are combined as a composite variable with 4 levels: (−)ssRNA, enveloped (+)ssRNA, nonenveloped (+)ssRNA, and dsRNA. Reference categories are (−)ssRNA and nonvector borne. Coefficient estimates are shown for each level of the variable rather than for the variable overall. *N* = 149.

The number of distinct, identifiable human virus lineages has grown over time (Fig. 3c), faster than the numbers of human virus species/types (cf. Fig. 1a). However, the absolute number of mixed lineages is now falling, and the proportion of mixed lineages has been falling over the past 50 yr (Fig. 3c and d).

**Fig. 5.**
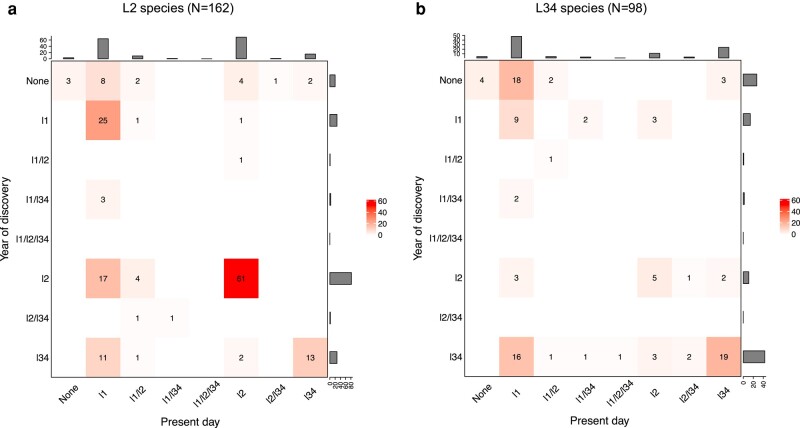
Nearest relatives. Matrix showing changes in nearest known congeneric relative from time of discovery to present day for L2 species a) and L34 species b) including 101 species/types and 8 possible categories: L1, L2, L34, L1/L2, L1/L34, L2/L34, L1/L2/L34, and none.

There is clear evidence of phylogenetic clustering of IT level. At the time of discovery, L34 viruses were 3.9× more likely to have a L34 nearest relative than L2, and L2 viruses were 3.0× more likely to have a L2 nearest relative than L34 (*P* < 0.001). However, the IT level of the nearest known relatives of human viruses has changed for 37% of L2 viruses and 61% of L34 viruses since they were first discovered (Fig. 5). The majority of human viruses with no congeneric relative at the time of discovery (26 to 30 out of 47, where the range allows for some ambiguity in nearest relative for subset of phylogenies) are now considered most closely related to a L1 virus. Of human viruses originally with a L2 or L34 nearest relative, at least 28% (47 to 57 out of 165) now have a L1 nearest relative. Less than 10% (15 to 16 out of 162) L2 viruses now have a L34 nearest known relative and less than 20% (11 to 19 out of 98) L34 viruses a L2 nearest known relative (Fig. 5).

## Discussion

Virus evolution, discovery, and emergence are distinct concepts, and it is not always clear how knowledge of one might inform knowledge of the others. Here, we have filtered patterns in RNA virus phylogenies (evolution) through a lens of increasing knowledge of virus diversity (discovery) to draw inferences about future risk to humans (emergence). Our focus is transmissibility—the key trait that determines epidemic potential. Only 37% of the human RNA viruses in our data set are human transmissible (L34), so it is important to identify risk factors specific to this subset.

The substantial increase in effort invested in virus detection in the past 20 yr (Ladner 2021) has had no obvious impact on the rate of discovery of human RNA viruses, whether they are human transmissible (L34) or strictly zoonotic (L2; Fig. 1). Fewer new human viruses were discovered in the period 2010 to 2019 (23) than 1960 to 1969 (28). There is even clearer evidence of a slowdown in the accumulation of higher taxa (genera and families) containing L2 and/or L34 viruses (Fig. 1), a pattern consistent with a limited diversity of these viruses (Woolhouse and Adair 2013). These observations suggest that new human RNA viruses are becoming harder to find; it is even possible that the majority of extant human RNA viruses have already been discovered (Woolhouse and Adair 2013). In marked contrast, there has been a rapid and accelerating increase in numbers of known nonhuman viruses (L1; Fig. 1). Our understanding of the phylogenetic origins of human viruses has changed as a consequence and is likely to continue to do so as it is widely anticipated that many more L1 viruses remain to be discovered (Carlson et al. 2021; Wille et al. 2021).

Over time, as more L1 viruses have been recognized and placed in phylogenies, 3 patterns have become apparent. First, both the absolute number and the fraction of RNA virus genera estimated to have a human-infective ancestral state have sharply declined (Fig. 4a and b); just 15% of multispecies genera now fall in this category, and we conjecture that the decline will continue. Second, the number of distinct human virus lineages has increased as more L1 viruses have been discovered, but the number and fraction that contain both L2 and L34 viruses (mixed lineages) are both declining (Fig. 3c and d). Again, we expect these trends to continue. Third, the number of human viruses with probable or possible L1 nearest relatives has increased since the viruses were first discovered (Fig. 5). For human-transmissible viruses, 48 to 56 out of 98 are now estimated to have a nonhuman-infective nearest relative, approximately double the number with a human-transmissible nearest relative and 4× the number with a strictly zoonotic nearest relative (Fig. 5b).

The appearance of a new (and extant) human-transmissible lineage appears to be a relatively infrequent event; there are just 73 known instances over the entire evolutionary history of the 52 genera considered here (Fig. 2). Less expectedly, our findings suggest that the same applies to strictly zoonotic lineages: just 51 instances. We have previously suggested a model where the L2 trait is easily gained and easily lost (Woolhouse et al. 2013), but that model is not supported by the analysis reported here.

Of the major genome types, (−)ssRNA viruses appear least likely to adapt fully to human hosts. They are disproportionately strictly zoonotic (Geoghegan et al. 2016), and they are rarely specialist human viruses: just 2 (−)ssRNA species (both of the *Rubulavirus* genus) are thought to infect only humans, compared with 20 (+)ssRNA species from multiple genera (Woolhouse and Brierley 2018). Most vector-borne viruses are also poorly adapted to humans; just 4 species (all (+)ssRNA viruses) are capable of extensive spread in human populations (level 4 not level 3), whereas strictly zoonotic vector-borne viruses (L2) are common (Woolhouse and Brierley 2018). A possible explanation is that viruses transmitted by biting arthropods pass directly into the host's blood system, thereby bypassing infection barriers without necessarily possessing traits associated with human transmissibility.

Together, these observations are consistent with the hypothesis that there is a phylogenetic separation between strictly zoonotic and human-transmissible RNA virus lineages, and they typically evolve independently from nonhuman viruses. This pattern is only now being revealed as previously underrepresented nonhuman viruses are discovered in large numbers. The pattern is partly accounted for by taxonomy as human-transmissible and strictly zoonotic virus lineages are differently distributed across RNA virus taxa (Fig. 4). However, there is still substantial taxonomic overlap, and even among genera with both human-transmissible and strictly zoonotic viruses, there are only 12 mixed strictly zoonotic and human-transmissible lineages of a total of 82.

The phylogenetic pattern is consistent with epidemiological experience of human RNA viruses going back over 100 yr. There have been multiple examples of the emergence of new human-transmissible viruses from nonhuman reservoirs (including HIV-1 and SARS-CoV-2) and several examples of outbreak (L3 by definition) viruses becoming epidemic (L4) viruses (including Ebola virus and Chikungunya virus). In contrast, there have been no clear examples of strictly zoonotic (L2) viruses becoming human transmissible (though the possibility has been raised as a concern, especially for the avian influenzas; Woolhouse et al. 2016).

The absence of a clear phylogenetic association between human infectivity and human transmissibility may reflect the key role that cell receptors play in determining a virus’ capacity both to infect and to be transmitted by humans (Kuiken et al. 2006; Woolhouse et al. 2012). Cell receptor usage varies between and sometimes within virus genera (Lefkowitz et al. 2018). Host switching (to humans or to any other new host) is facilitated by a virus using a cell receptor with an amino acid sequence that is conserved between hosts (Woolhouse et al. 2012). However, if the receptor has a differential expression across human tissues, then infectivity may not equate with transmissibility (Kuiken et al. 2006). Evolutionary shifts in receptor usage can therefore lead to changes in human infectivity, transmissibility, or both.

One limitation to our study is the possible misclassification of IT level. We note that 25 of the L2 virus species/types, but none of the L34 viruses, in our data set were first discovered in nonhuman hosts and would have been classified as L1 for periods ranging from 1 to >60 yr. Some viruses currently classified as L1 may turn out to be able to infect humans in the future. Much less frequently, putative L2 viruses may be reclassified as L34 (e.g. Nipah virus, though after <1 yr). However, IT level remains unchanged for the great majority of RNA viruses, so reclassifications seem likely to cause only modest shifts to the patterns reported here.

A second limitation is the definition of “species” and “types.” Here, we use these taxa partly to guide selection of sequences for analysis, aiming to capture as much as practicable of the diversity of nonhuman, human-infective, and human-transmissible viruses. For our main analysis, we use lineage rather than taxon as the unit of study (Figs. 2–4). Where we do use counts of species/types (Figs. 1 and 5), this is for the purpose of capturing historical patterns in the most straightforward way. However, this does make the outputs of those analyses sensitive to the extent of splitting or lumping in the International Committee on Taxonomy of Viruses (ICTV) classifications, particularly if host range were used as a taxonomic indicator. We minimize this issue by applying current taxonomic classifications retrospectively rather than attempting to trace the history of virus taxonomy back in time. However, we note that taxonomic classifications may change in the future, and our analysis would then need to be updated—the same is true for any of a very large number of published studies of patterns in the taxonomic diversity of viruses.

The results of this study are consistent with a model of emerging infections whereby new human viruses with epidemic potential are related to other human-transmissible viruses but emerge independently from a nonhuman reservoir. Importantly, having a close relative that is strictly zoonotic does not appear to be a risk factor for epidemic potential—human-transmissible and strictly zoonotic viruses tend to evolve independently. This model was first proposed in 2019 (Lu et al. 2019) and accurately captured the ancestry of SARS-CoV-2, i.e. closely related to SARS-CoV but even more closely to SARS-like viruses in bats (Zhou et al. 2021). It is already discernible as a common model of emergence, even with a data set heavily biased toward human viruses. On current trends, as more and more viruses are discovered in nonhuman reservoirs, it seems likely to become the dominant model, helping to narrow down the list of viruses with greatest potential to cause epidemics in human populations.

## Materials and Methods

### Data

Data for this study were compiled from 3 sources: our previously published human RNA virus database (Woolhouse and Brierley 2018), the ICTV taxonomy (2022; Lefkowitz et al. 2018), and the “The Global Virome in One Network database” (VIRION, version 0.2.1; Carlson et al. 2022). Our main data set consisted of all 59 human RNA virus genera, each containing at least 1 human virus species. The IT level of each viruswas classified as L1, L2, or L3/4, using an updated version of our published data set (Woolhouse and Brierley 2018). Five virus species (*Influenza A virus*, *Norovirus*, *Sapovirus*, *Orthohepevirus A*, and *Aichivirus A*) with recognized subtypes that differ in IT level were included as types rather than single species. Among the 59 virus genera, 52 contained multiple virus species/types and more than 1 transmission level, 7 (*Recovirus*, *Marburgvirus*, *Betainfluenzavirus*, *Gammainfluenzavirus*, *Tanzavirus*, *Erbovirus*, and *Salivirus*) contained a single species and a single level, and *Cosavirus* contained multiple species and a single level (see supplementary data file S1, Supplementary Material online for details). There have been recent suggestions that viruses in the *Picobirnavirus* genus are not truly human infective (Ghosh and Malik 2021; Reddy et al. 2023), but we include them here while the question remains unresolved, noting that the genus contributes just 2 human-transmissible lineages so does not materially influence our findings.

The 59 virus genera were composed of 743 virus species/types, 260 of which are able to infect humans. These included 162 strictly zoonotic species/types (classified as L2) and 98 human-transmissible species/types (L34). The remaining 483 species/types were nonhuman infective (L1). For each virus species/type, we obtained a sequence data set from the NCBI GenBank database, including 1,408 complete or nearly complete polymerase (or functional equivalent) gene sequences that were the reference sequences indicated in the ICTV taxonomy and were found naturally (i.e. excluding deliberate laboratory exposures) in humans, other mammals, or birds. We also linked these sequences to the year in which the virus species was first discovered in the given host according to the VIRION database. We used the discovery year in humans rather than other hosts for human viruses (L2 and L34) if there was a discrepancy between VIRION database and our human-infective virus database. This approach was applied to 47% of human virus species/types, with 24% of species/types showing a difference of more than 5 yr. Links to the sequences and associated metadata can be found in supplementary data file S2, Supplementary Material online.

### Phylogenetic Analysis

For each genus, we translated the polymerase gene sequences to amino acids and aligned them using MAFFT (version 6.240; Katoh and Standley 2014) and then reconstructed Bayesian phylogenies for each of the 52 virus genera using the BEAST software package (V1.10.4; Suchard et al. 2018). Our primary focus was on the tree topology, with branch lengths scaling to the number of amino acid substitutions. We did not attempt to date the nodes, as such estimates are likely to be unreliable given the long-time scales involved. We used a WAG model with a gamma distribution across sites as the substitution model, an uncorrelated lognormal relaxed molecular clock model, and a constant size coalescent process prior over the phylogenies. We allowed the branch length to be scaled by substitutions per site rather than by time (with ucld.mean equal to 1). The MCMC chains were run for 100 million iterations with subsampling every 10,000 iterations and a 10% burn-in. We validated our phylogenies by comparing their topologies to the representative phylogenies at the family/genus level published by the ICTV taxonomy (Lefkowitz et al. 2018).

We reconstructed the ancestral state of IT level using asymmetric discrete trait models (Lemey et al. 2009) over each genus-level tree to visualize and summarize the phylogenetic separation between strictly zoonotic and human-transmissible RNA virus lineages and lineages containing both L2 and L3/4 viruses (supplementary data file S3, Supplementary Material online). All of the Bayesian phylogenies mapped by IT level traits are provided in Fig. 2 and supplementary data file S4, Supplementary Material online.

To compare the lineages identified using current available sequence data to those of viruses discovered in the past, we generated sets of historical phylogenies by dropping the tips of the original Bayesian phylogenies of the 52 virus genera in 10-year increments reverse to time (up to 2022, up to 2012, up to 2002, and so on until up to 1942) in the ape package (version 5.6-2; Paradis and Schliep 2019) in R. We then reconstructed the evolution of IT level changes using the ancestral character estimation (ACE) function, also available in the ape package. Each IT level was considered as a discrete trait, and maximum likelihood was employed to estimate the ancestral state. For this analysis, we employed the all-rates-different model (ARD model), assuming independent transitions among states with varying rates. The analyses were executed on a set of trees (total *n* = 210) with tips removed back in time from the 52 original Bayesian phylogenetic trees (supplementary data file S5, Supplementary Material online). We then compared the lineage types and ancestral states of the genera across different time periods. Similarly, we compared the nearest known relative of each L2 and L3/4 virus now to its nearest known relative at the time of discovery, as defined according to the trees.

### Statistical Analysis

We used a binomial generalized linear mixed model to examine whether proportions of the 3 different lineage types (strictly zoonotic, human transmissible, and mixed) varied among families, genera, genome types, and between vector-borne/nonvector-borne viruses. The model compares strictly zoonotic (*N* = 79) to human-transmissible and mixed lineages (*N* = 70). We constructed a composite variable representing the genome type and enveloped structure of viruses involved in transition events. This had 4 levels: (−)ssRNA (all enveloped), (+)ssRNA enveloped, (+)ssRNA nonenveloped, and dsRNA (all nonenveloped). Models with genus as the only random factor and models with both genus and family as random factors had similar Akaike information criterion (AIC) values (ΔAIC < 2); only the former are reported here.

## Supplementary Material

msad272_Supplementary_Data

## Data Availability

The supporting data used in these analyses are all freely available (see supplementary data files, Supplementary Material online and included link to sequence data in GenBank).
