# Supplementary material for: Temporal Dynamics, Discovery, and Emergence of Human-Transmissible RNA Viruses
Source: Mol Biol Evol. 2024 Jan 18;41(1):msad272. doi: 10.1093/molbev/msad272 (PMC10797954; doi:10.1093/molbev/msad272)
Supplement: msad272_Supplementary_Data [file msad272_supplementary_data.zip › Data file 4.pdf]

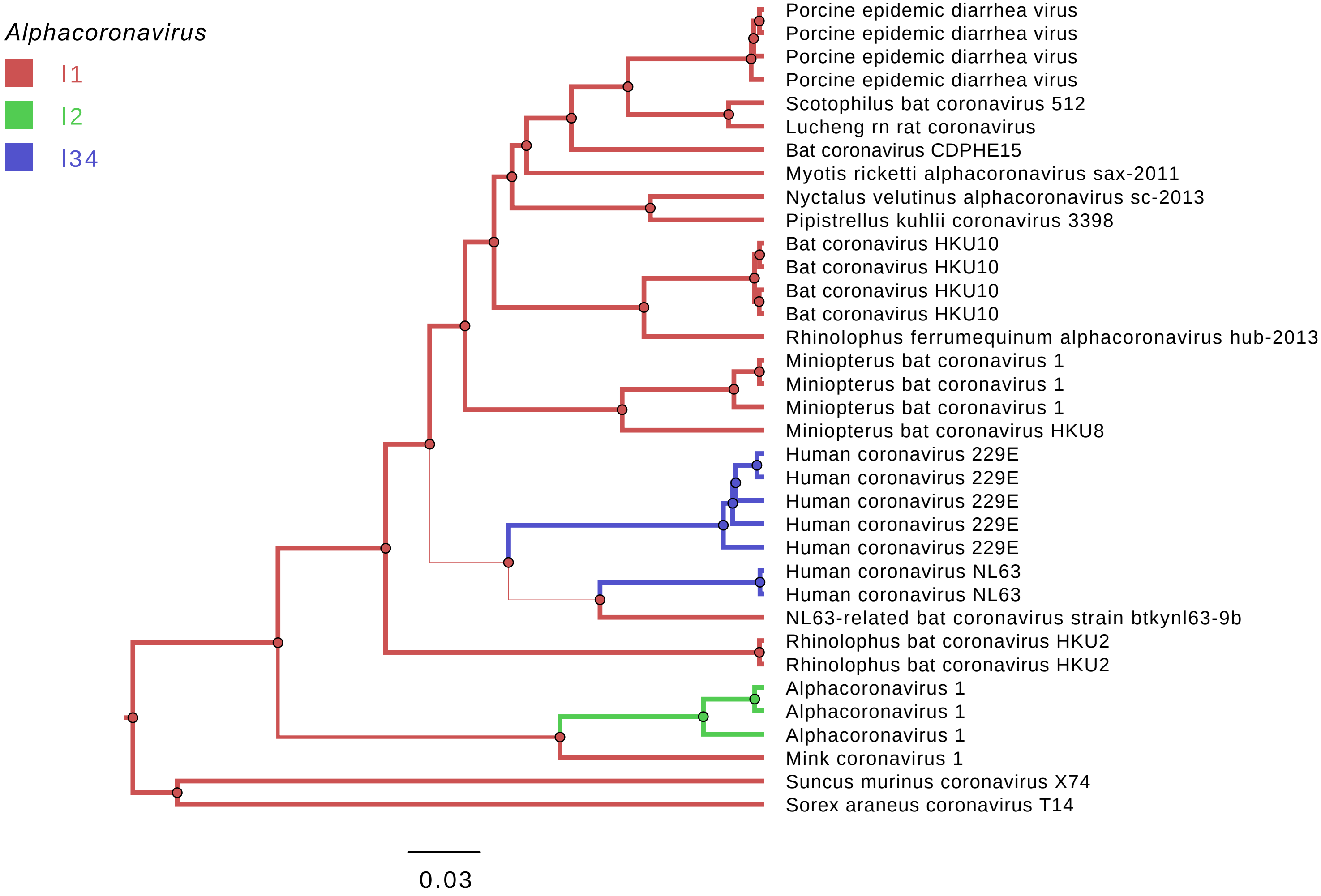

Alphacoronavirus
I1
I2
I34
Porcine epidemic diarrhea virus
Porcine epidemic diarrhea virus
Porcine epidemic diarrhea virus
Porcine epidemic diarrhea virus
Scotophilus bat coronavirus 512
Lucheng rn rat coronavirus
Bat coronavirus CDPHE15
Myotis ricketti alphacoronavirus sax-2011
Nyctalus velutinus alphacoronavirus sc-2013
Pipistrellus kuhlii coronavirus 3398
Bat coronavirus HKU10
Bat coronavirus HKU10
Bat coronavirus HKU10
Bat coronavirus HKU10
Rhinolophus ferrumequinum alphacoronavirus hub-2013
Miniopterus bat coronavirus 1
Miniopterus bat coronavirus 1
Miniopterus bat coronavirus 1
Miniopterus bat coronavirus HKU8
Human coronavirus 229E
Human coronavirus 229E
Human coronavirus 229E
Human coronavirus 229E
Human coronavirus 229E
Human coronavirus NL63
Human coronavirus NL63
NL63-related bat coronavirus strain btkynl63-9b
Rhinolophus bat coronavirus HKU2
Rhinolophus bat coronavirus HKU2
Alphacoronavirus 1
Alphacoronavirus 1
Alphacoronavirus 1
Mink coronavirus 1
Suncus murinus coronavirus X74
Sorex araneus coronavirus T14
0.03

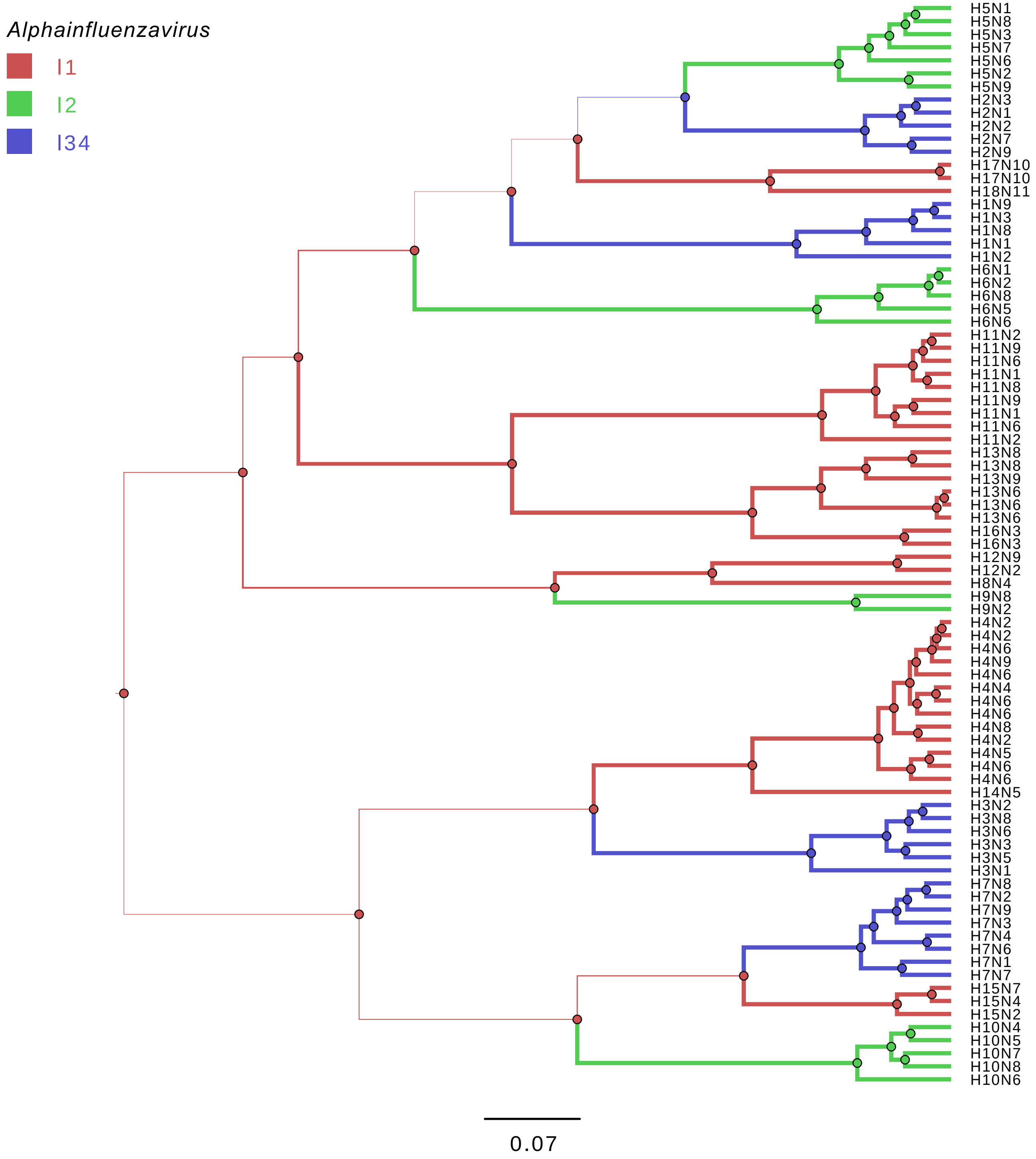

Alphainfluenzavirus
I1
I2
I34
H5N1
H5N8
H5N3
H5N7
H5N6
H5N2
H5N9
H2N3
H2N1
H2N2
H2N7
H2N9
H17N10
H17N10
H18N11
H1N9
H1N3
H1N8
H1N1
H1N2
H6N1
H6N2
H6N8
H6N5
H6N6
H11N2
H11N9
H11N6
H11N1
H11N8
H11N9
H11N1
H11N6
H11N2
H13N8
H13N8
H13N9
H13N6
H13N6
H13N6
H16N3
H16N3
H12N9
H12N2
H8N4
H9N8
H9N2
H4N2
H4N2
H4N6
H4N9
H4N6
H4N4
H4N6
H4N6
H4N8
H4N2
H4N5
H4N6
H4N6
H14N5
H3N2
H3N8
H3N6
H3N3
H3N5
H3N1
H7N8
H7N2
H7N9
H7N3
H7N4
H7N6
H7N1
H7N7
H15N7
H15N4
H15N2
H10N4
H10N5
H10N7
H10N8
H10N6
0.07

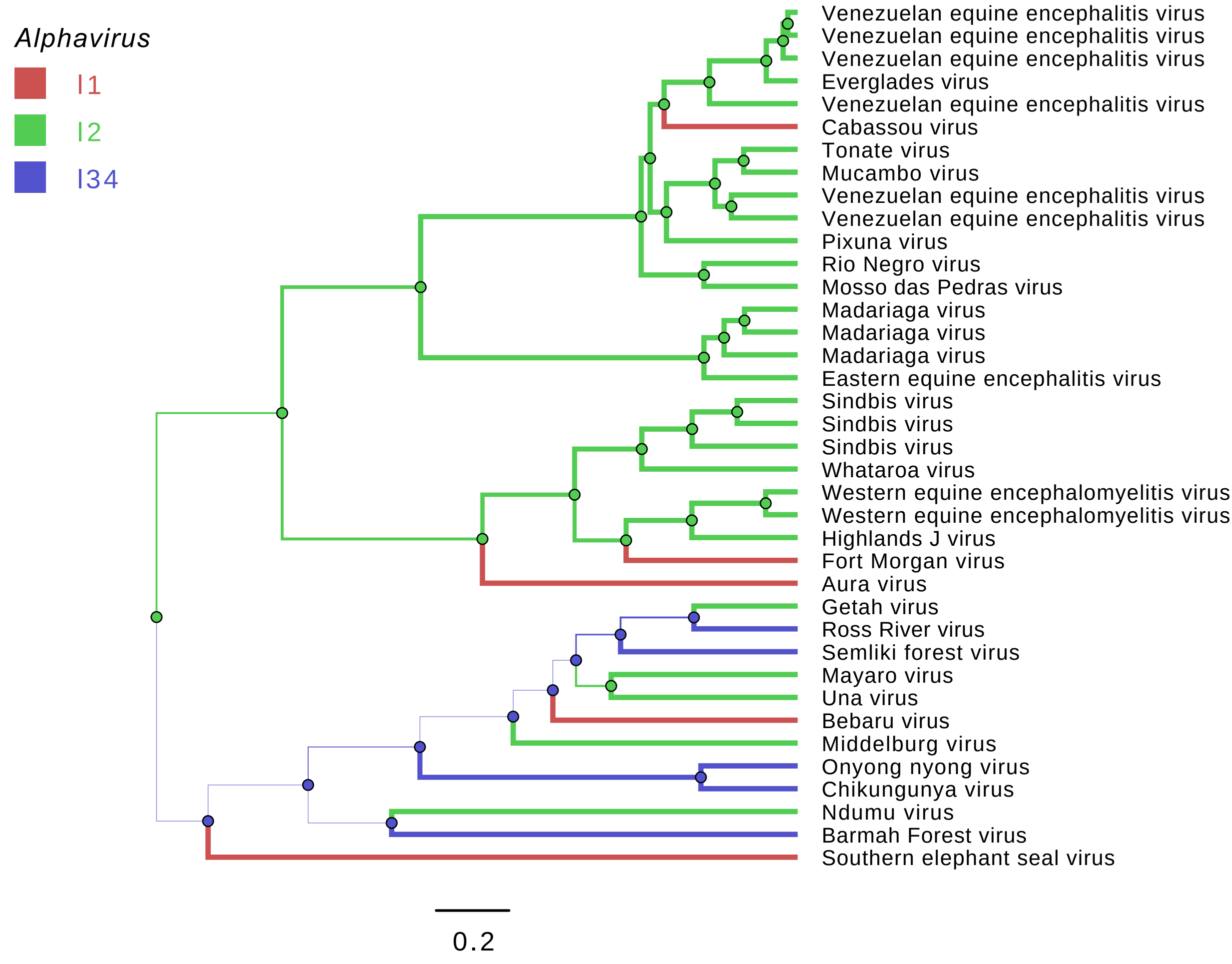

Alphavirus
I1
I2
I34
Venezuelan equine encephalitis virus
Venezuelan equine encephalitis virus
Venezuelan equine encephalitis virus
Everglades virus
Venezuelan equine encephalitis virus
Cabassou virus
Tonate virus
Mucambo virus
Venezuelan equine encephalitis virus
Venezuelan equine encephalitis virus
Pixuna virus
Rio Negro virus
Mosso das Pedras virus
Madariaga virus
Madariaga virus
Madariaga virus
Eastern equine encephalitis virus
Sindbis virus
Sindbis virus
Sindbis virus
Whataroa virus
Western equine encephalomyelitis virus
Western equine encephalomyelitis virus
Highlands J virus
Fort Morgan virus
Aura virus
Getah virus
Ross River virus
Semliki forest virus
Mayaro virus
Una virus
Bebaru virus
Middelburg virus
Onyong nyong virus
Chikungunya virus
Ndumu virus
Barmah Forest virus
Southern elephant seal virus
0.2

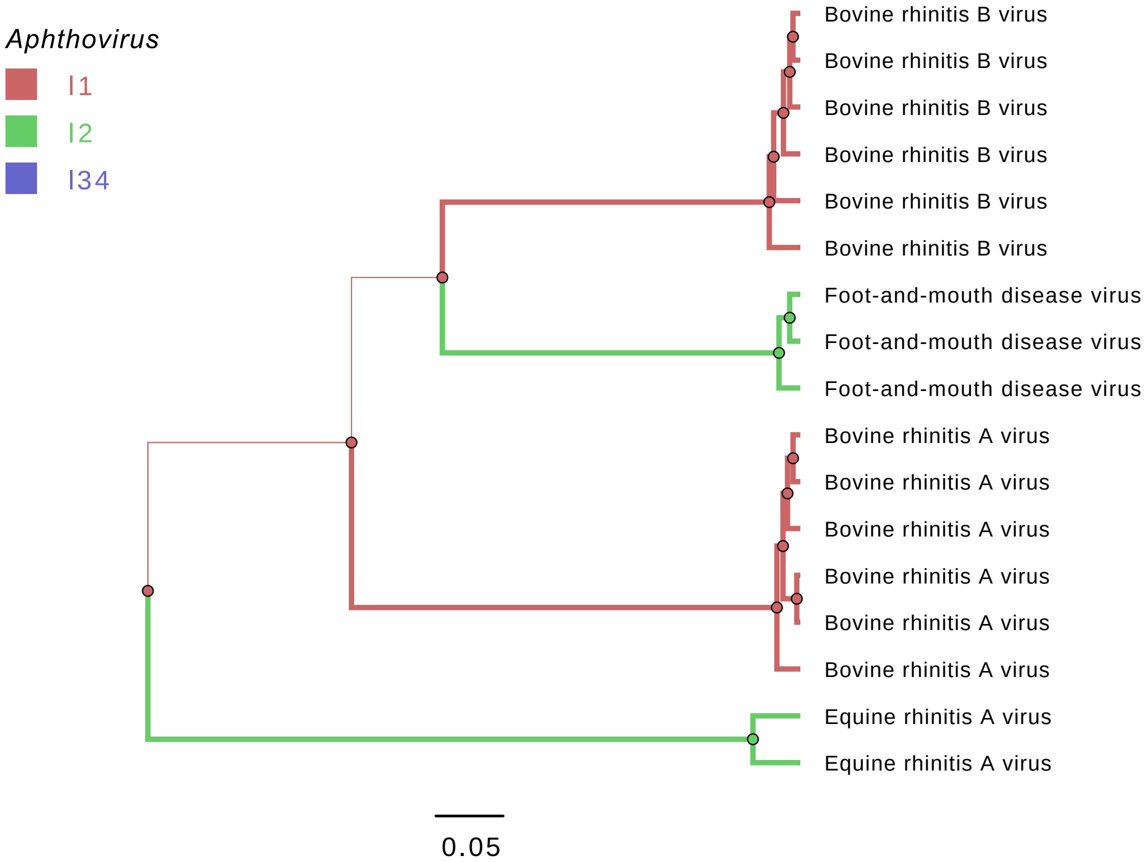
Aphthovirus
I1
I2
I34
Bovine rhinitis B virus
Bovine rhinitis B virus
Bovine rhinitis B virus
Bovine rhinitis B virus
Bovine rhinitis B virus
Bovine rhinitis B virus
Foot-and-mouth disease virus
Foot-and-mouth disease virus
Foot-and-mouth disease virus
Bovine rhinitis A virus
Bovine rhinitis A virus
Bovine rhinitis A virus
Bovine rhinitis A virus
Bovine rhinitis A virus
Bovine rhinitis A virus
Equine rhinitis A virus
Equine rhinitis A virus
0.05

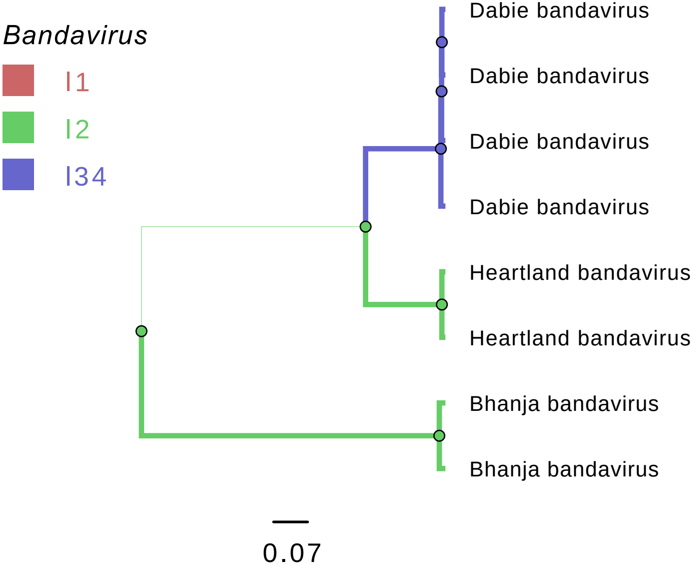
Bandavirus
I1
I2
I34
Dabie bandavirus
Dabie bandavirus
Dabie bandavirus
Dabie bandavirus
Heartland bandavirus
Heartland bandavirus
Bhanja bandavirus
Bhanja bandavirus
0.07

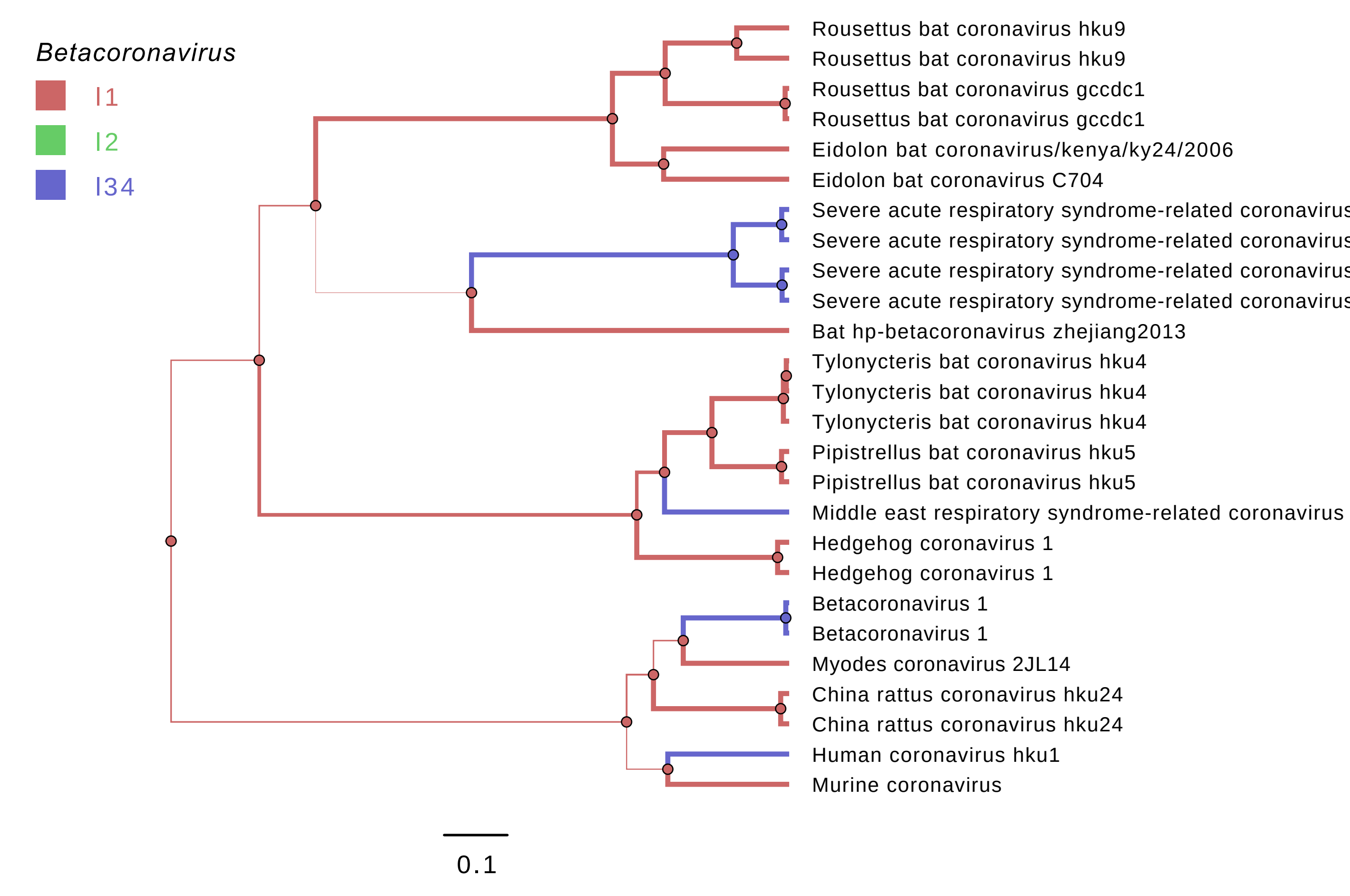

Betacoronavirus
l1
l2
l34
Rousettus bat coronavirus hku9
Rousettus bat coronavirus hku9
Rousettus bat coronavirus gccdc1
Rousettus bat coronavirus gccdc1
Eidolon bat coronavirus/kenya/ky24/2006
Eidolon bat coronavirus C704
Severe acute respiratory syndrome-related coronavirus
Severe acute respiratory syndrome-related coronavirus
Severe acute respiratory syndrome-related coronavirus
Severe acute respiratory syndrome-related coronavirus
Bat hp-betacoronavirus zhejiang2013
Tylonycteris bat coronavirus hku4
Tylonycteris bat coronavirus hku4
Tylonycteris bat coronavirus hku4
Pipistrellus bat coronavirus hku5
Pipistrellus bat coronavirus hku5
Middle east respiratory syndrome-related coronavirus
Hedgehog coronavirus 1
Hedgehog coronavirus 1
Betacoronavirus 1
Betacoronavirus 1
Myodes coronavirus 2JL14
China rattus coronavirus hku24
China rattus coronavirus hku24
Human coronavirus hku1
Murine coronavirus
0.1

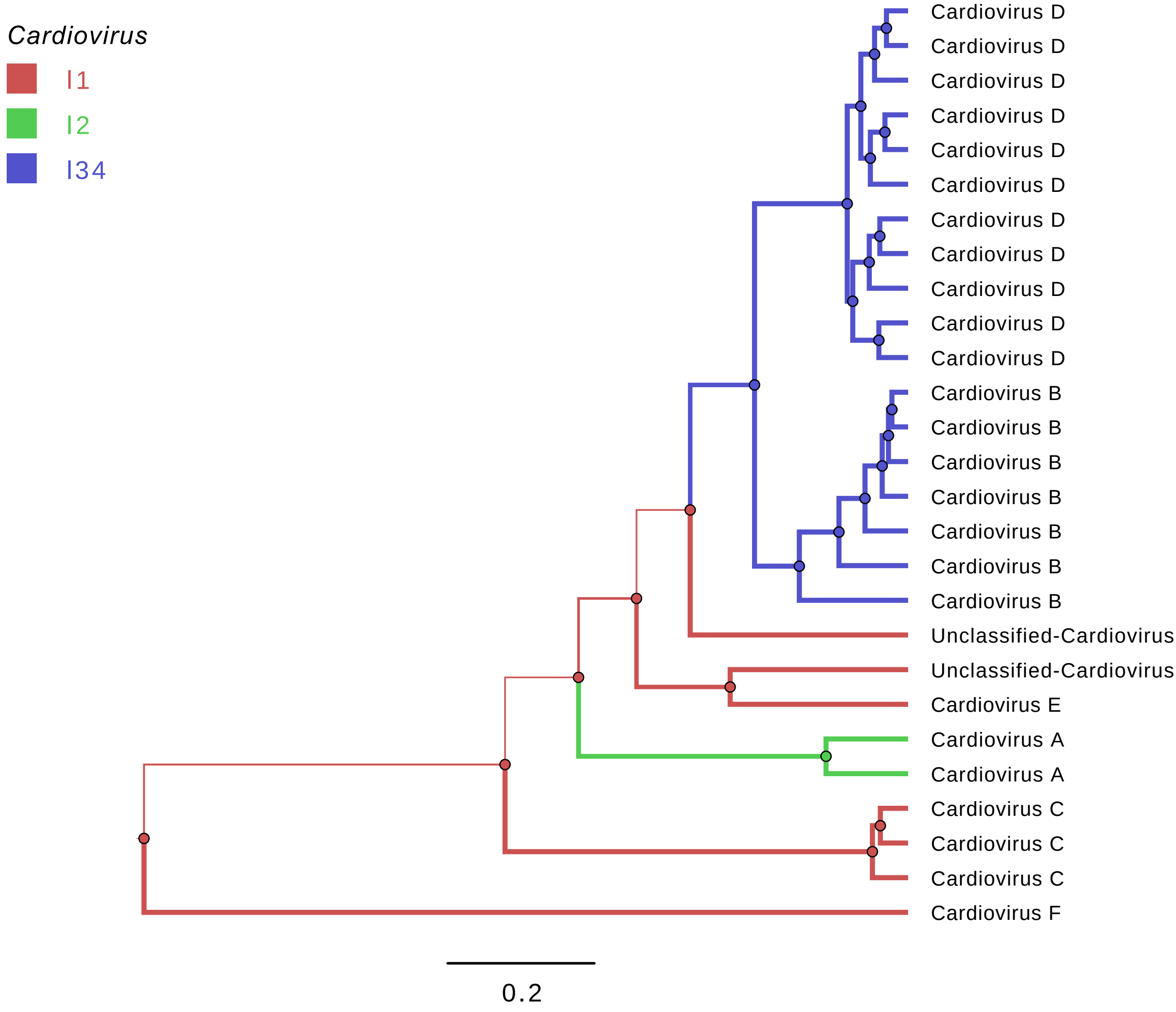

Cardiovirus
I1
I2
I34
Cardiovirus D
Cardiovirus D
Cardiovirus D
Cardiovirus D
Cardiovirus D
Cardiovirus D
Cardiovirus D
Cardiovirus D
Cardiovirus D
Cardiovirus D
Cardiovirus D
Cardiovirus B
Cardiovirus B
Cardiovirus B
Cardiovirus B
Cardiovirus B
Cardiovirus B
Cardiovirus B
Unclassified-Cardiovirus
Unclassified-Cardiovirus
Cardiovirus E
Cardiovirus A
Cardiovirus A
Cardiovirus C
Cardiovirus C
Cardiovirus C
Cardiovirus F
0.2

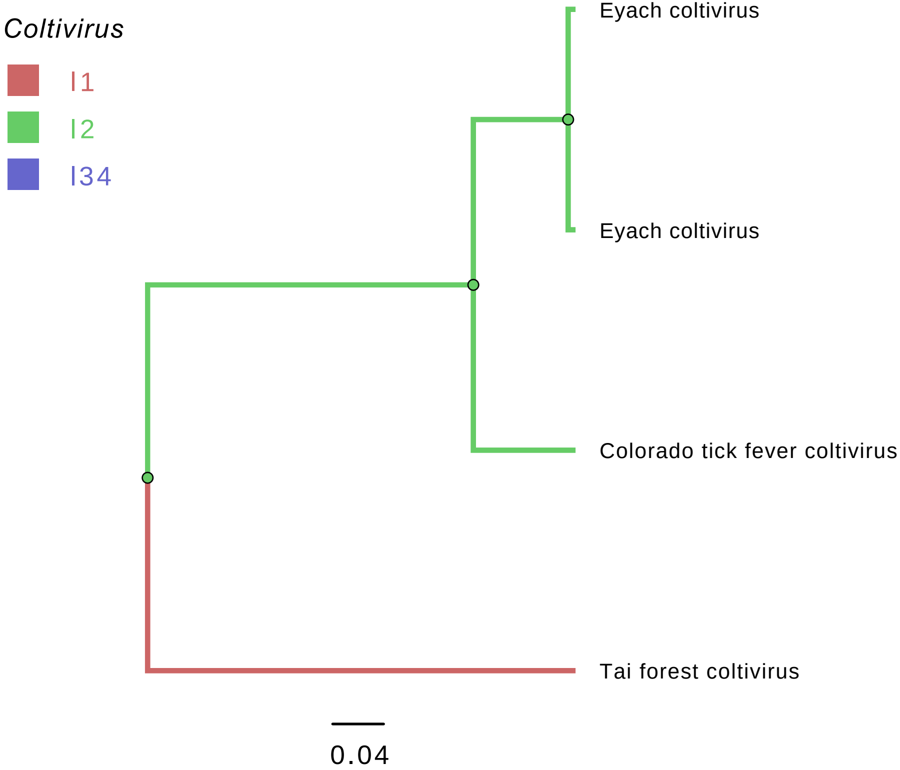

Coltivirus
I1
I2
I34
Eyach coltivirus
Eyach coltivirus
Colorado tick fever coltivirus
Tai forest coltivirus
0.04

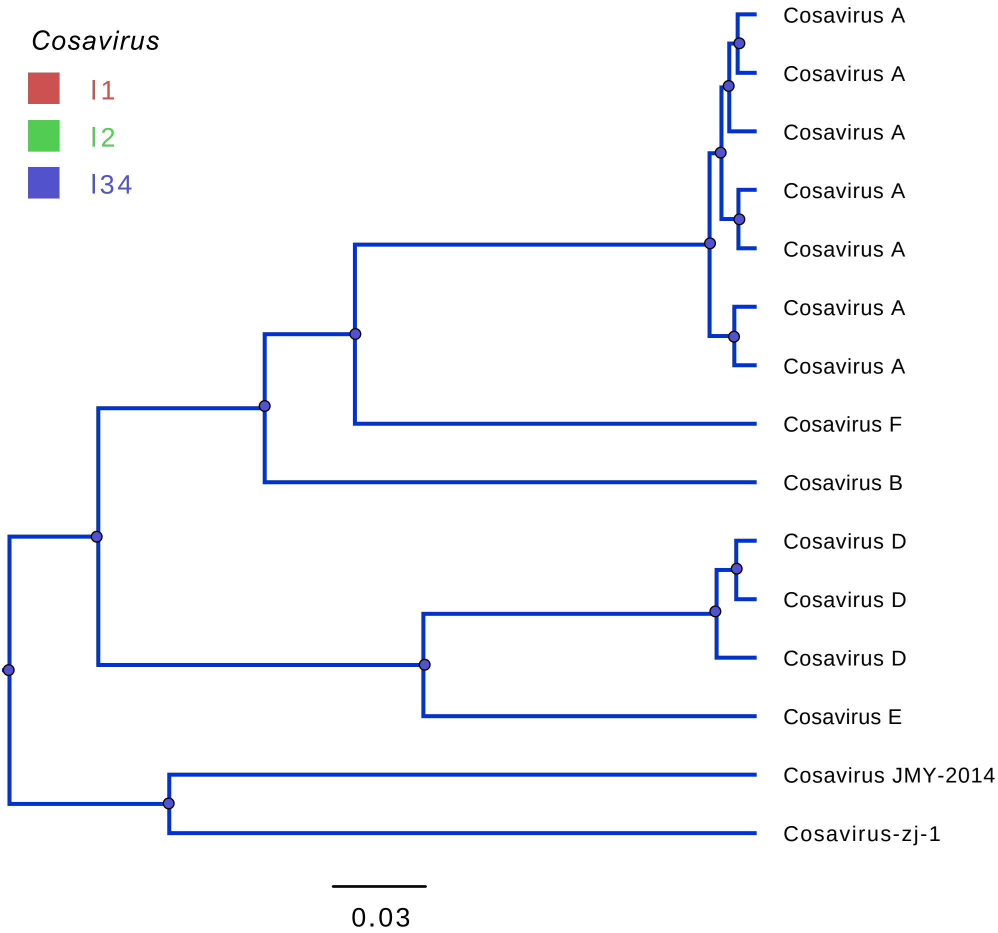
Cosavirus
I1
I2
I34
Cosavirus A
Cosavirus A
Cosavirus A
Cosavirus A
Cosavirus A
Cosavirus A
Cosavirus A
Cosavirus F
Cosavirus B
Cosavirus D
Cosavirus D
Cosavirus D
Cosavirus E
Cosavirus JMY-2014
Cosavirus-zj-1
0.03

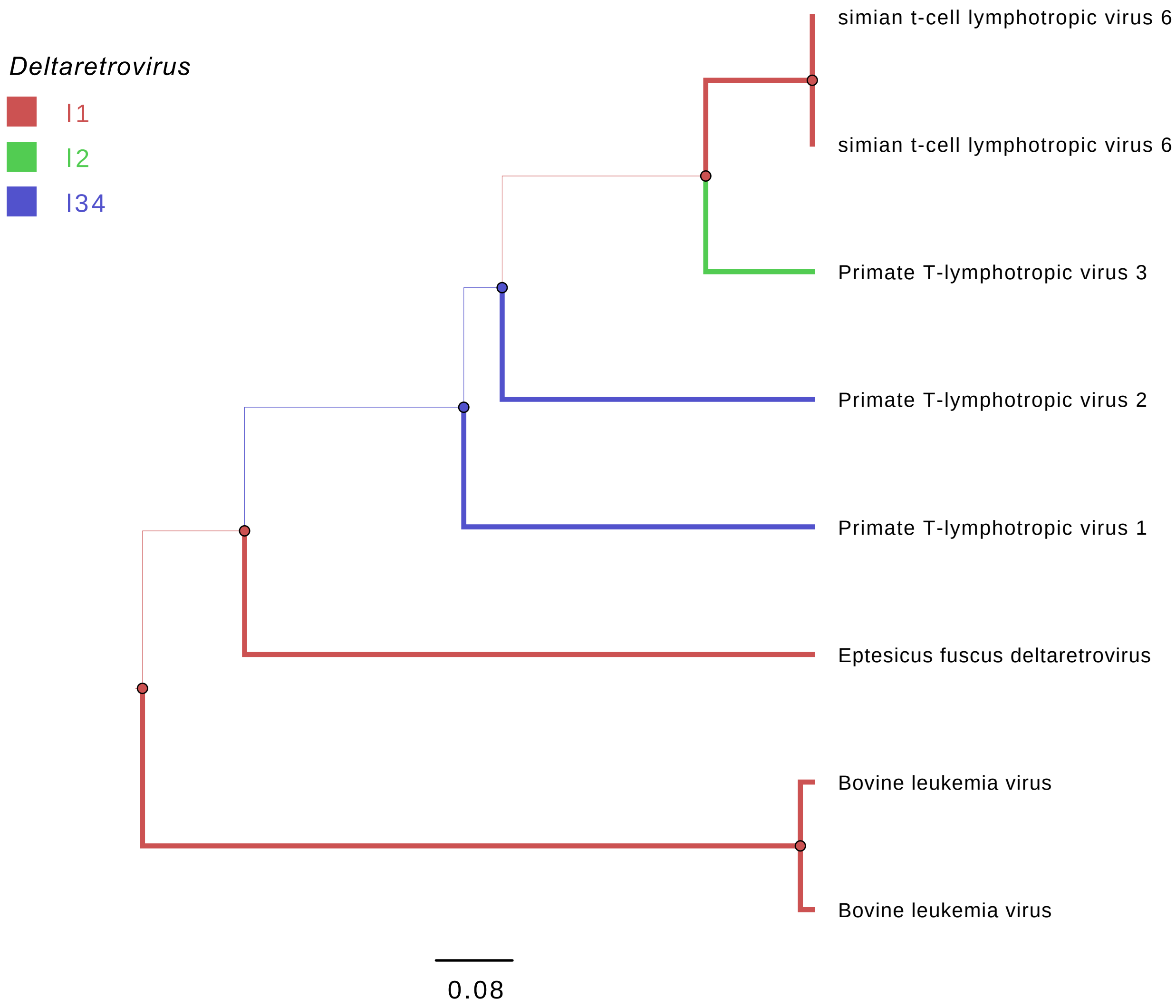
Deltaretrovirus
I1
I2
I34
simian t-cell lymphotropic virus 6
simian t-cell lymphotropic virus 6
Primate T-lymphotropic virus 3
Primate T-lymphotropic virus 2
Primate T-lymphotropic virus 1
Eptesicus fuscus deltaretrovirus
Bovine leukemia virus
Bovine leukemia virus
0.08

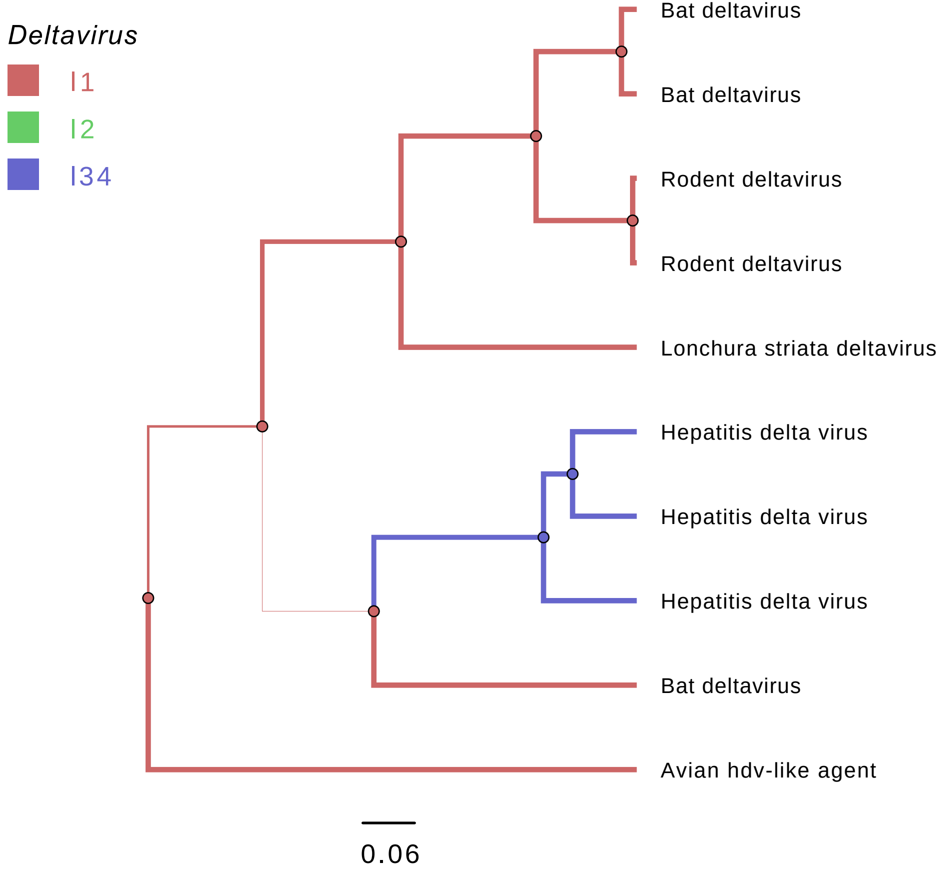

Deltavirus
I1
I2
I34
Bat deltavirus
Bat deltavirus
Rodent deltavirus
Rodent deltavirus
Lonchura striata deltavirus
Hepatitis delta virus
Hepatitis delta virus
Hepatitis delta virus
Bat deltavirus
Avian hdv-like agent
0.06

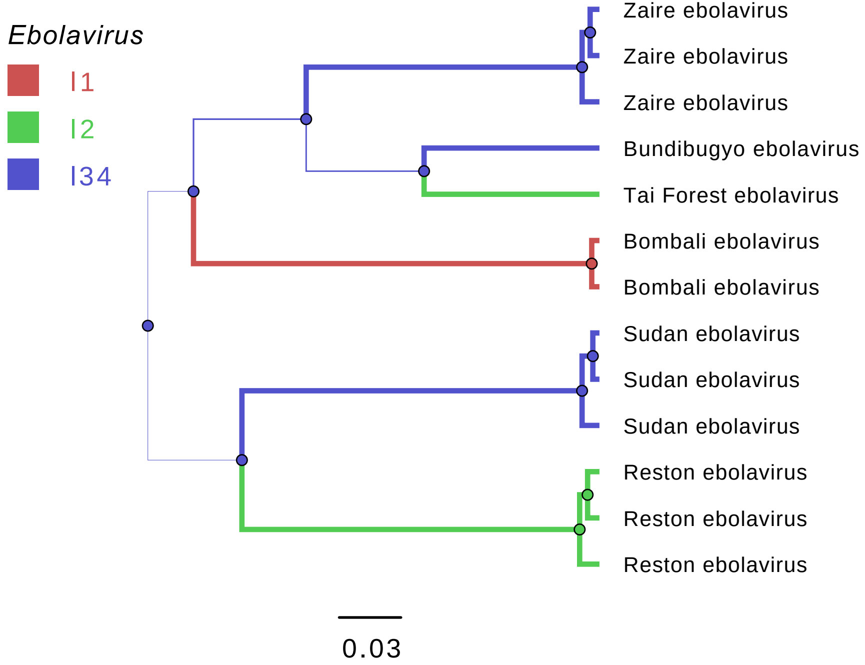
Ebolavirus
I1
I2
I34
Zaire ebolavirus
Zaire ebolavirus
Zaire ebolavirus
Bundibugyo ebolavirus
Tai Forest ebolavirus
Bombali ebolavirus
Bombali ebolavirus
Sudan ebolavirus
Sudan ebolavirus
Sudan ebolavirus
Reston ebolavirus
Reston ebolavirus
Reston ebolavirus
0.03

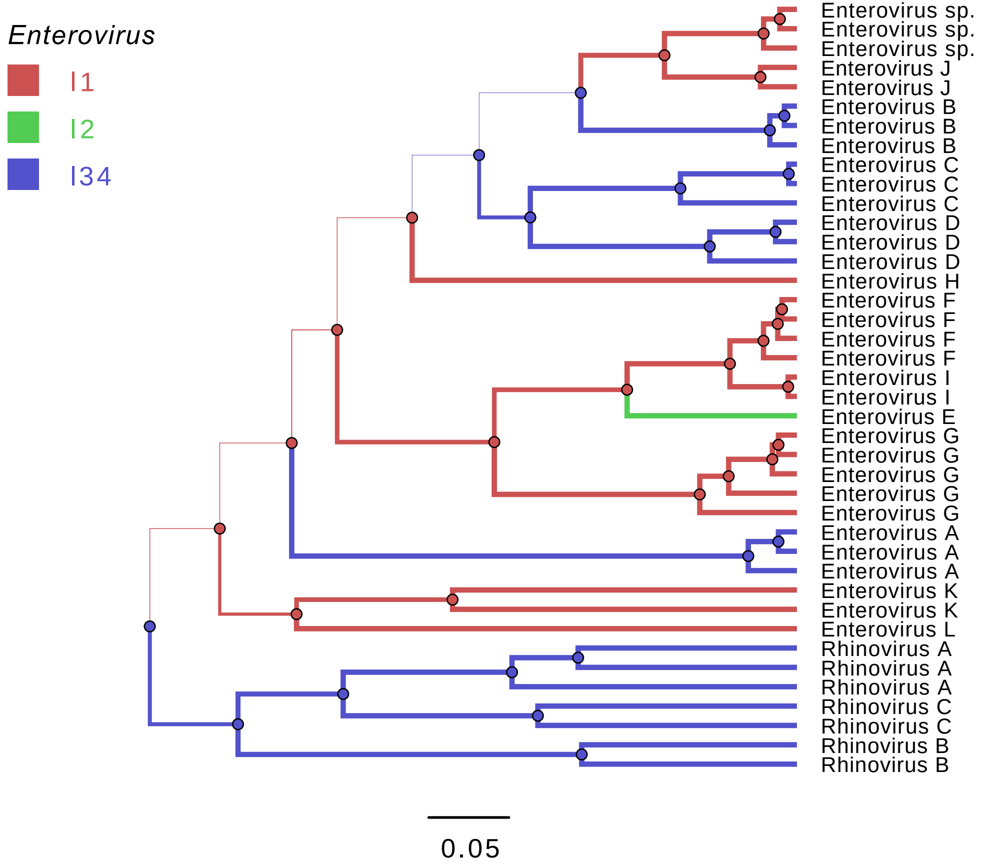

Enterovirus
I1
I2
I34
Enterovirus sp.
Enterovirus sp.
Enterovirus sp.
Enterovirus J
Enterovirus J
Enterovirus B
Enterovirus B
Enterovirus B
Enterovirus C
Enterovirus C
Enterovirus C
Enterovirus D
Enterovirus D
Enterovirus D
Enterovirus H
Enterovirus F
Enterovirus F
Enterovirus F
Enterovirus F
Enterovirus I
Enterovirus I
Enterovirus E
Enterovirus G
Enterovirus G
Enterovirus G
Enterovirus G
Enterovirus G
Enterovirus A
Enterovirus A
Enterovirus A
Enterovirus K
Enterovirus K
Enterovirus L
Rhinovirus A
Rhinovirus A
Rhinovirus A
Rhinovirus C
Rhinovirus C
Rhinovirus B
Rhinovirus B
0.05

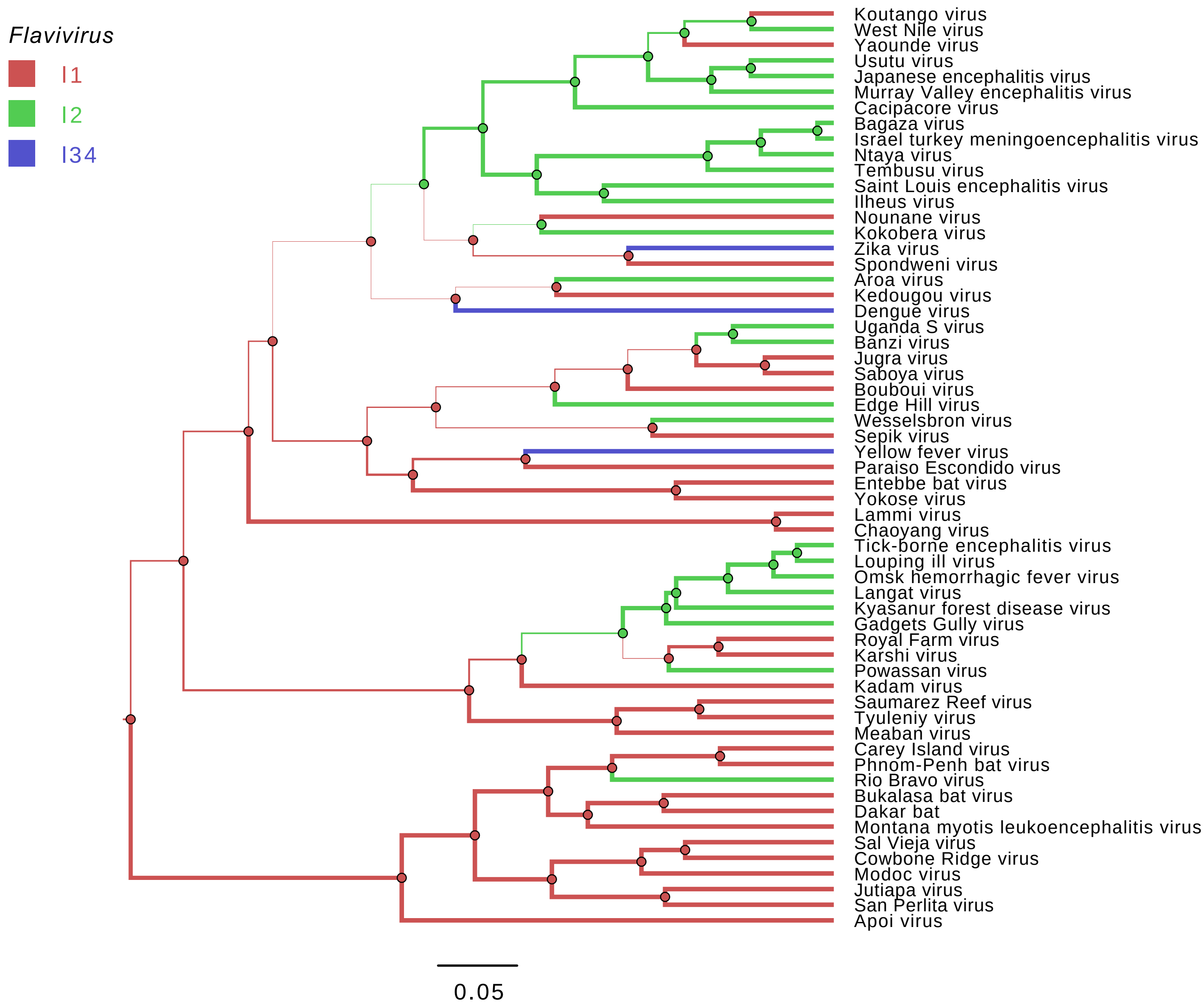
Flavivirus
I1
I2
I34
Koutango virus
West Nile virus
Yaounde virus
Usutu virus
Japanese encephalitis virus
Murray Valley encephalitis virus
Cacipacore virus
Bagaza virus
Israel turkey meningoencephalitis virus
Ntaya virus
Tembusu virus
Saint Louis encephalitis virus
Ilheus virus
Nounane virus
Kokobera virus
Zika virus
Spondweni virus
Aroa virus
Kedougou virus
Dengue virus
Uganda S virus
Banzi virus
Jugra virus
Saboya virus
Bouboui virus
Edge Hill virus
Wesselsbron virus
Sepik virus
Yellow fever virus
Paraiso Escondido virus
Entebbe bat virus
Yokose virus
Lammi virus
Chaoyang virus
Tick-borne encephalitis virus
Louping ill virus
Omsk hemorrhagic fever virus
Langat virus
Kyasanur forest disease virus
Gadgets Gully virus
Royal Farm virus
Karshi virus
Powassan virus
Kadam virus
Saumarez Reef virus
Tyuleniy virus
Meaban virus
Carey Island virus
Phnom-Penh bat virus
Rio Bravo virus
Bukalasa bat virus
Dakar bat
Montana myotis leukoencephalitis virus
Sal Vieja virus
Cowbone Ridge virus
Modoc virus
Jutiapa virus
San Perlita virus
Apoi virus
0.05

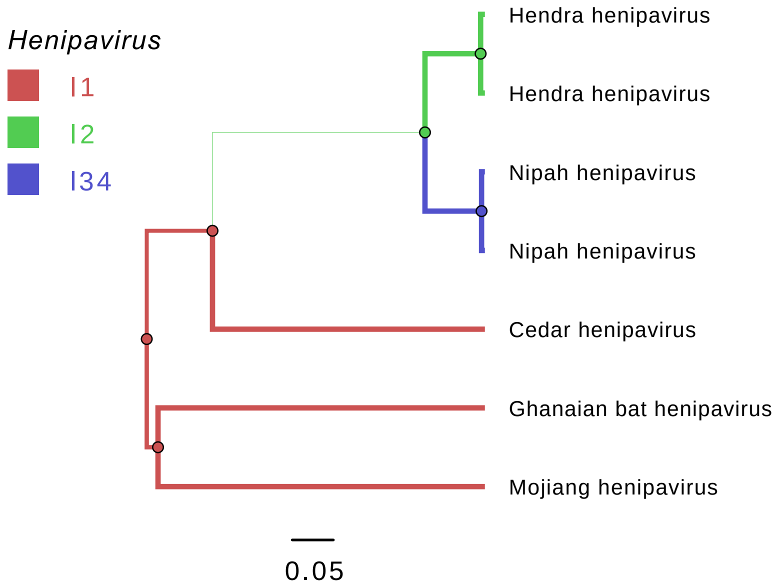
Henipavirus
I1
I2
I34
Hendra henipavirus
Hendra henipavirus
Nipah henipavirus
Nipah henipavirus
Cedar henipavirus
Ghanaian bat henipavirus
Mojiang henipavirus
0.05

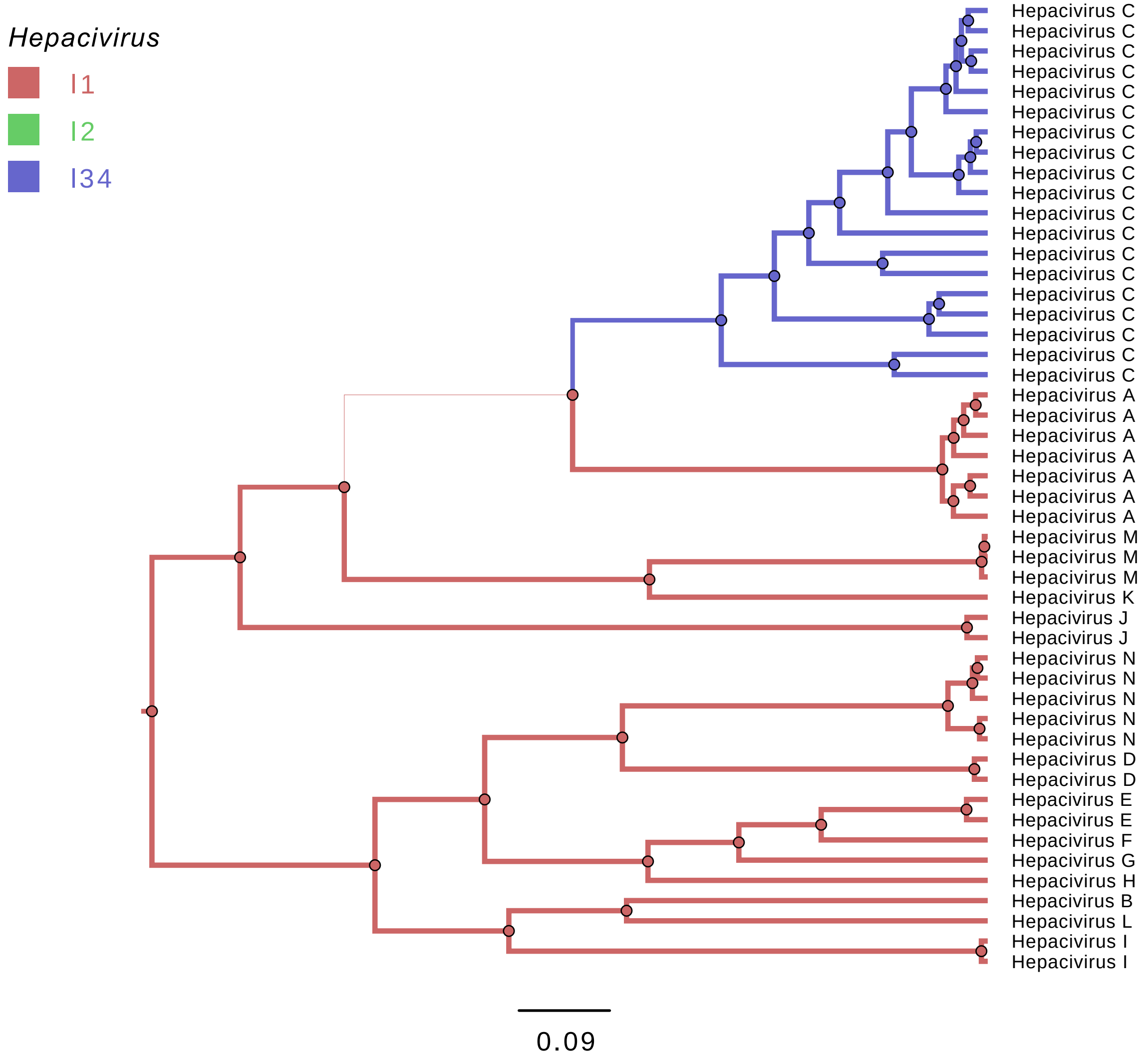
Hepacivirus
I1
I2
I34
Hepacivirus C
Hepacivirus C
Hepacivirus C
Hepacivirus C
Hepacivirus C
Hepacivirus C
Hepacivirus C
Hepacivirus C
Hepacivirus C
Hepacivirus C
Hepacivirus C
Hepacivirus C
Hepacivirus C
Hepacivirus C
Hepacivirus C
Hepacivirus C
Hepacivirus C
Hepacivirus C
Hepacivirus C
Hepacivirus A
Hepacivirus A
Hepacivirus A
Hepacivirus A
Hepacivirus A
Hepacivirus A
Hepacivirus A
Hepacivirus M
Hepacivirus M
Hepacivirus M
Hepacivirus K
Hepacivirus J
Hepacivirus J
Hepacivirus N
Hepacivirus N
Hepacivirus N
Hepacivirus N
Hepacivirus N
Hepacivirus D
Hepacivirus D
Hepacivirus E
Hepacivirus E
Hepacivirus F
Hepacivirus G
Hepacivirus H
Hepacivirus B
Hepacivirus L
Hepacivirus I
Hepacivirus I
0.09

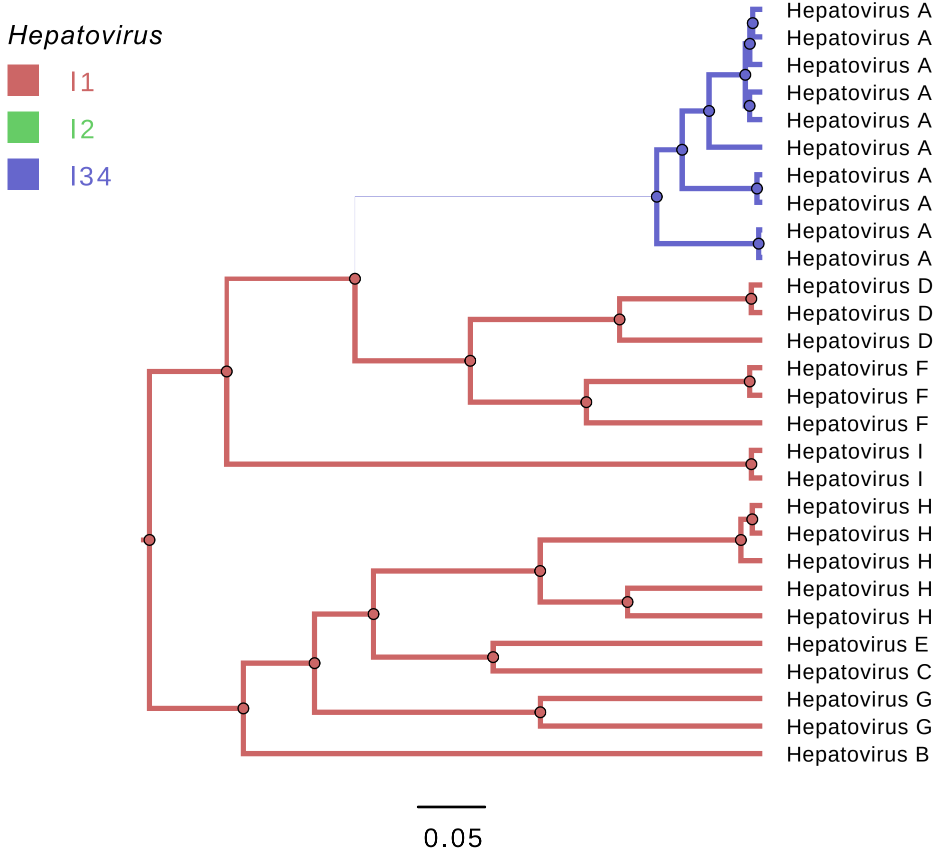
Hepatovirus
I1
I2
I34
Hepatovirus A
Hepatovirus A
Hepatovirus A
Hepatovirus A
Hepatovirus A
Hepatovirus A
Hepatovirus A
Hepatovirus A
Hepatovirus A
Hepatovirus A
Hepatovirus D
Hepatovirus D
Hepatovirus D
Hepatovirus F
Hepatovirus F
Hepatovirus F
Hepatovirus I
Hepatovirus I
Hepatovirus H
Hepatovirus H
Hepatovirus H
Hepatovirus H
Hepatovirus H
Hepatovirus E
Hepatovirus C
Hepatovirus G
Hepatovirus G
Hepatovirus B
0.05

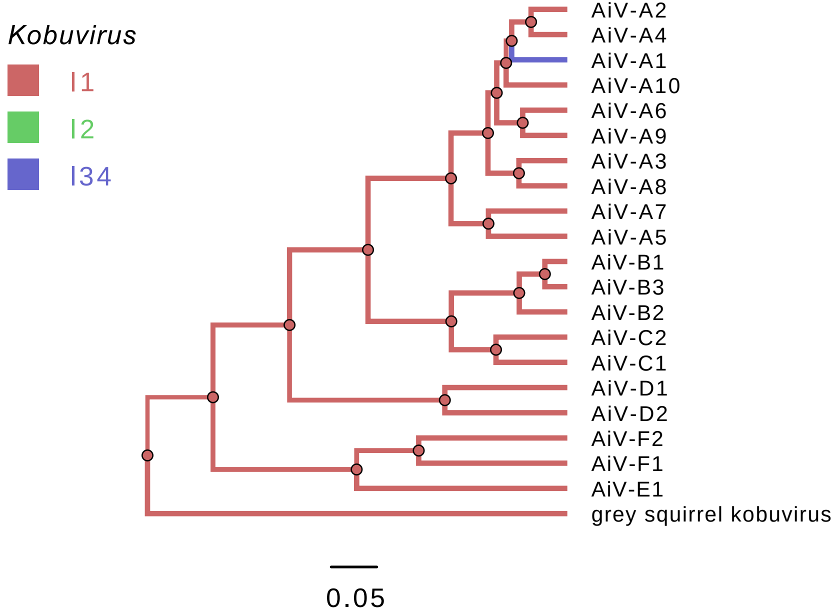
Kobuvirus
I1
I2
I34
AiV-A2
AiV-A4
AiV-A1
AiV-A10
AiV-A6
AiV-A9
AiV-A3
AiV-A8
AiV-A7
AiV-A5
AiV-B1
AiV-B3
AiV-B2
AiV-C2
AiV-C1
AiV-D1
AiV-D2
AiV-F2
AiV-F1
AiV-E1
grey squirrel kobuvirus
0.05

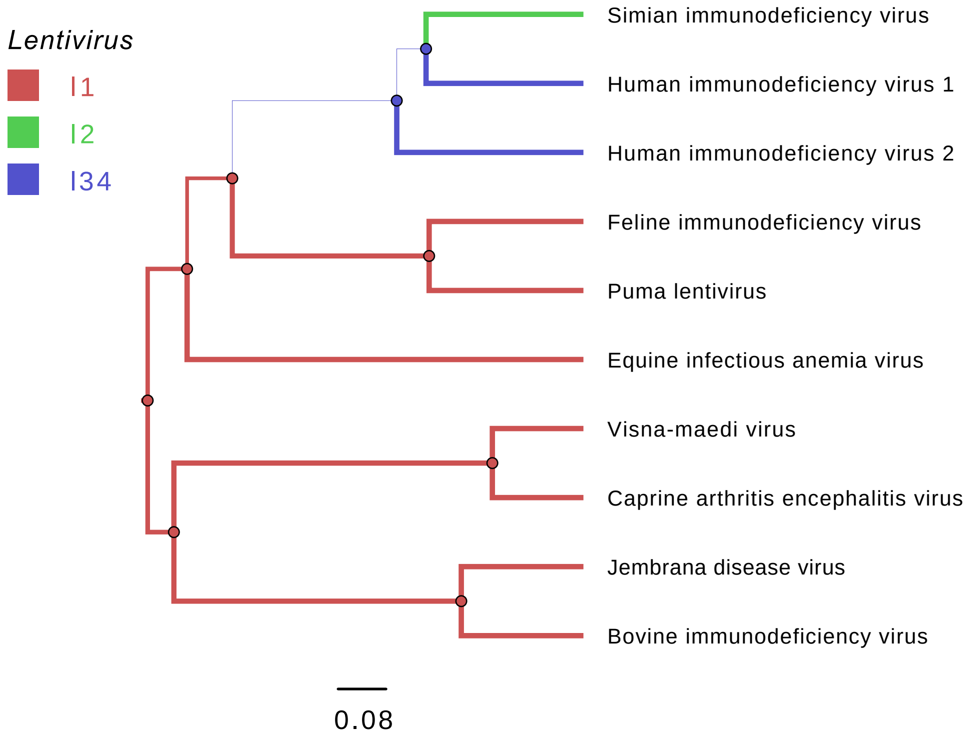
Lentivirus
I1
I2
I34
Simian immunodeficiency virus
Human immunodeficiency virus 1
Human immunodeficiency virus 2
Feline immunodeficiency virus
Puma lentivirus
Equine infectious anemia virus
Visna-maedi virus
Caprine arthritis encephalitis virus
Jembrana disease virus
Bovine immunodeficiency virus
0.08

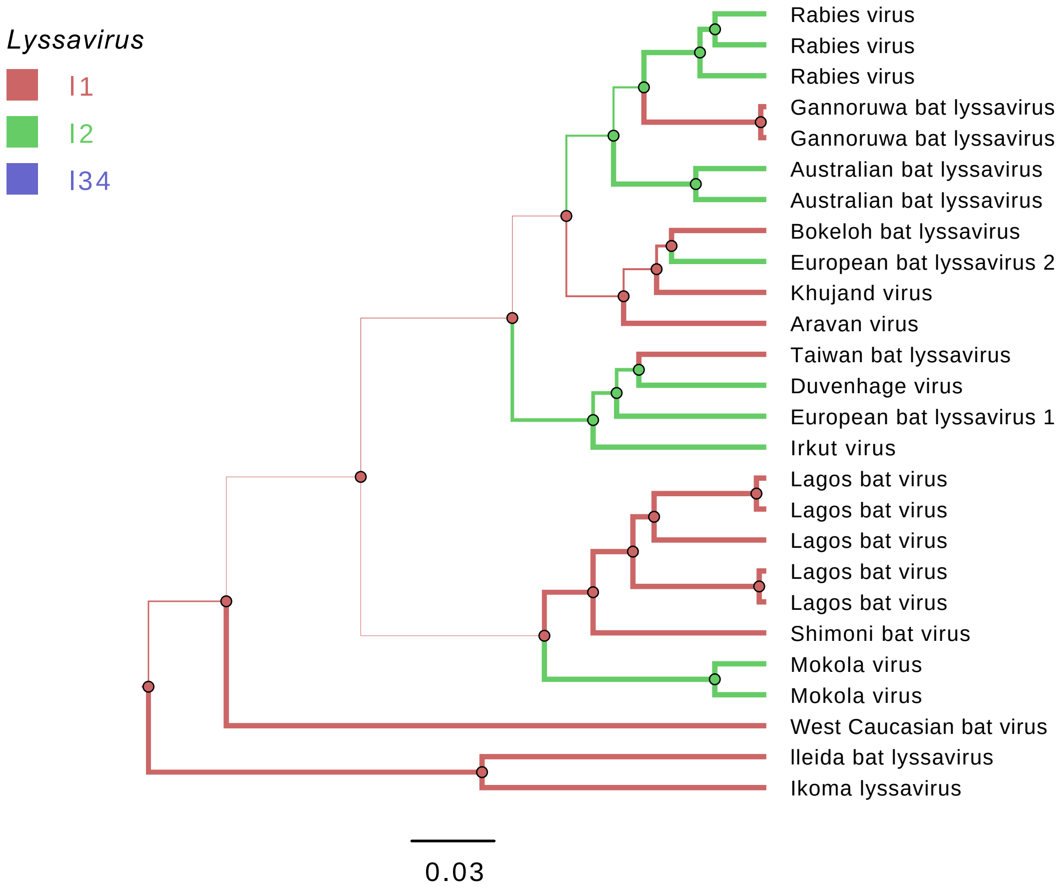
Lyssavirus
l1
l2
l34
Rabies virus
Rabies virus
Rabies virus
Gannoruwa bat lyssavirus
Gannoruwa bat lyssavirus
Australian bat lyssavirus
Australian bat lyssavirus
Bokeloh bat lyssavirus
European bat lyssavirus 2
Khujand virus
Aravan virus
Taiwan bat lyssavirus
Duvenhage virus
European bat lyssavirus 1
Irkut virus
Lagos bat virus
Lagos bat virus
Lagos bat virus
Lagos bat virus
Lagos bat virus
Shimoni bat virus
Mokola virus
Mokola virus
West Caucasian bat virus
Ileida bat lyssavirus
Ikoma lyssavirus
0.03

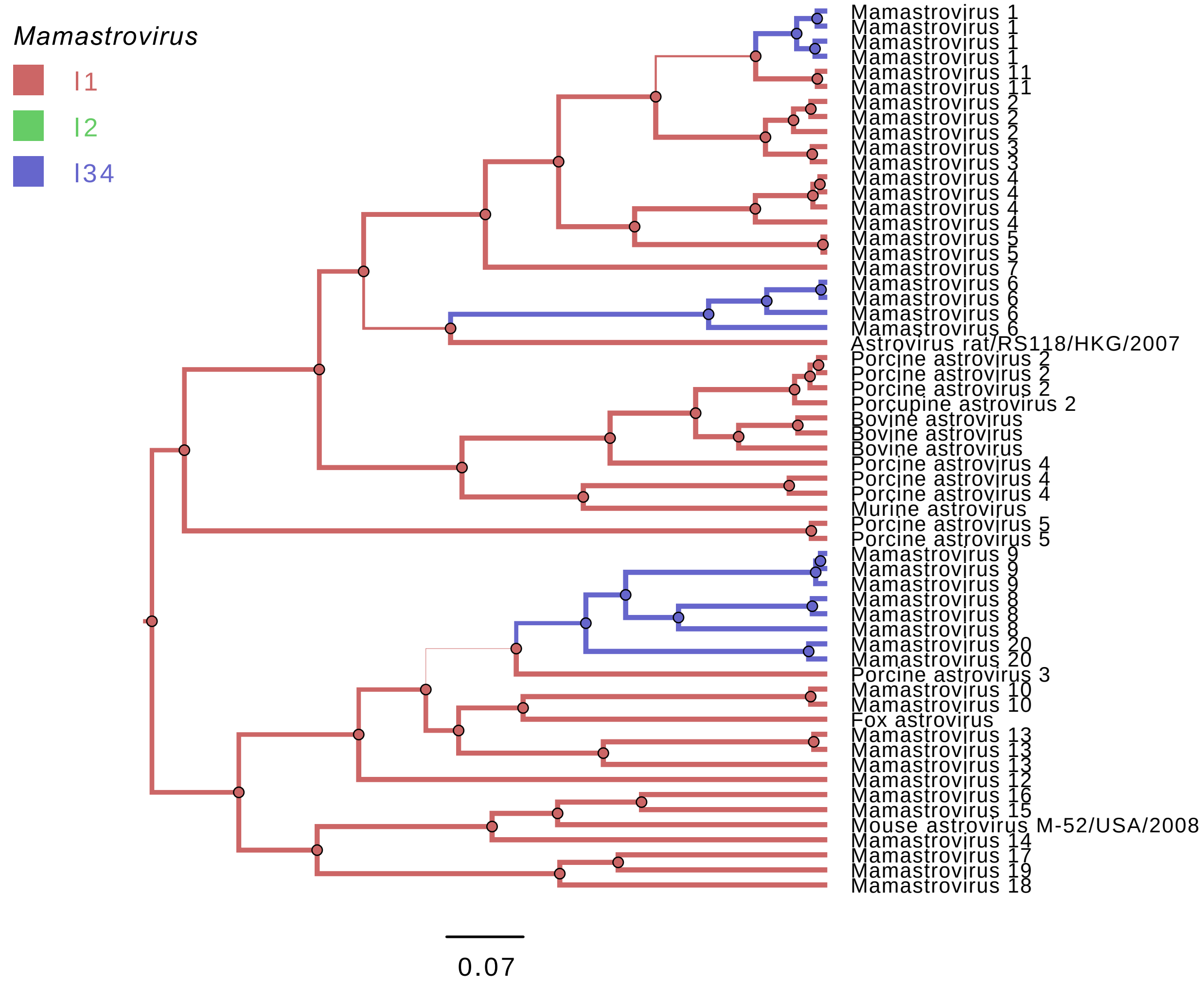

Mamastrovirus
I1
I2
I34
Mamastrovirus 1
Mamastrovirus 1
Mamastrovirus 1
Mamastrovirus 1
Mamastrovirus 11
Mamastrovirus 11
Mamastrovirus 2
Mamastrovirus 2
Mamastrovirus 2
Mamastrovirus 3
Mamastrovirus 3
Mamastrovirus 4
Mamastrovirus 4
Mamastrovirus 4
Mamastrovirus 4
Mamastrovirus 5
Mamastrovirus 5
Mamastrovirus 7
Mamastrovirus 6
Mamastrovirus 6
Mamastrovirus 6
Mamastrovirus 6
Astrovirus rat/RS118/HKG/2007
Porcine astrovirus 2
Porcine astrovirus 2
Porcine astrovirus 2
Porcupine astrovirus 2
Bovine astrovirus
Bovine astrovirus
Bovine astrovirus
Porcine astrovirus 4
Porcine astrovirus 4
Porcine astrovirus 4
Murine astrovirus
Porcine astrovirus 5
Porcine astrovirus 5
Mamastrovirus 9
Mamastrovirus 9
Mamastrovirus 9
Mamastrovirus 8
Mamastrovirus 8
Mamastrovirus 8
Mamastrovirus 20
Mamastrovirus 20
Porcine astrovirus 3
Mamastrovirus 10
Mamastrovirus 10
Fox astrovirus
Mamastrovirus 13
Mamastrovirus 13
Mamastrovirus 13
Mamastrovirus 12
Mamastrovirus 16
Mamastrovirus 15
Mouse astrovirus M-52/USA/2008
Mamastrovirus 14
Mamastrovirus 17
Mamastrovirus 19
Mamastrovirus 18
0.07

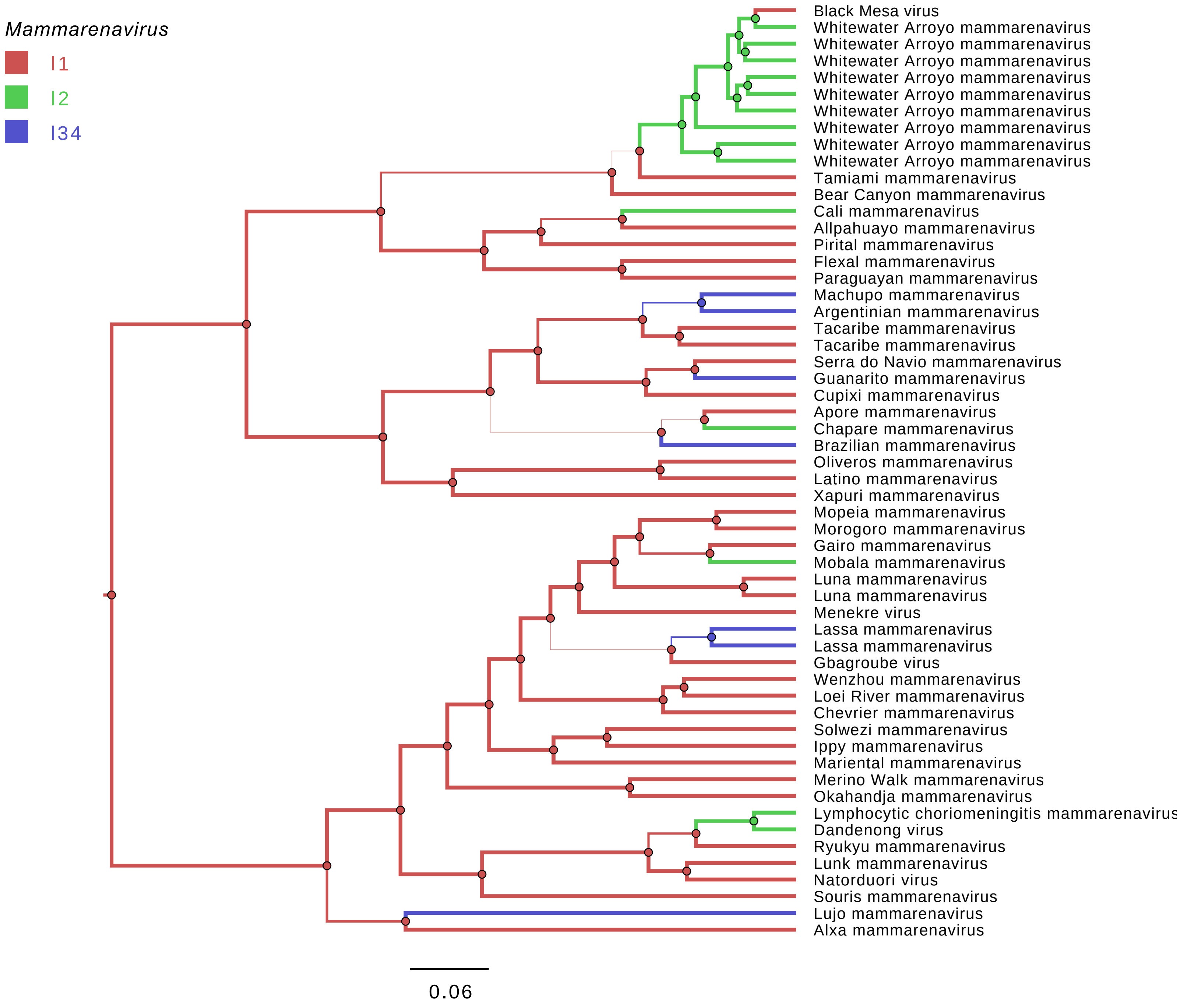
Mammarenavirus
I1
I2
I34
Black Mesa virus
Whitewater Arroyo mammarenavirus
Whitewater Arroyo mammarenavirus
Whitewater Arroyo mammarenavirus
Whitewater Arroyo mammarenavirus
Whitewater Arroyo mammarenavirus
Whitewater Arroyo mammarenavirus
Whitewater Arroyo mammarenavirus
Whitewater Arroyo mammarenavirus
Whitewater Arroyo mammarenavirus
Tamiami mammarenavirus
Bear Canyon mammarenavirus
Cali mammarenavirus
Allpahuayo mammarenavirus
Pirital mammarenavirus
Flexal mammarenavirus
Paraguayan mammarenavirus
Machupo mammarenavirus
Argentinian mammarenavirus
Tacaribe mammarenavirus
Tacaribe mammarenavirus
Serra do Navio mammarenavirus
Guanarito mammarenavirus
Cupixi mammarenavirus
Apore mammarenavirus
Chapare mammarenavirus
Brazilian mammarenavirus
Oliveros mammarenavirus
Latino mammarenavirus
Xapuri mammarenavirus
Mopeia mammarenavirus
Morogoro mammarenavirus
Gairo mammarenavirus
Mobala mammarenavirus
Luna mammarenavirus
Luna mammarenavirus
Menekre virus
Lassa mammarenavirus
Lassa mammarenavirus
Gbagroube virus
Wenzhou mammarenavirus
Loei River mammarenavirus
Chevrier mammarenavirus
Solwezi mammarenavirus
Ippy mammarenavirus
Mariental mammarenavirus
Merino Walk mammarenavirus
Okahandja mammarenavirus
Lymphocytic choriomeningitis mammarenavirus
Dandenong virus
Ryukyu mammarenavirus
Lunk mammarenavirus
Natorduori virus
Souris mammarenavirus
Lujo mammarenavirus
Alxa mammarenavirus
0.06

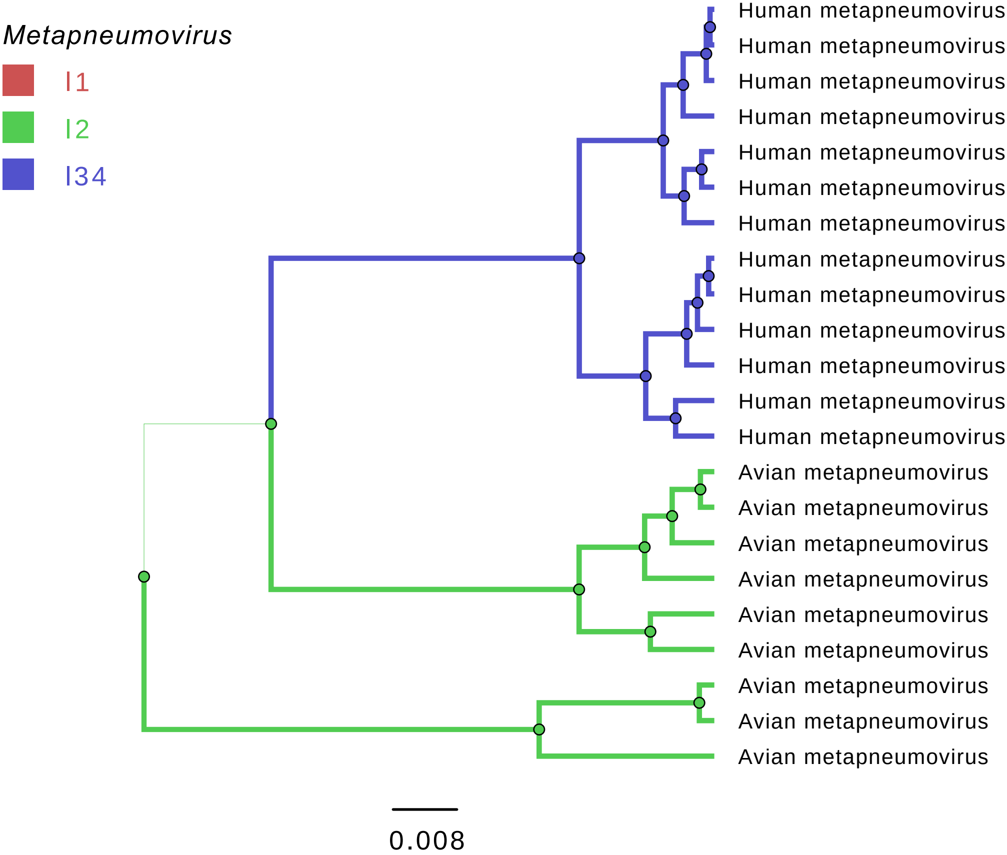
Metapneumovirus
I1
I2
I34
Human metapneumovirus
Human metapneumovirus
Human metapneumovirus
Human metapneumovirus
Human metapneumovirus
Human metapneumovirus
Human metapneumovirus
Human metapneumovirus
Human metapneumovirus
Human metapneumovirus
Human metapneumovirus
Human metapneumovirus
Human metapneumovirus
Avian metapneumovirus
Avian metapneumovirus
Avian metapneumovirus
Avian metapneumovirus
Avian metapneumovirus
Avian metapneumovirus
Avian metapneumovirus
Avian metapneumovirus
Avian metapneumovirus
0.008

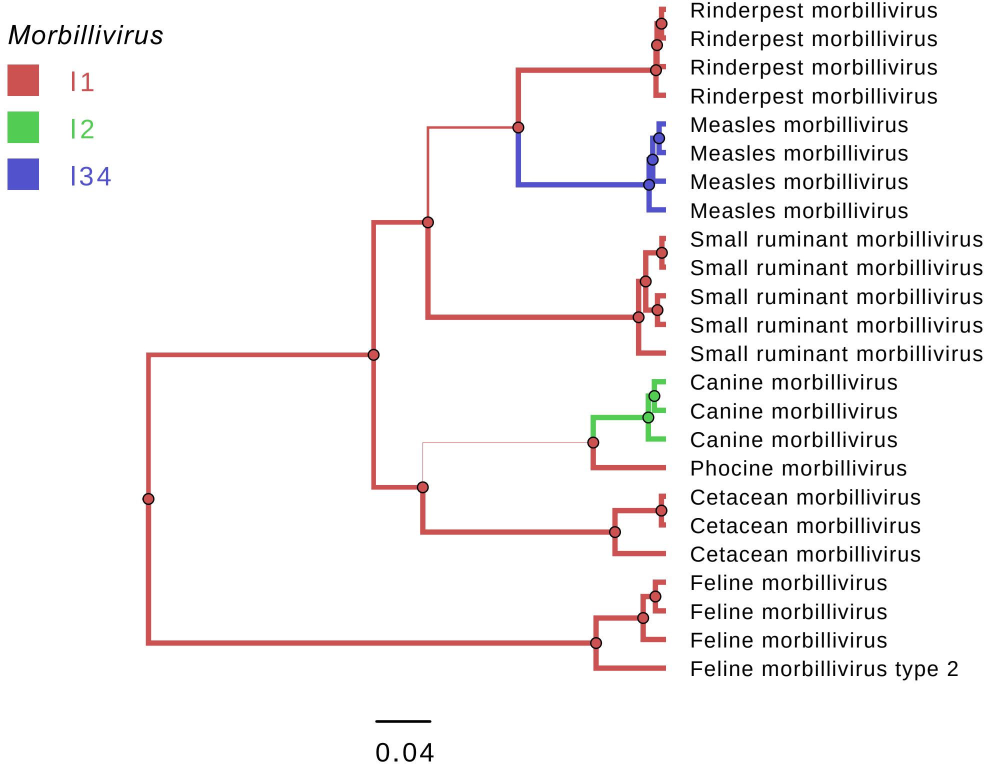

Morbillivirus
I1
I2
I34
Rinderpest morbillivirus
Rinderpest morbillivirus
Rinderpest morbillivirus
Rinderpest morbillivirus
Measles morbillivirus
Measles morbillivirus
Measles morbillivirus
Measles morbillivirus
Small ruminant morbillivirus
Small ruminant morbillivirus
Small ruminant morbillivirus
Small ruminant morbillivirus
Small ruminant morbillivirus
Canine morbillivirus
Canine morbillivirus
Canine morbillivirus
Phocine morbillivirus
Cetacean morbillivirus
Cetacean morbillivirus
Cetacean morbillivirus
Feline morbillivirus
Feline morbillivirus
Feline morbillivirus
Feline morbillivirus type 2
0.04

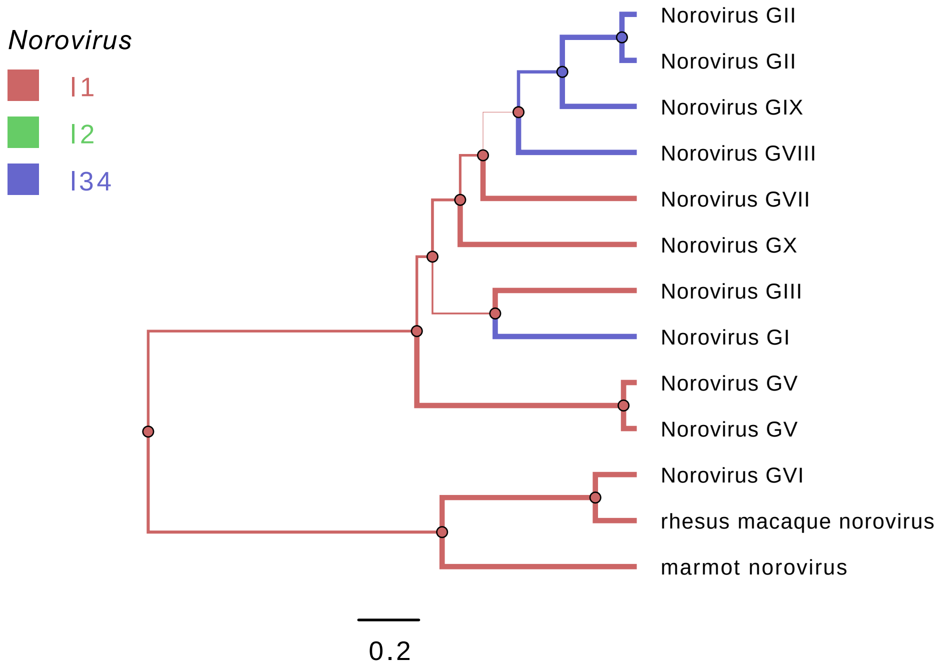
Norovirus
I1
I2
I34
Norovirus GII
Norovirus GII
Norovirus GIX
Norovirus GVIII
Norovirus GVII
Norovirus GX
Norovirus GIII
Norovirus GI
Norovirus GV
Norovirus GV
Norovirus GVI
rhesus macaque norovirus
marmot norovirus
0.2

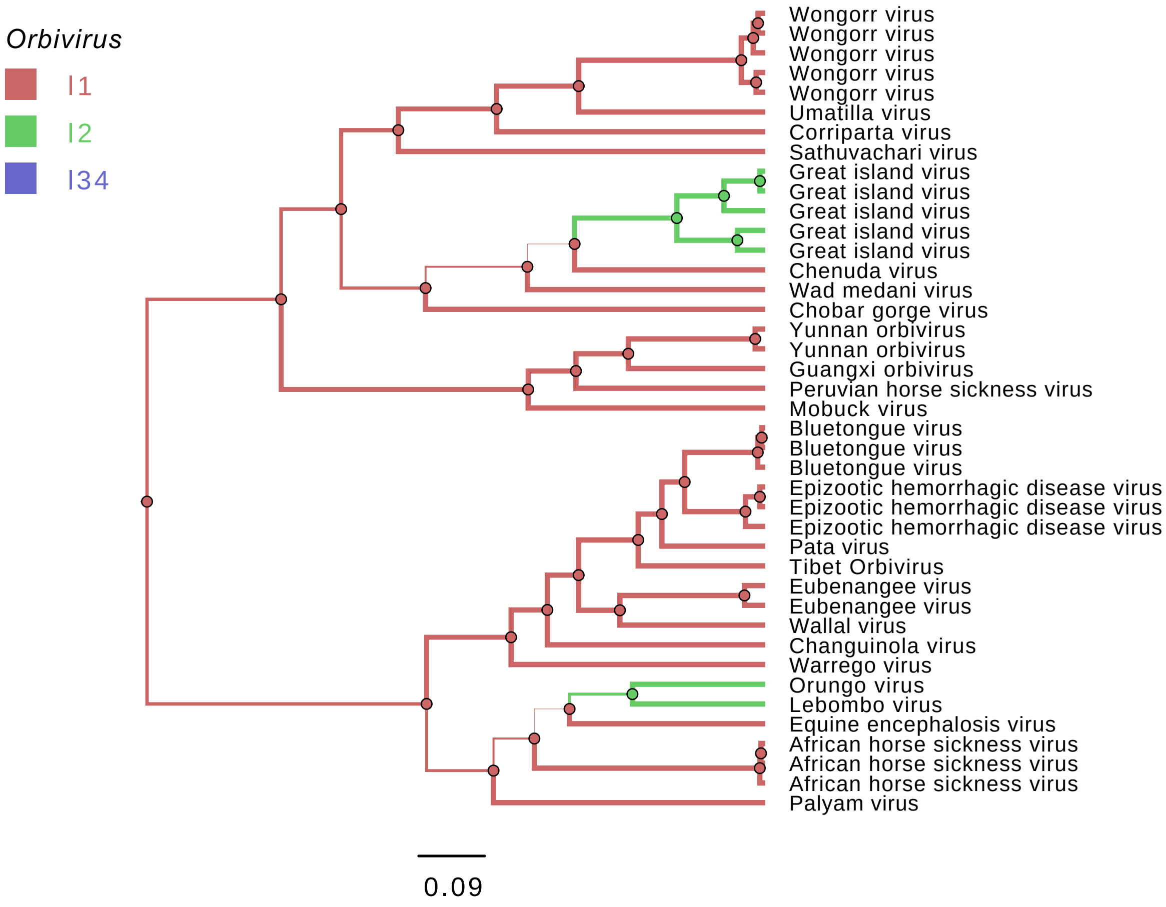
Orbivirus
I1
I2
I34
Wongorr virus
Wongorr virus
Wongorr virus
Wongorr virus
Wongorr virus
Umatilla virus
Corriparta virus
Sathuvachari virus
Great island virus
Great island virus
Great island virus
Great island virus
Great island virus
Chenuda virus
Wad medani virus
Chobar gorge virus
Yunnan orbivirus
Yunnan orbivirus
Guangxi orbivirus
Peruvian horse sickness virus
Mobuck virus
Bluetongue virus
Bluetongue virus
Bluetongue virus
Epizootic hemorrhagic disease virus
Epizootic hemorrhagic disease virus
Epizootic hemorrhagic disease virus
Pata virus
Tibet Orbivirus
Eubenangee virus
Eubenangee virus
Wallal virus
Changuinola virus
Warrego virus
Orungo virus
Lebombo virus
Equine encephalosis virus
African horse sickness virus
African horse sickness virus
African horse sickness virus
Palyam virus
0.09

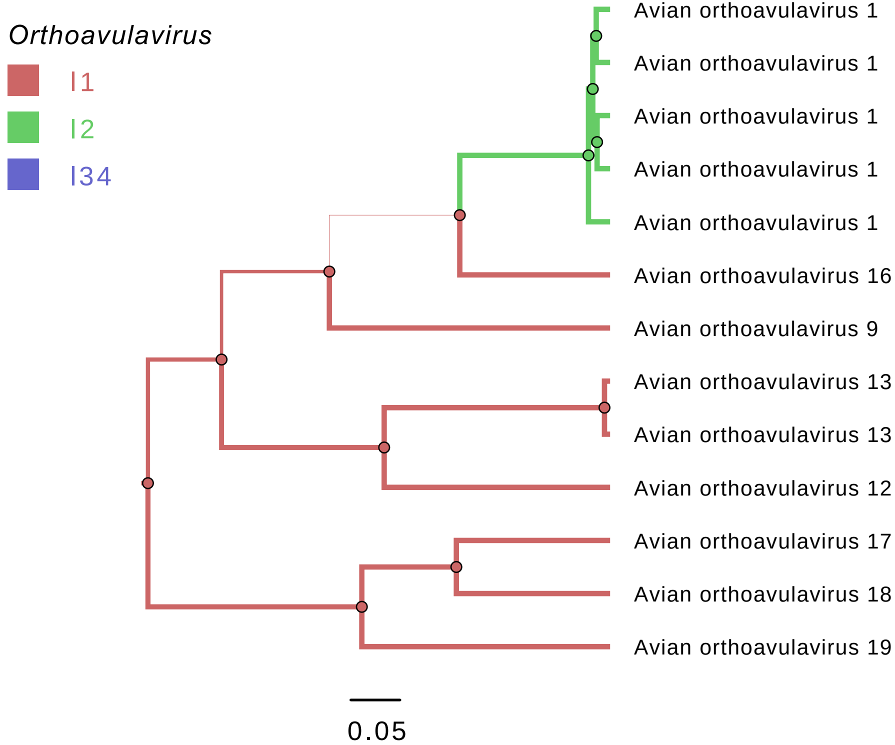

Orthoavulavirus
I1
I2
I34
Avian orthoavulavirus 1
Avian orthoavulavirus 1
Avian orthoavulavirus 1
Avian orthoavulavirus 1
Avian orthoavulavirus 1
Avian orthoavulavirus 16
Avian orthoavulavirus 9
Avian orthoavulavirus 13
Avian orthoavulavirus 13
Avian orthoavulavirus 12
Avian orthoavulavirus 17
Avian orthoavulavirus 18
Avian orthoavulavirus 19
0.05

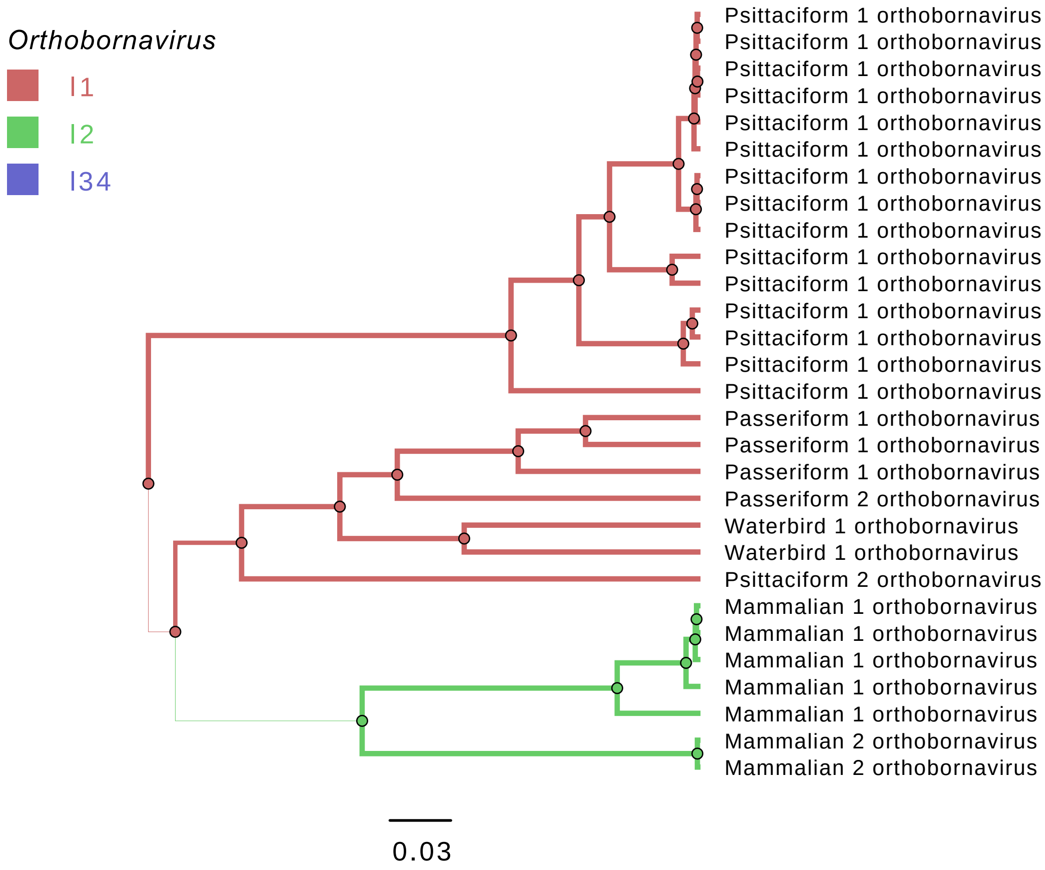
Orthobornavirus
I1
I2
I34
Psittaciform 1 orthobornavirus
Psittaciform 1 orthobornavirus
Psittaciform 1 orthobornavirus
Psittaciform 1 orthobornavirus
Psittaciform 1 orthobornavirus
Psittaciform 1 orthobornavirus
Psittaciform 1 orthobornavirus
Psittaciform 1 orthobornavirus
Psittaciform 1 orthobornavirus
Psittaciform 1 orthobornavirus
Psittaciform 1 orthobornavirus
Psittaciform 1 orthobornavirus
Psittaciform 1 orthobornavirus
Psittaciform 1 orthobornavirus
Psittaciform 1 orthobornavirus
Passeriform 1 orthobornavirus
Passeriform 1 orthobornavirus
Passeriform 1 orthobornavirus
Passeriform 2 orthobornavirus
Waterbird 1 orthobornavirus
Waterbird 1 orthobornavirus
Psittaciform 2 orthobornavirus
Mammalian 1 orthobornavirus
Mammalian 1 orthobornavirus
Mammalian 1 orthobornavirus
Mammalian 1 orthobornavirus
Mammalian 1 orthobornavirus
Mammalian 2 orthobornavirus
Mammalian 2 orthobornavirus
0.03

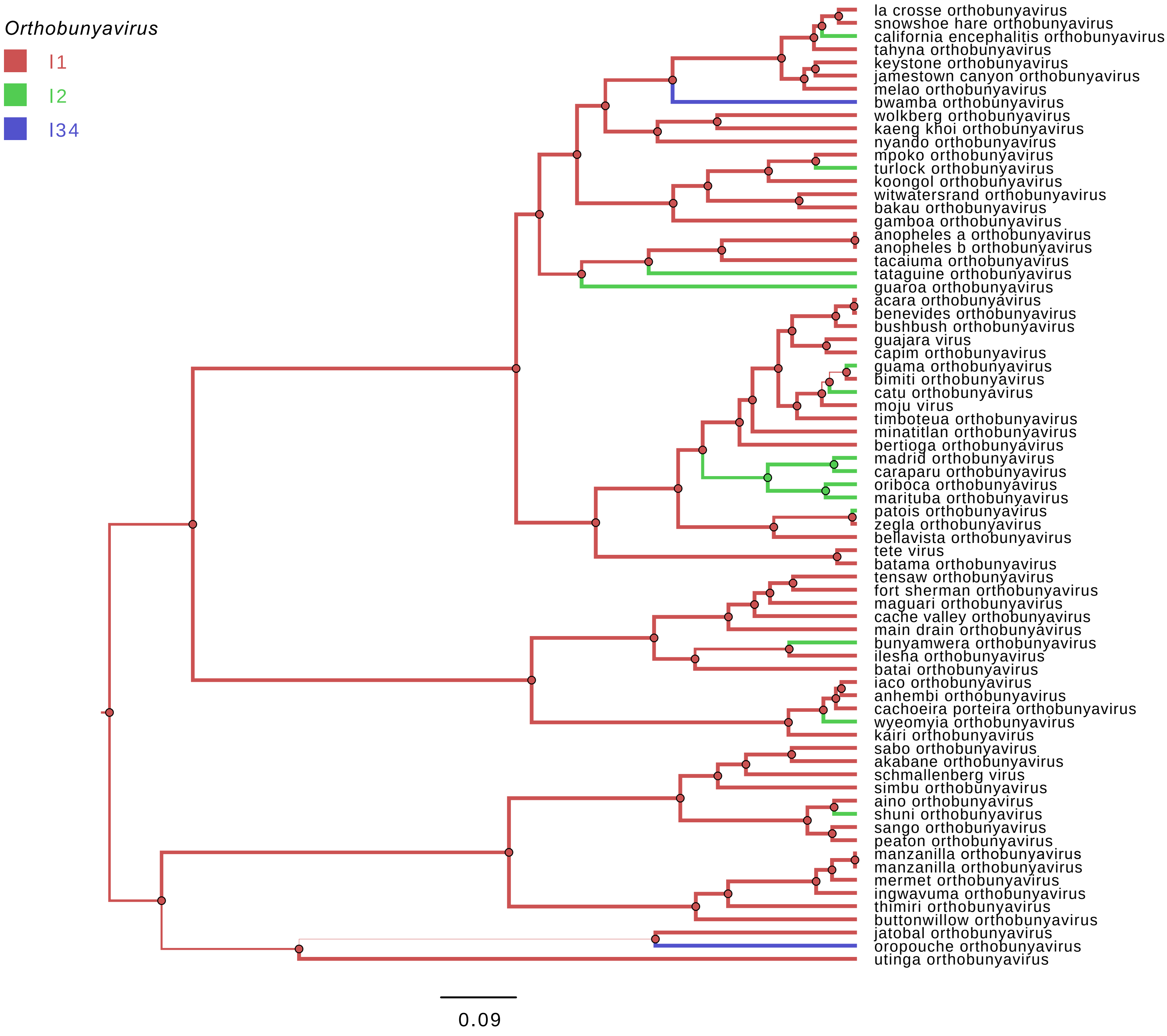

Orthobunyavirus
I1
I2
I34
la crosse orthobunyavirus
snowshoe hare orthobunyavirus
california encephalitis orthobunyavirus
tahyna orthobunyavirus
keystone orthobunyavirus
jamestown canyon orthobunyavirus
melao orthobunyavirus
bwamba orthobunyavirus
wolkberg orthobunyavirus
kaeng khoi orthobunyavirus
nyando orthobunyavirus
mpoko orthobunyavirus
turlock orthobunyavirus
koongol orthobunyavirus
witwatersrand orthobunyavirus
bakau orthobunyavirus
gamboa orthobunyavirus
anopheles a orthobunyavirus
anopheles b orthobunyavirus
tacaiuma orthobunyavirus
tataguine orthobunyavirus
guaroa orthobunyavirus
acara orthobunyavirus
benevides orthobunyavirus
bushbush orthobunyavirus
guajara virus
capim orthobunyavirus
guama orthobunyavirus
bimiti orthobunyavirus
catu orthobunyavirus
moju virus
timboteua orthobunyavirus
minatitlan orthobunyavirus
bertioga orthobunyavirus
madrid orthobunyavirus
caraparu orthobunyavirus
oriboca orthobunyavirus
marituba orthobunyavirus
patois orthobunyavirus
zegla orthobunyavirus
bellavista orthobunyavirus
tete virus
batama orthobunyavirus
tensaw orthobunyavirus
fort sherman orthobunyavirus
maguari orthobunyavirus
cache valley orthobunyavirus
main drain orthobunyavirus
bunyamwera orthobunyavirus
ilesha orthobunyavirus
batai orthobunyavirus
iaco orthobunyavirus
anhembi orthobunyavirus
cachoeira porteira orthobunyavirus
wyeomyia orthobunyavirus
kairi orthobunyavirus
sabo orthobunyavirus
akabane orthobunyavirus
schmallenberg virus
simbu orthobunyavirus
aino orthobunyavirus
shuni orthobunyavirus
sango orthobunyavirus
peaton orthobunyavirus
manzanilla orthobunyavirus
manzanilla orthobunyavirus
mermet orthobunyavirus
ingwavuma orthobunyavirus
thimiri orthobunyavirus
buttonwillow orthobunyavirus
jatobal orthobunyavirus
oropouche orthobunyavirus
utinga orthobunyavirus
0.09

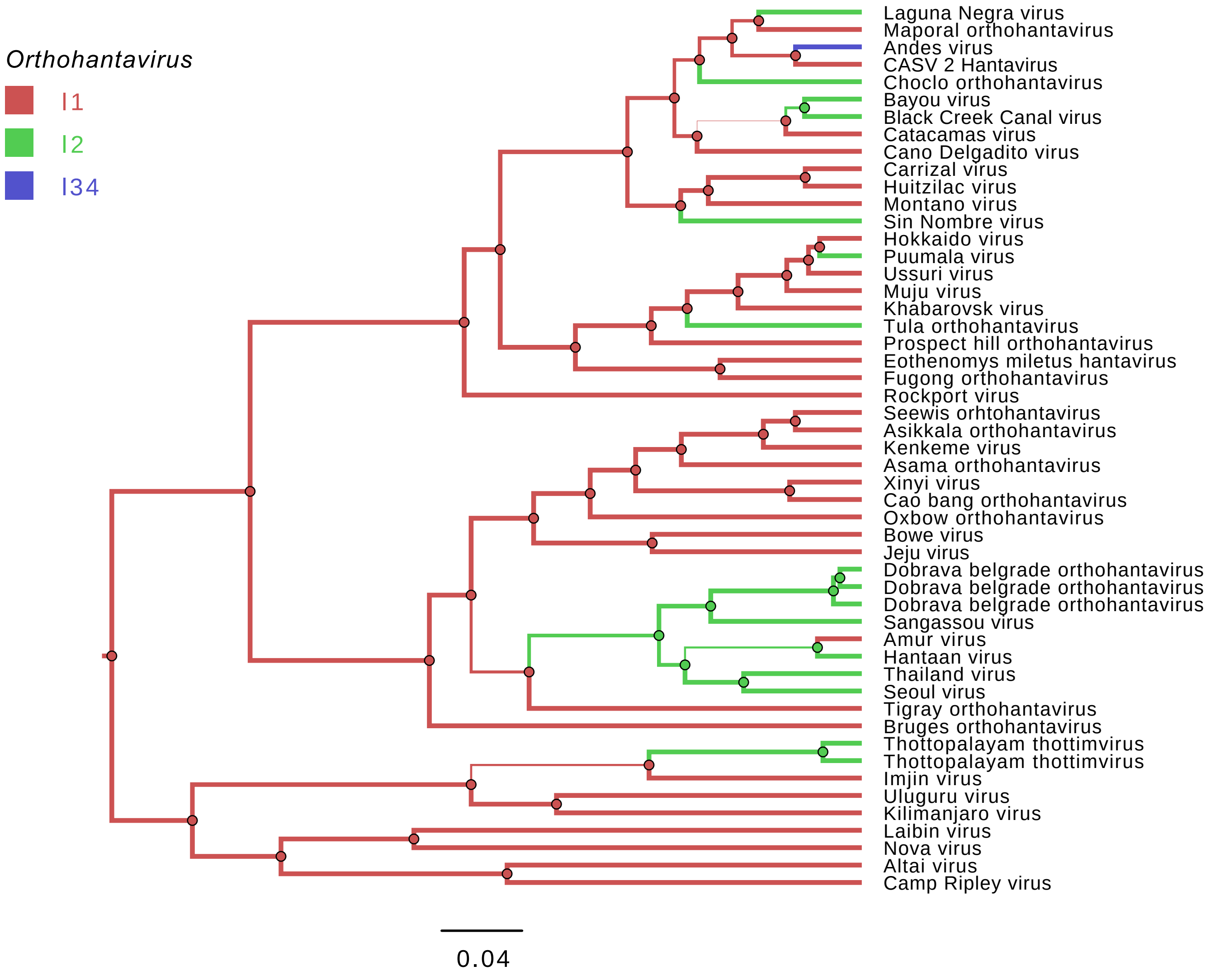

Orthohantavirus
I1
I2
I34
Laguna Negra virus
Maporal orthohantavirus
Andes virus
CASV 2 Hantavirus
Choclo orthohantavirus
Bayou virus
Black Creek Canal virus
Catacamas virus
Cano Delgadito virus
Carrizal virus
Huitzilac virus
Montano virus
Sin Nombre virus
Hokkaido virus
Puumala virus
Ussuri virus
Muju virus
Khabarovsk virus
Tula orthohantavirus
Prospect hill orthohantavirus
Eothenomys miletus hantavirus
Fugong orthohantavirus
Rockport virus
Seewis orhtohantavirus
Asikkala orthohantavirus
Kenkeme virus
Asama orthohantavirus
Xinyi virus
Cao bang orthohantavirus
Oxbow orthohantavirus
Bowe virus
Jeju virus
Dobrava belgrade orthohantavirus
Dobrava belgrade orthohantavirus
Dobrava belgrade orthohantavirus
Sangassou virus
Amur virus
Hantaan virus
Thailand virus
Seoul virus
Tigray orthohantavirus
Bruges orthohantavirus
Thottopalayam thottimvirus
Thottopalayam thottimvirus
Imjin virus
Uluguru virus
Kilimanjaro virus
Laibin virus
Nova virus
Altai virus
Camp Ripley virus
0.04

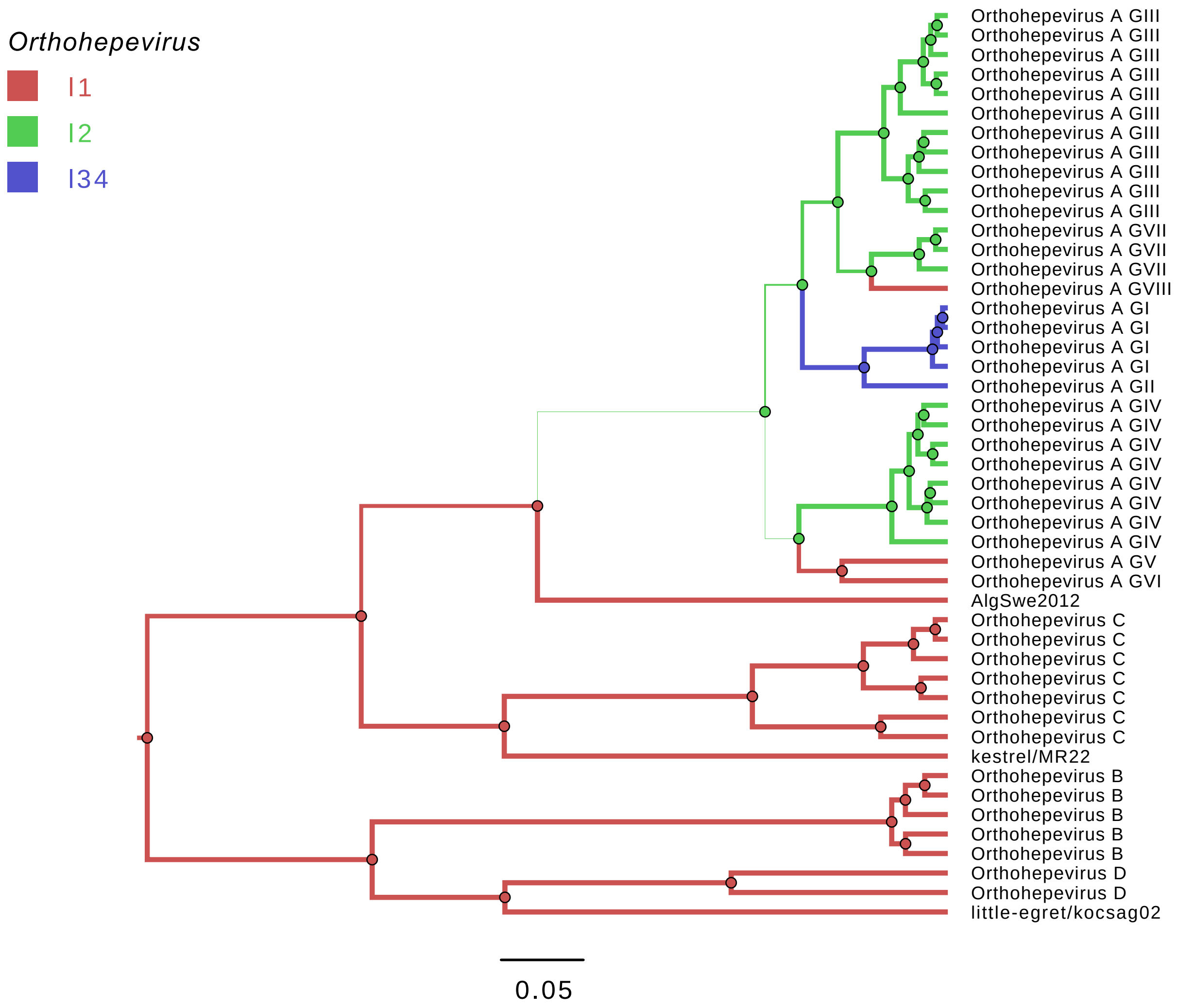
Orthohepevirus
I1
I2
I34
Orthohepevirus A GIII
Orthohepevirus A GIII
Orthohepevirus A GIII
Orthohepevirus A GIII
Orthohepevirus A GIII
Orthohepevirus A GIII
Orthohepevirus A GIII
Orthohepevirus A GIII
Orthohepevirus A GIII
Orthohepevirus A GIII
Orthohepevirus A GIII
Orthohepevirus A GVII
Orthohepevirus A GVII
Orthohepevirus A GVII
Orthohepevirus A GVIII
Orthohepevirus A GI
Orthohepevirus A GI
Orthohepevirus A GI
Orthohepevirus A GI
Orthohepevirus A GII
Orthohepevirus A GIV
Orthohepevirus A GIV
Orthohepevirus A GIV
Orthohepevirus A GIV
Orthohepevirus A GIV
Orthohepevirus A GIV
Orthohepevirus A GIV
Orthohepevirus A GIV
Orthohepevirus A GV
Orthohepevirus A GVI
AlgSwe2012
Orthohepevirus C
Orthohepevirus C
Orthohepevirus C
Orthohepevirus C
Orthohepevirus C
Orthohepevirus C
Orthohepevirus C
kestrel/MR22
Orthohepevirus B
Orthohepevirus B
Orthohepevirus B
Orthohepevirus B
Orthohepevirus B
Orthohepevirus D
Orthohepevirus D
little-egret/kocsag02
0.05

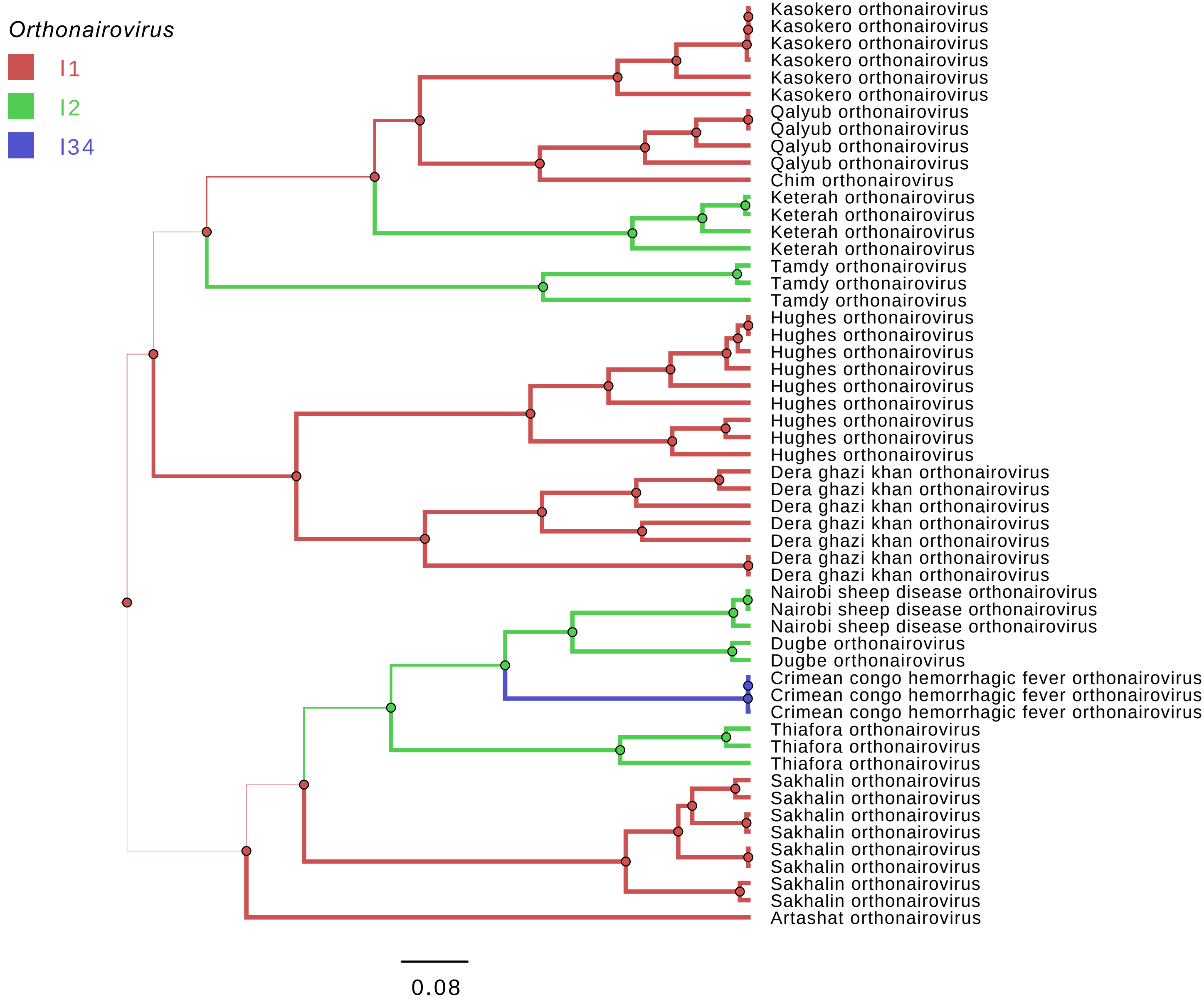
Orthonairovirus
I1
I2
I34
Kasokero orthonairovirus
Kasokero orthonairovirus
Kasokero orthonairovirus
Kasokero orthonairovirus
Kasokero orthonairovirus
Kasokero orthonairovirus
Qalyub orthonairovirus
Qalyub orthonairovirus
Qalyub orthonairovirus
Qalyub orthonairovirus
Chim orthonairovirus
Keterah orthonairovirus
Keterah orthonairovirus
Keterah orthonairovirus
Keterah orthonairovirus
Tamdy orthonairovirus
Tamdy orthonairovirus
Tamdy orthonairovirus
Hughes orthonairovirus
Hughes orthonairovirus
Hughes orthonairovirus
Hughes orthonairovirus
Hughes orthonairovirus
Hughes orthonairovirus
Hughes orthonairovirus
Hughes orthonairovirus
Hughes orthonairovirus
Dera ghazi khan orthonairovirus
Dera ghazi khan orthonairovirus
Dera ghazi khan orthonairovirus
Dera ghazi khan orthonairovirus
Dera ghazi khan orthonairovirus
Dera ghazi khan orthonairovirus
Dera ghazi khan orthonairovirus
Nairobi sheep disease orthonairovirus
Nairobi sheep disease orthonairovirus
Nairobi sheep disease orthonairovirus
Dugbe orthonairovirus
Dugbe orthonairovirus
Crimean congo hemorrhagic fever orthonairovirus
Crimean congo hemorrhagic fever orthonairovirus
Crimean congo hemorrhagic fever orthonairovirus
Thiafora orthonairovirus
Thiafora orthonairovirus
Thiafora orthonairovirus
Sakhalin orthonairovirus
Sakhalin orthonairovirus
Sakhalin orthonairovirus
Sakhalin orthonairovirus
Sakhalin orthonairovirus
Sakhalin orthonairovirus
Sakhalin orthonairovirus
Sakhalin orthonairovirus
Artashat orthonairovirus
0.08

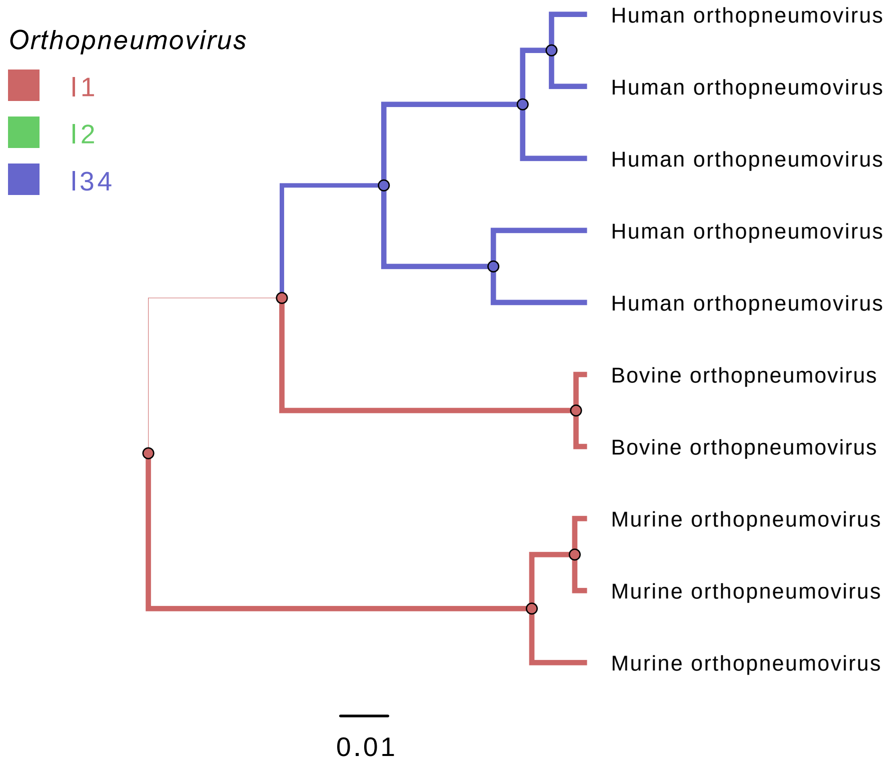
Orthopneumovirus
I1
I2
I34
Human orthopneumovirus
Human orthopneumovirus
Human orthopneumovirus
Human orthopneumovirus
Human orthopneumovirus
Bovine orthopneumovirus
Bovine orthopneumovirus
Murine orthopneumovirus
Murine orthopneumovirus
Murine orthopneumovirus
0.01

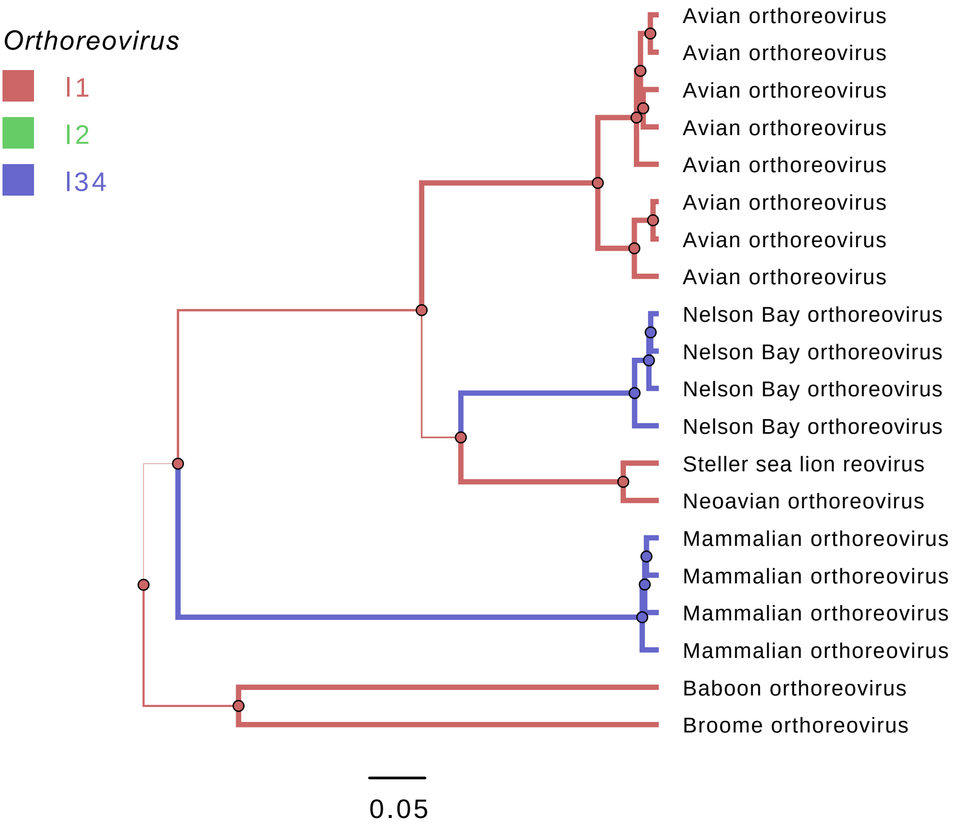

Orthoreovirus
I1
I2
I34
Avian orthoreovirus
Avian orthoreovirus
Avian orthoreovirus
Avian orthoreovirus
Avian orthoreovirus
Avian orthoreovirus
Avian orthoreovirus
Avian orthoreovirus
Nelson Bay orthoreovirus
Nelson Bay orthoreovirus
Nelson Bay orthoreovirus
Nelson Bay orthoreovirus
Steller sea lion reovirus
Neoavian orthoreovirus
Mammalian orthoreovirus
Mammalian orthoreovirus
Mammalian orthoreovirus
Mammalian orthoreovirus
Baboon orthoreovirus
Broome orthoreovirus
0.05

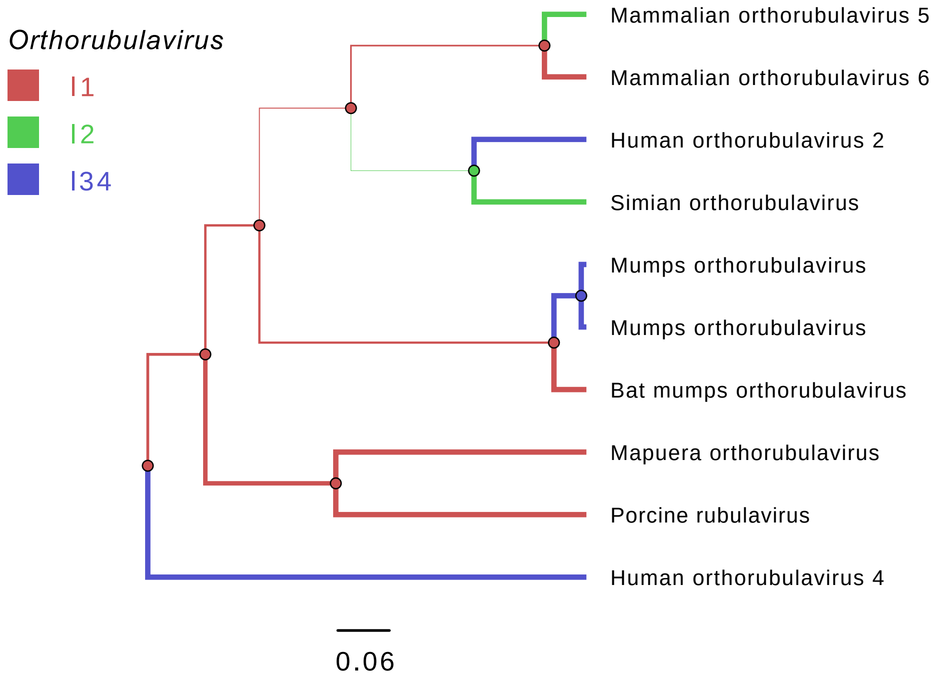
Orthorubulavirus
I1
I2
I34
Mammalian orthorubulavirus 5
Mammalian orthorubulavirus 6
Human orthorubulavirus 2
Simian orthorubulavirus
Mumps orthorubulavirus
Mumps orthorubulavirus
Bat mumps orthorubulavirus
Mapuera orthorubulavirus
Porcine rubulavirus
Human orthorubulavirus 4
0.06

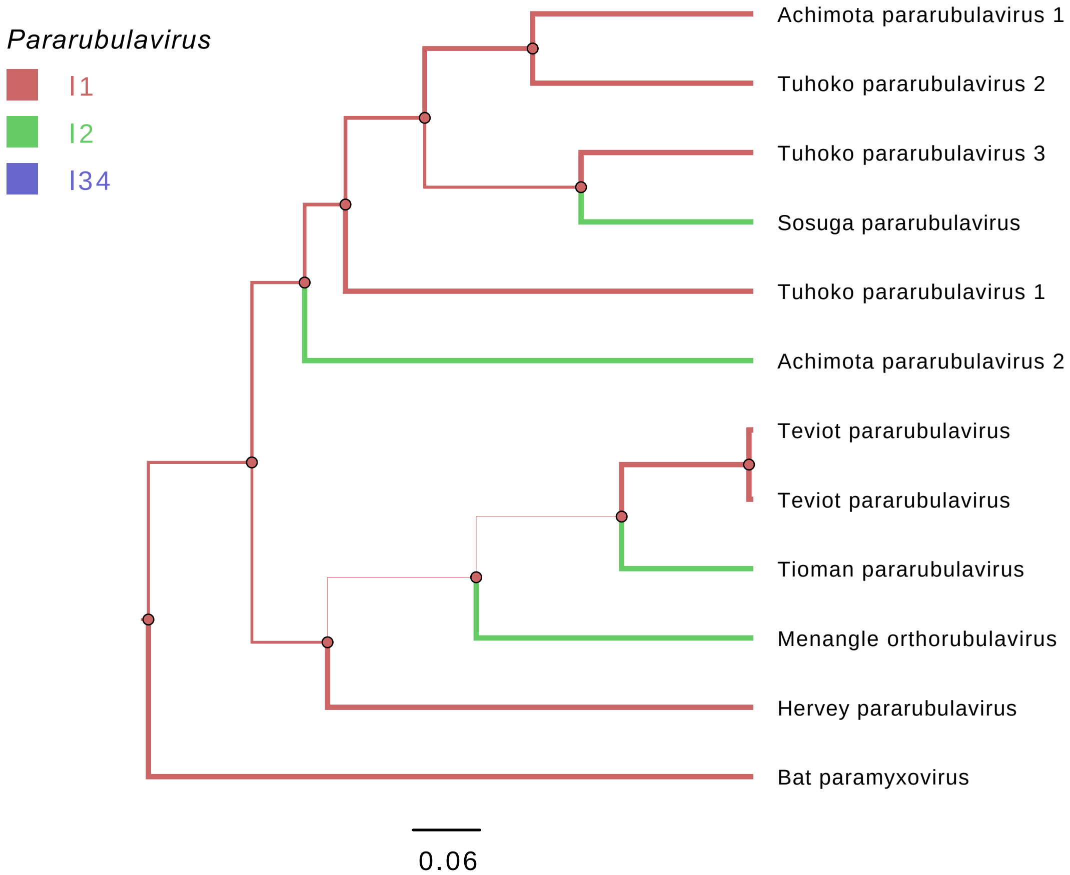

Pararubulavirus
I1
I2
I34
Achimota pararubulavirus 1
Tuhoko pararubulavirus 2
Tuhoko pararubulavirus 3
Sosuga pararubulavirus
Tuhoko pararubulavirus 1
Achimota pararubulavirus 2
Teviot pararubulavirus
Teviot pararubulavirus
Tioman pararubulavirus
Menangle orthorubulavirus
Hervey pararubulavirus
Bat paramyxovirus
0.06

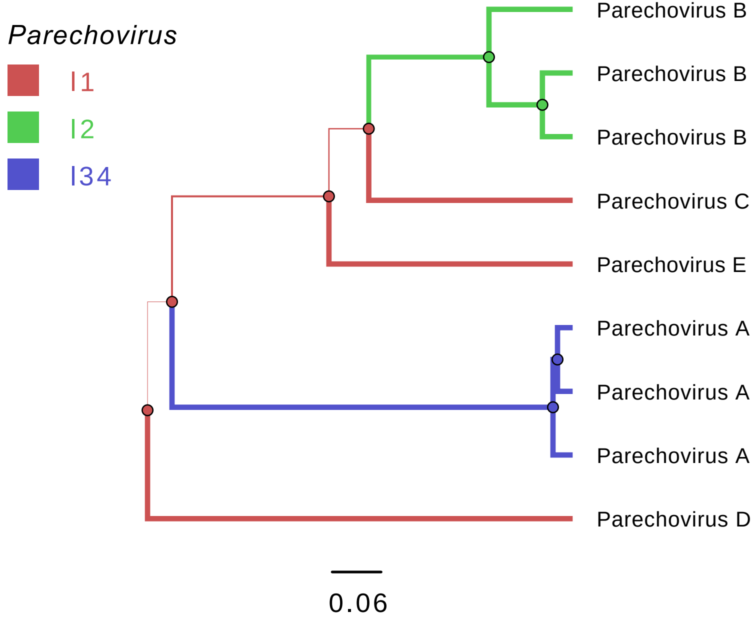

Parechovirus
I1
I2
I34
Parechovirus B
Parechovirus B
Parechovirus B
Parechovirus C
Parechovirus E
Parechovirus A
Parechovirus A
Parechovirus A
Parechovirus D
0.06

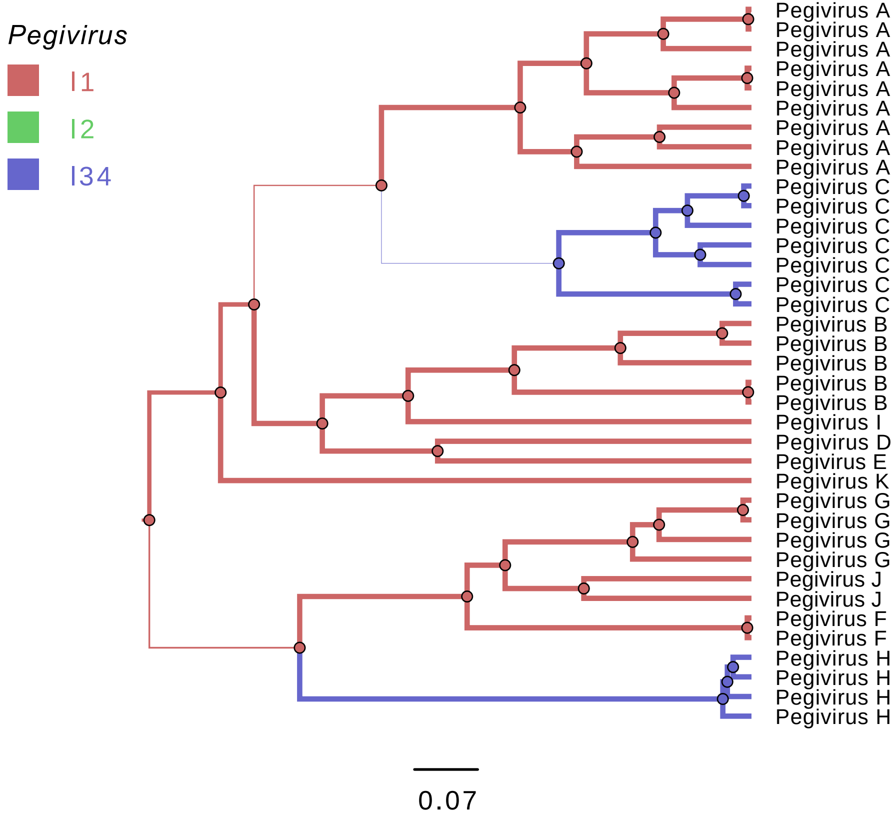

Pegivirus
I1
I2
I34
Pegivirus A
Pegivirus A
Pegivirus A
Pegivirus A
Pegivirus A
Pegivirus A
Pegivirus A
Pegivirus A
Pegivirus A
Pegivirus C
Pegivirus C
Pegivirus C
Pegivirus C
Pegivirus C
Pegivirus C
Pegivirus C
Pegivirus B
Pegivirus B
Pegivirus B
Pegivirus B
Pegivirus B
Pegivirus I
Pegivirus D
Pegivirus E
Pegivirus K
Pegivirus G
Pegivirus G
Pegivirus G
Pegivirus G
Pegivirus J
Pegivirus J
Pegivirus F
Pegivirus F
Pegivirus H
Pegivirus H
Pegivirus H
Pegivirus H
0.07

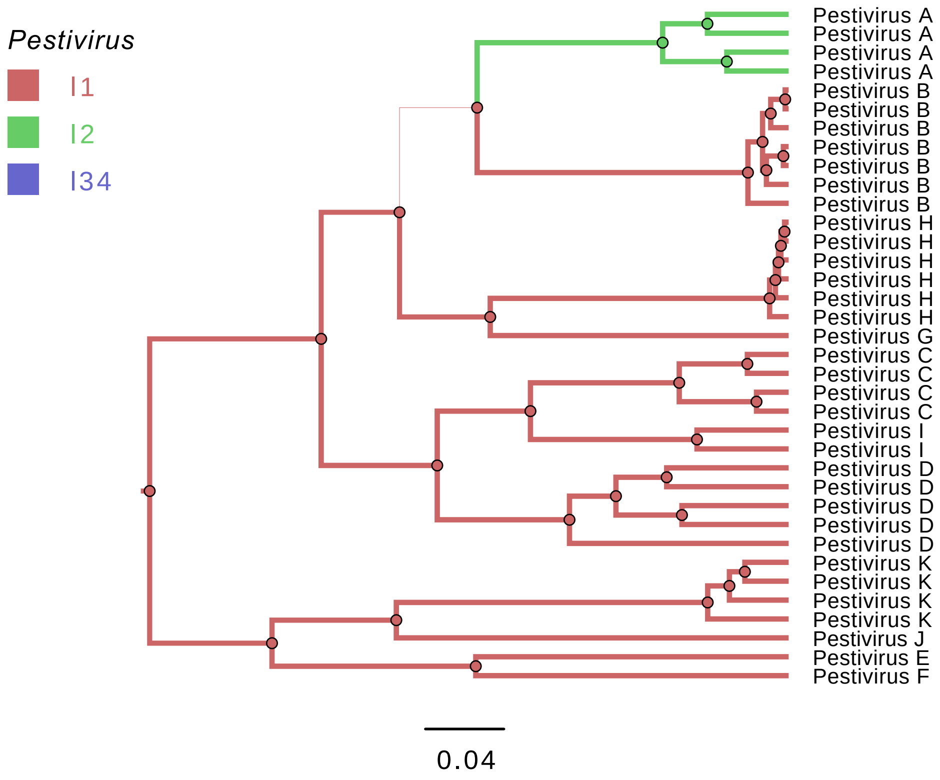

Pestivirus
I1
I2
I34
Pestivirus A
Pestivirus A
Pestivirus A
Pestivirus A
Pestivirus B
Pestivirus B
Pestivirus B
Pestivirus B
Pestivirus B
Pestivirus B
Pestivirus B
Pestivirus H
Pestivirus H
Pestivirus H
Pestivirus H
Pestivirus H
Pestivirus H
Pestivirus G
Pestivirus C
Pestivirus C
Pestivirus C
Pestivirus C
Pestivirus I
Pestivirus I
Pestivirus D
Pestivirus D
Pestivirus D
Pestivirus D
Pestivirus D
Pestivirus K
Pestivirus K
Pestivirus K
Pestivirus K
Pestivirus J
Pestivirus E
Pestivirus F
0.04

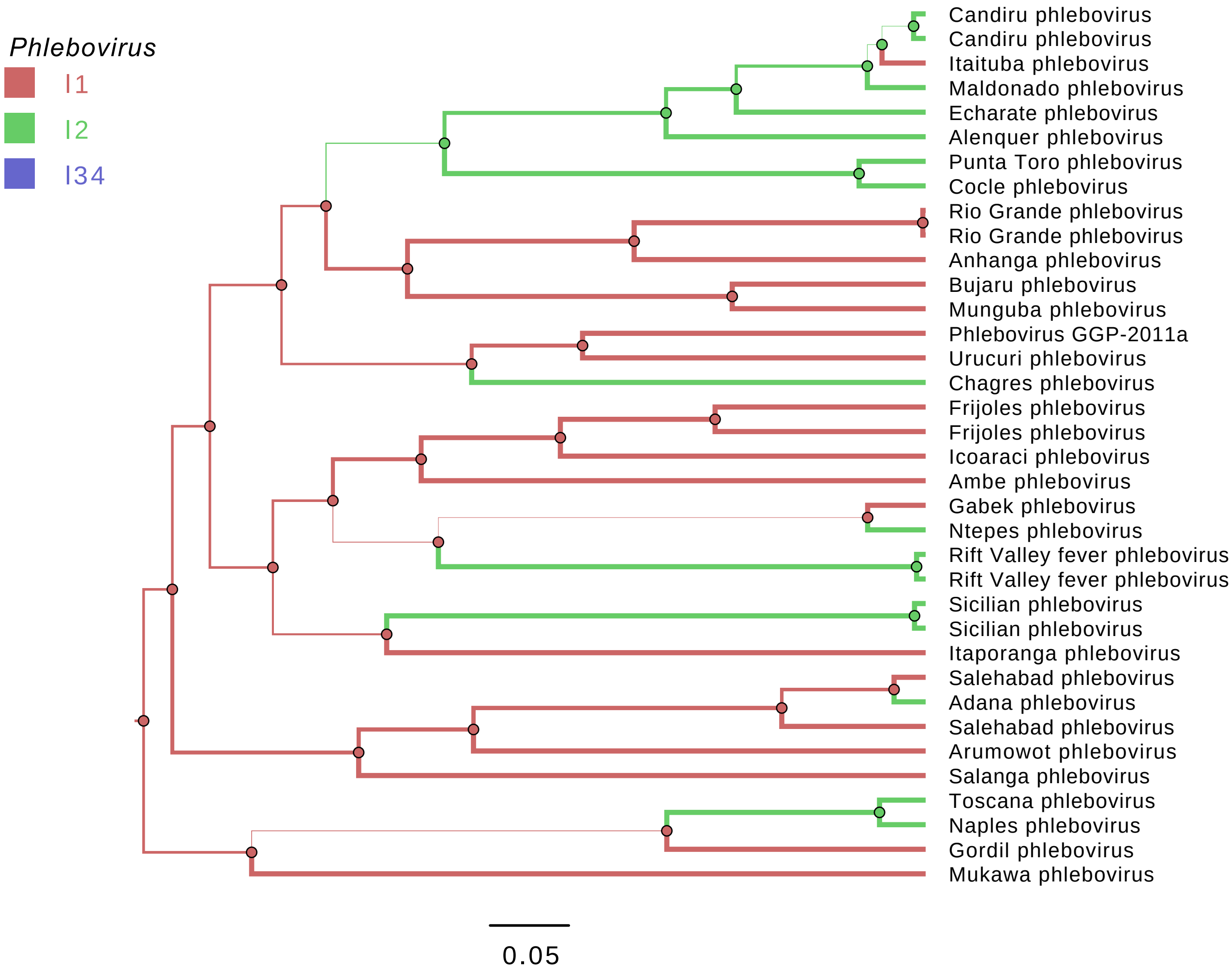
Phlebovirus
I1
I2
I34
Candiru phlebovirus
Candiru phlebovirus
Itaituba phlebovirus
Maldonado phlebovirus
Echarate phlebovirus
Alenquer phlebovirus
Punta Toro phlebovirus
Cocle phlebovirus
Rio Grande phlebovirus
Rio Grande phlebovirus
Anhanga phlebovirus
Bujaru phlebovirus
Munguba phlebovirus
Phlebovirus GGP-2011a
Urucuri phlebovirus
Chagres phlebovirus
Frijoles phlebovirus
Frijoles phlebovirus
Icoaraci phlebovirus
Ambe phlebovirus
Gabek phlebovirus
Ntepes phlebovirus
Rift Valley fever phlebovirus
Rift Valley fever phlebovirus
Sicilian phlebovirus
Sicilian phlebovirus
Itaporanga phlebovirus
Salehabad phlebovirus
Adana phlebovirus
Salehabad phlebovirus
Arumowot phlebovirus
Salanga phlebovirus
Toscana phlebovirus
Naples phlebovirus
Gordil phlebovirus
Mukawa phlebovirus
0.05

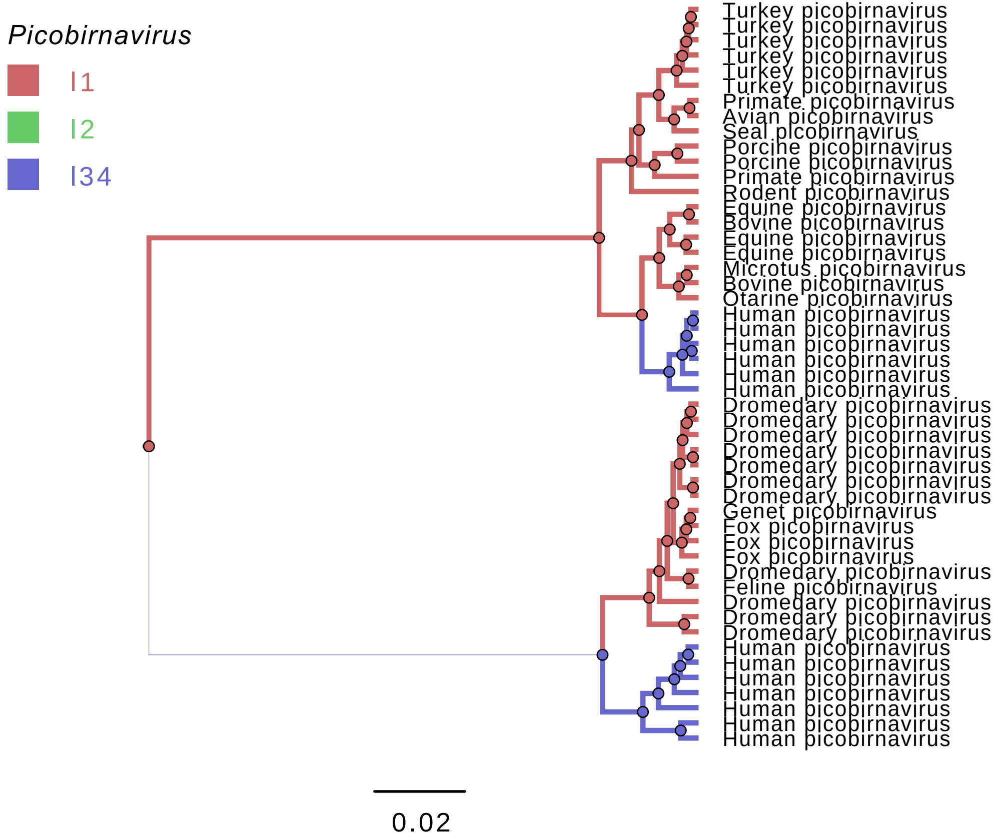

Picobirnavirus
I1
I2
I34
Turkey picobirnavirus
Turkey picobirnavirus
Turkey picobirnavirus
Turkey picobirnavirus
Turkey picobirnavirus
Turkey picobirnavirus
Primate picobirnavirus
Avian picobirnavirus
Seal picobirnavirus
Porcine picobirnavirus
Porcine picobirnavirus
Primate picobirnavirus
Rodent picobirnavirus
Equine picobirnavirus
Bovine picobirnavirus
Equine picobirnavirus
Equine picobirnavirus
Microtus picobirnavirus
Bovine picobirnavirus
Otarine picobirnavirus
Human picobirnavirus
Human picobirnavirus
Human picobirnavirus
Human picobirnavirus
Human picobirnavirus
Human picobirnavirus
Dromedary picobirnavirus
Dromedary picobirnavirus
Dromedary picobirnavirus
Dromedary picobirnavirus
Dromedary picobirnavirus
Dromedary picobirnavirus
Dromedary picobirnavirus
Genet picobirnavirus
Fox picobirnavirus
Fox picobirnavirus
Fox picobirnavirus
Dromedary picobirnavirus
Feline picobirnavirus
Dromedary picobirnavirus
Dromedary picobirnavirus
Dromedary picobirnavirus
Human picobirnavirus
Human picobirnavirus
Human picobirnavirus
Human picobirnavirus
Human picobirnavirus
Human picobirnavirus
Human picobirnavirus
0.02

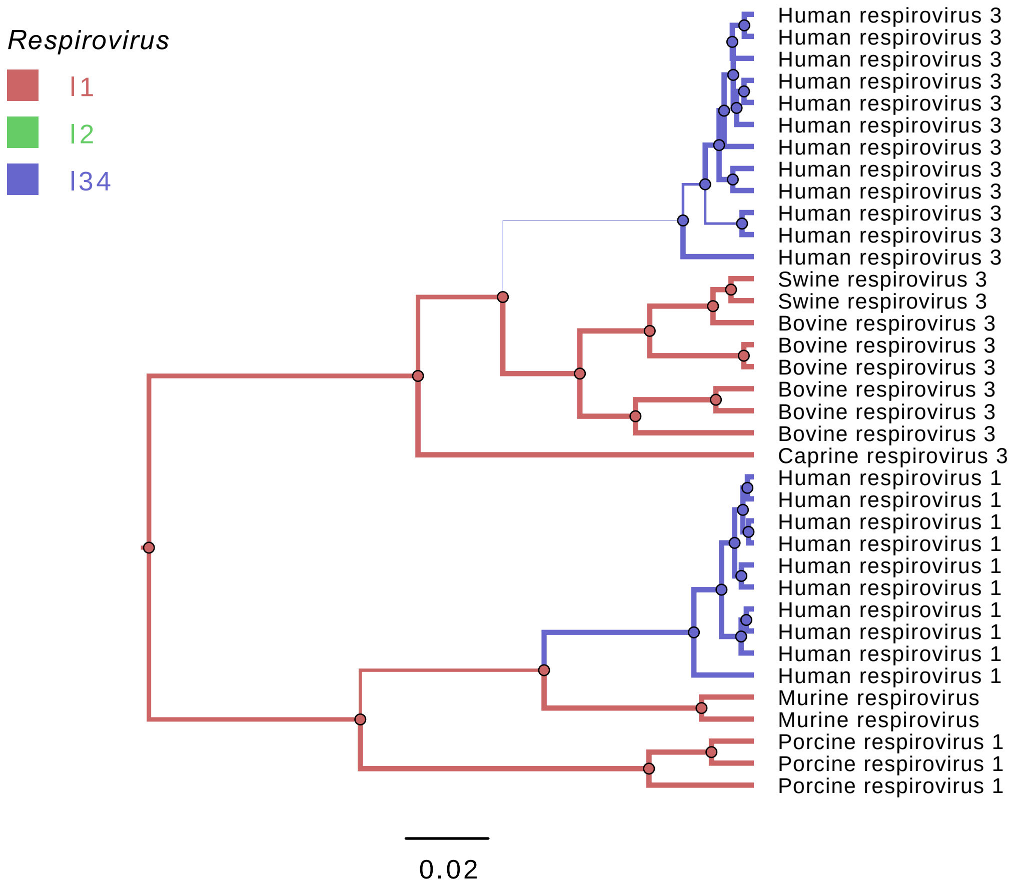

Respirovirus
l1
l2
l34
Human respirovirus 3
Human respirovirus 3
Human respirovirus 3
Human respirovirus 3
Human respirovirus 3
Human respirovirus 3
Human respirovirus 3
Human respirovirus 3
Human respirovirus 3
Human respirovirus 3
Human respirovirus 3
Human respirovirus 3
Swine respirovirus 3
Swine respirovirus 3
Bovine respirovirus 3
Bovine respirovirus 3
Bovine respirovirus 3
Bovine respirovirus 3
Bovine respirovirus 3
Bovine respirovirus 3
Caprine respirovirus 3
Human respirovirus 1
Human respirovirus 1
Human respirovirus 1
Human respirovirus 1
Human respirovirus 1
Human respirovirus 1
Human respirovirus 1
Human respirovirus 1
Human respirovirus 1
Human respirovirus 1
Murine respirovirus
Murine respirovirus
Porcine respirovirus 1
Porcine respirovirus 1
Porcine respirovirus 1
0.02

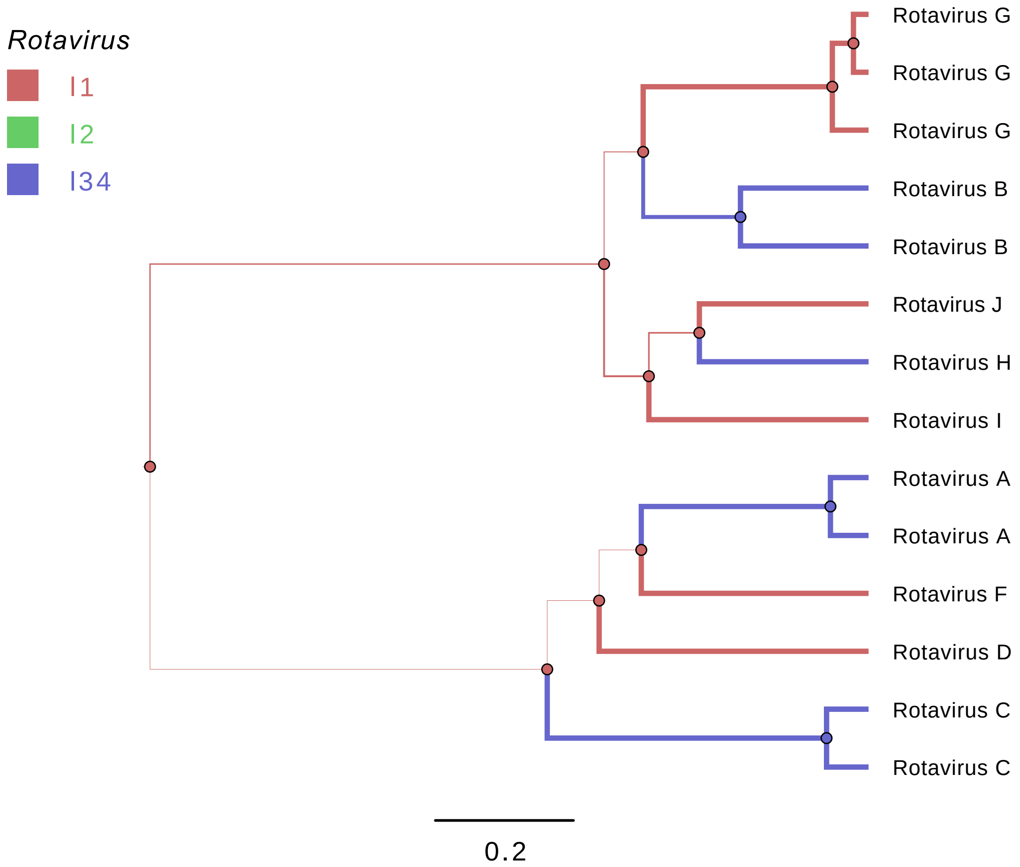

Rotavirus
I1
I2
I34
Rotavirus G
Rotavirus G
Rotavirus G
Rotavirus B
Rotavirus B
Rotavirus J
Rotavirus H
Rotavirus I
Rotavirus A
Rotavirus A
Rotavirus F
Rotavirus D
Rotavirus C
Rotavirus C
0.2

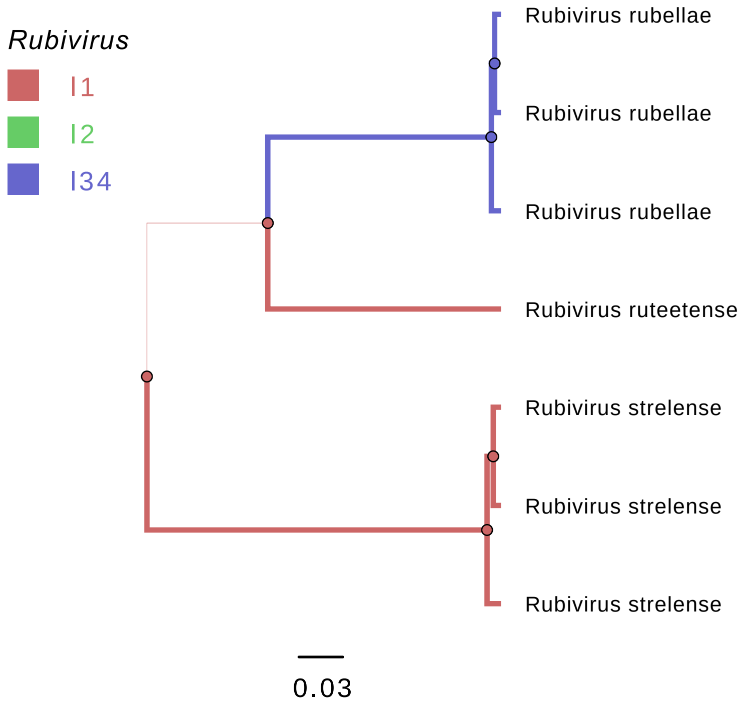
Rubivirus
I1
I2
I34
Rubivirus rubellae
Rubivirus rubellae
Rubivirus rubellae
Rubivirus ruteetense
Rubivirus strelense
Rubivirus strelense
Rubivirus strelense
0.03

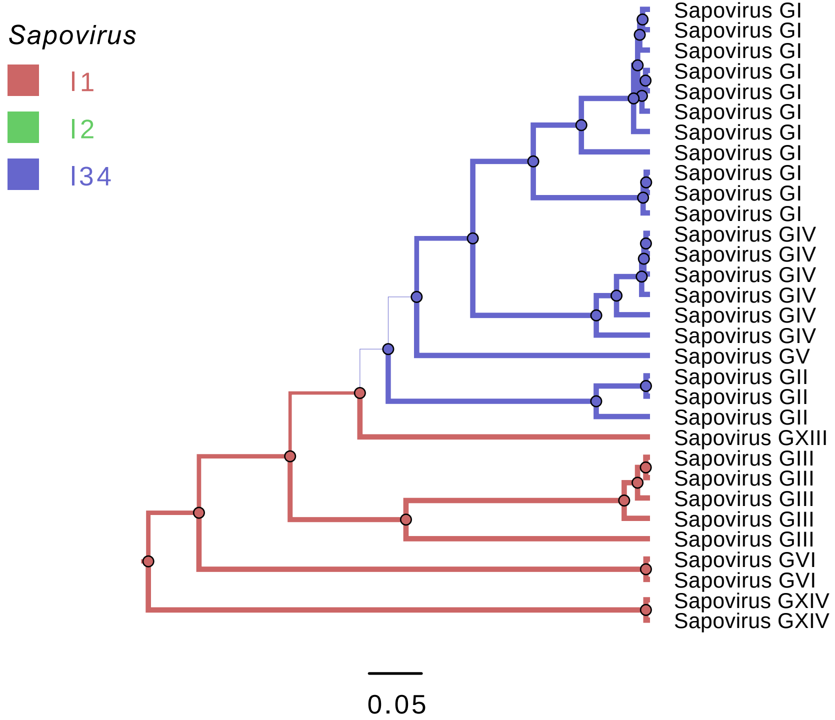
Sapovirus
I1
I2
I34
Sapovirus GI
Sapovirus GI
Sapovirus GI
Sapovirus GI
Sapovirus GI
Sapovirus GI
Sapovirus GI
Sapovirus GI
Sapovirus GI
Sapovirus GI
Sapovirus GI
Sapovirus GIV
Sapovirus GIV
Sapovirus GIV
Sapovirus GIV
Sapovirus GIV
Sapovirus GIV
Sapovirus GV
Sapovirus GII
Sapovirus GII
Sapovirus GII
Sapovirus GXIII
Sapovirus GIII
Sapovirus GIII
Sapovirus GIII
Sapovirus GIII
Sapovirus GIII
Sapovirus GVI
Sapovirus GVI
Sapovirus GXIV
Sapovirus GXIV
0.05

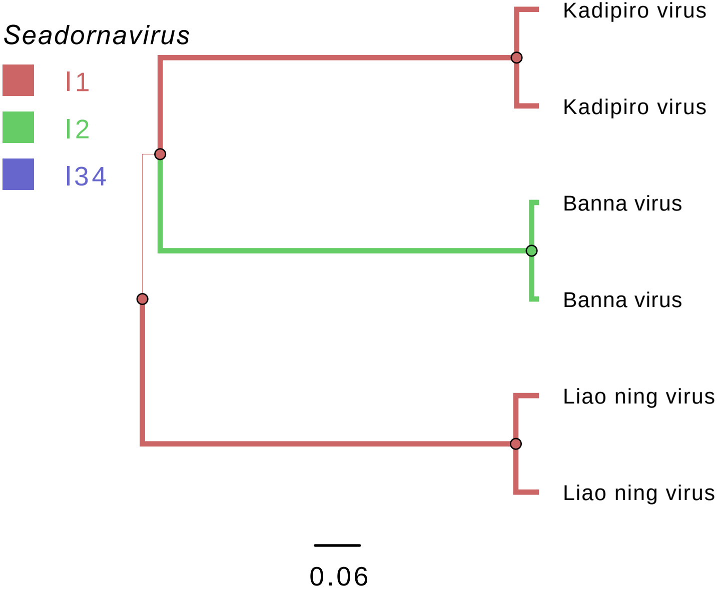

Seadornavirus
I1
I2
I34
Kadipiro virus
Kadipiro virus
Banna virus
Banna virus
Liao ning virus
Liao ning virus
0.06

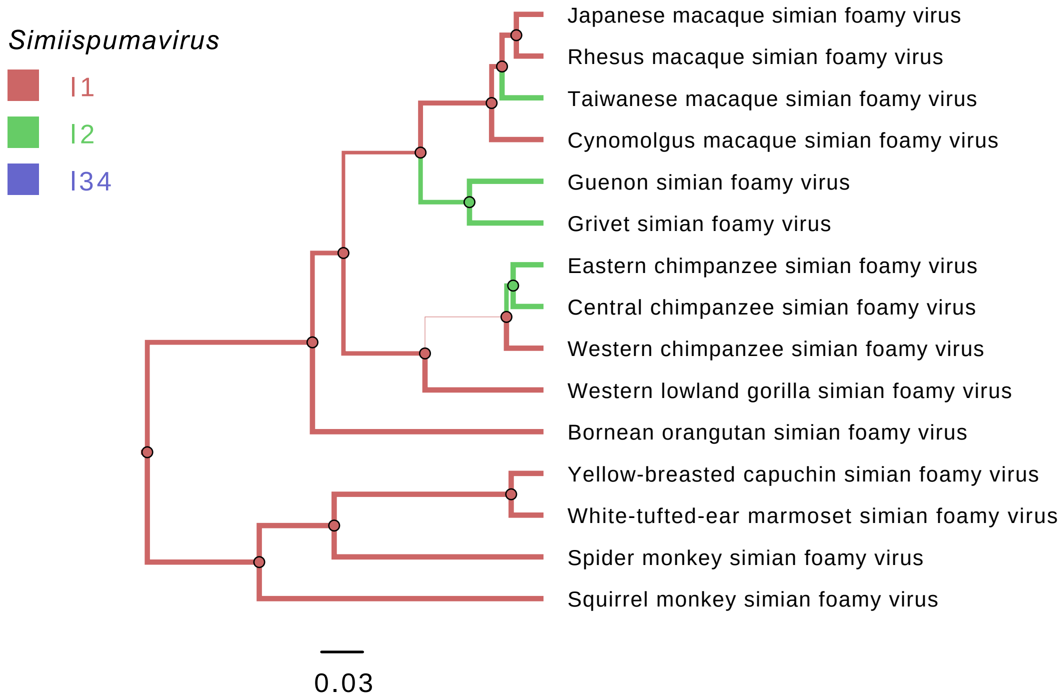

Simiispumavirus
I1
I2
I34
Japanese macaque simian foamy virus
Rhesus macaque simian foamy virus
Taiwanese macaque simian foamy virus
Cynomolgus macaque simian foamy virus
Guenon simian foamy virus
Grivet simian foamy virus
Eastern chimpanzee simian foamy virus
Central chimpanzee simian foamy virus
Western chimpanzee simian foamy virus
Western lowland gorilla simian foamy virus
Bornean orangutan simian foamy virus
Yellow-breasted capuchin simian foamy virus
White-tufted-ear marmoset simian foamy virus
Spider monkey simian foamy virus
Squirrel monkey simian foamy virus
0.03

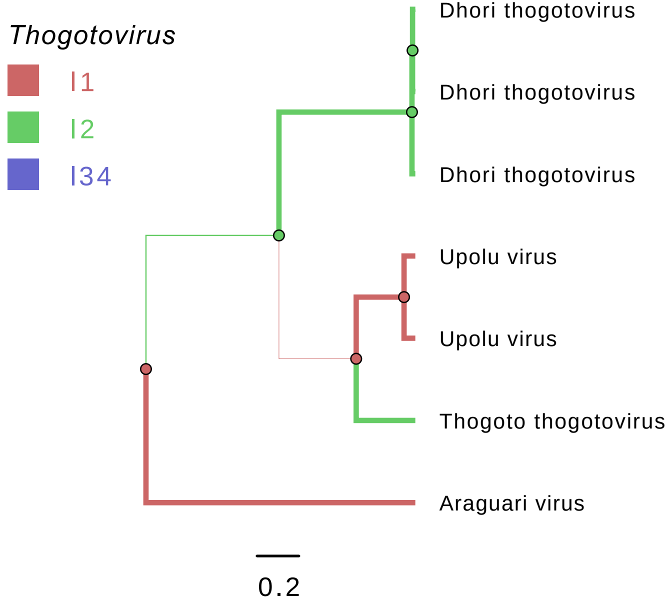
Thogotovirus
1
2
34
Dhori thogotovirus
Dhori thogotovirus
Dhori thogotovirus
Upolu virus
Upolu virus
Thogoto thogotovirus
Araguari virus
0.2

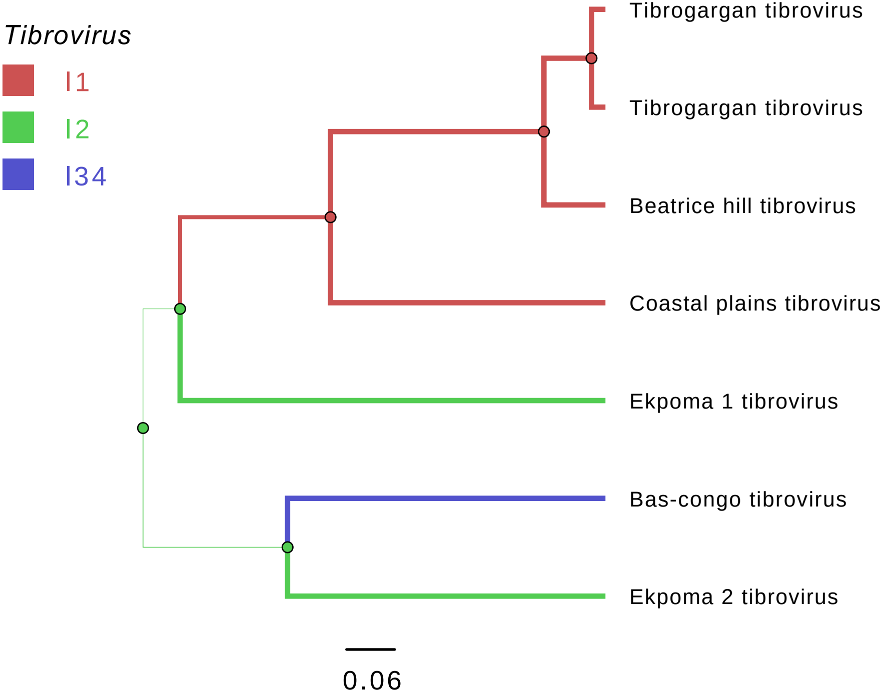
Tibrovirus
I1
I2
I34
Tibrogargan tibrovirus
Tibrogargan tibrovirus
Beatrice hill tibrovirus
Coastal plains tibrovirus
Ekpoma 1 tibrovirus
Bas-congo tibrovirus
Ekpoma 2 tibrovirus
0.06

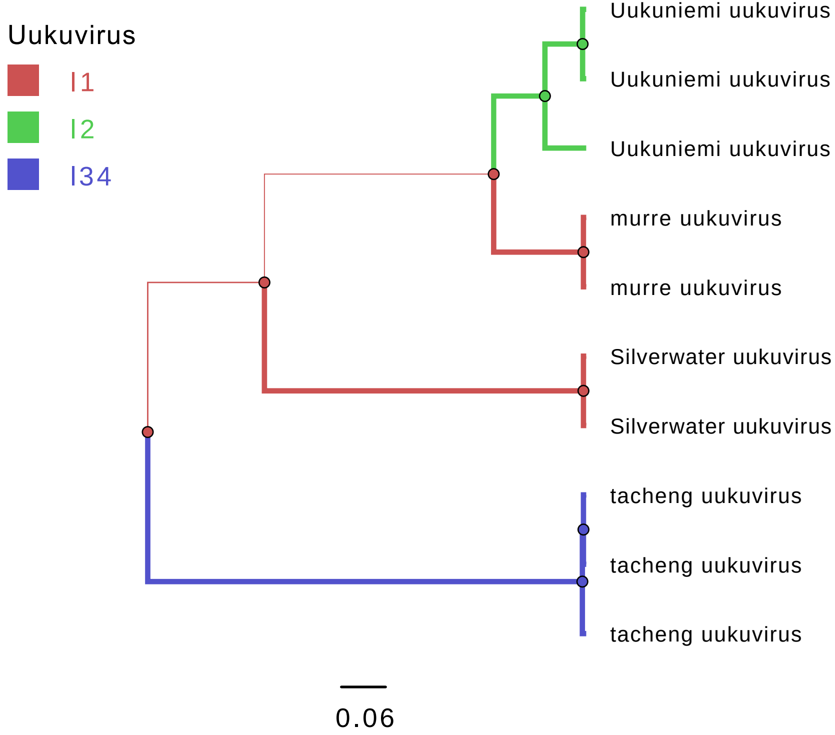
Uukuvirus
I1
I2
I34
Uukuniemi uukuvirus
Uukuniemi uukuvirus
Uukuniemi uukuvirus
murre uukuvirus
murre uukuvirus
Silverwater uukuvirus
Silverwater uukuvirus
tacheng uukuvirus
tacheng uukuvirus
tacheng uukuvirus
0.06

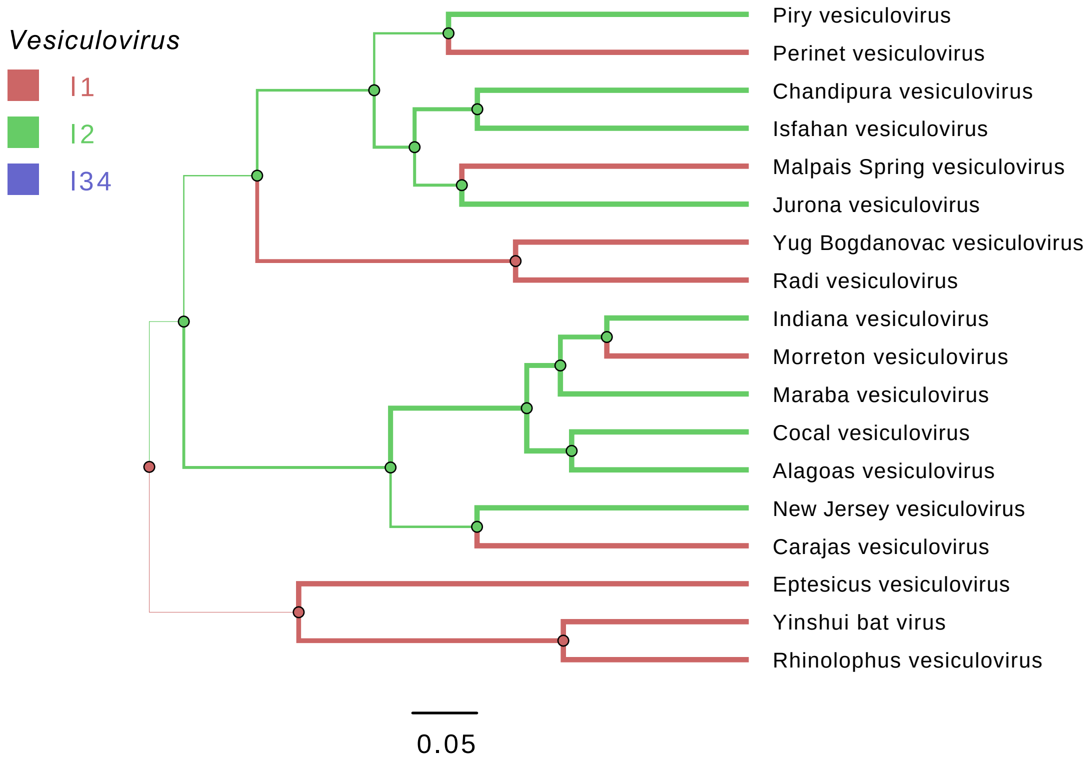
Vesiculovirus
I1
I2
I34
Piry vesiculovirus
Perinet vesiculovirus
Chandipura vesiculovirus
Isfahan vesiculovirus
Malpais Spring vesiculovirus
Jurona vesiculovirus
Yug Bogdanovac vesiculovirus
Radi vesiculovirus
Indiana vesiculovirus
Morreton vesiculovirus
Maraba vesiculovirus
Cocal vesiculovirus
Alagoas vesiculovirus
New Jersey vesiculovirus
Carajas vesiculovirus
Eptesicus vesiculovirus
Yinshui bat virus
Rhinolophus vesiculovirus
0.05

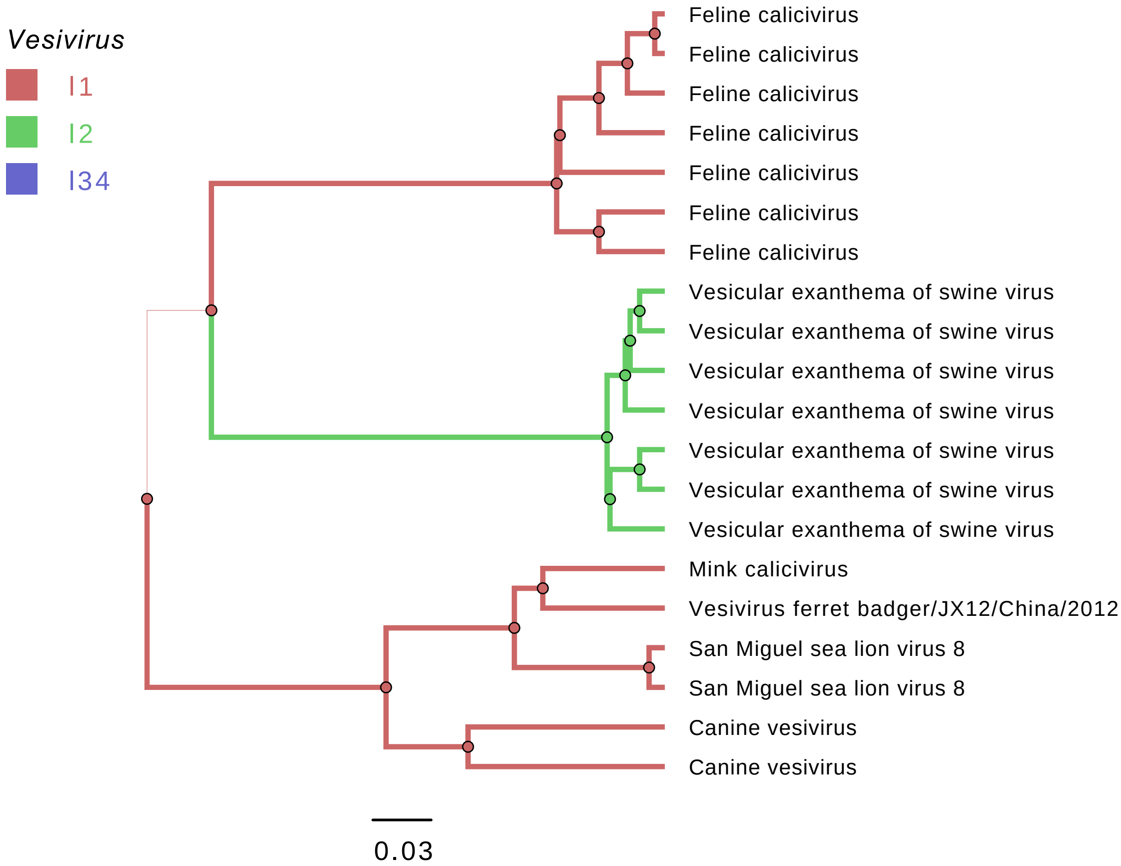

Vesivirus
I1
I2
I34
Feline calicivirus
Feline calicivirus
Feline calicivirus
Feline calicivirus
Feline calicivirus
Feline calicivirus
Feline calicivirus
Vesicular exanthema of swine virus
Vesicular exanthema of swine virus
Vesicular exanthema of swine virus
Vesicular exanthema of swine virus
Vesicular exanthema of swine virus
Vesicular exanthema of swine virus
Vesicular exanthema of swine virus
Mink calicivirus
Vesivirus ferret badger/JX12/China/2012
San Miguel sea lion virus 8
San Miguel sea lion virus 8
Canine vesivirus
Canine vesivirus
0.03
