# Supplementary material for: Temporal Dynamics, Discovery, and Emergence of Human-Transmissible RNA Viruses
Source: Mol Biol Evol. 2024 Jan 18;41(1):msad272. doi: 10.1093/molbev/msad272 (PMC10797954; doi:10.1093/molbev/msad272)

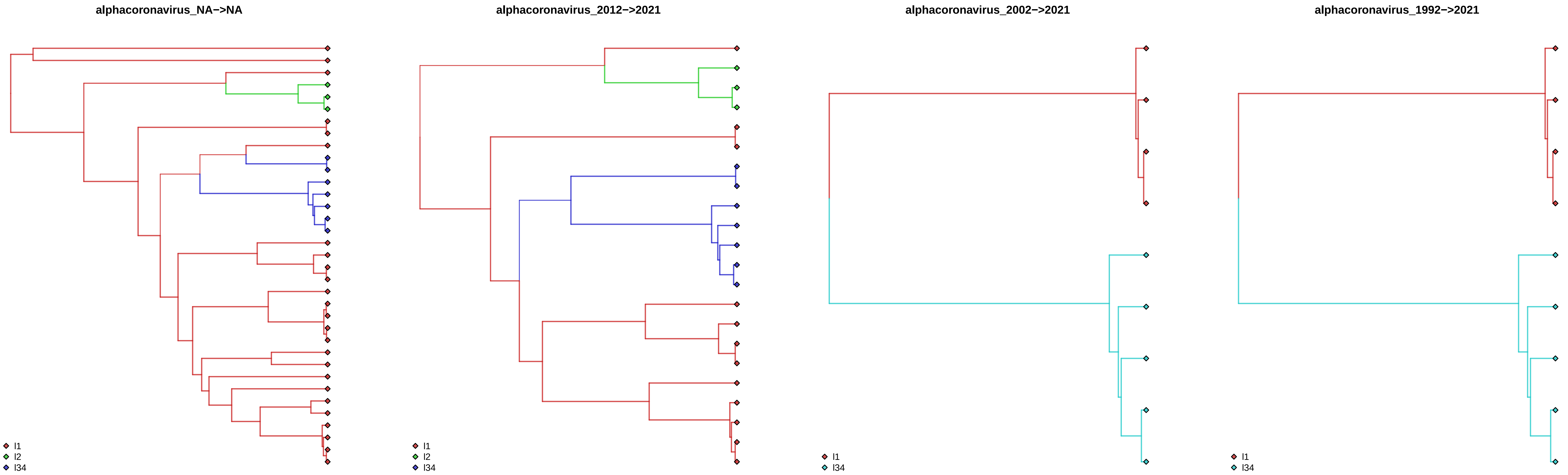
alphacoronavirus_NA−>NA
l1
l2
l34
alphacoronavirus_2012−>2021
l1
l2
l34
alphacoronavirus_2002−>2021
l1
l34
alphacoronavirus_1992−>2021
l1
l34

alphainfluenzavirus_NA−>NA

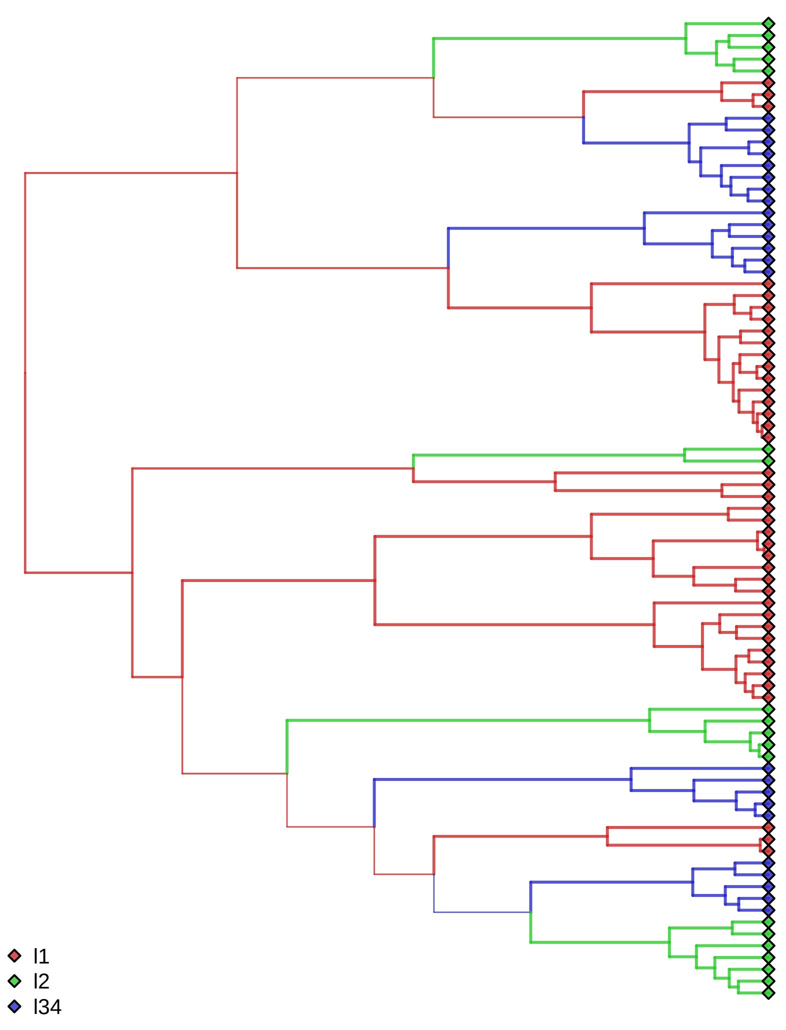

alphainfluenzavirus_2012−>2021

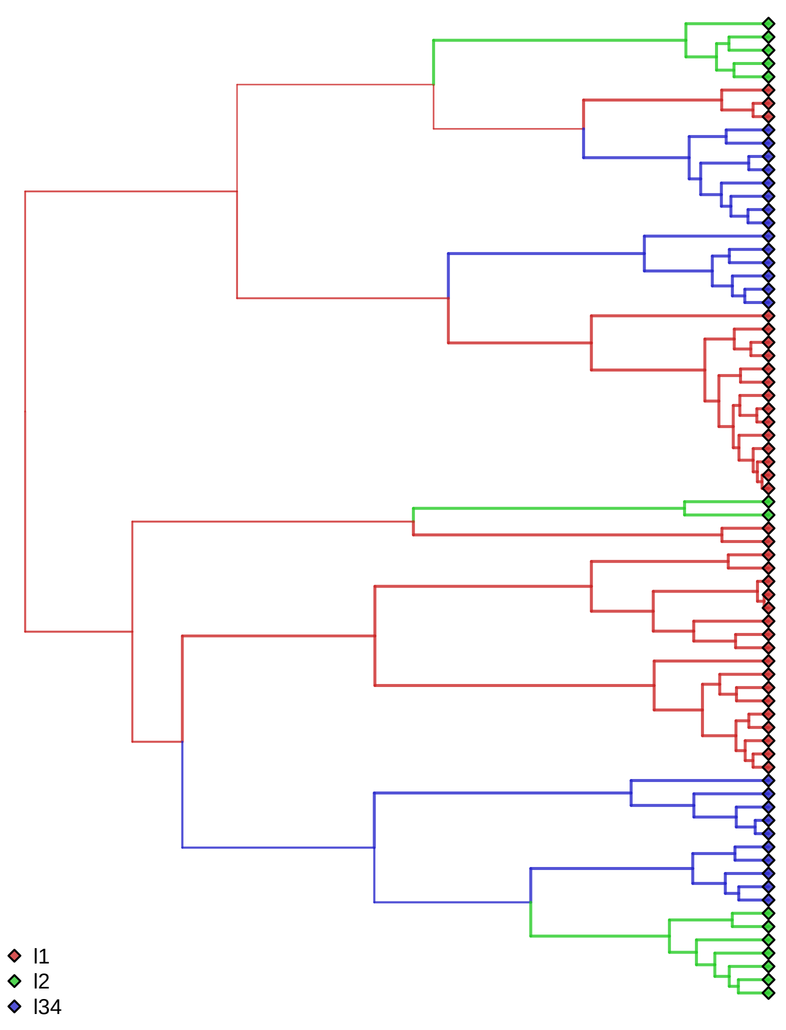

alphainfluenzavirus_2002−>2021

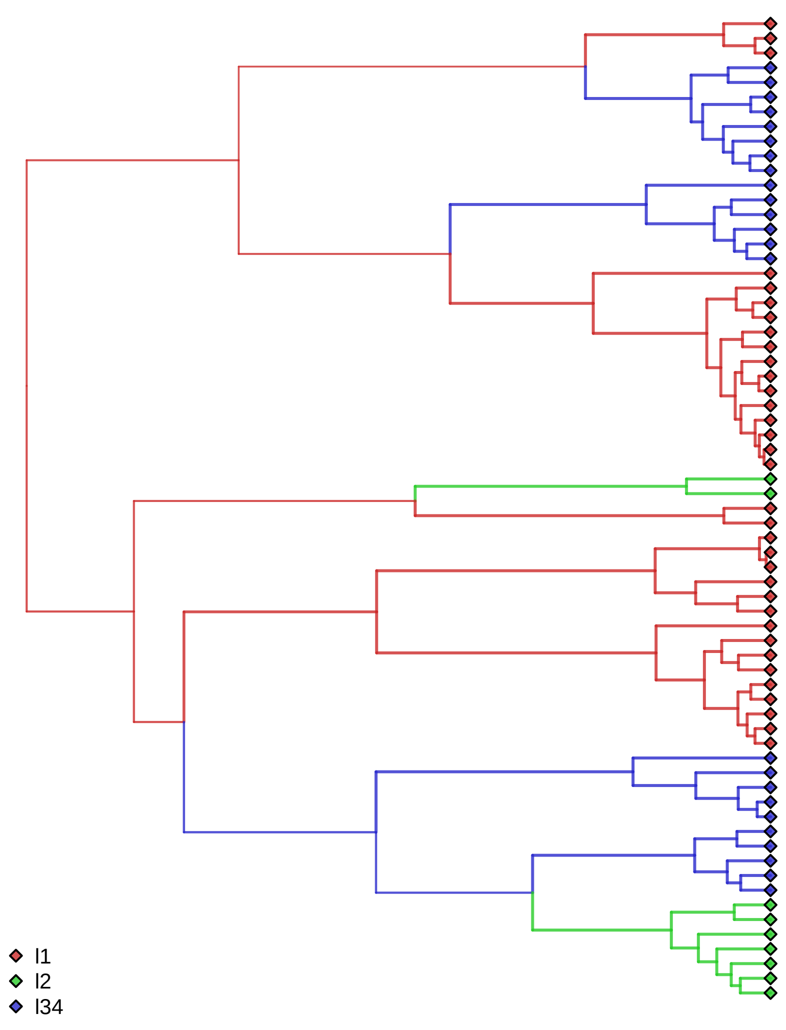

alphainfluenzavirus_1992−>2021

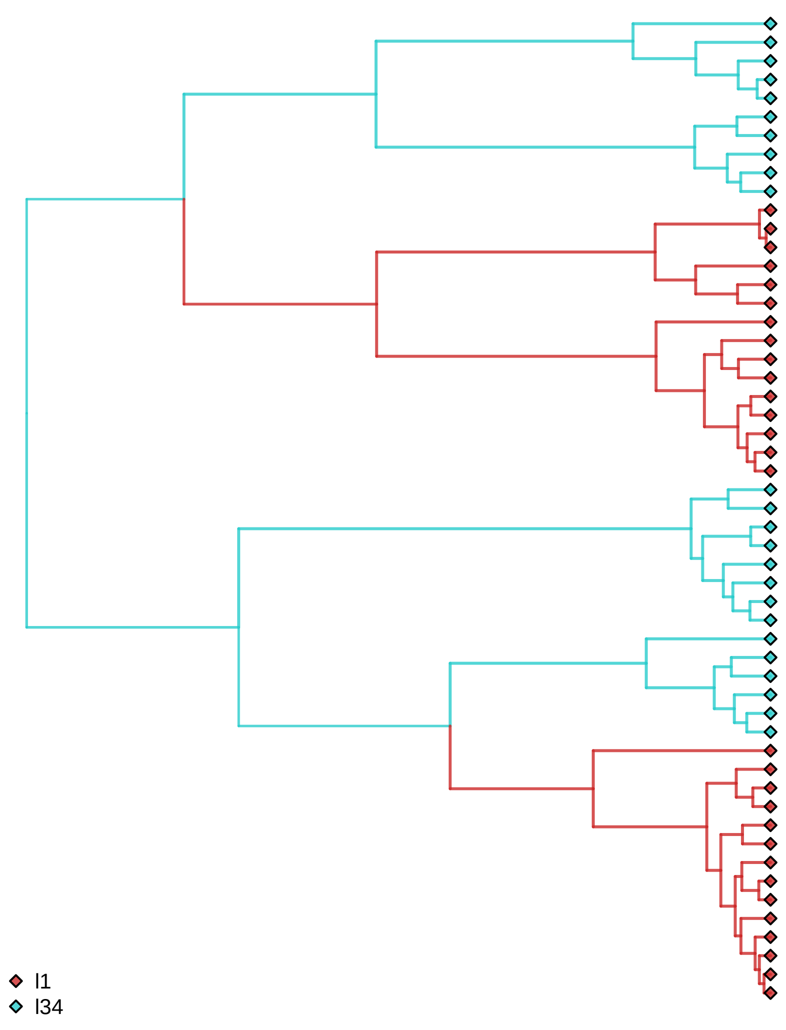

alphainfluenzavirus_1982−>2021

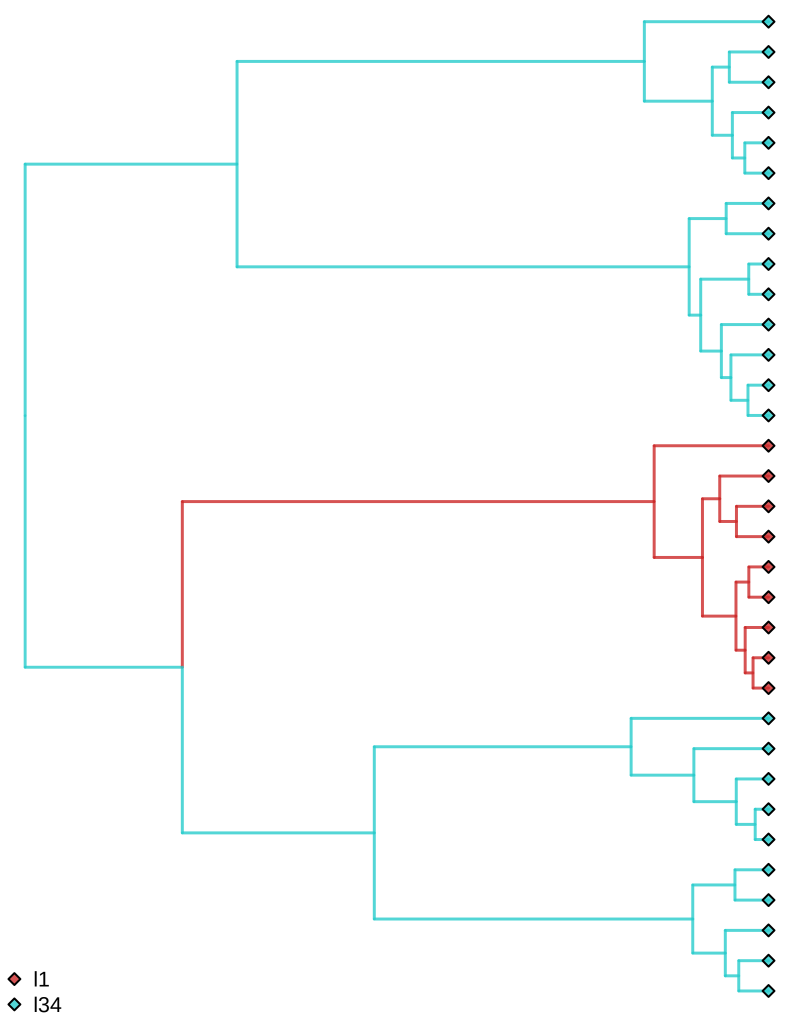

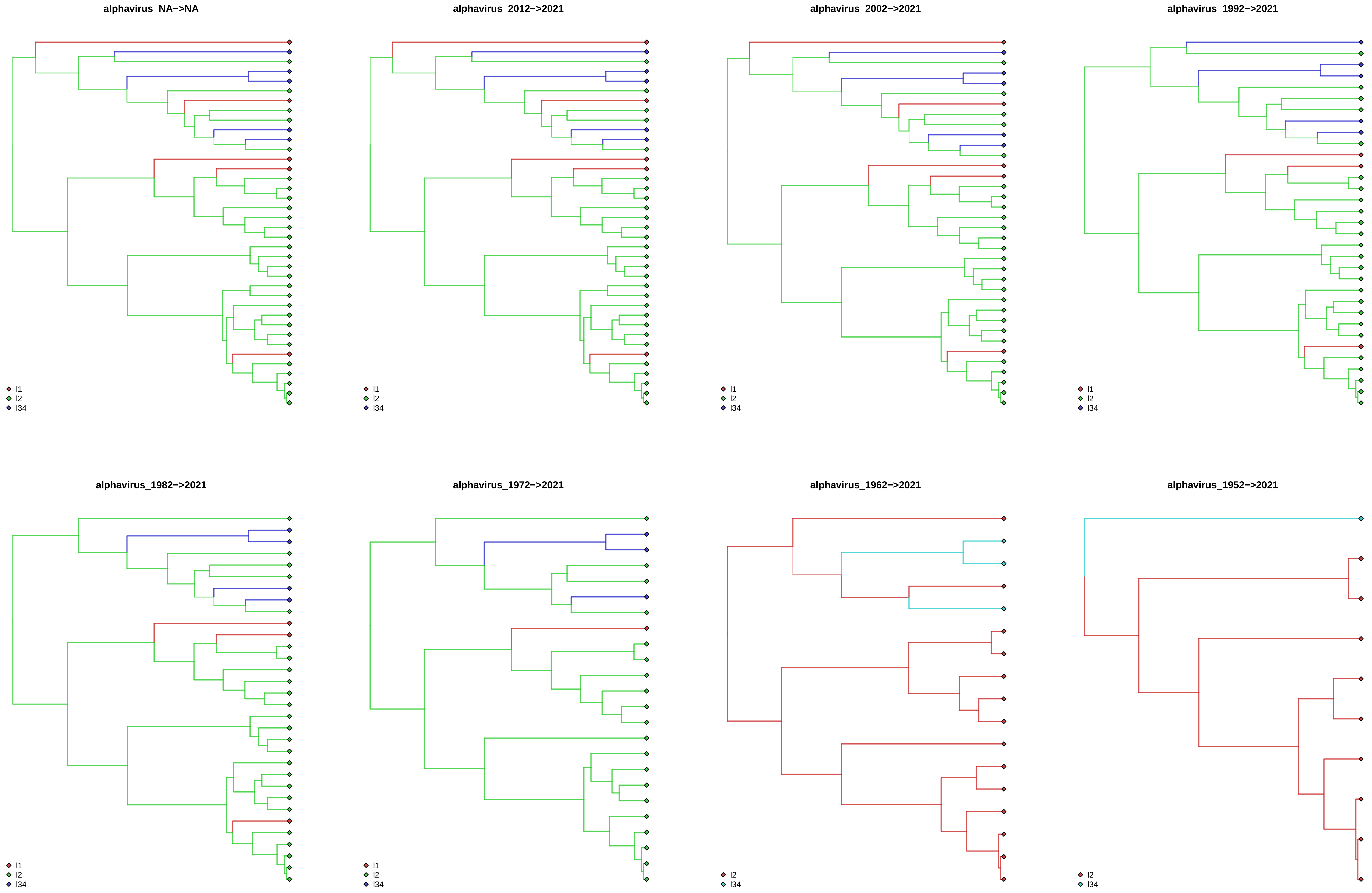
alphavirus_NA−>NA
I1
I2
I34
alphavirus_2012−>2021
I1
I2
I34
alphavirus_2002−>2021
I1
I2
I34
alphavirus_1992−>2021
I1
I2
I34
alphavirus_1982−>2021
I1
I2
I34
alphavirus_1972−>2021
I1
I2
I34
alphavirus_1962−>2021
I2
I34
alphavirus_1952−>2021
I2
I34

aphthovirus_NA−>NA

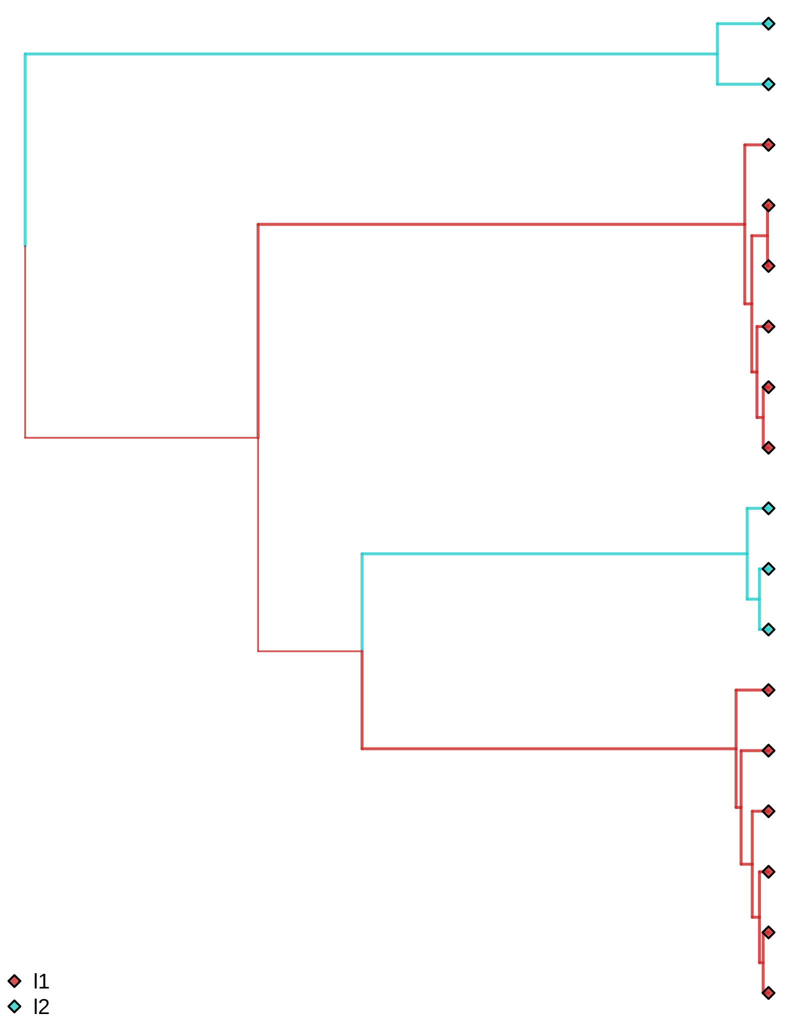

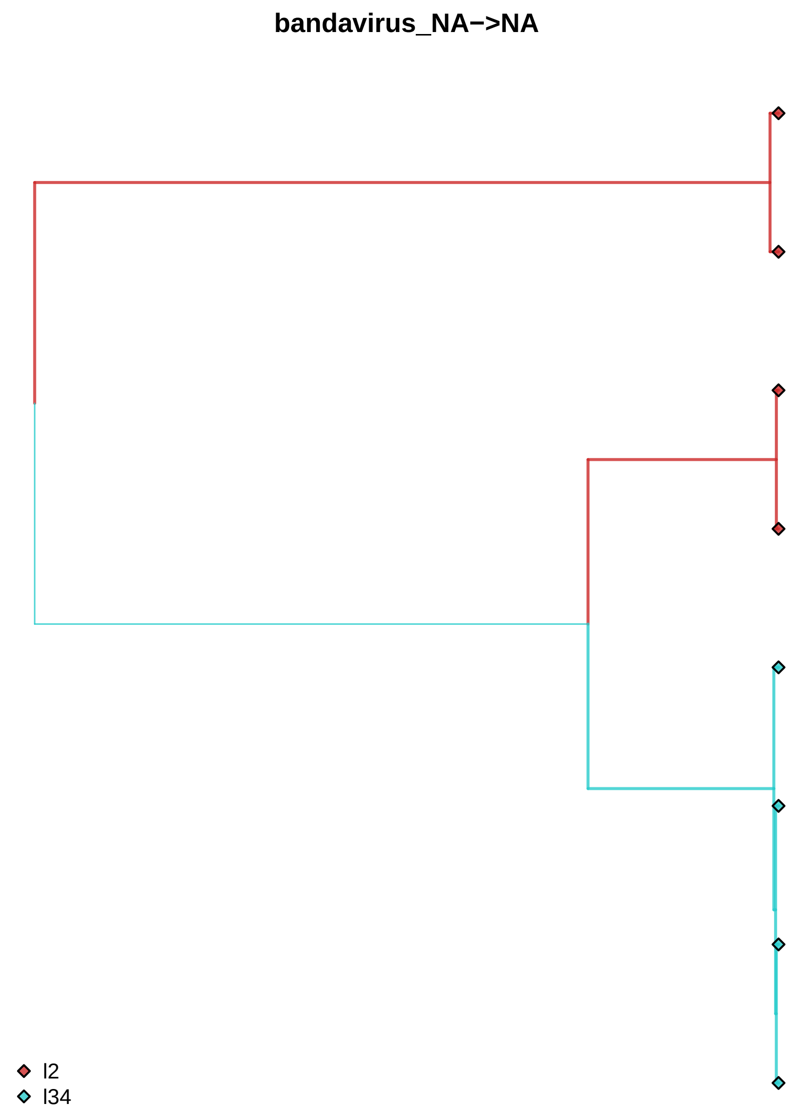
bandavirus_NA−>NA
l2
l34

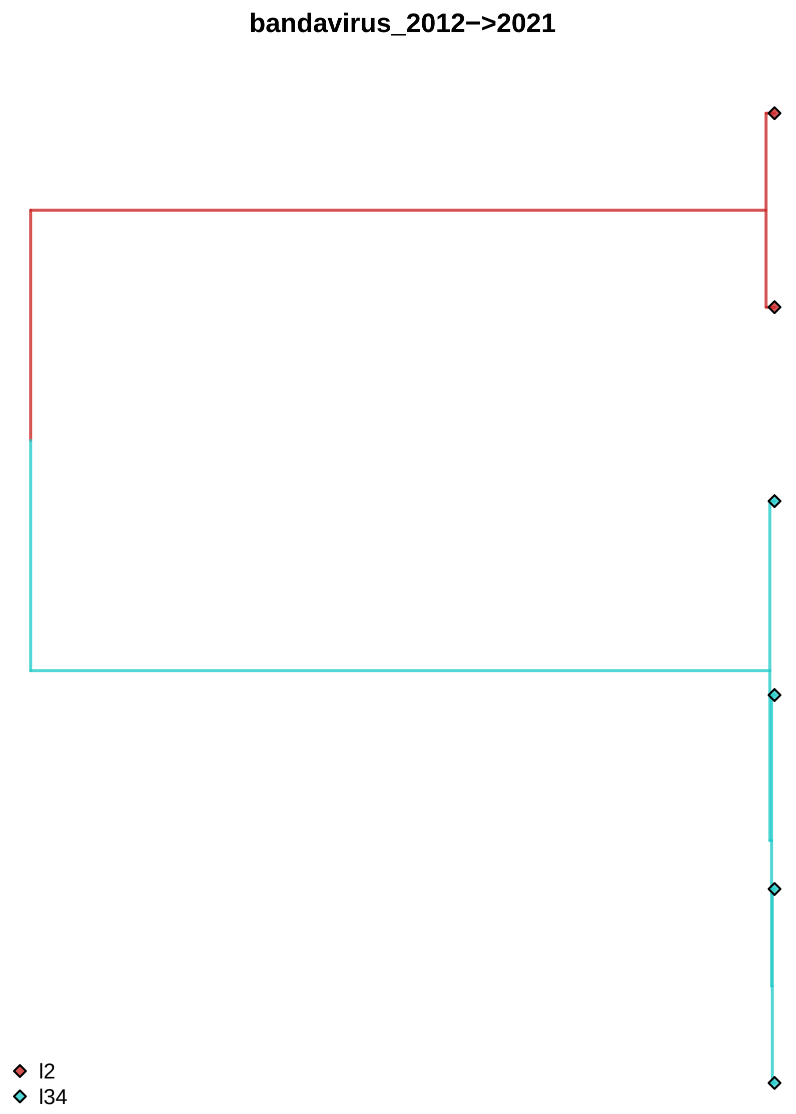
bandavirus_2012−>2021
l2
l34

betacoronavirus_NA−>NA

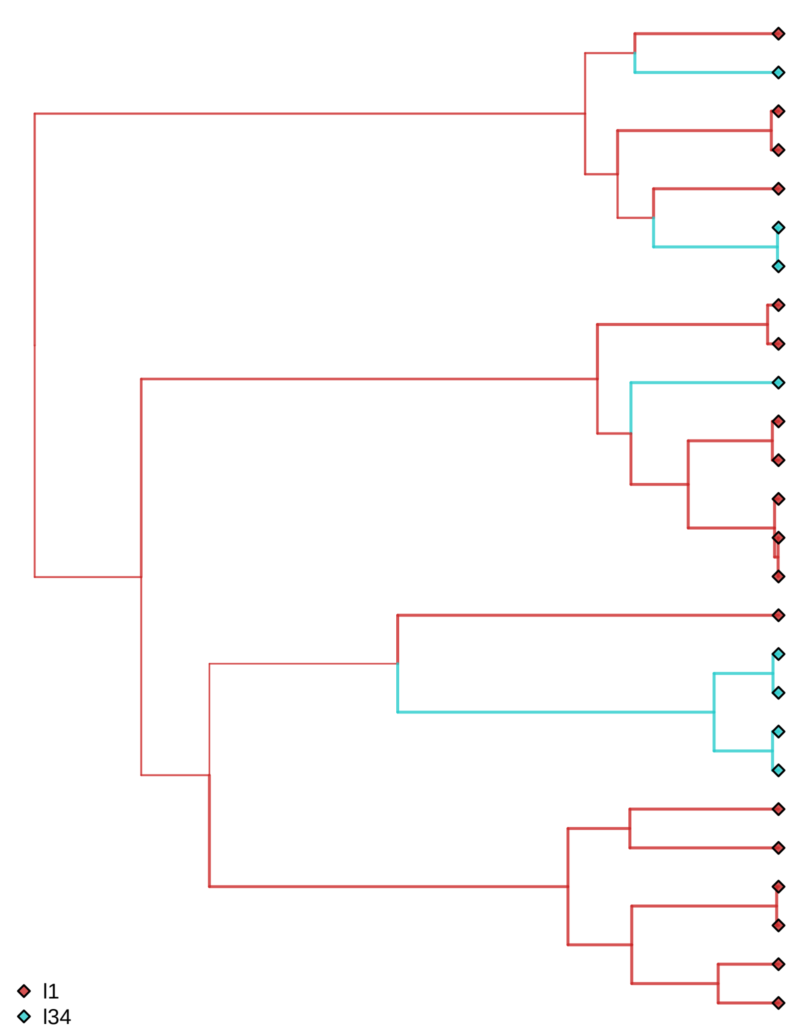

betacoronavirus_2012−>2021

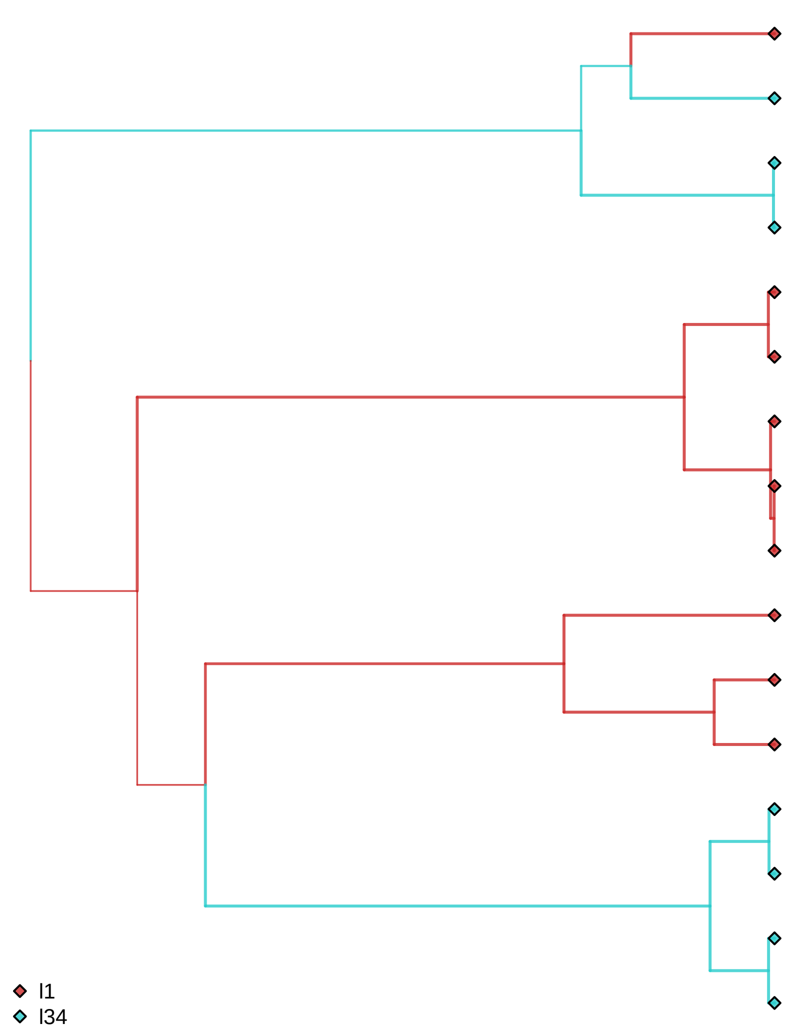

betacoronavirus_2002−>2021

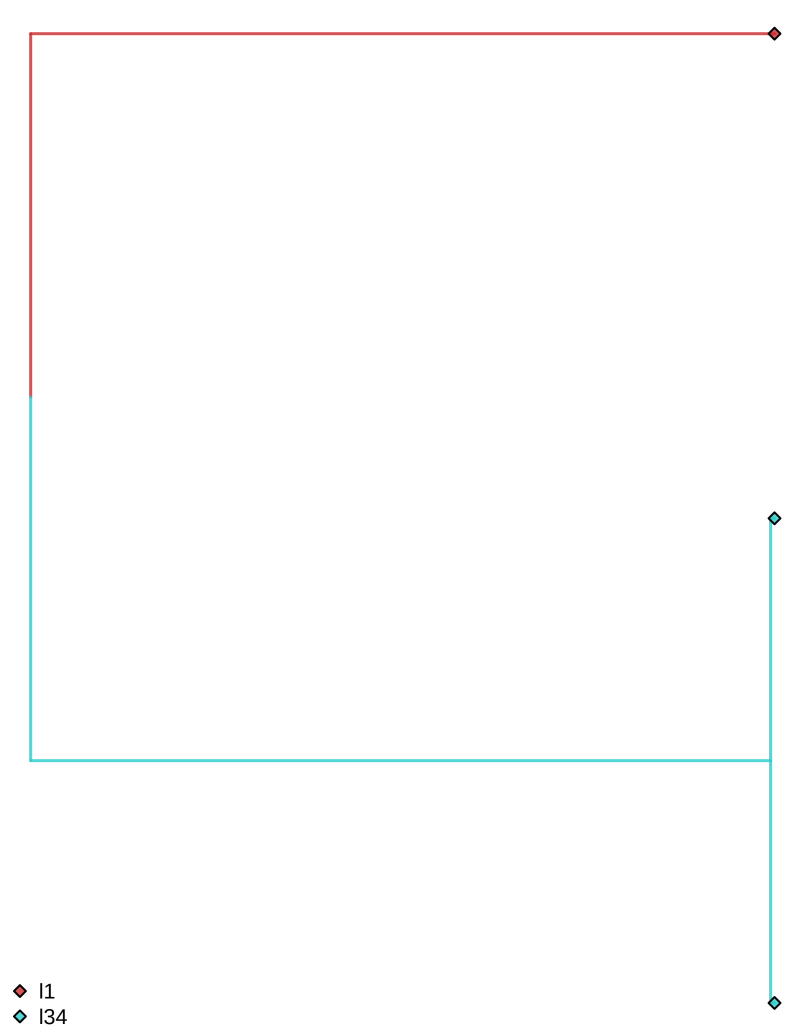

betacoronavirus_1992−>2021

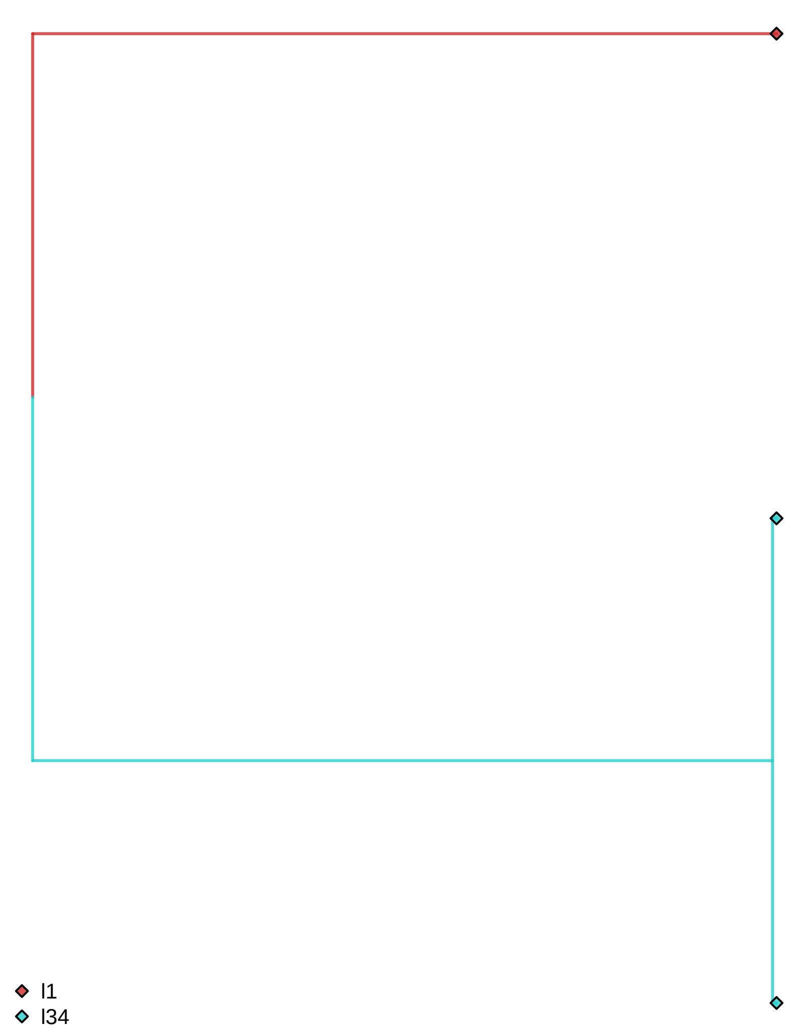

betacoronavirus_1982−>2021

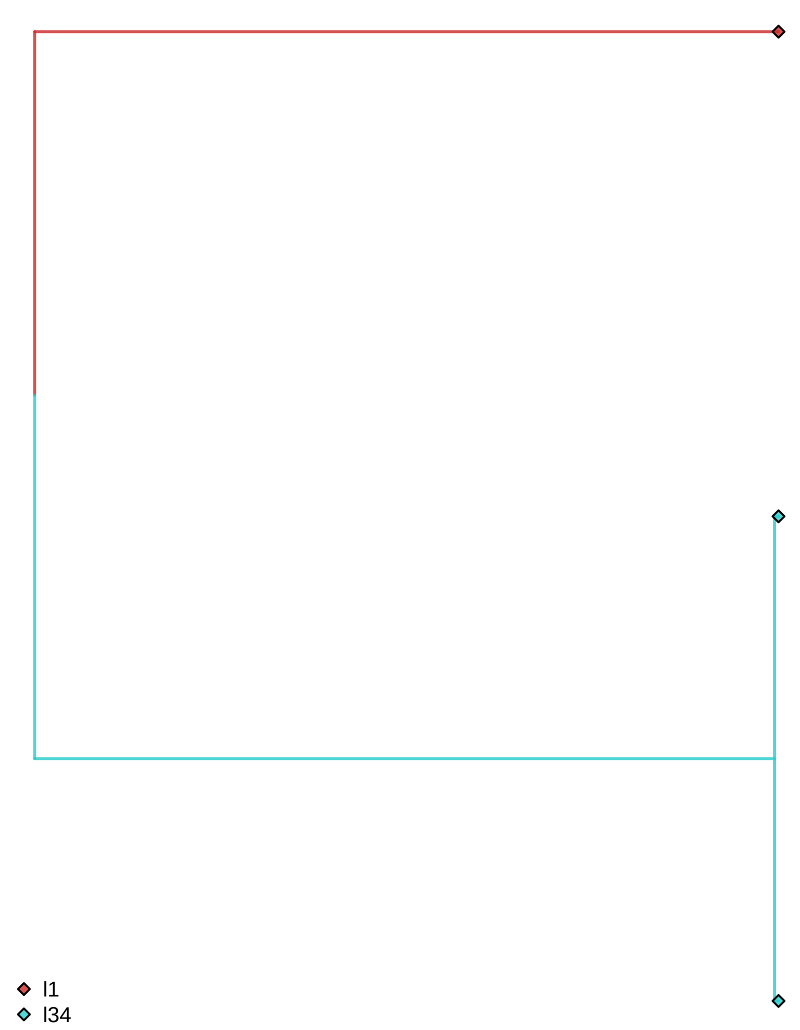

betacoronavirus_1972−>2021

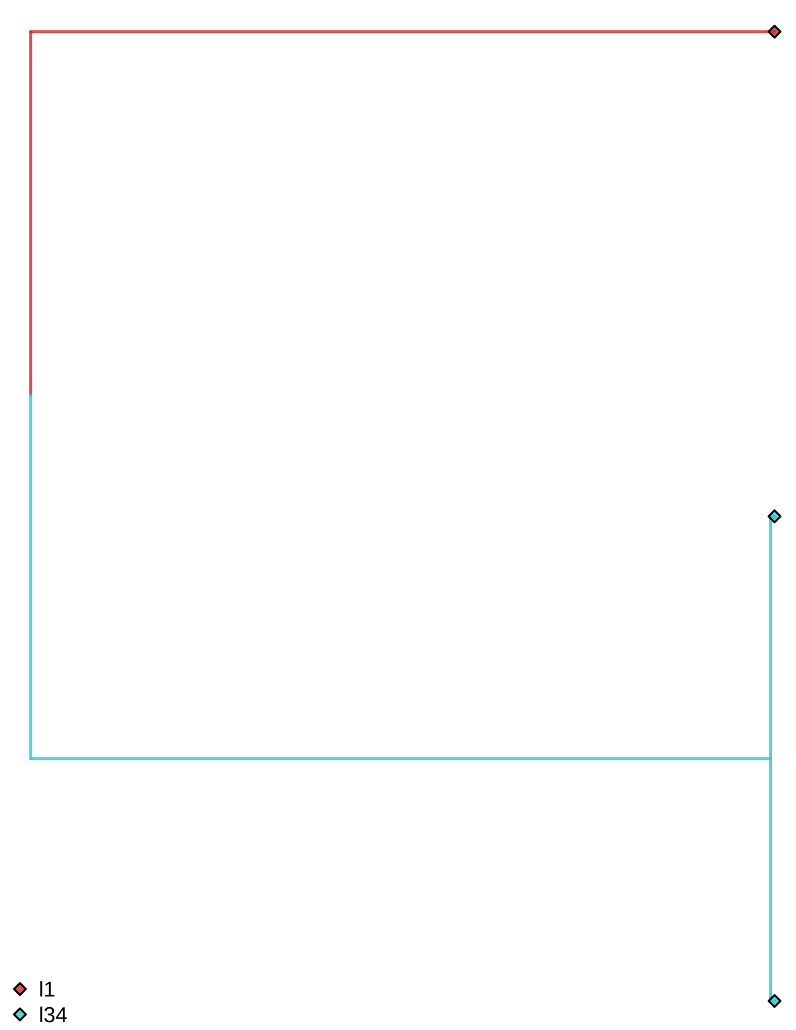

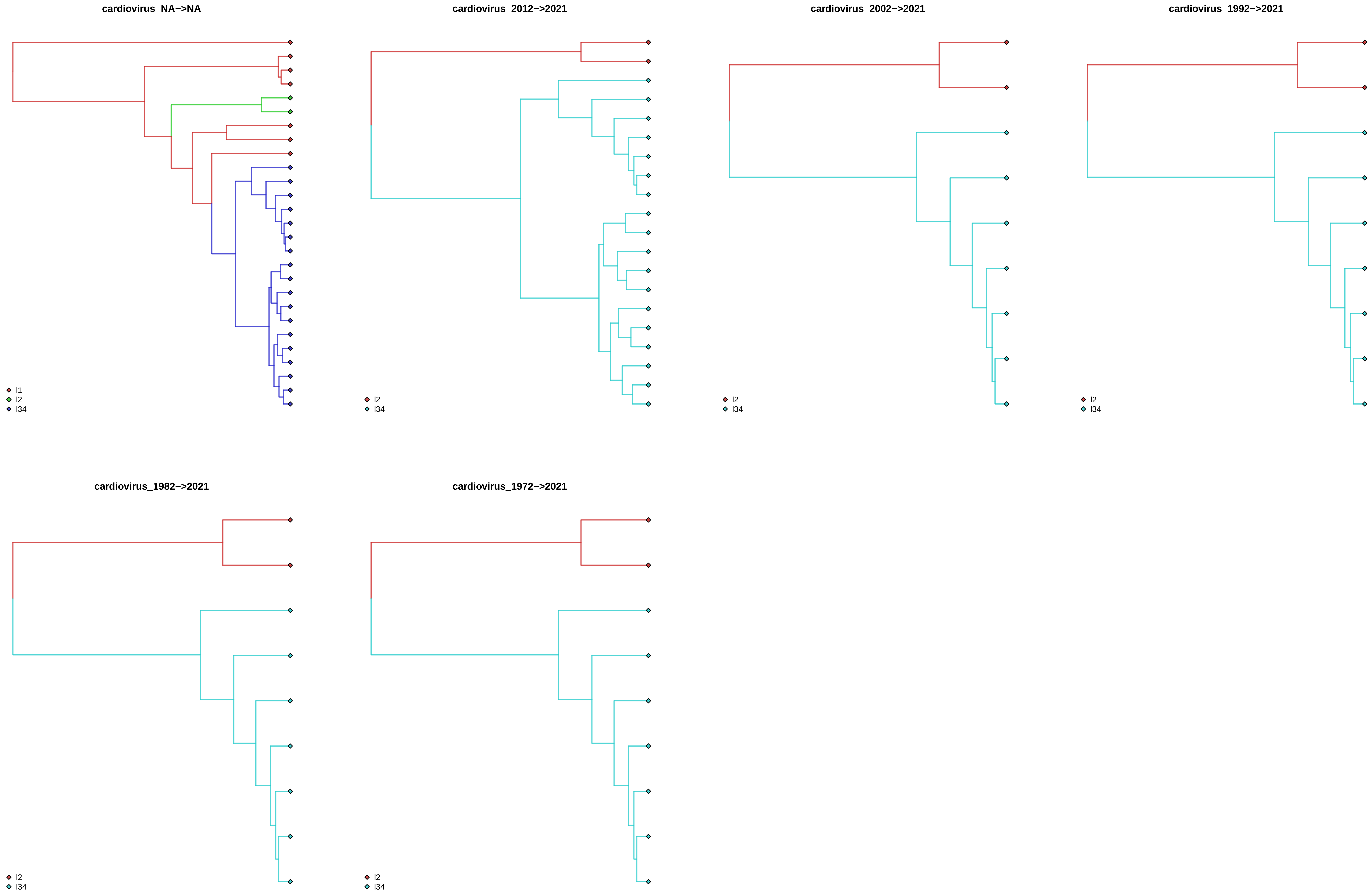
cardiovirus_NA−>NA
l1
l2
l34
cardiovirus_2012−>2021
l2
l34
cardiovirus_2002−>2021
l2
l34
cardiovirus_1992−>2021
l2
l34
cardiovirus_1982−>2021
l2
l34
cardiovirus_1972−>2021
l2
l34

coltivirus_NA−>NA

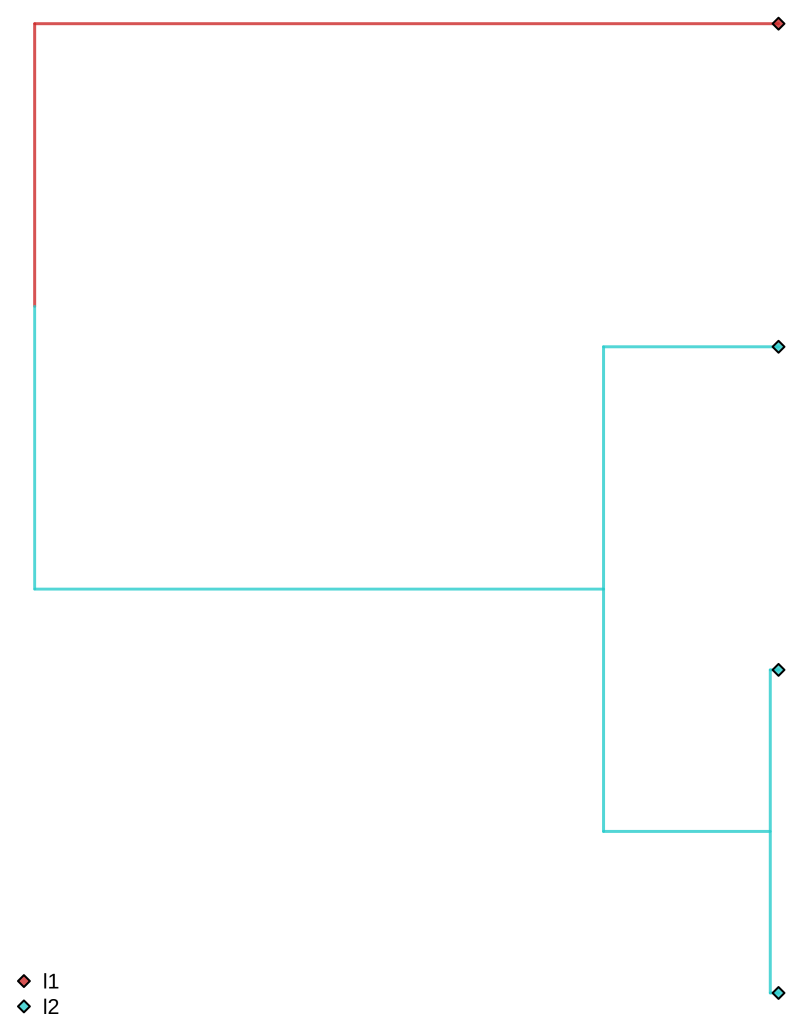

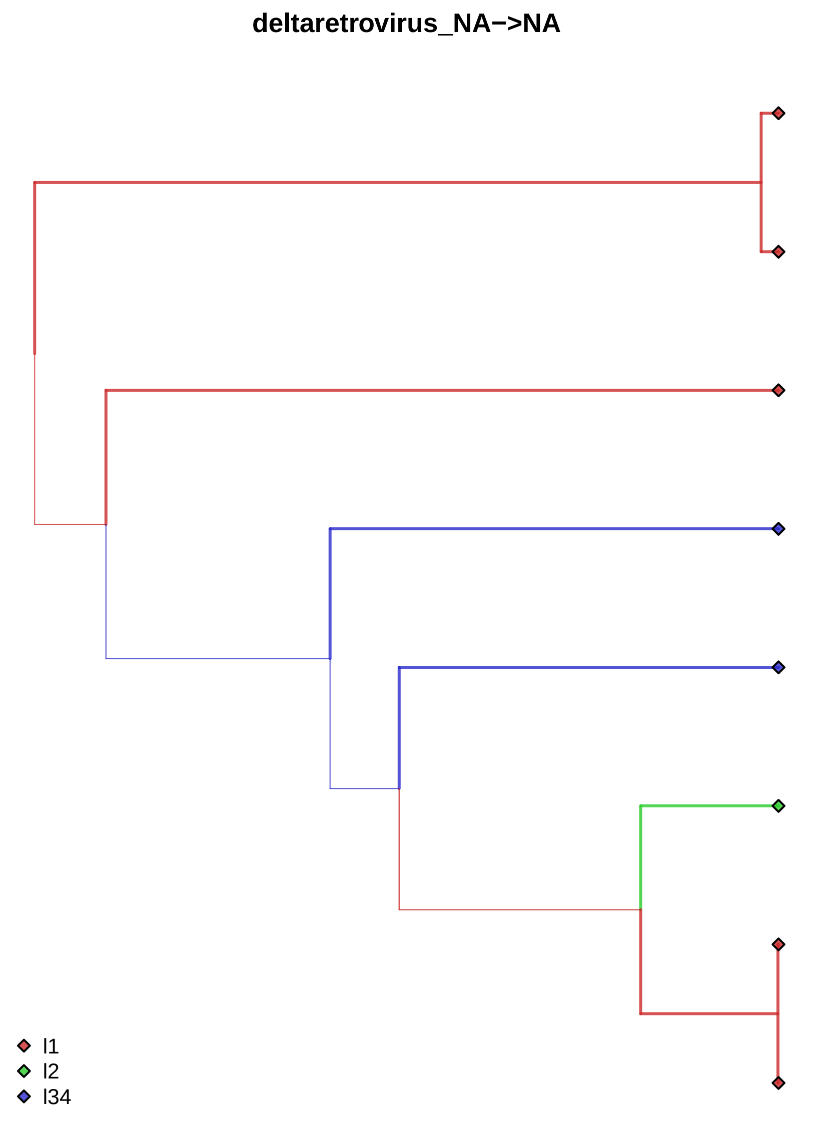
deltaretrovirus_NA−>NA
l1
l2
l34
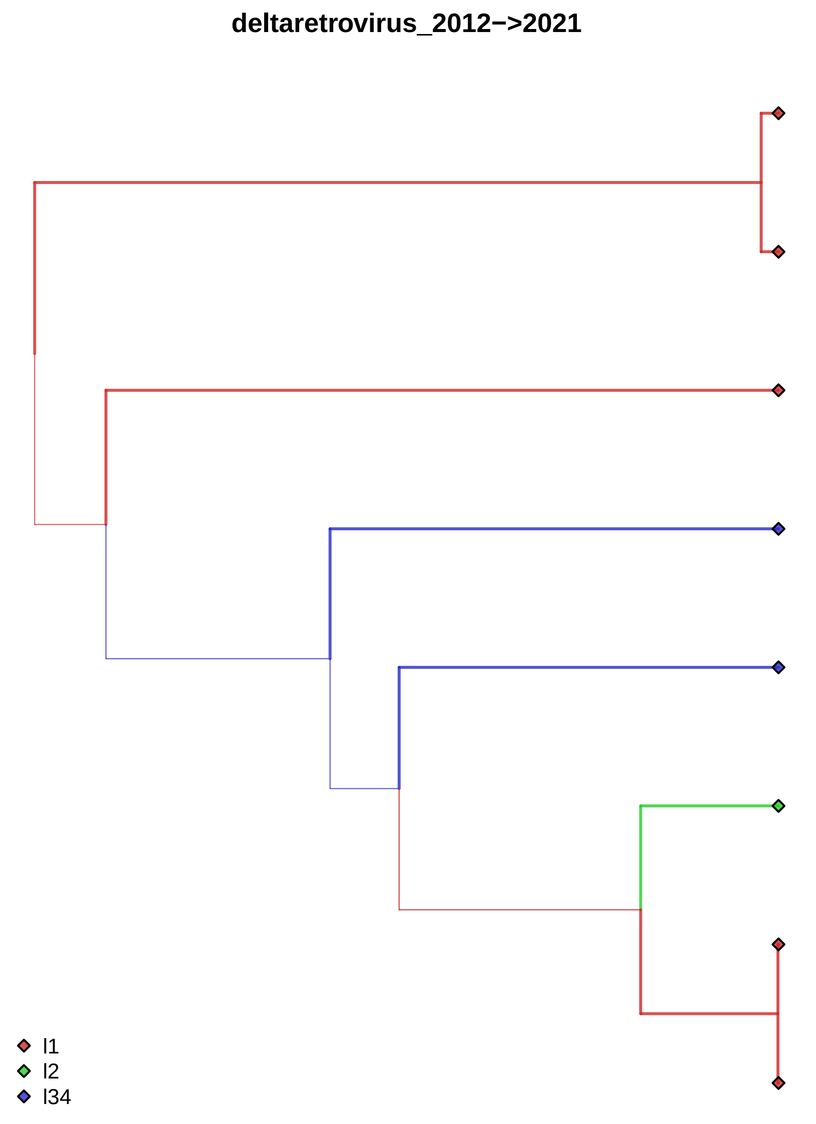
deltaretrovirus_2012−>2021
l1
l2
l34
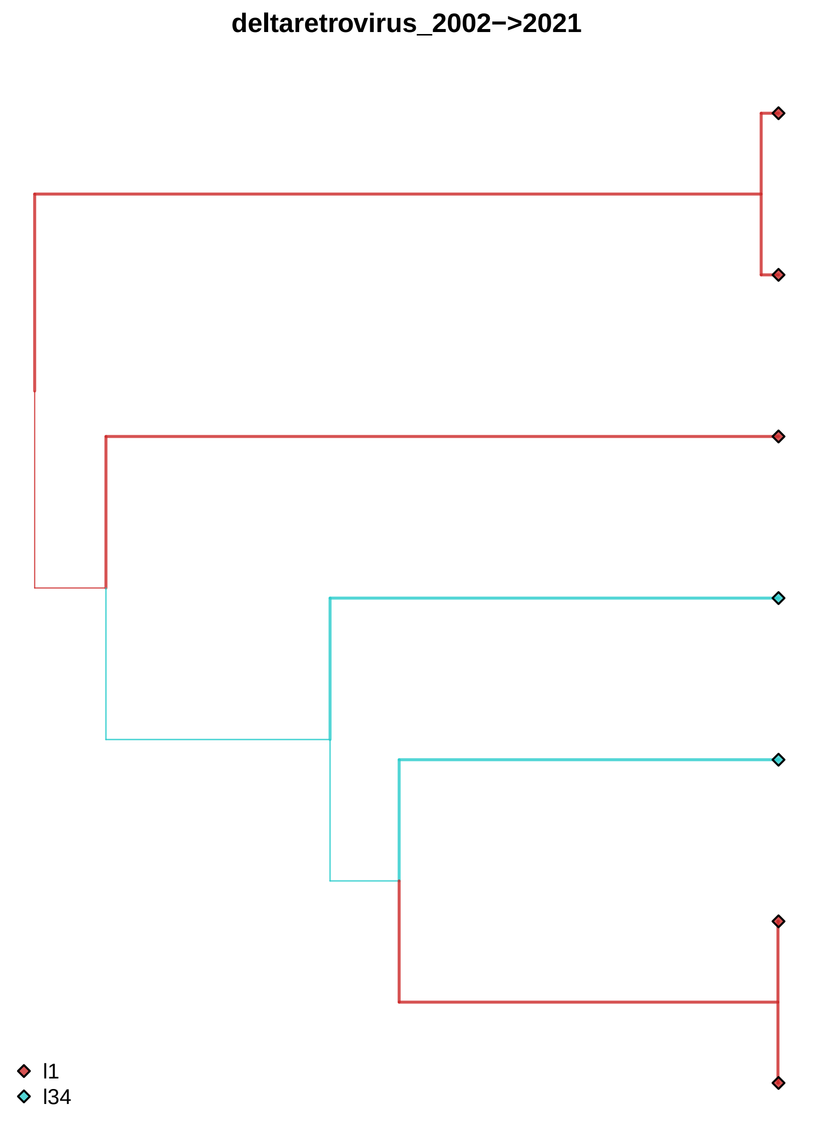
deltaretrovirus_2002−>2021
l1
l34
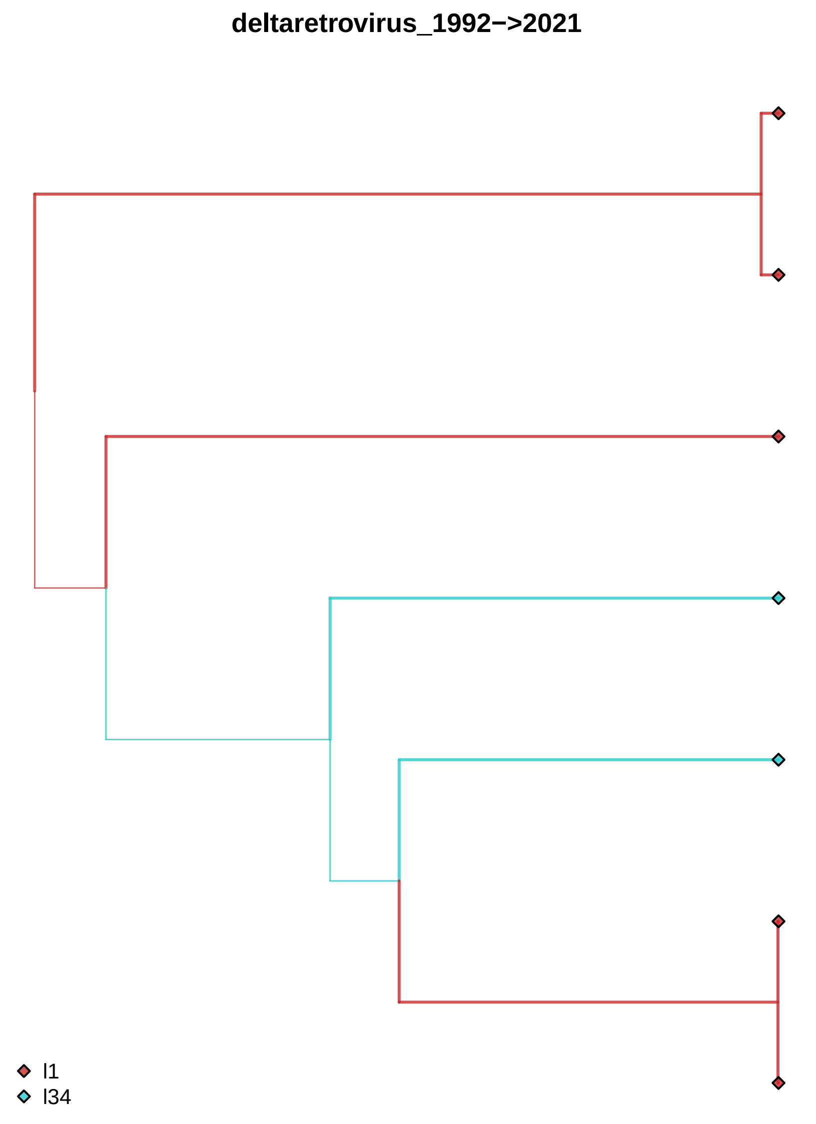
deltaretrovirus_1992−>2021
l1
l34
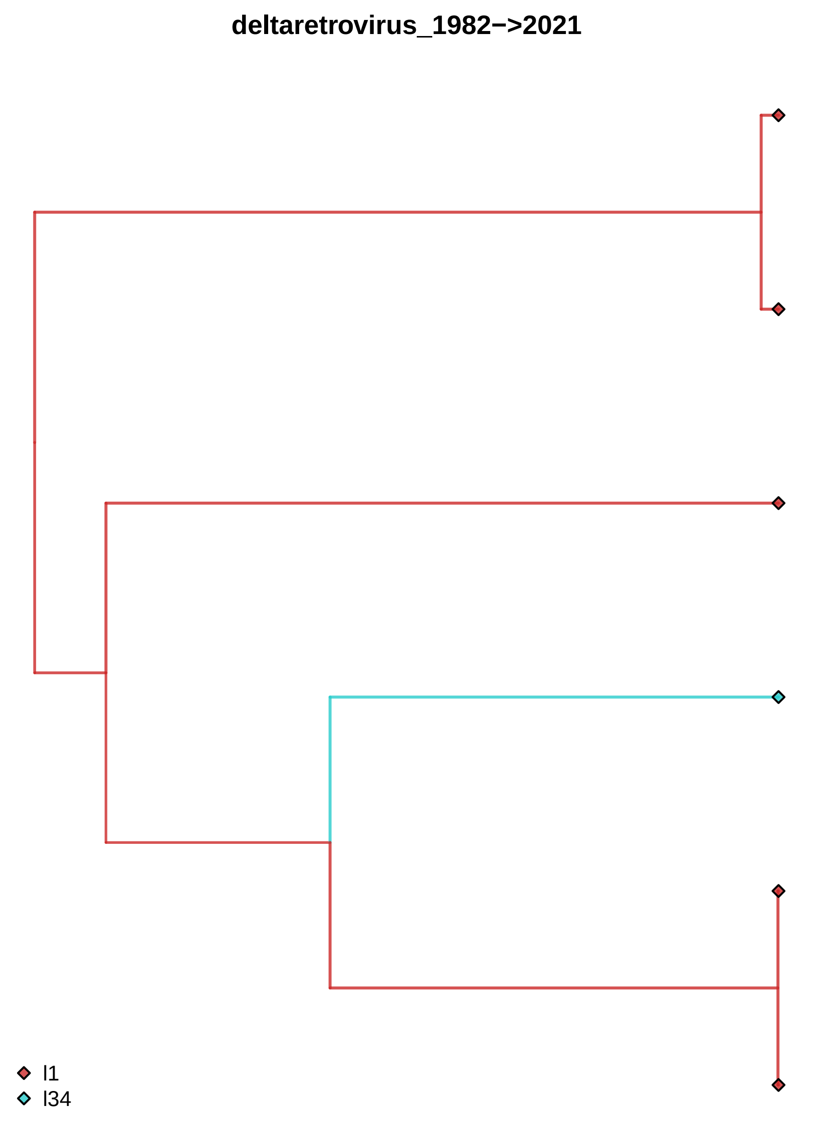
deltaretrovirus_1982−>2021
l1
l34

deltavirus_NA−>NA

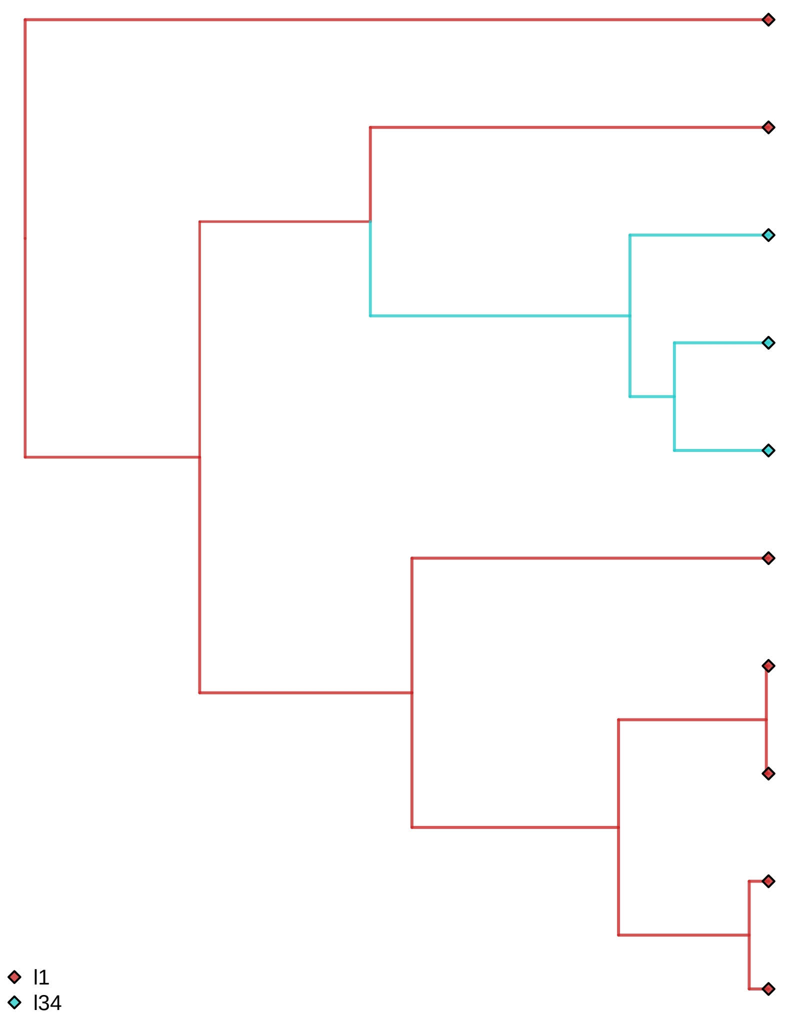

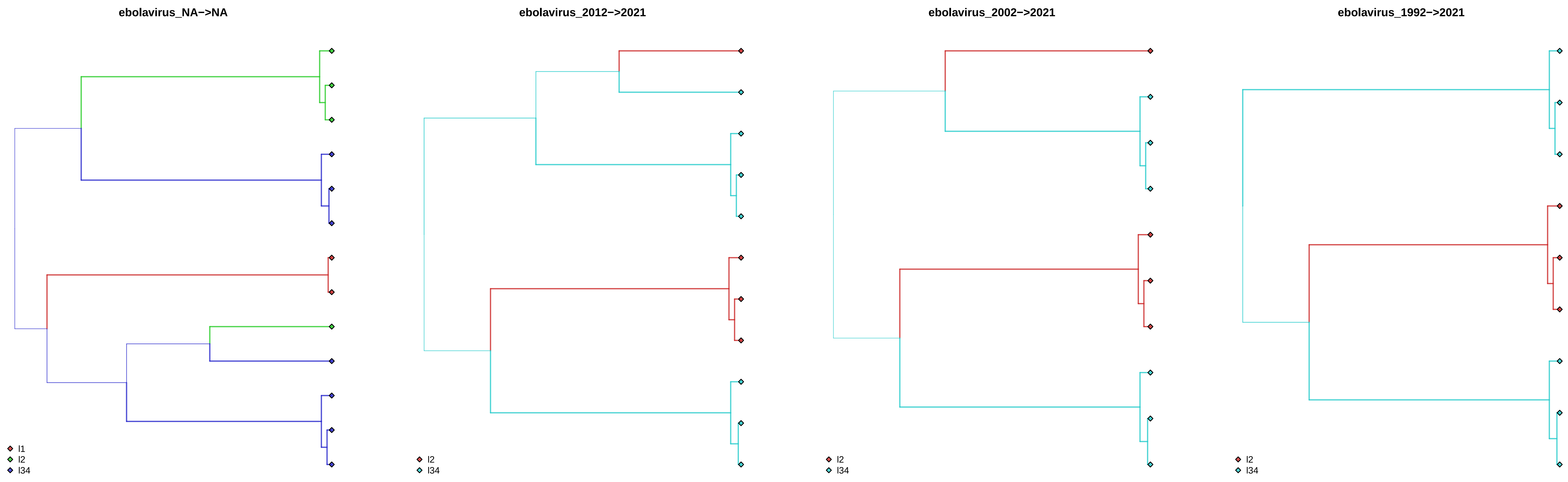
ebolavirus_NA−>NA
l1
l2
l34
ebolavirus_2012−>2021
l2
l34
ebolavirus_2002−>2021
l2
l34
ebolavirus_1992−>2021
l2
l34

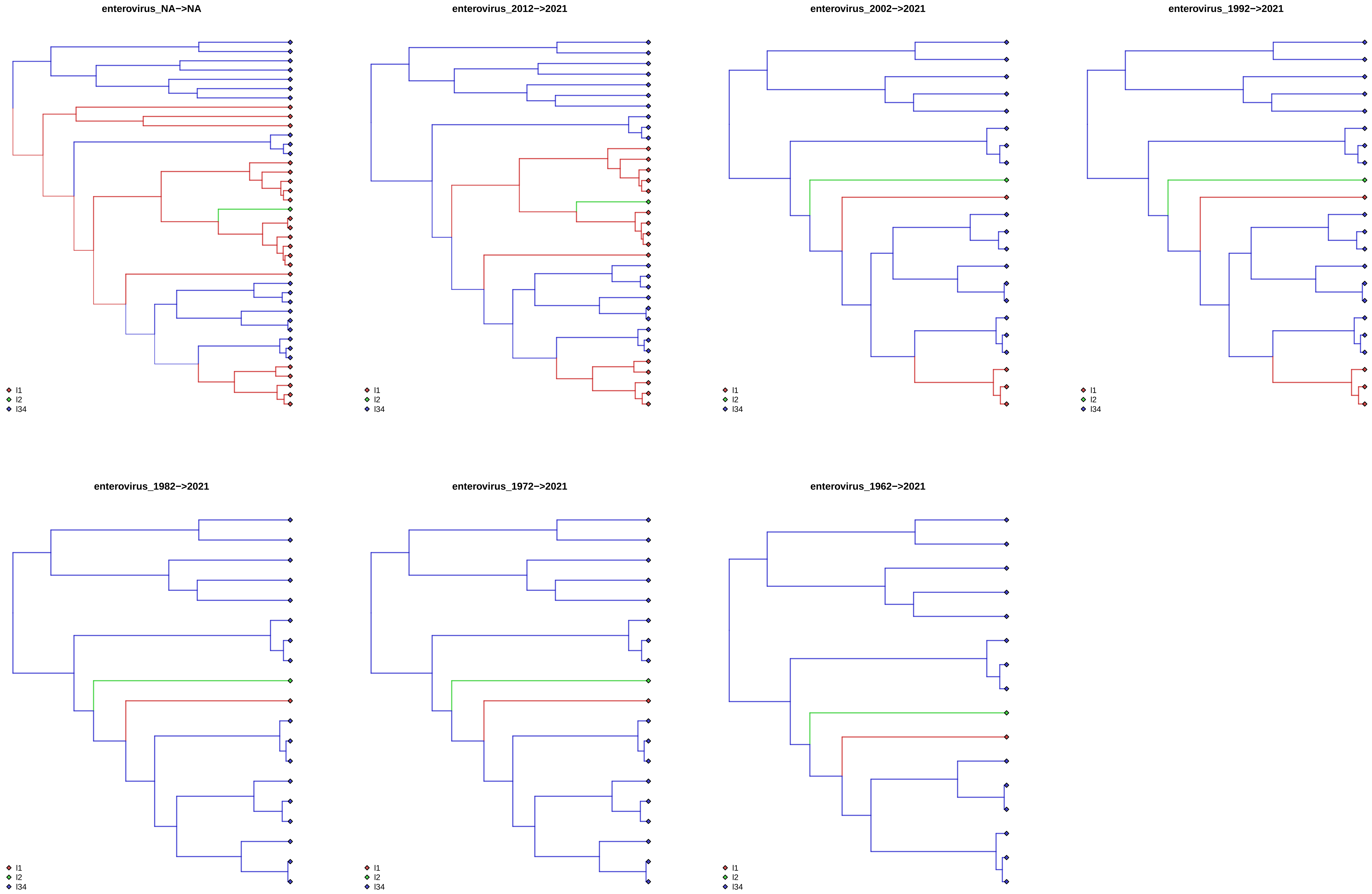
enterovirus_NA−>NA
I1
I2
I34
enterovirus_2012−>2021
I1
I2
I34
enterovirus_2002−>2021
I1
I2
I34
enterovirus_1992−>2021
I1
I2
I34
enterovirus_1982−>2021
I1
I2
I34
enterovirus_1972−>2021
I1
I2
I34
enterovirus_1962−>2021
I1
I2
I34

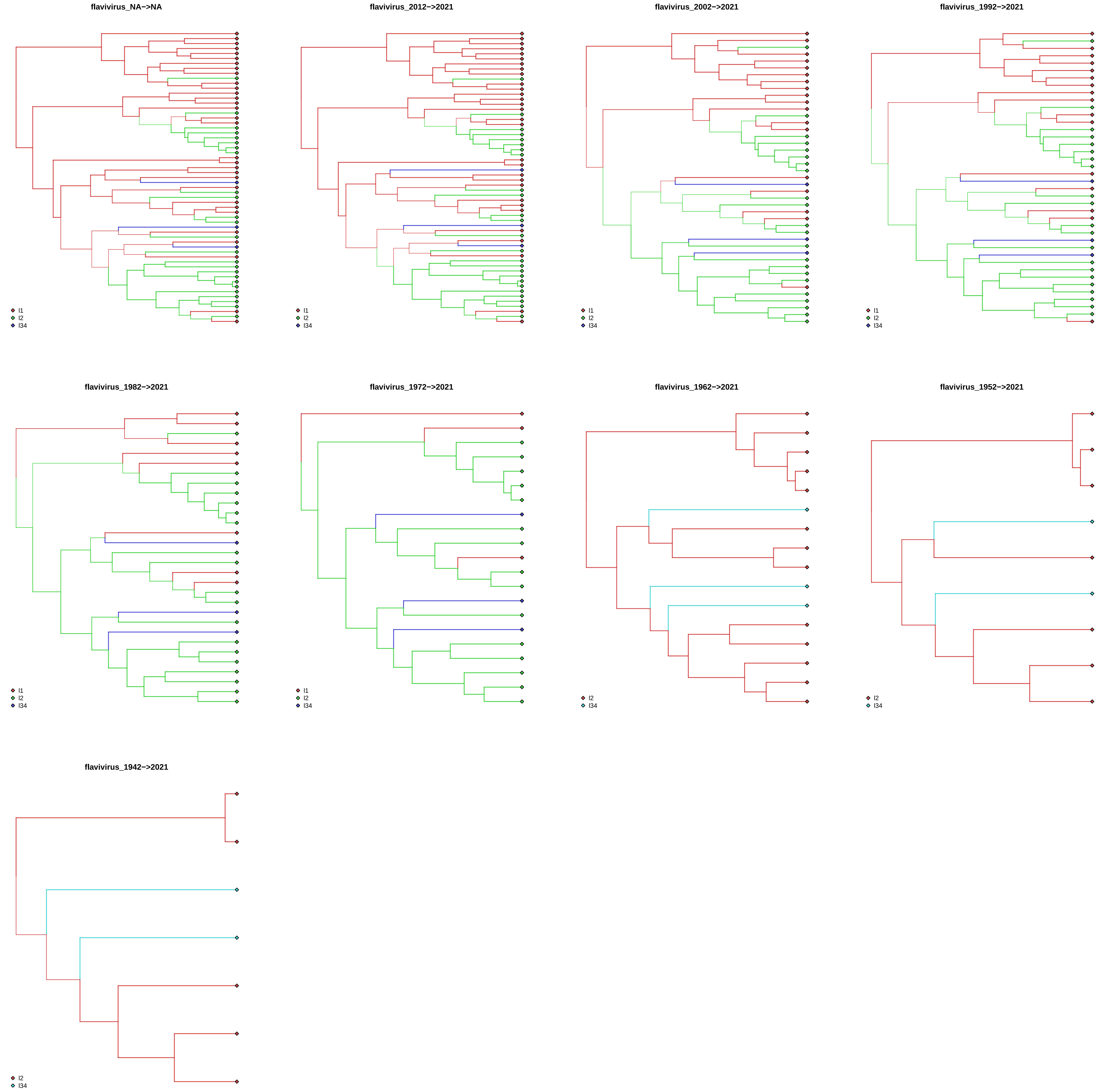
flavivirus_NA−>NA
I1
I2
I34
flavivirus_2012−>2021
I1
I2
I34
flavivirus_2002−>2021
I1
I2
I34
flavivirus_1992−>2021
I1
I2
I34
flavivirus_1982−>2021
I1
I2
I34
flavivirus_1972−>2021
I1
I2
I34
flavivirus_1962−>2021
I2
I34
flavivirus_1952−>2021
I2
I34
flavivirus_1942−>2021
I2
I34

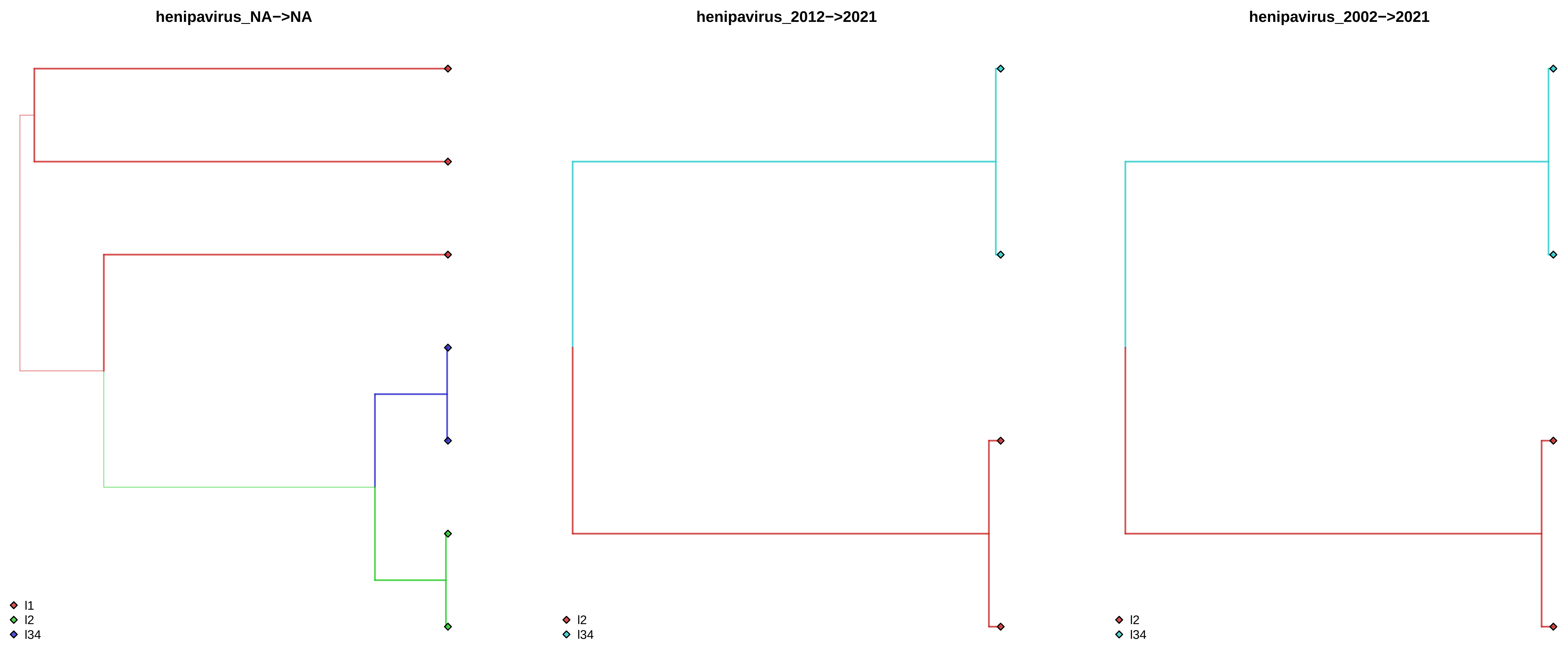
henipavirus_NA−>NA
l1
l2
l34
henipavirus_2012−>2021
l2
l34
henipavirus_2002−>2021
l2
l34

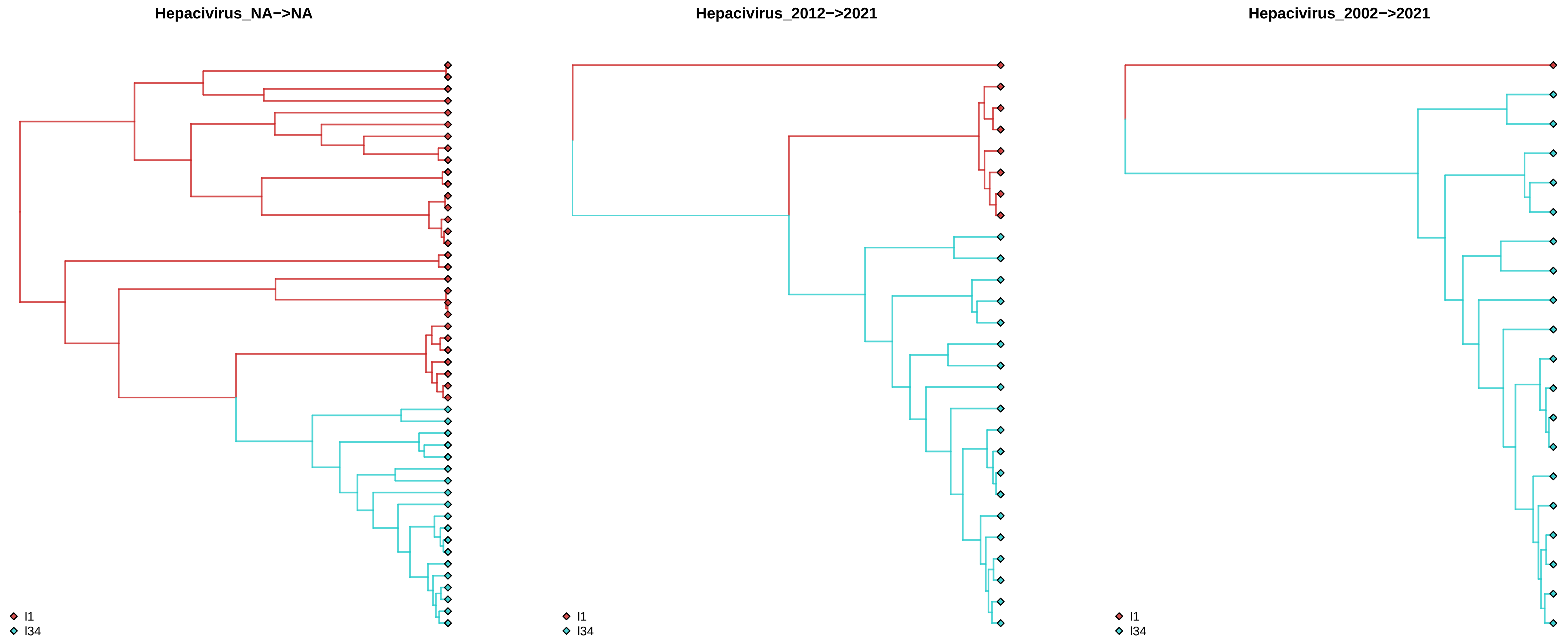
Hepacivirus_NA−>NA
Hepacivirus_2012−>2021
Hepacivirus_2002−>2021
l1
l34
l1
l34
l1
l34

hepatovirus_NA−>NA

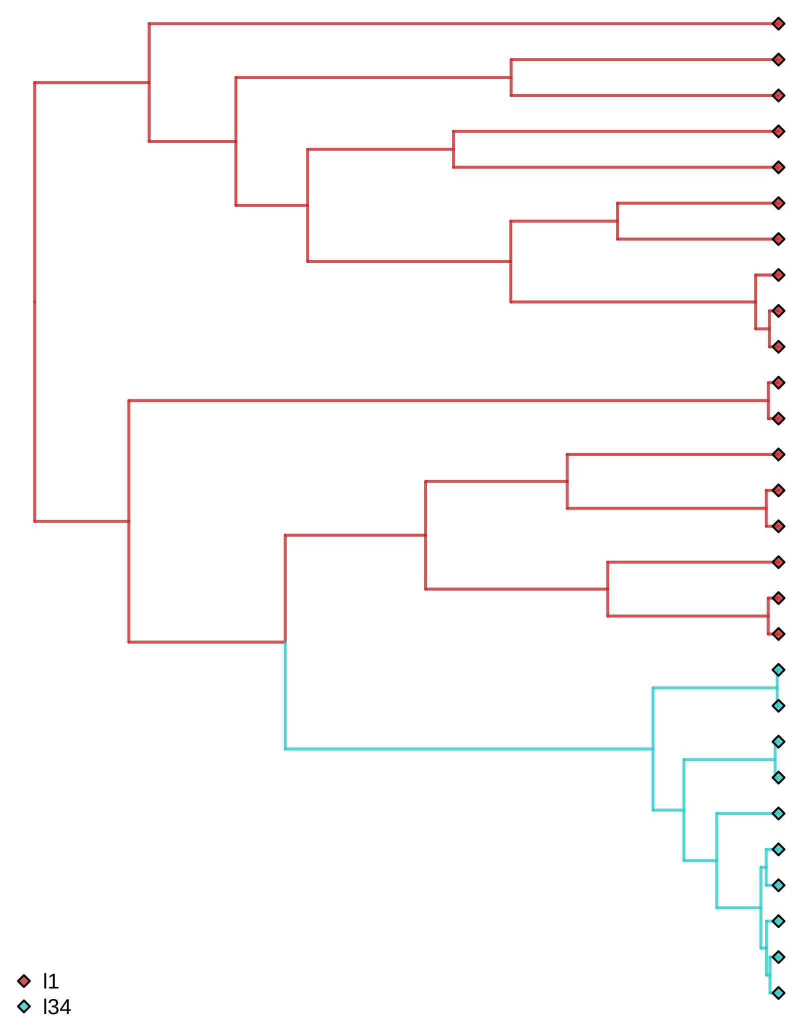

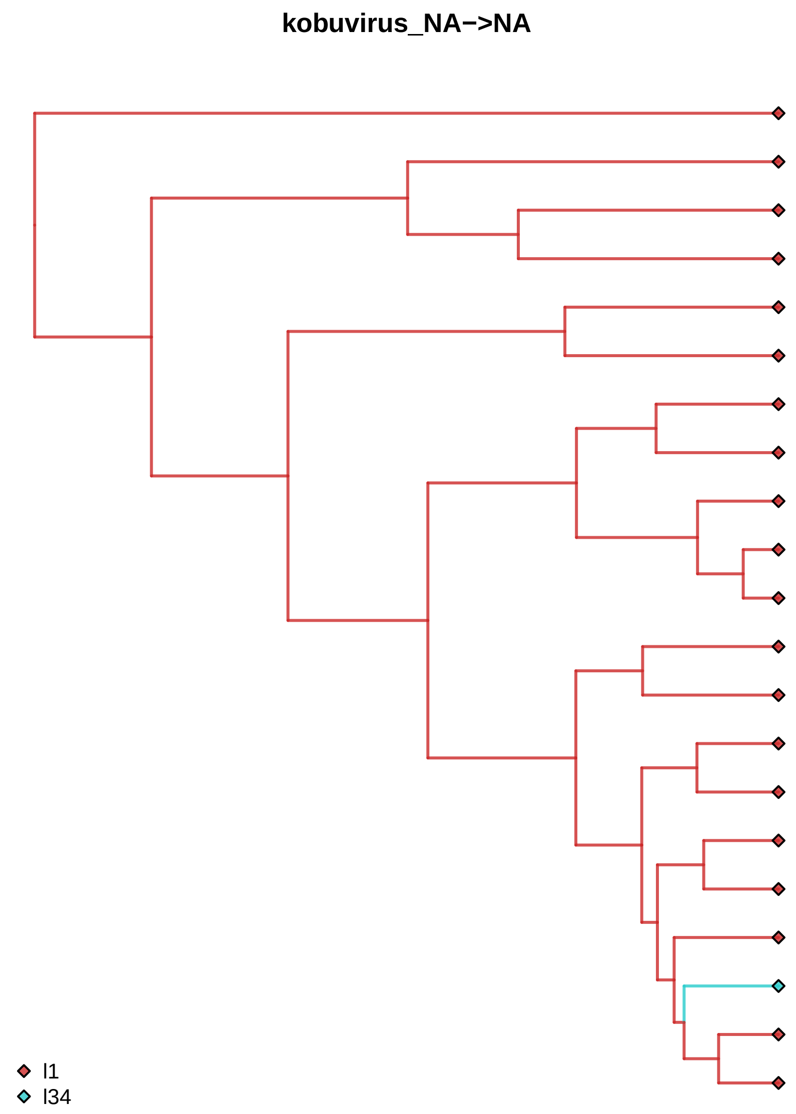
kobuvirus_NA−>NA
l1
l34

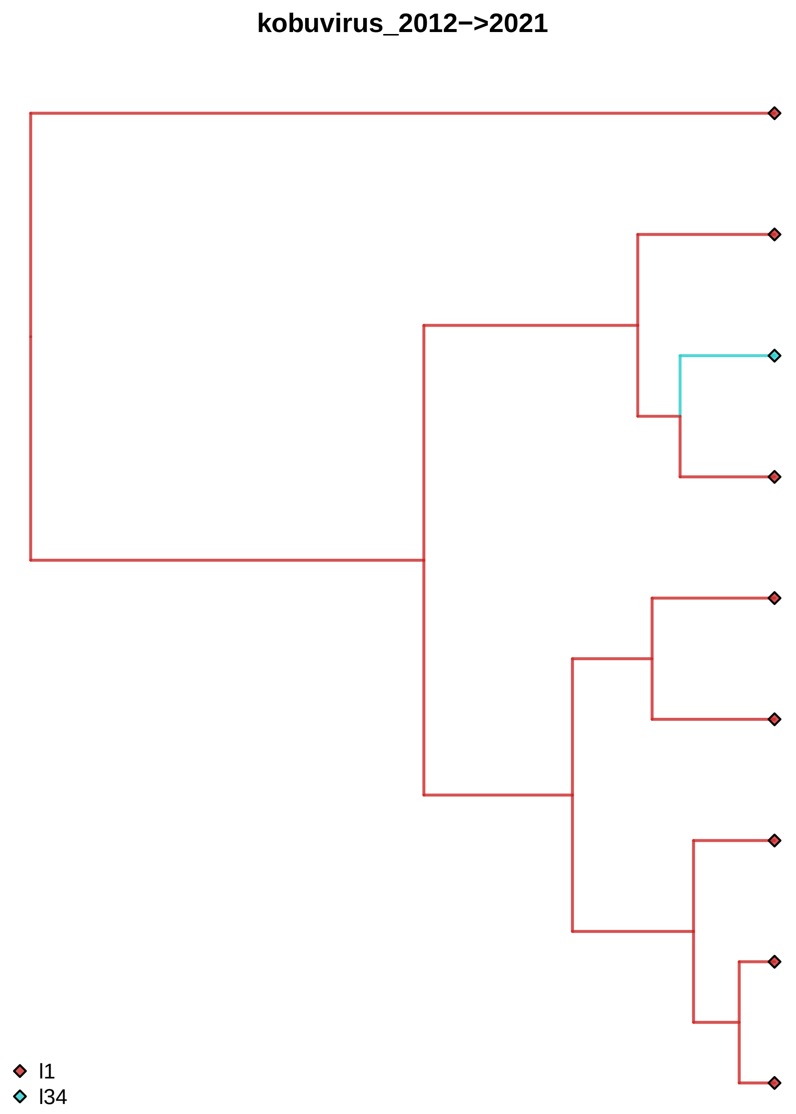
kobuvirus_2012−>2021
l1
l34

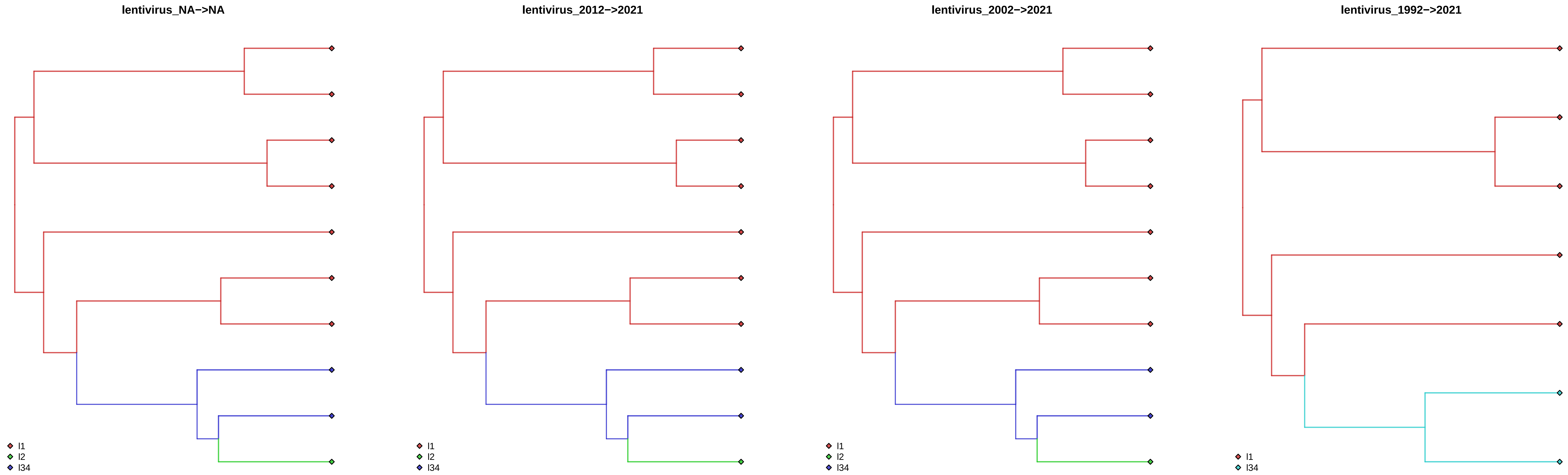
lentivirus_NA−>NA
l1
l2
l34
lentivirus_2012−>2021
l1
l2
l34
lentivirus_2002−>2021
l1
l2
l34
lentivirus_1992−>2021
l1
l34

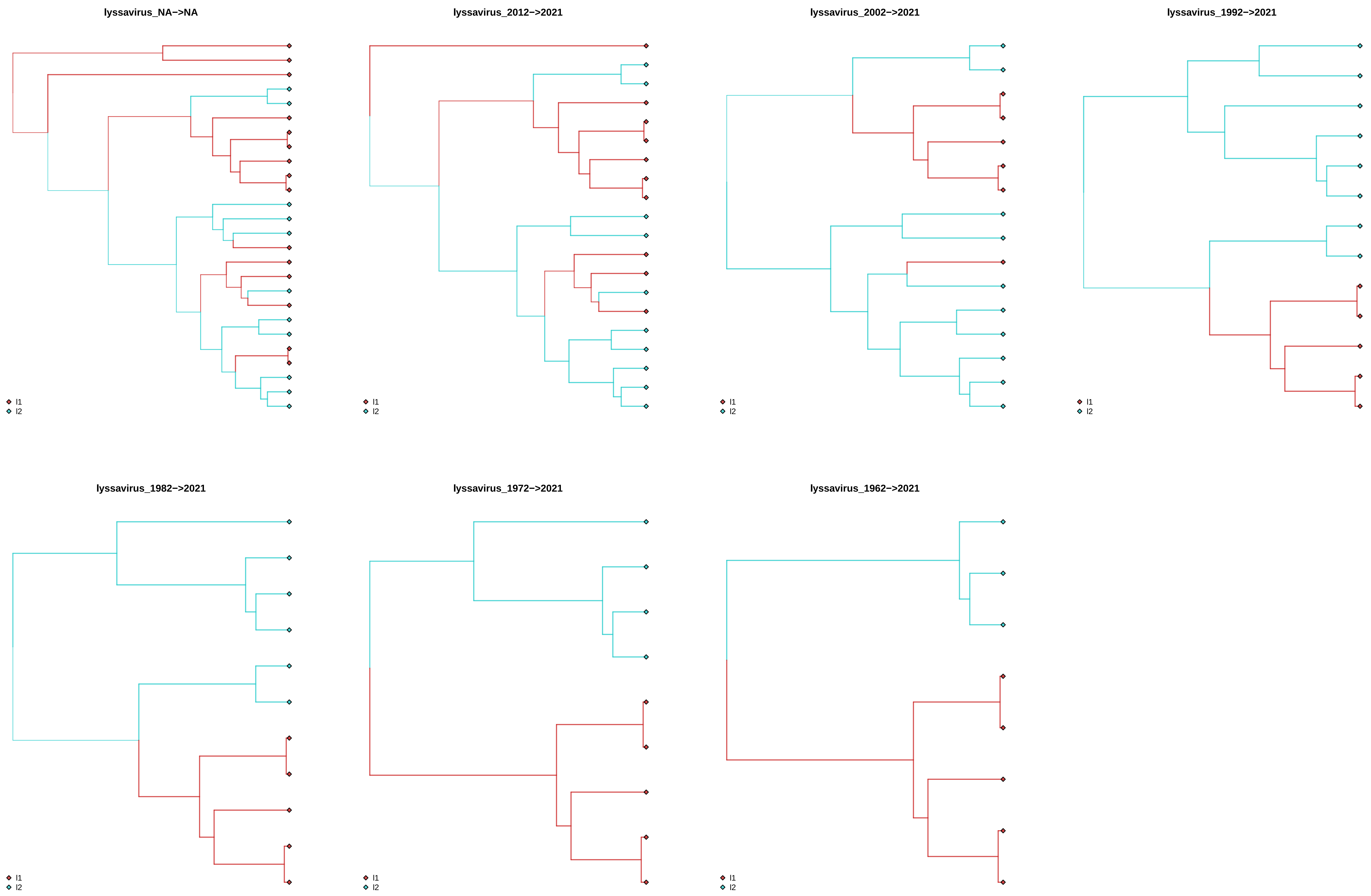
lyssavirus_NA−>NA
l1
l2
lyssavirus_2012−>2021
l1
l2
lyssavirus_2002−>2021
l1
l2
lyssavirus_1992−>2021
l1
l2
lyssavirus_1982−>2021
l1
l2
lyssavirus_1972−>2021
l1
l2
lyssavirus_1962−>2021
l1
l2

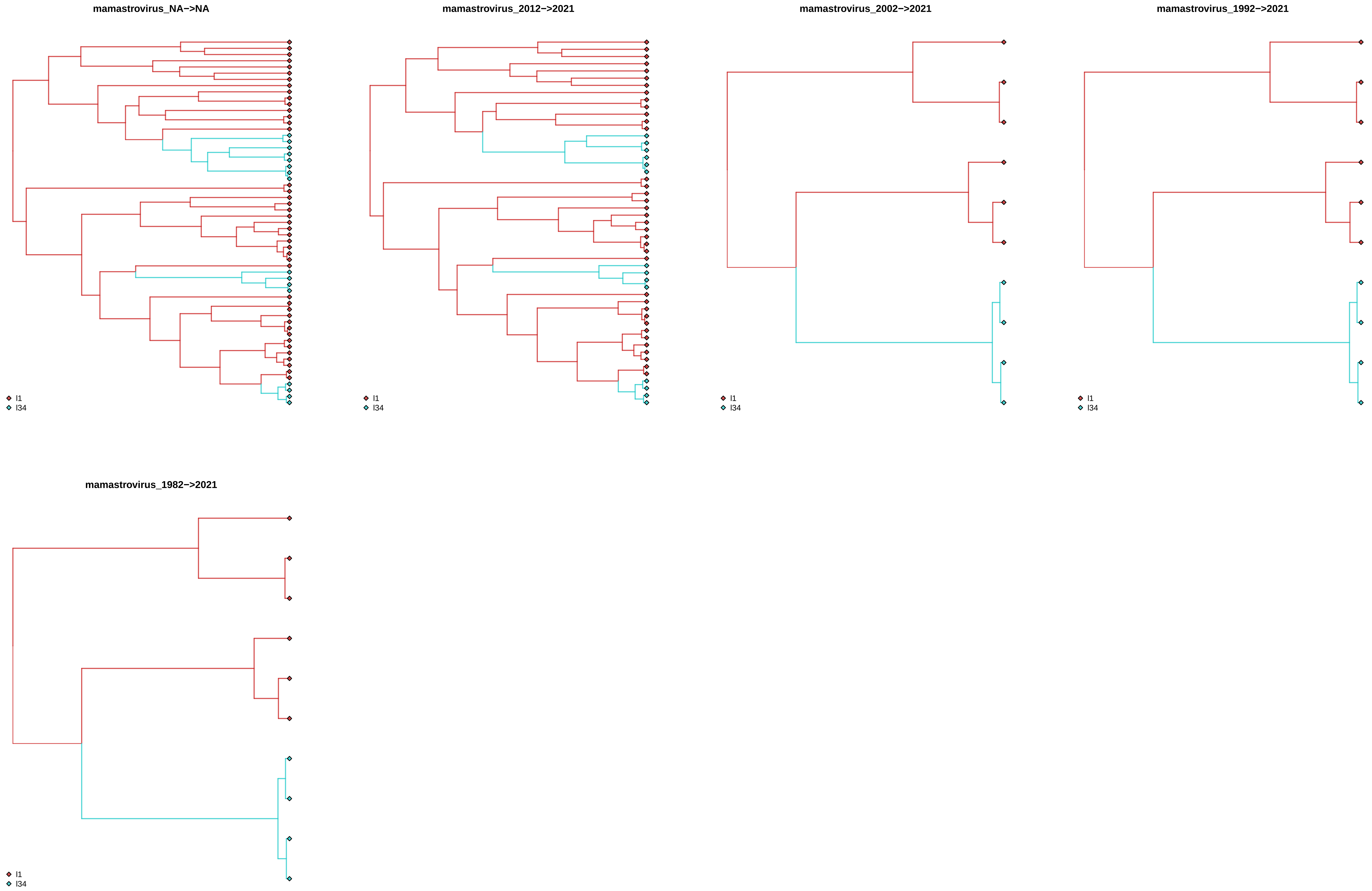
mamastrovirus_NA−>NA
l1
l34
mamastrovirus_2012−>2021
l1
l34
mamastrovirus_2002−>2021
l1
l34
mamastrovirus_1992−>2021
l1
l34
mamastrovirus_1982−>2021
l1
l34

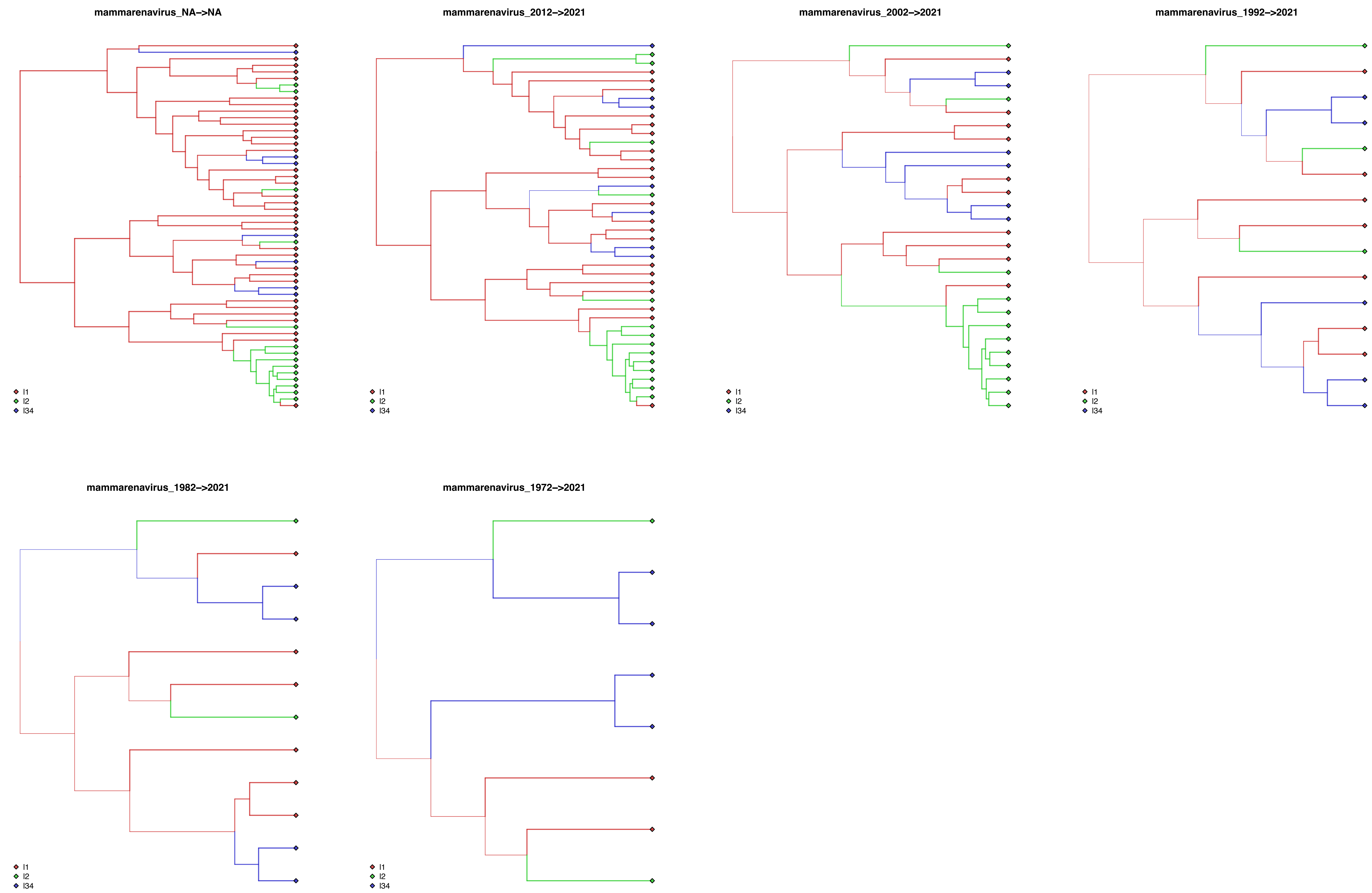
mammarenavirus_NA–>NA
l1
l2
l34
mammarenavirus_2012–>2021
l1
l2
l34
mammarenavirus_2002–>2021
l1
l2
l34
mammarenavirus_1992–>2021
l1
l2
l34
mammarenavirus_1982–>2021
l1
l2
l34
mammarenavirus_1972–>2021
l1
l2
l34

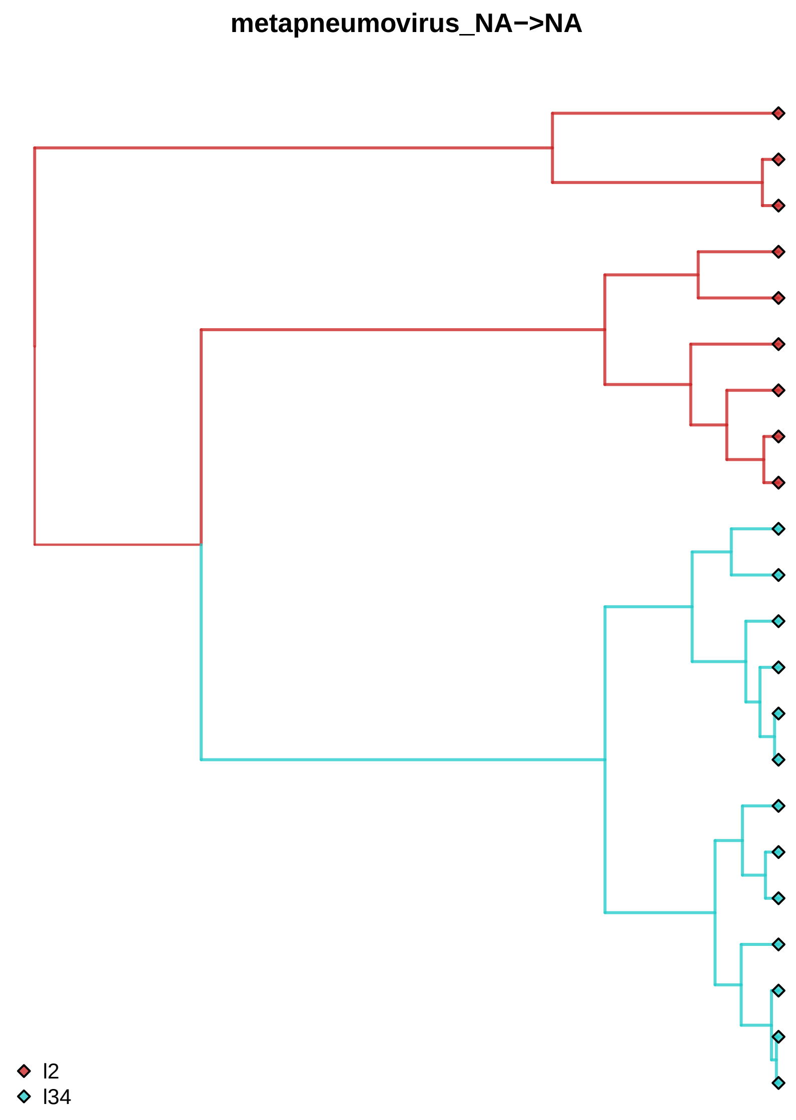
metapneumovirus_NA−>NA
l2
l34

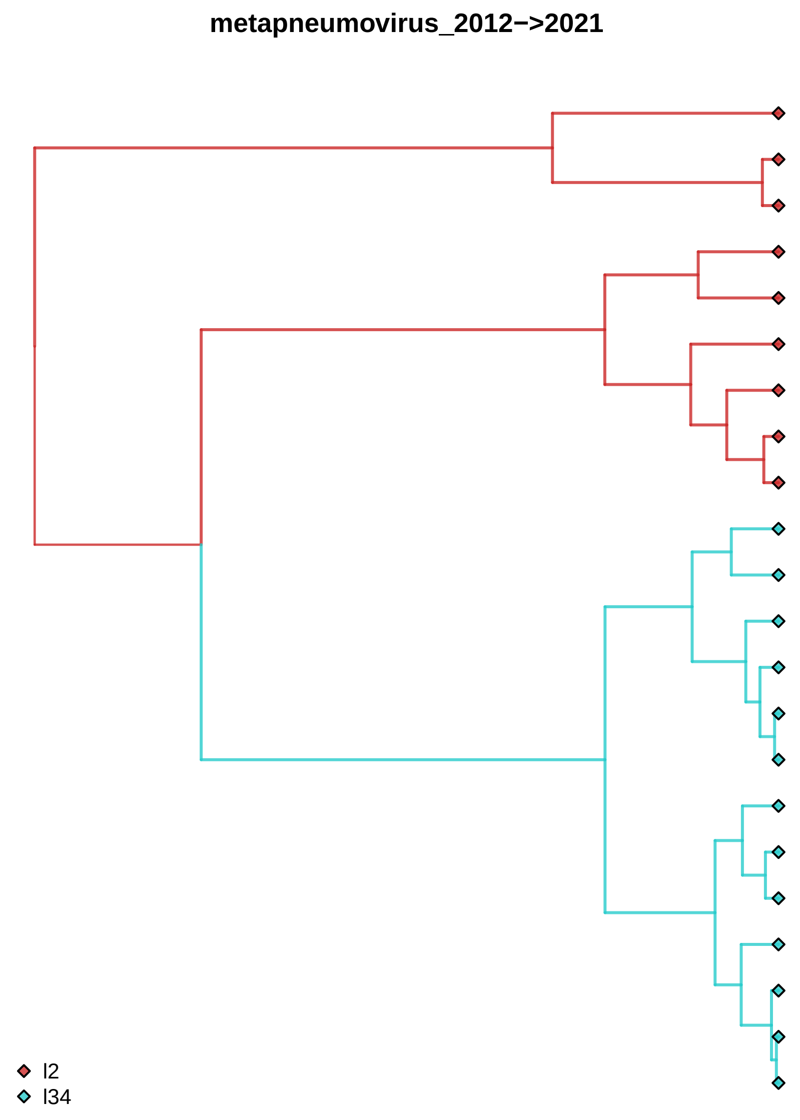
metapneumovirus_2012−>2021
l2
l34

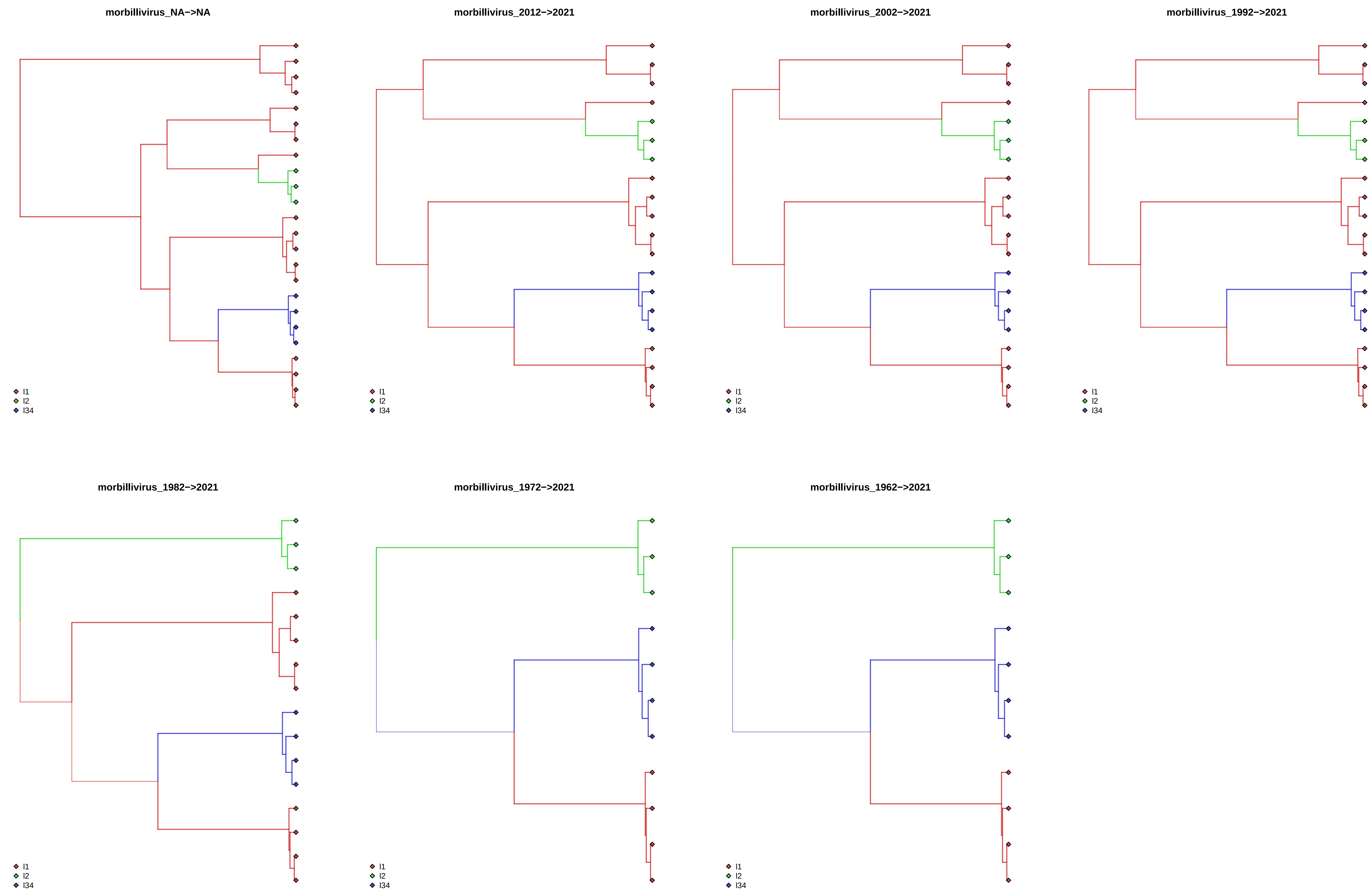
morbillivirus_NA−>NA
morbillivirus_2012−>2021
morbillivirus_2002−>2021
morbillivirus_1992−>2021
morbillivirus_1982−>2021
morbillivirus_1972−>2021
morbillivirus_1962−>2021
l1
l2
l34

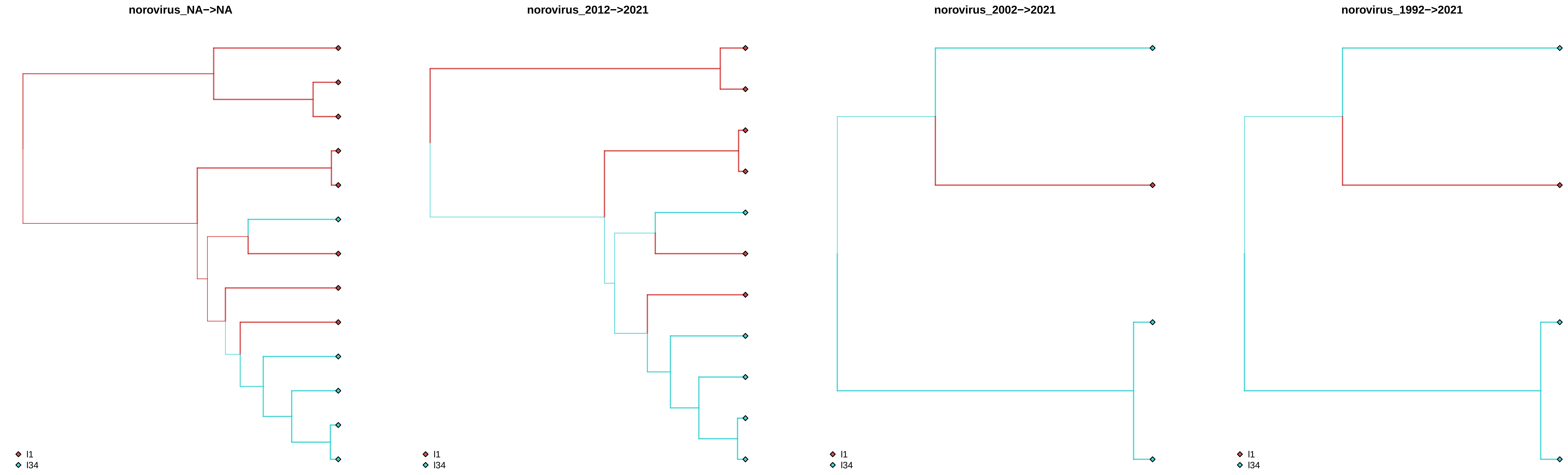
norovirus_NA−>NA
norovirus_2012−>2021
norovirus_2002−>2021
norovirus_1992−>2021
I1
I34

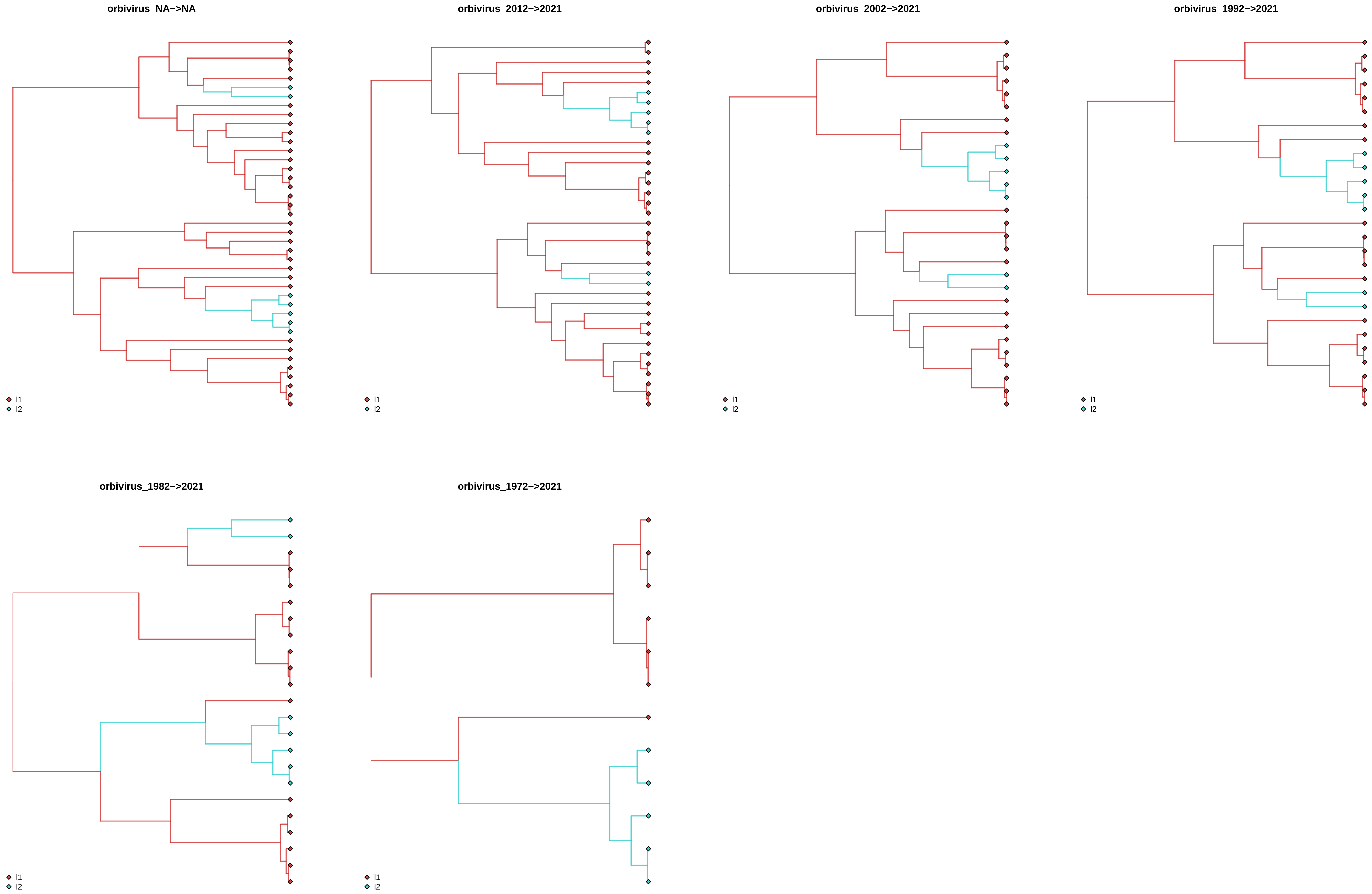
orbivirus_NA–>NA
orbivirus_2012–>2021
orbivirus_2002–>2021
orbivirus_1992–>2021
orbivirus_1982–>2021
orbivirus_1972–>2021
l1
l2

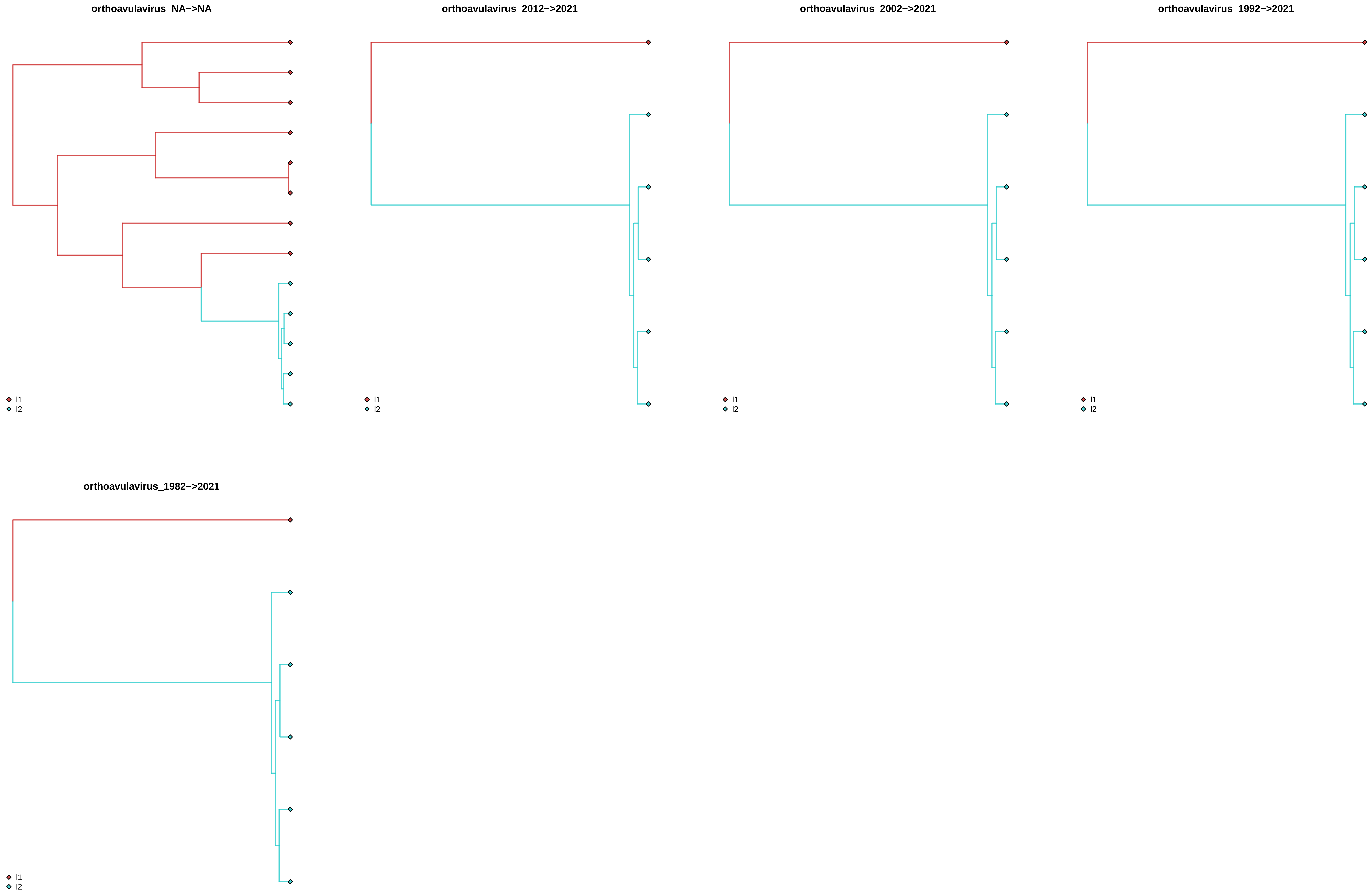
orthoavulavirus_NA−>NA
orthoavulavirus_2012−>2021
orthoavulavirus_2002−>2021
orthoavulavirus_1992−>2021
orthoavulavirus_1982−>2021
l1
l2

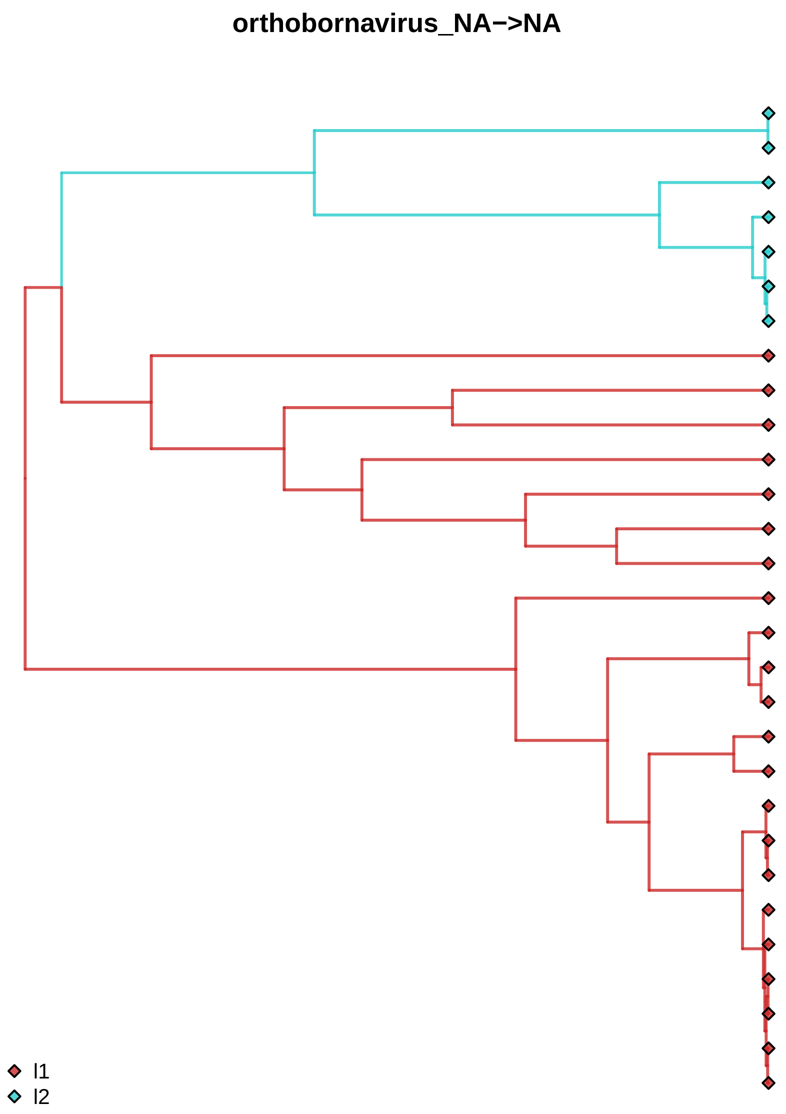
orthobornavirus_NA−>NA
l1
l2

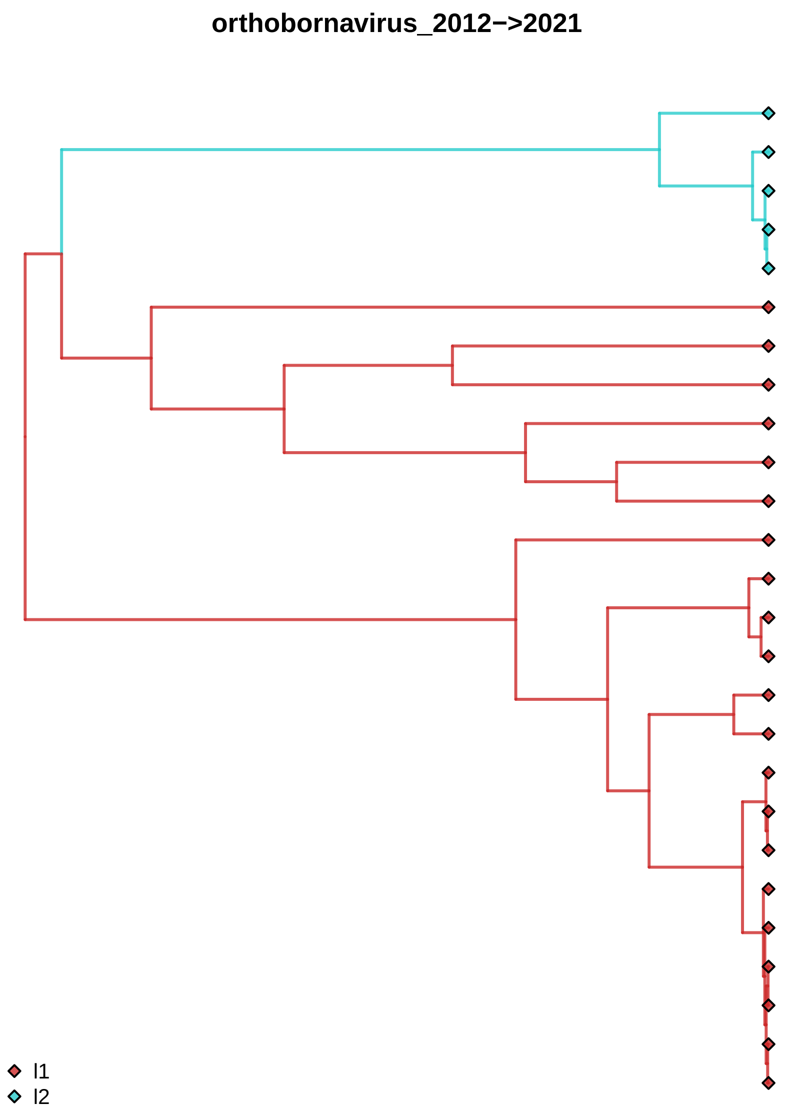
orthobornavirus_2012−>2021
l1
l2

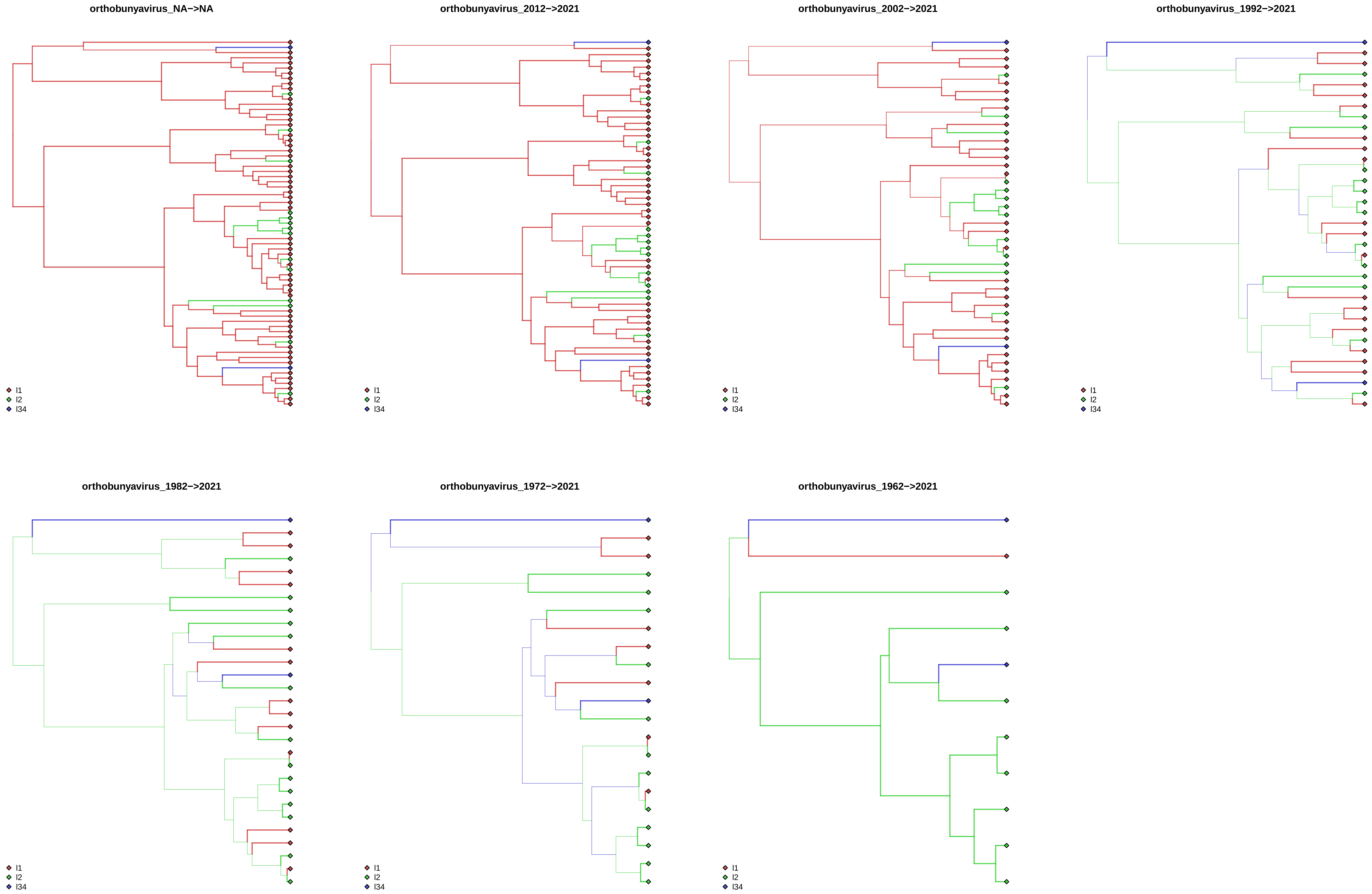
orthobunyavirus_NA−>NA
orthobunyavirus_2012−>2021
orthobunyavirus_2002−>2021
orthobunyavirus_1992−>2021
orthobunyavirus_1982−>2021
orthobunyavirus_1972−>2021
orthobunyavirus_1962−>2021
I1
I2
I34

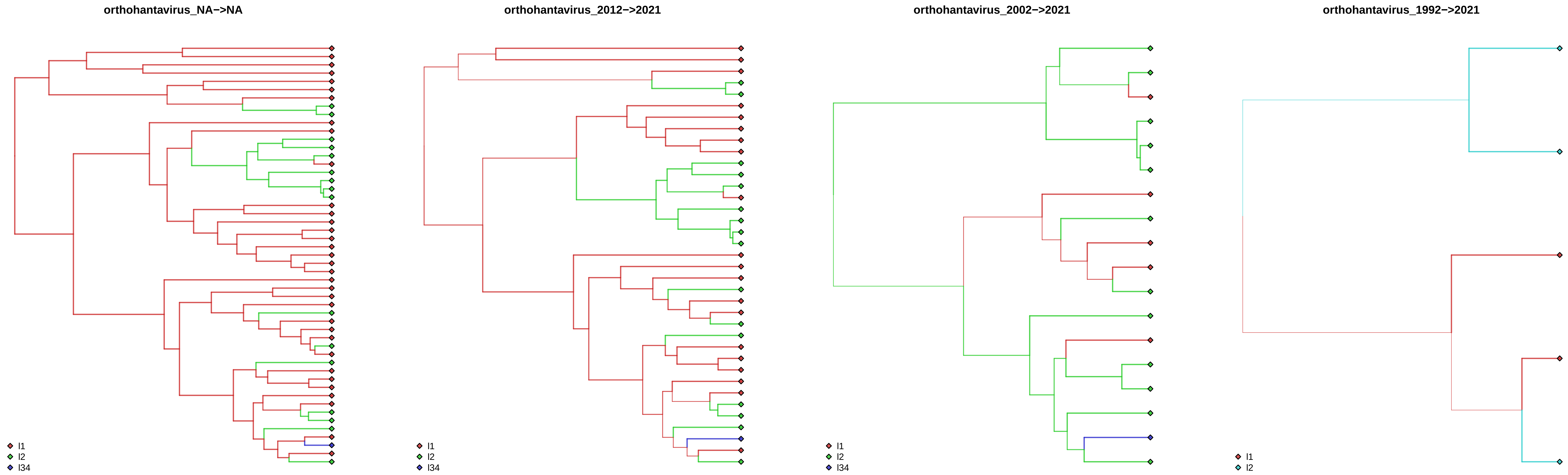
orthohantavirus_NA−>NA
l1
l2
l34
orthohantavirus_2012−>2021
l1
l2
l34
orthohantavirus_2002−>2021
l1
l2
l34
orthohantavirus_1992−>2021
l1
l2

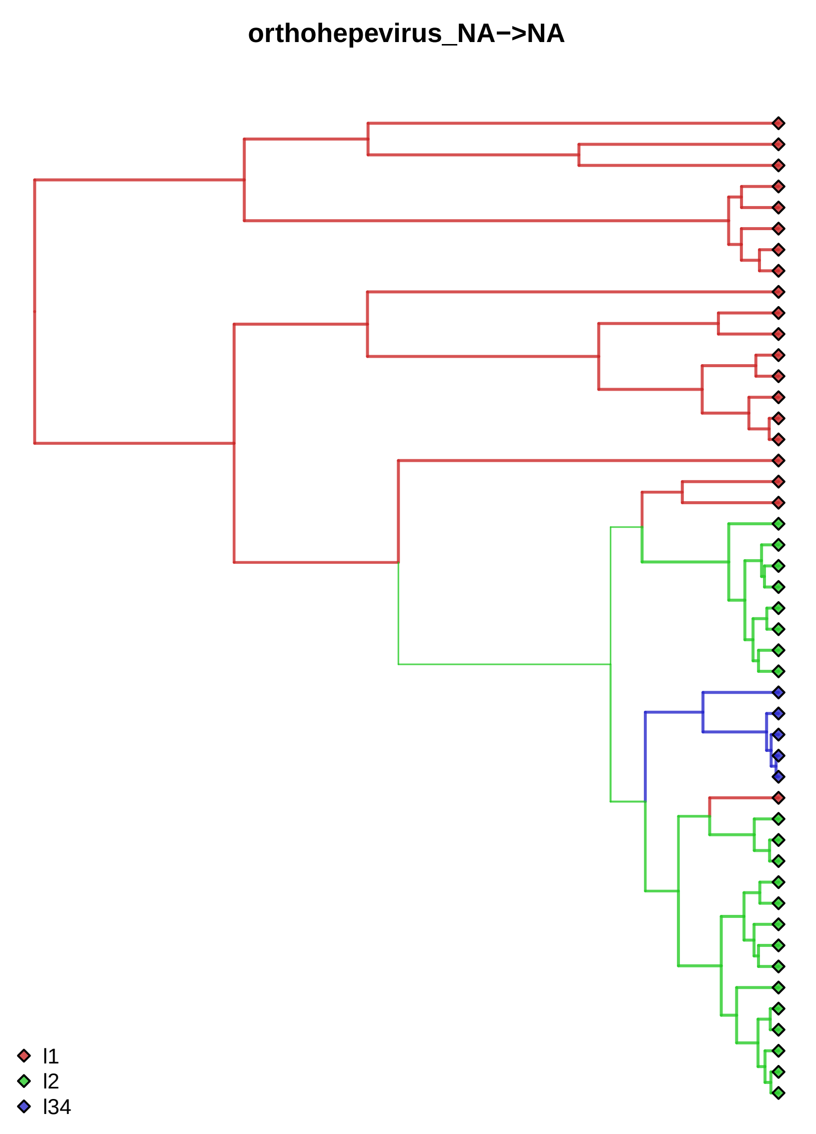
orthohepevirus_NA−>NA
l1
l2
l34

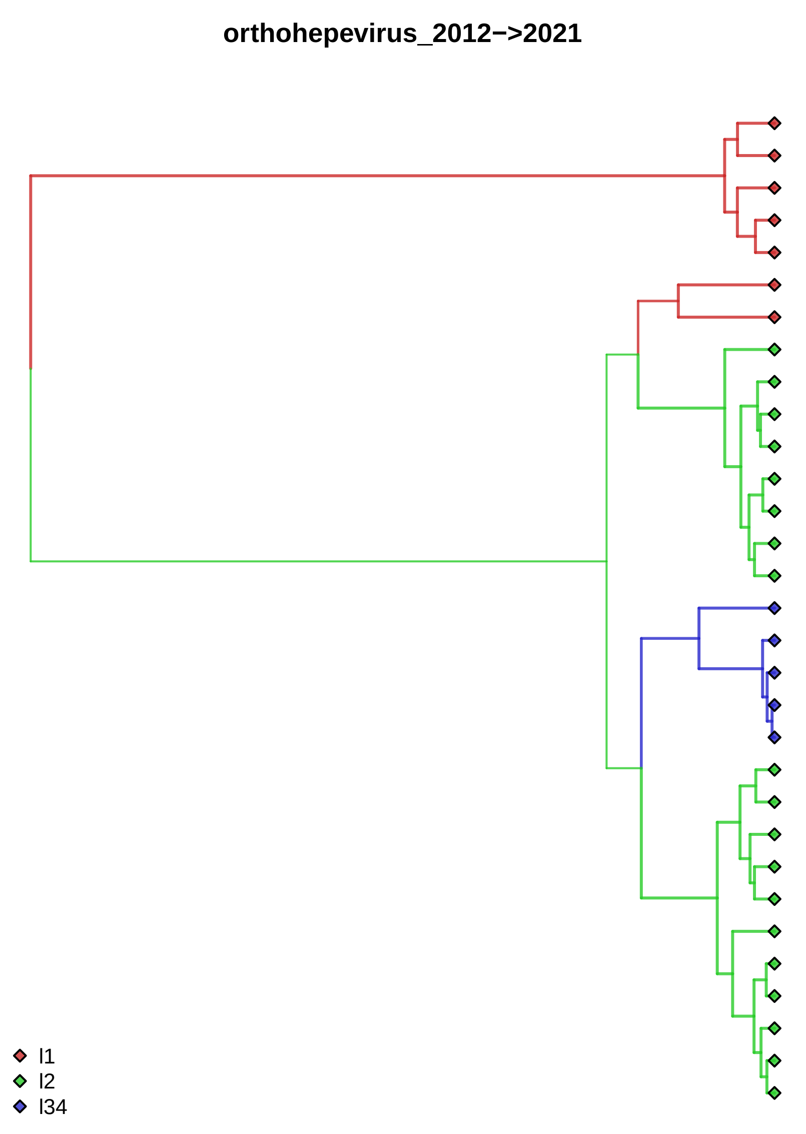
orthohepevirus_2012−>2021
l1
l2
l34

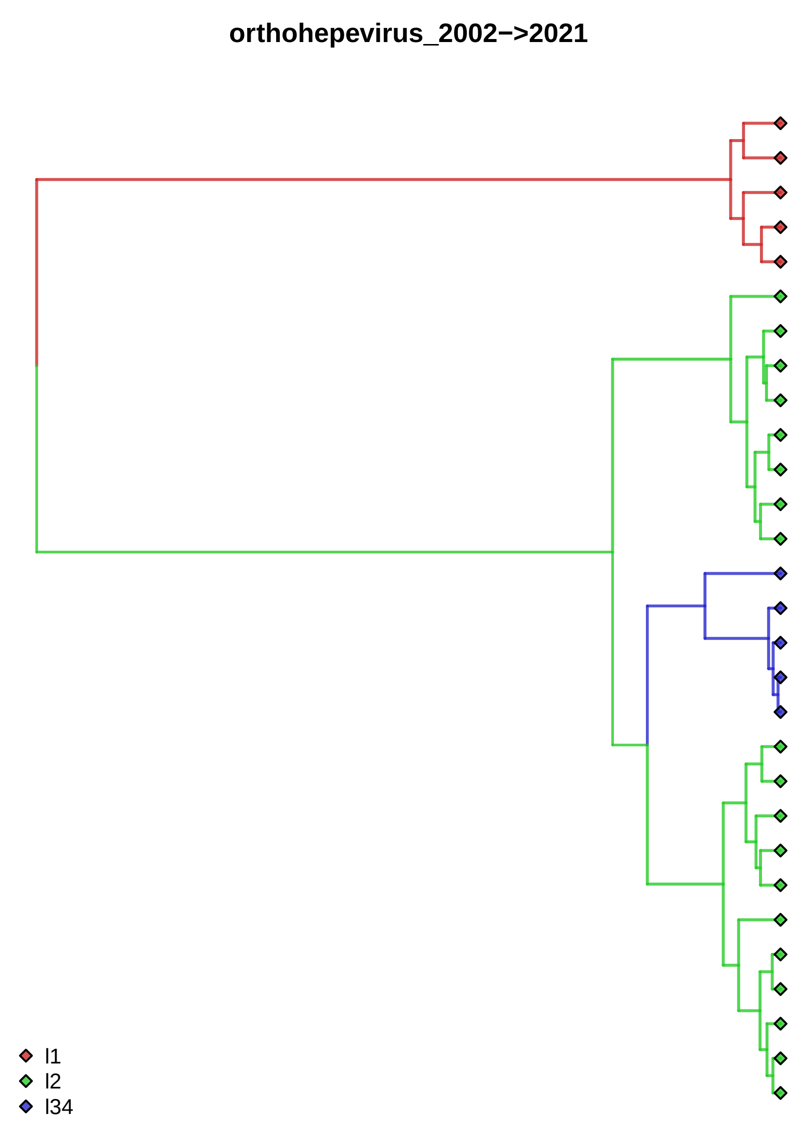
orthohepevirus_2002−>2021
l1
l2
l34

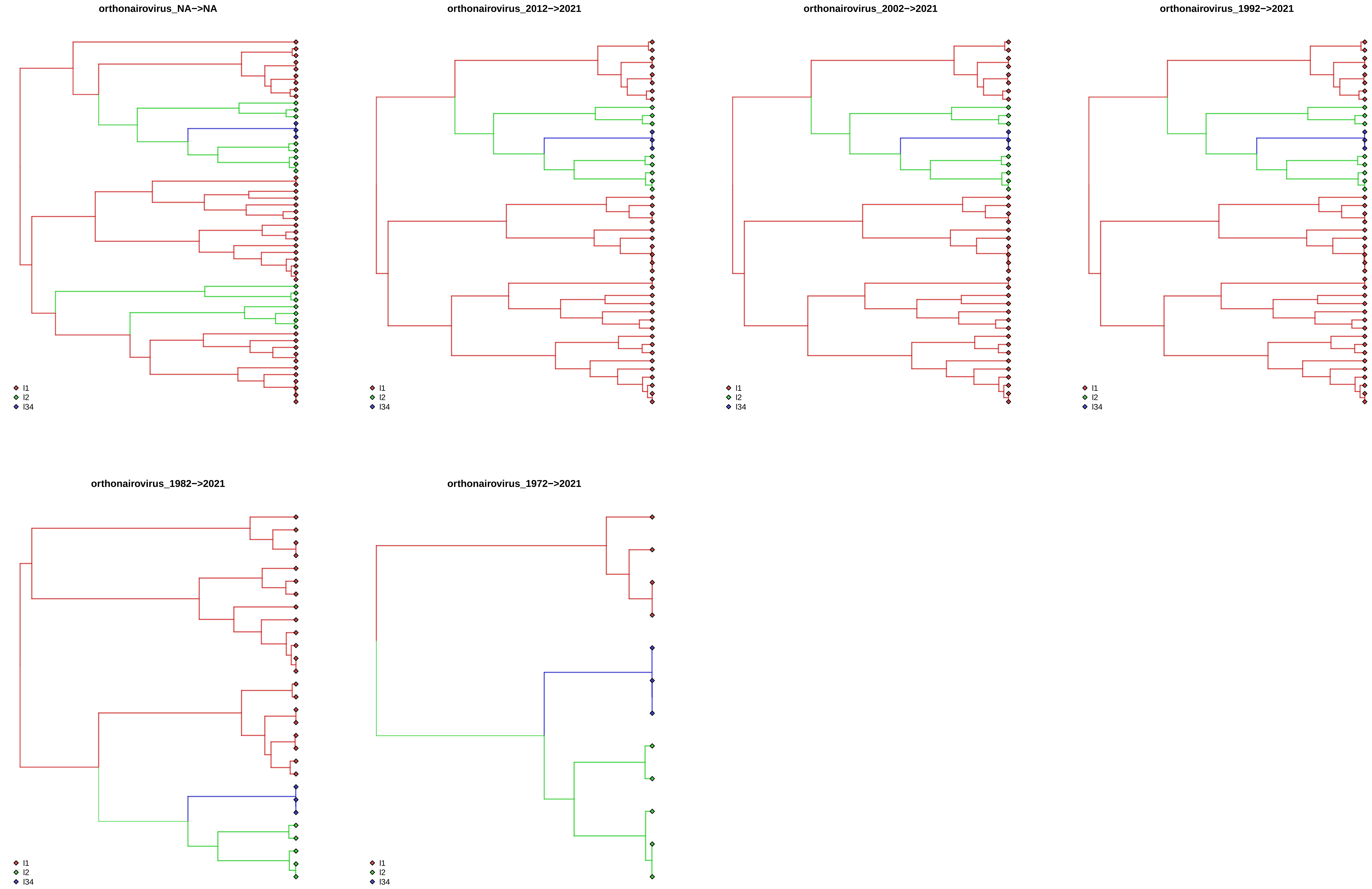
orthonairovirus_NA−>NA
l1
l2
l34
orthonairovirus_2012−>2021
l1
l2
l34
orthonairovirus_2002−>2021
l1
l2
l34
orthonairovirus_1992−>2021
l1
l2
l34
orthonairovirus_1982−>2021
l1
l2
l34
orthonairovirus_1972−>2021
l1
l2
l34

orthopneumovirus_NA−>NA

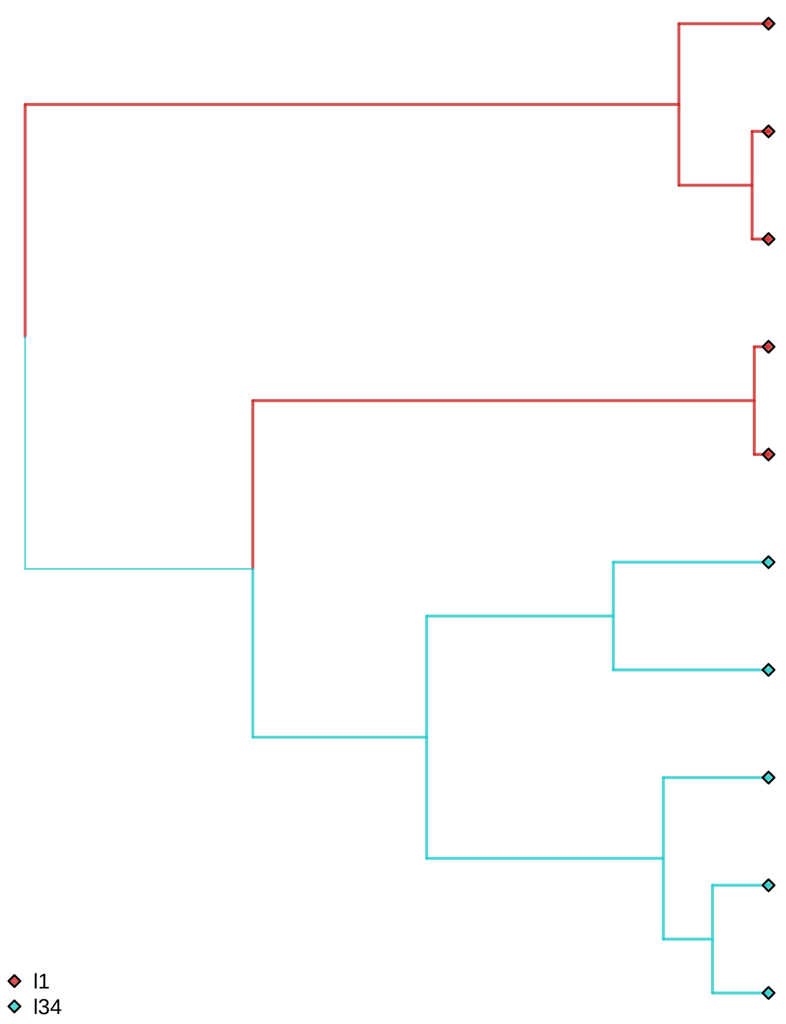

orthopneumovirus_2012−>2021

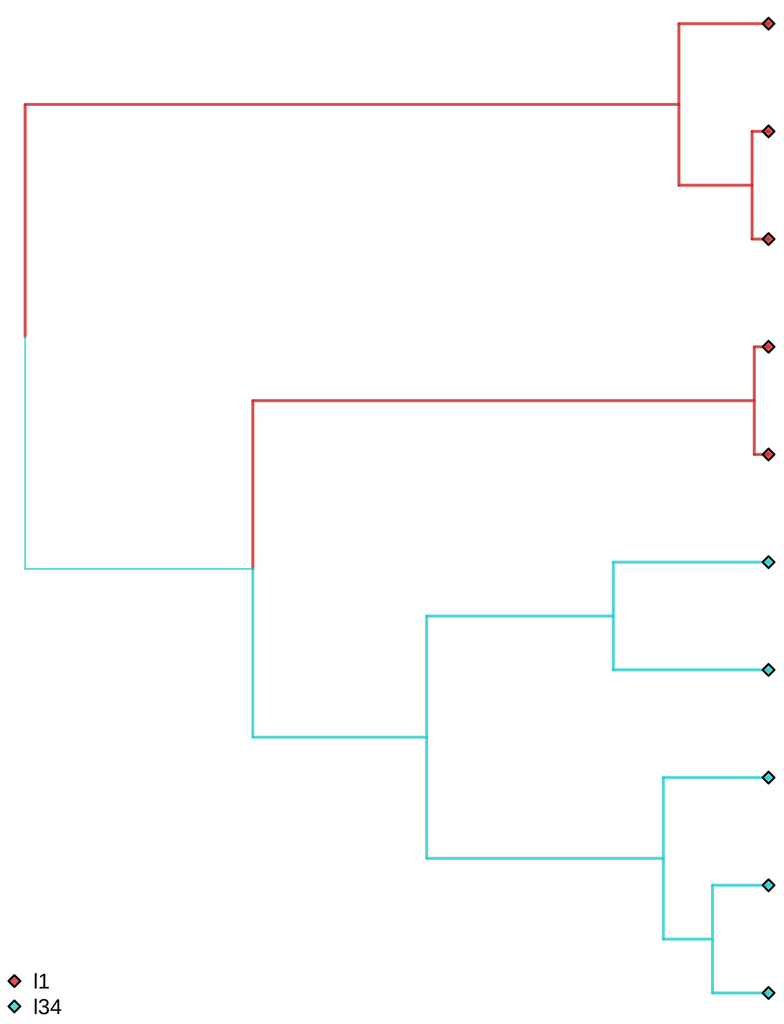

orthopneumovirus_2002−>2021

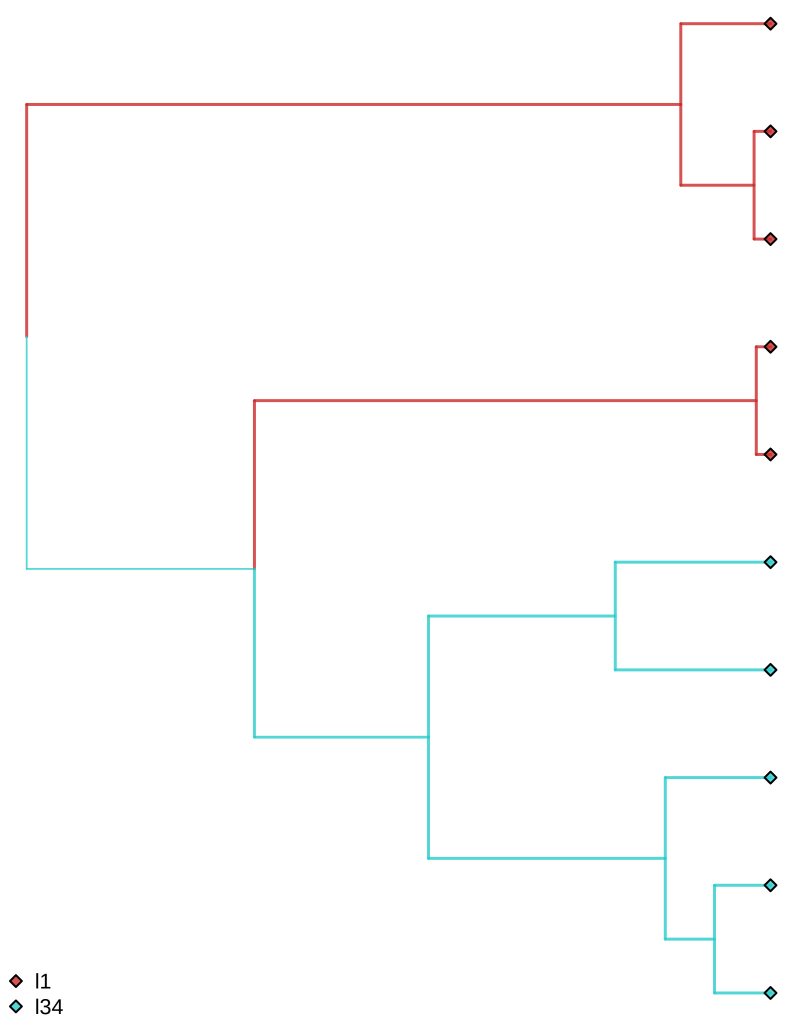

orthopneumovirus_1992−>2021

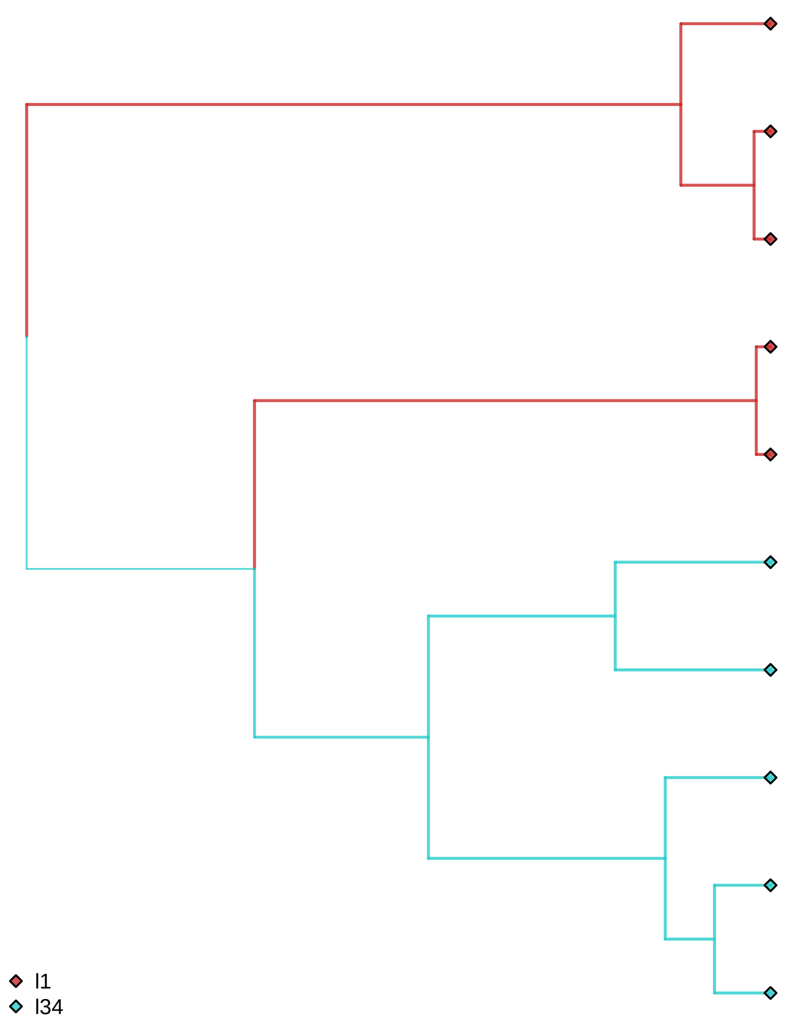

orthopneumovirus_1982−>2021

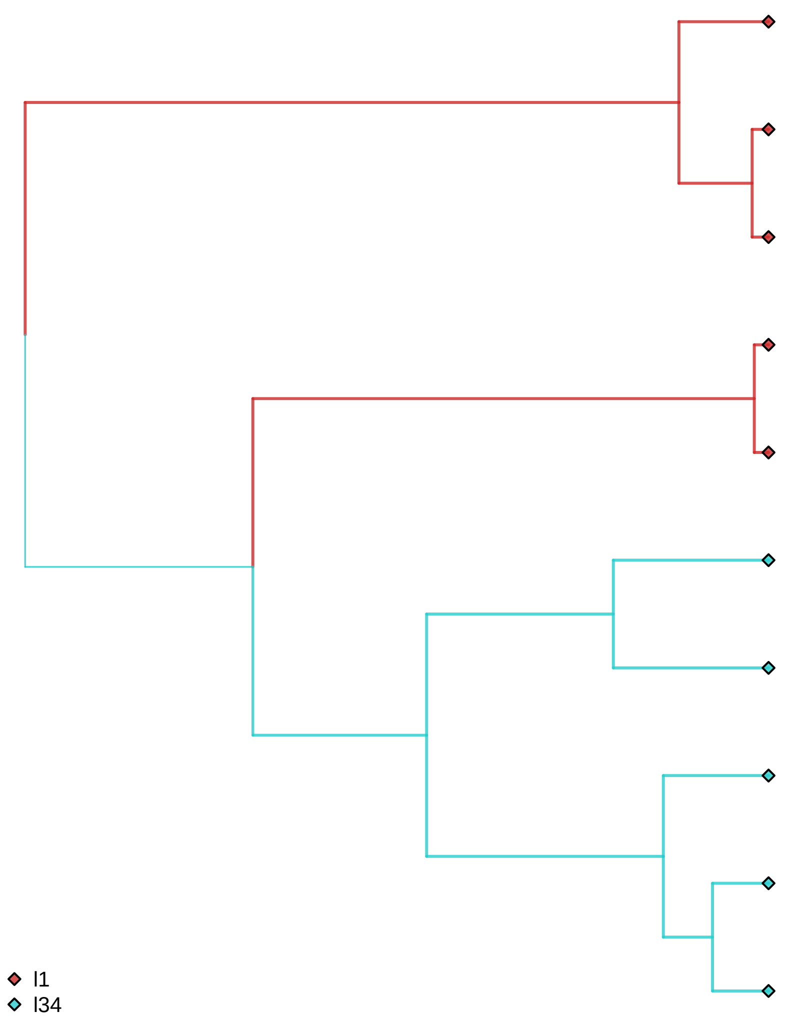

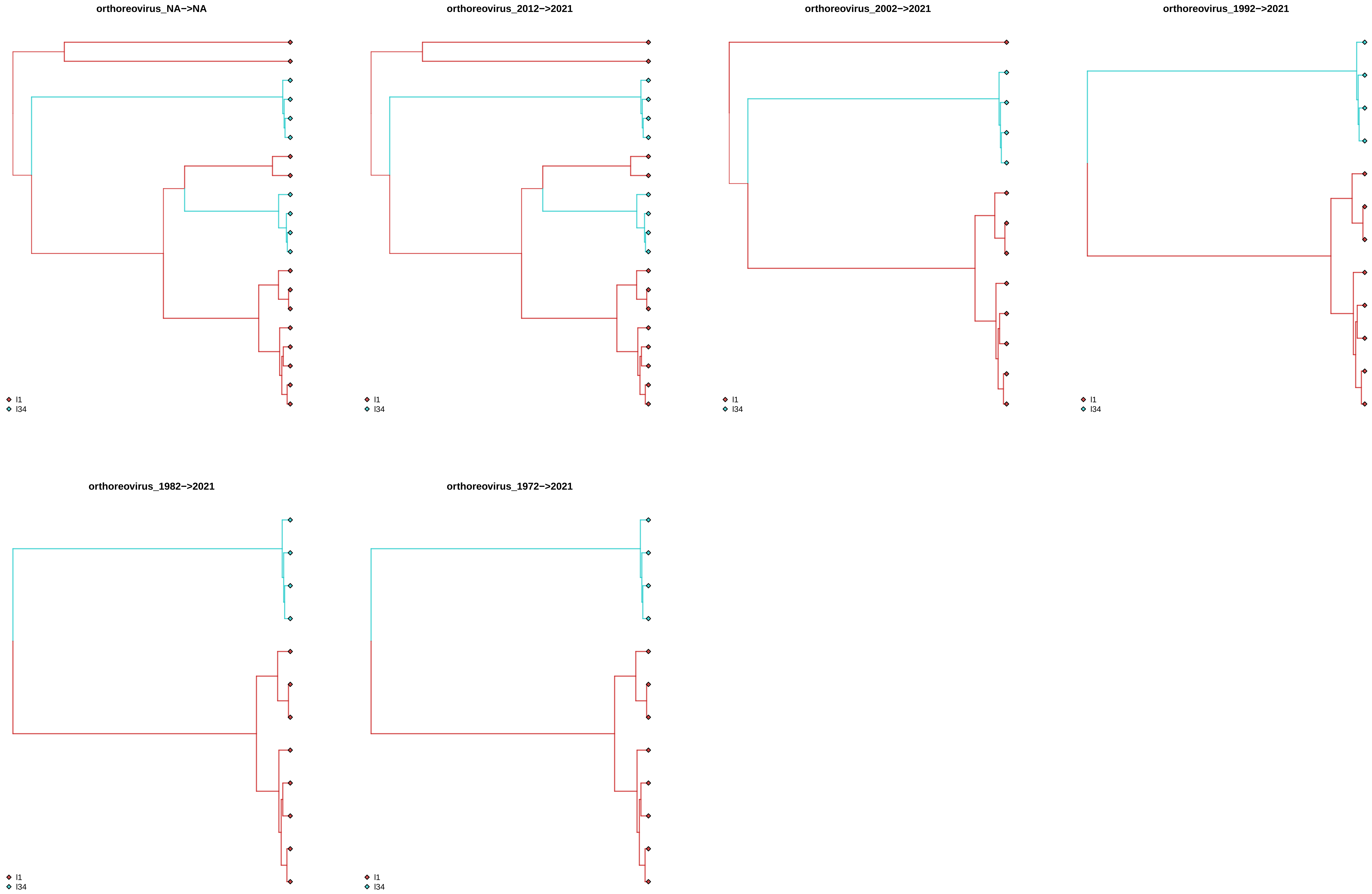

orthoreovirus_NA−>NA
orthoreovirus_2012−>2021
orthoreovirus_2002−>2021
orthoreovirus_1992−>2021
orthoreovirus_1982−>2021
orthoreovirus_1972−>2021
l1
l34

orthorubulavirus_NA−>NA

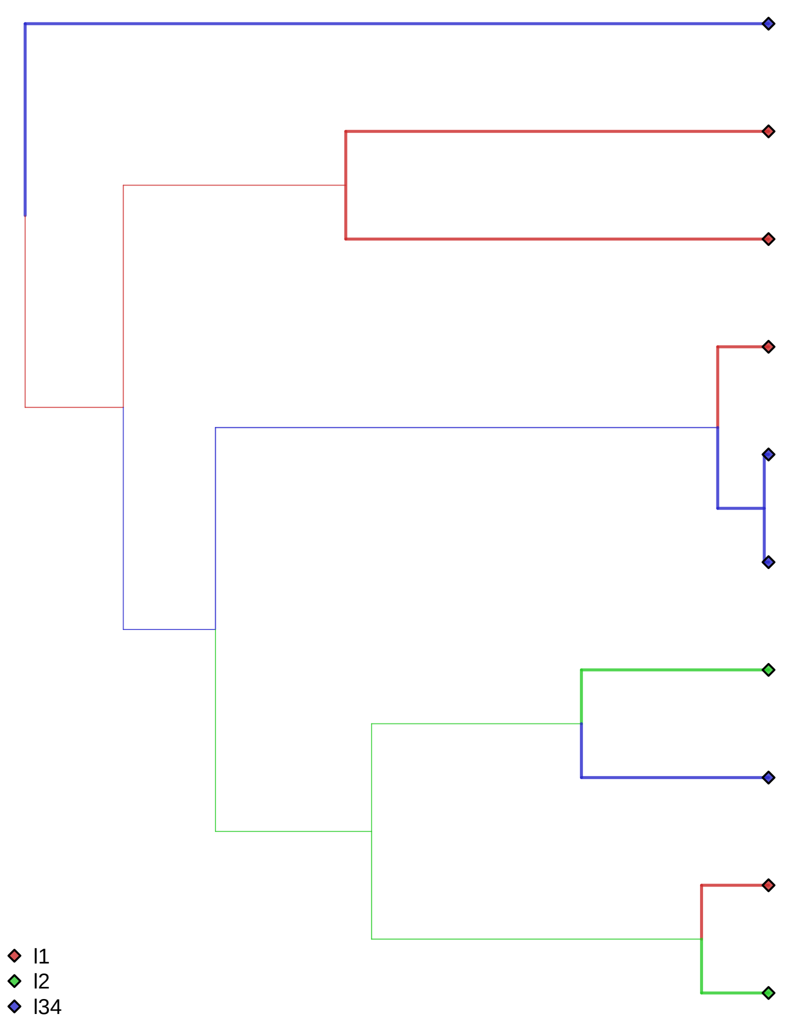

orthorubulavirus_2012−>2021

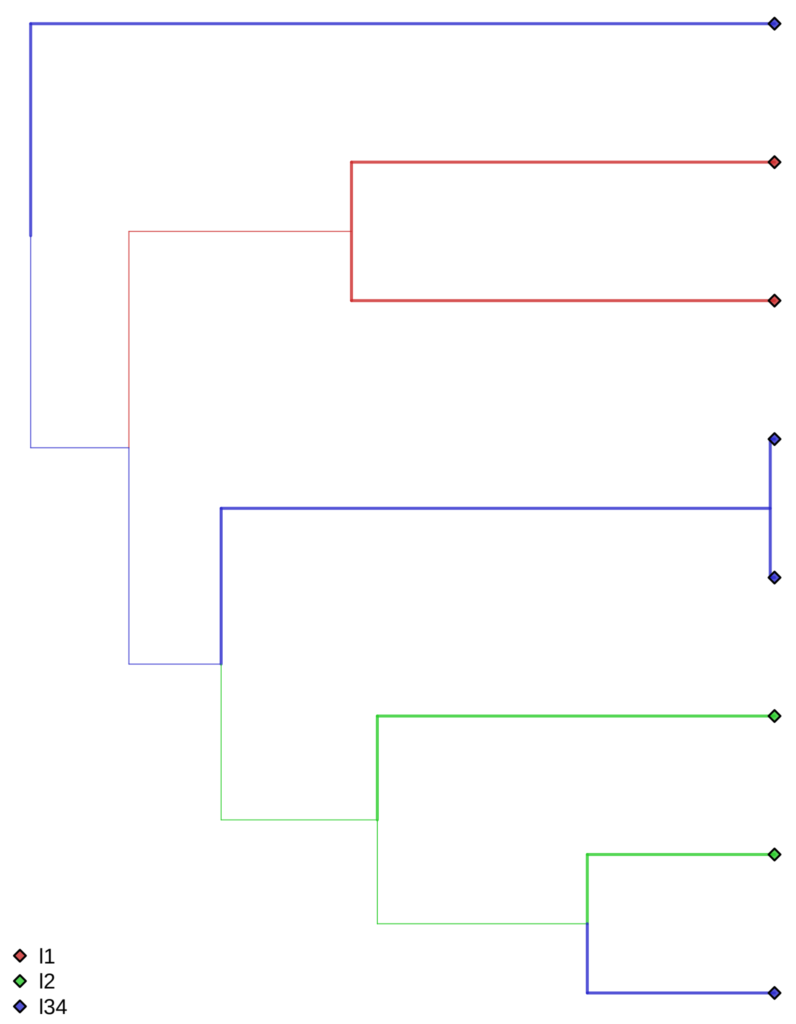

orthorubulavirus_2002−>2021

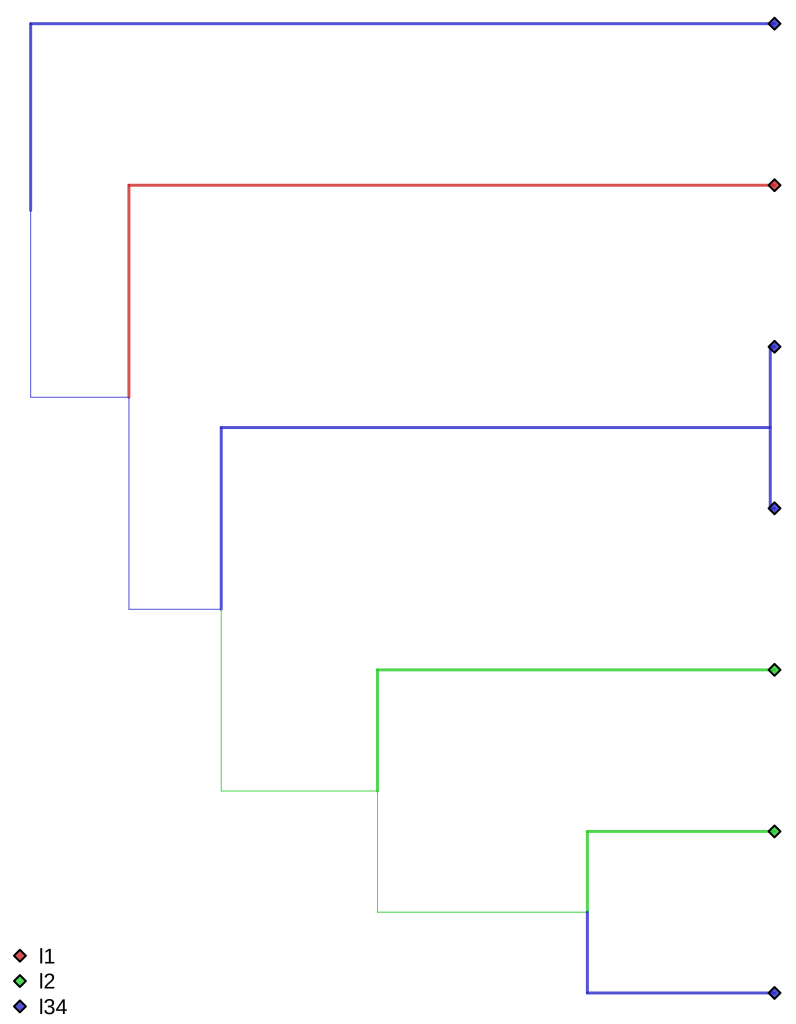

orthorubulavirus_1992−>2021

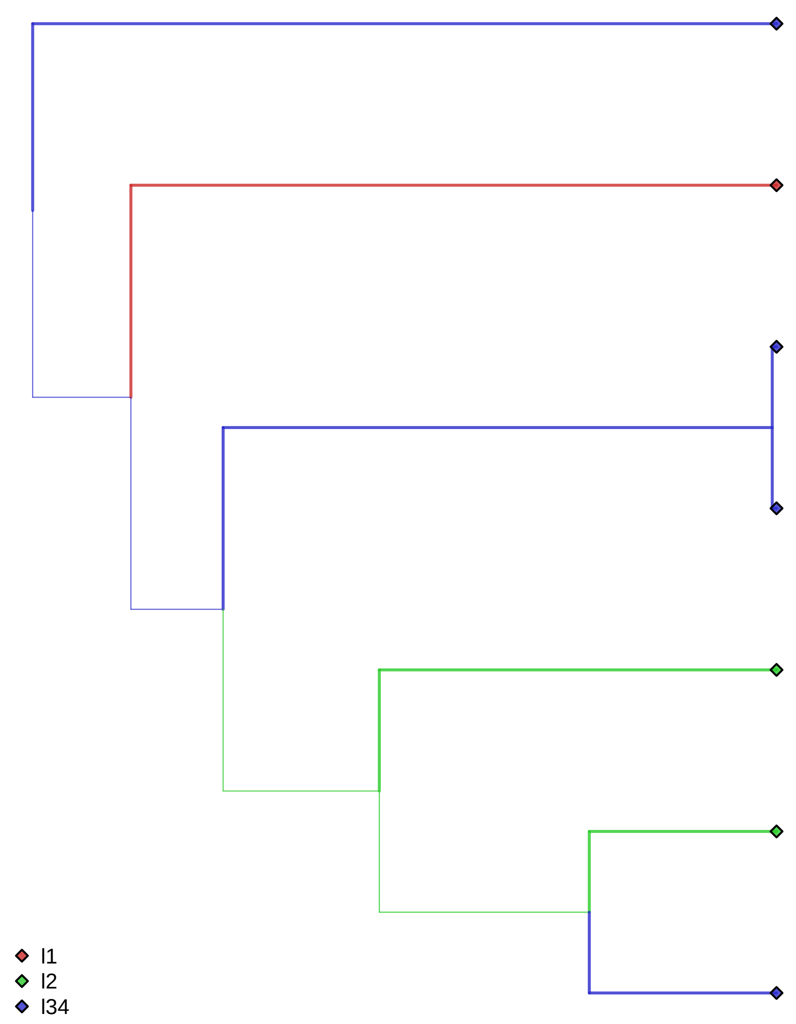

orthorubulavirus_1982−>2021

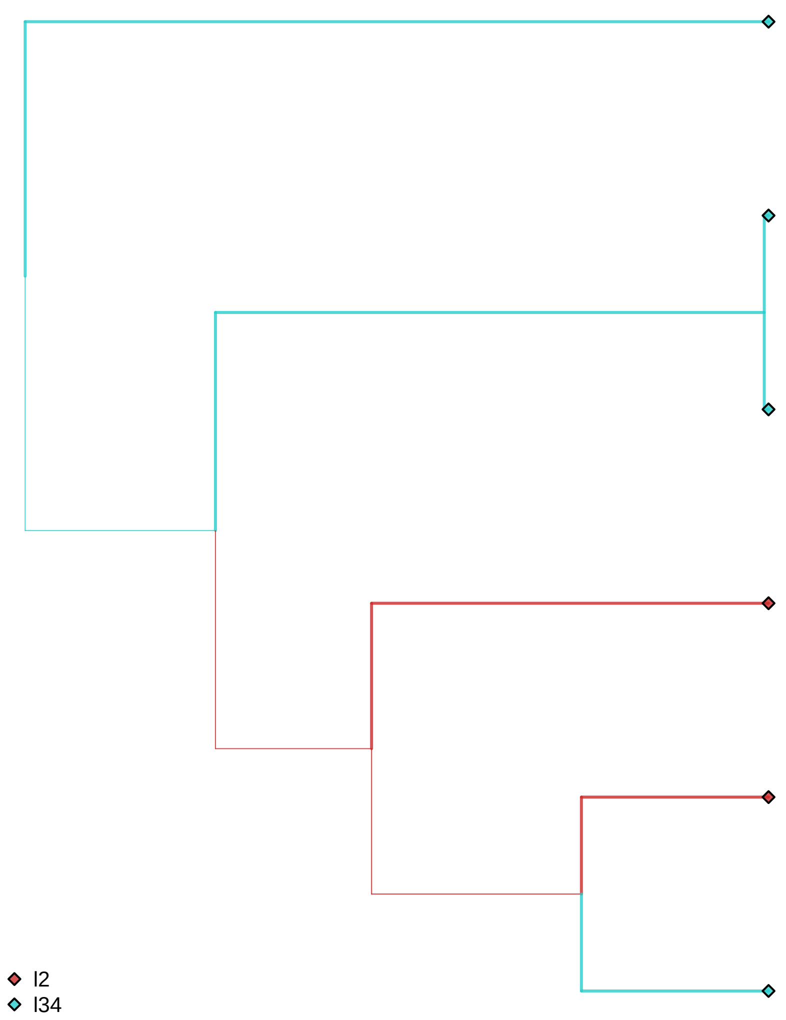

orthorubulavirus_1972−>2021

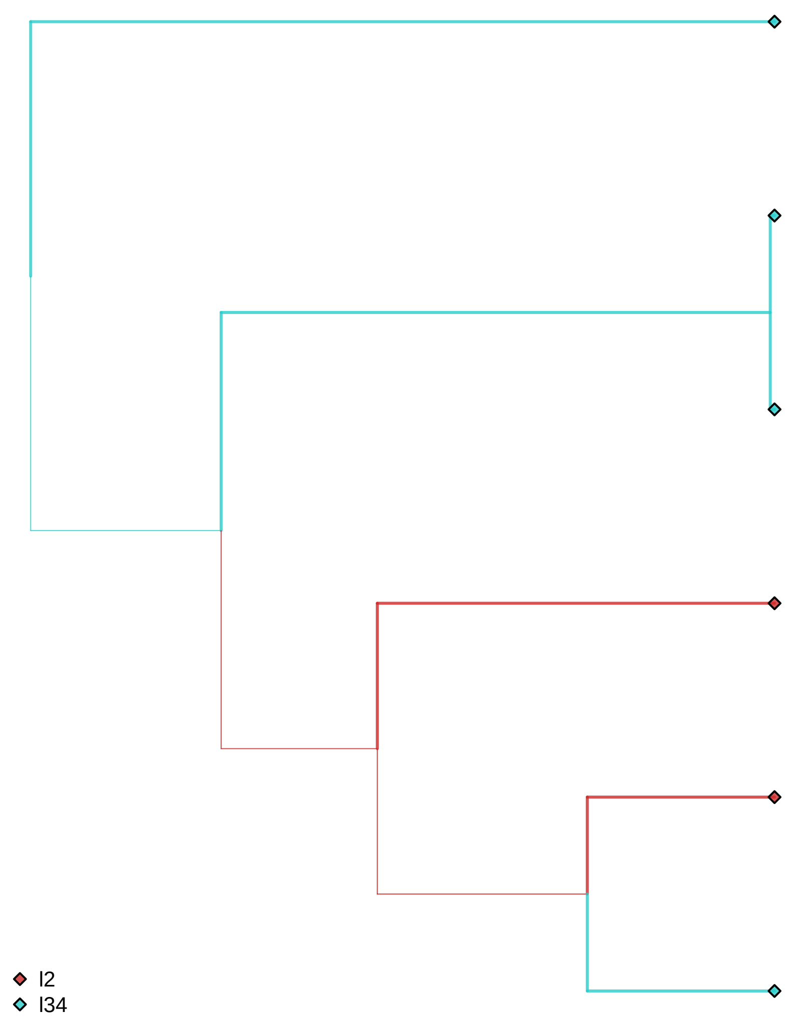

orthorubulavirus_1962−>2021

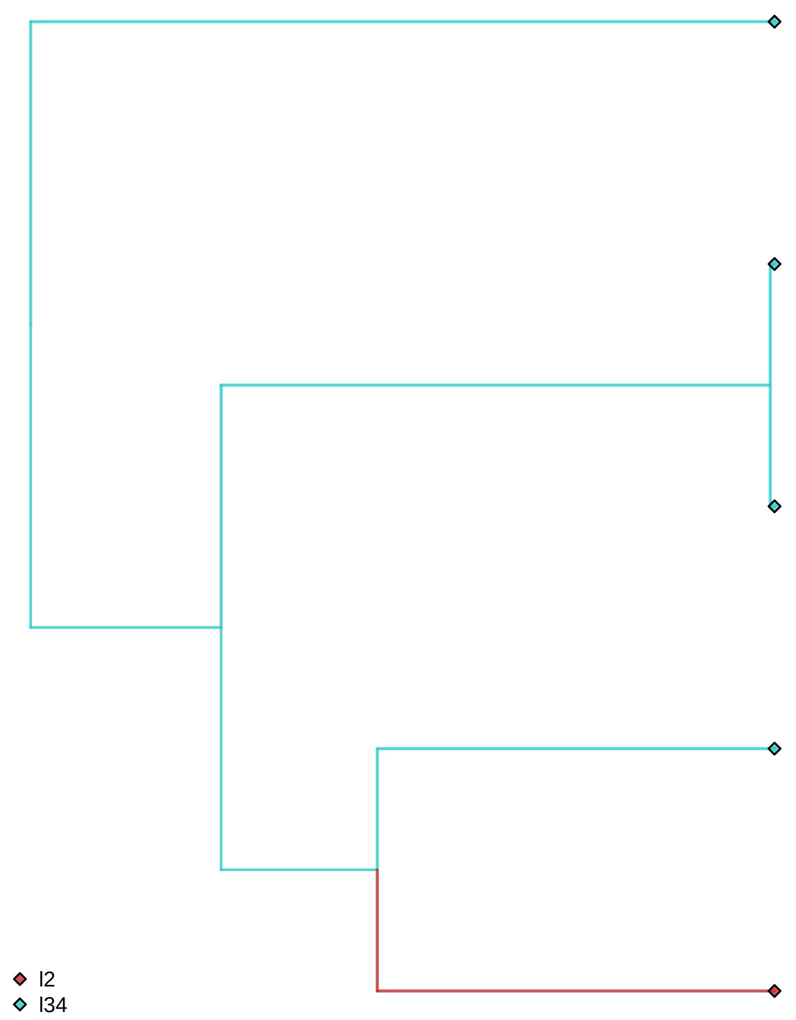

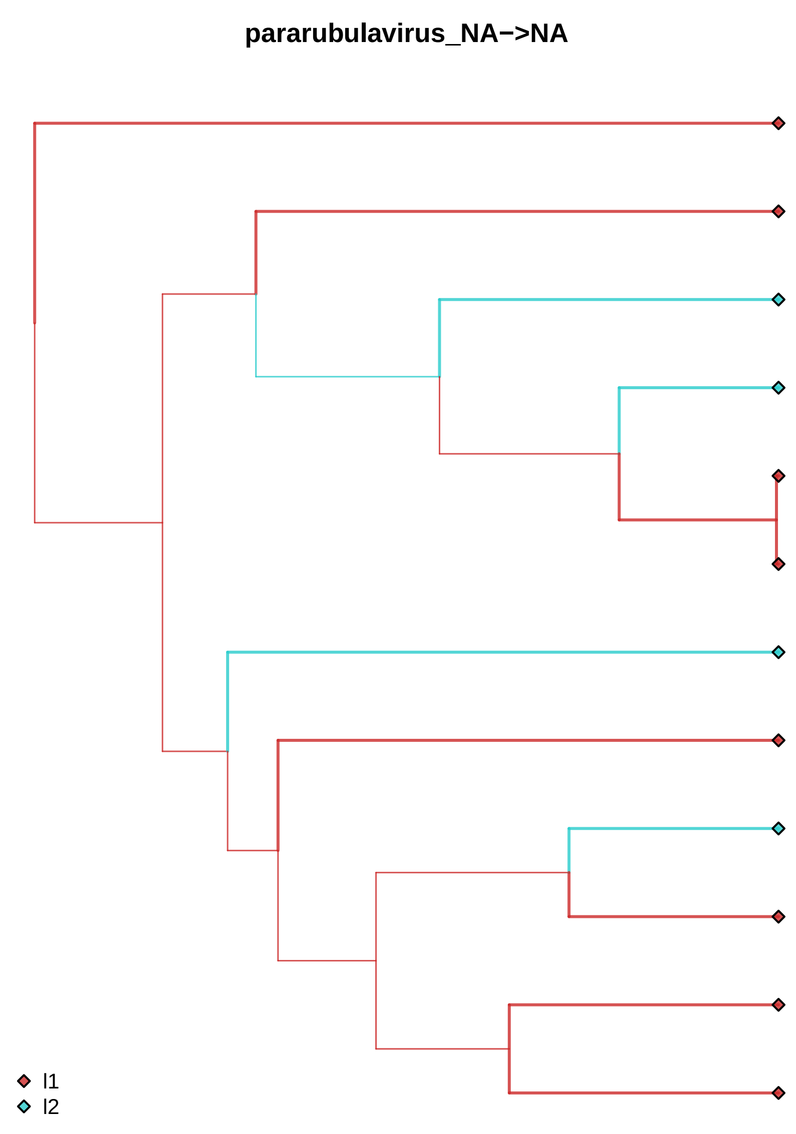
pararubulavirus_NA−>NA
l1
l2

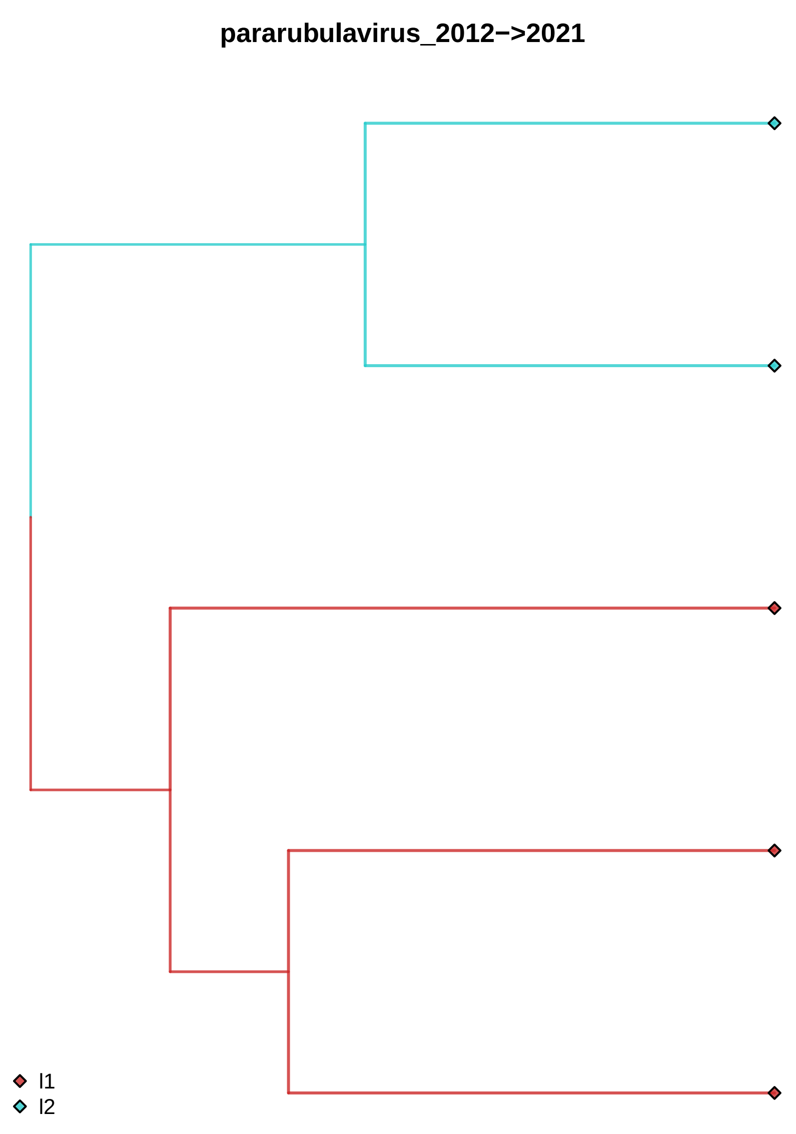
pararubulavirus_2012−>2021
l1
l2

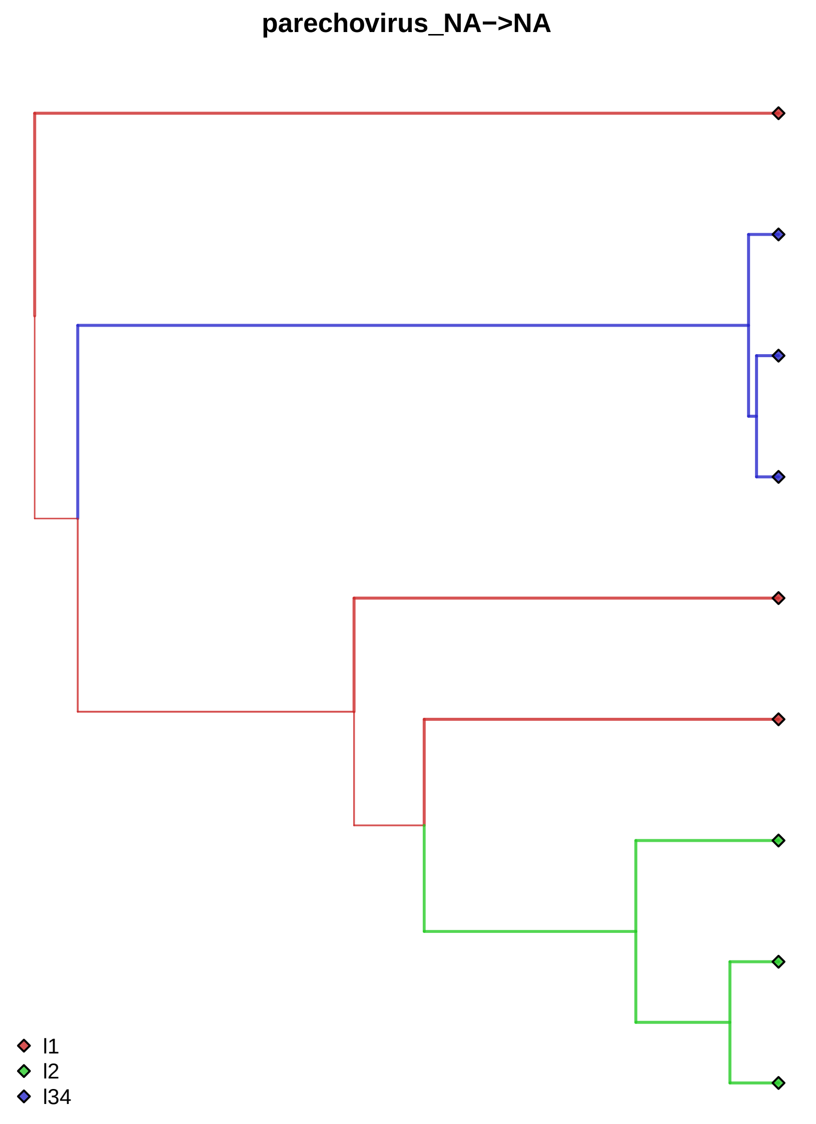

parechovirus_NA−>NA
l1
l2
l34

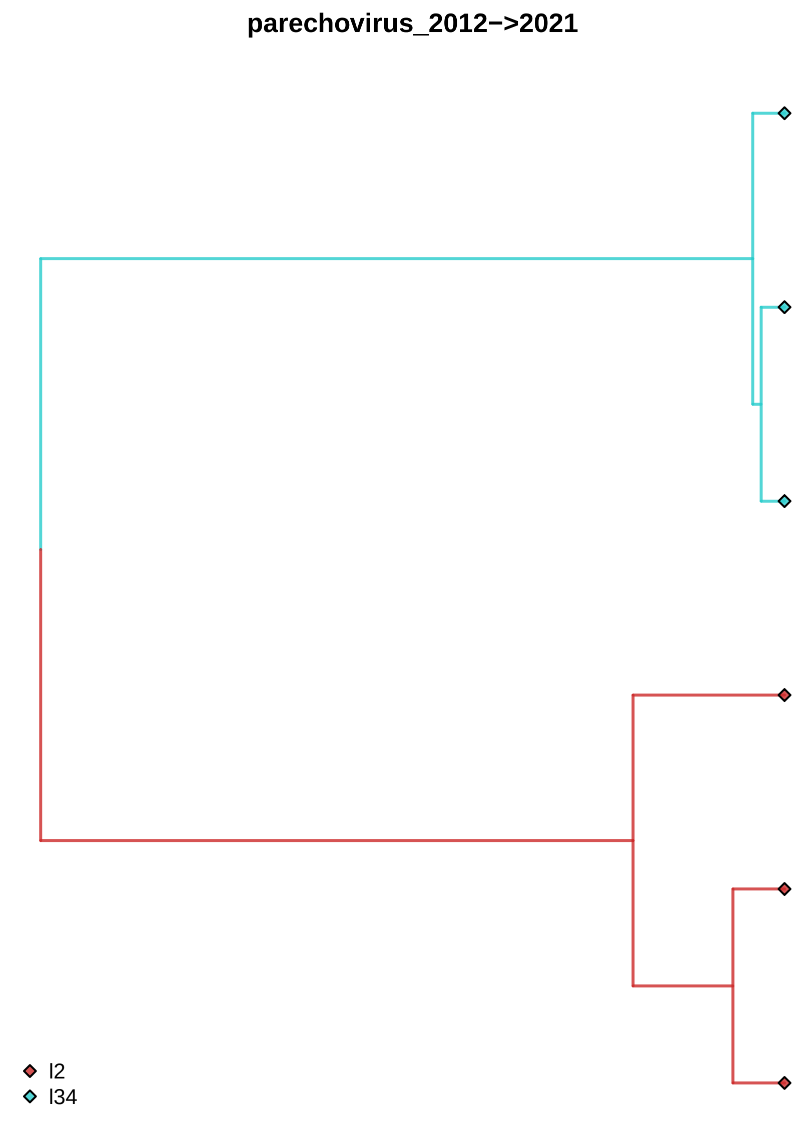

parechovirus_2012−>2021
l2
l34

pegivirus_NA−>NA

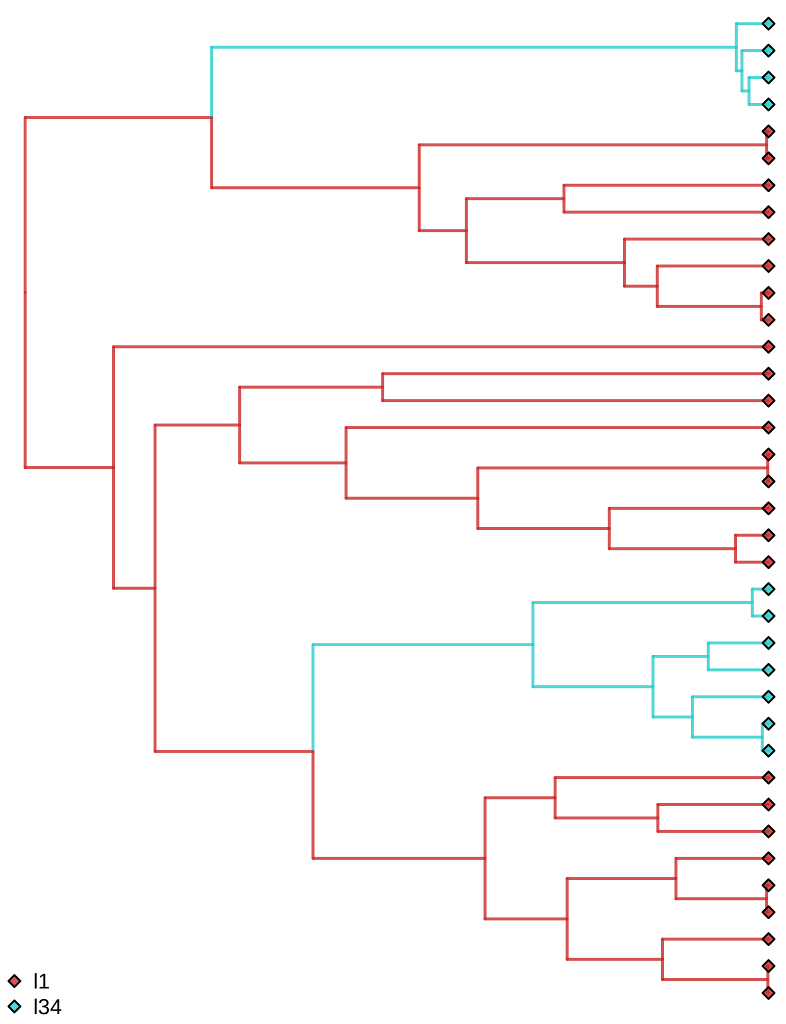

pegivirus_2012−>2021

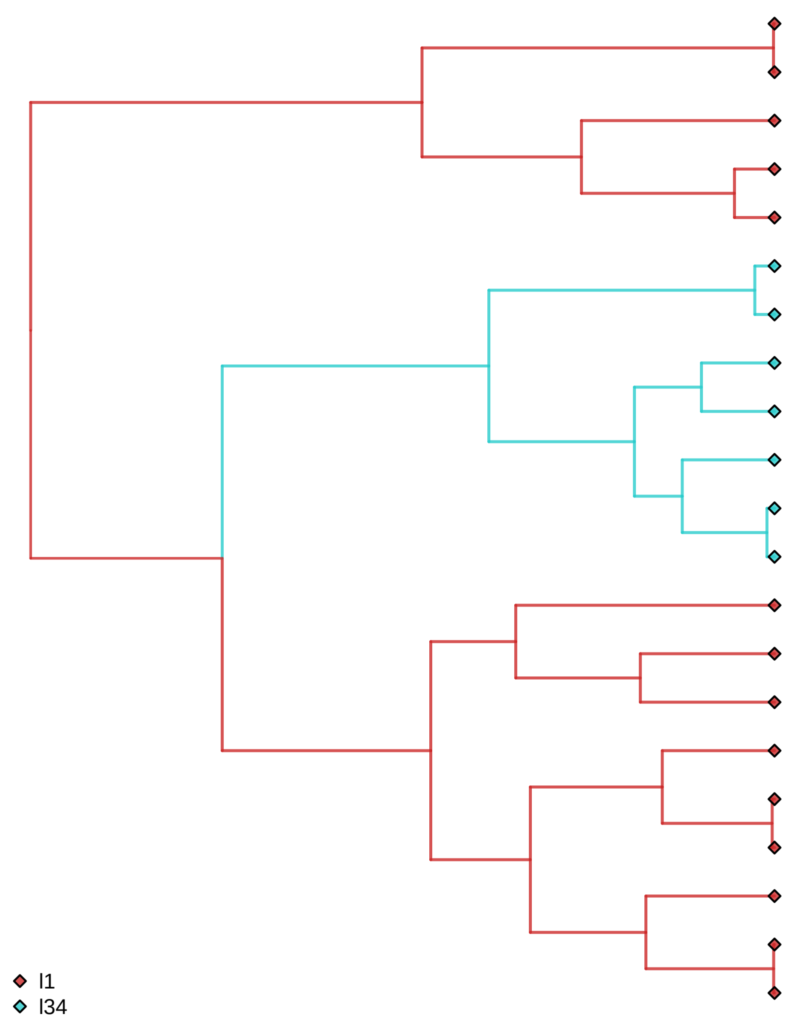

pegivirus_2002−>2021

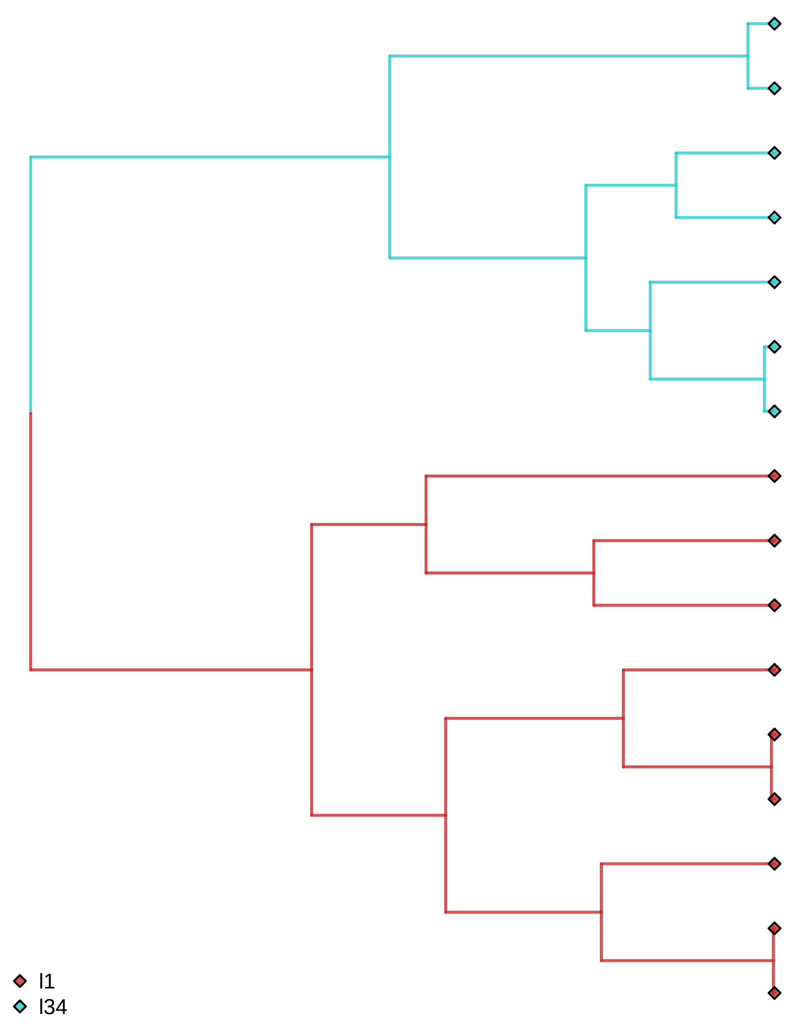

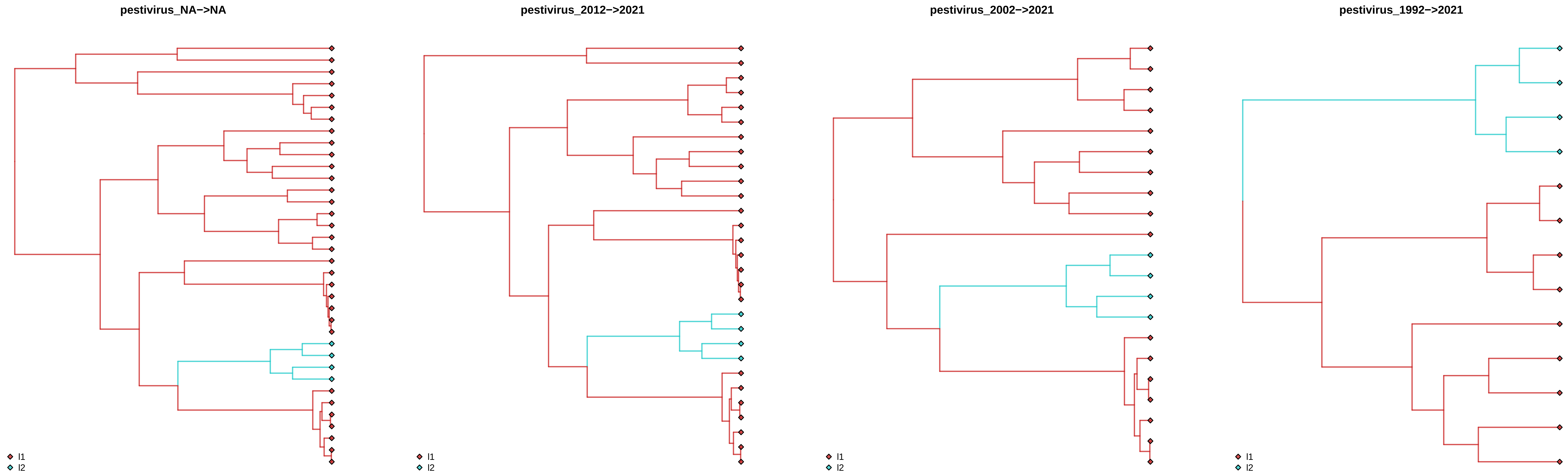

pestivirus_NA−>NA
pestivirus_2012−>2021
pestivirus_2002−>2021
pestivirus_1992−>2021
l1
l2

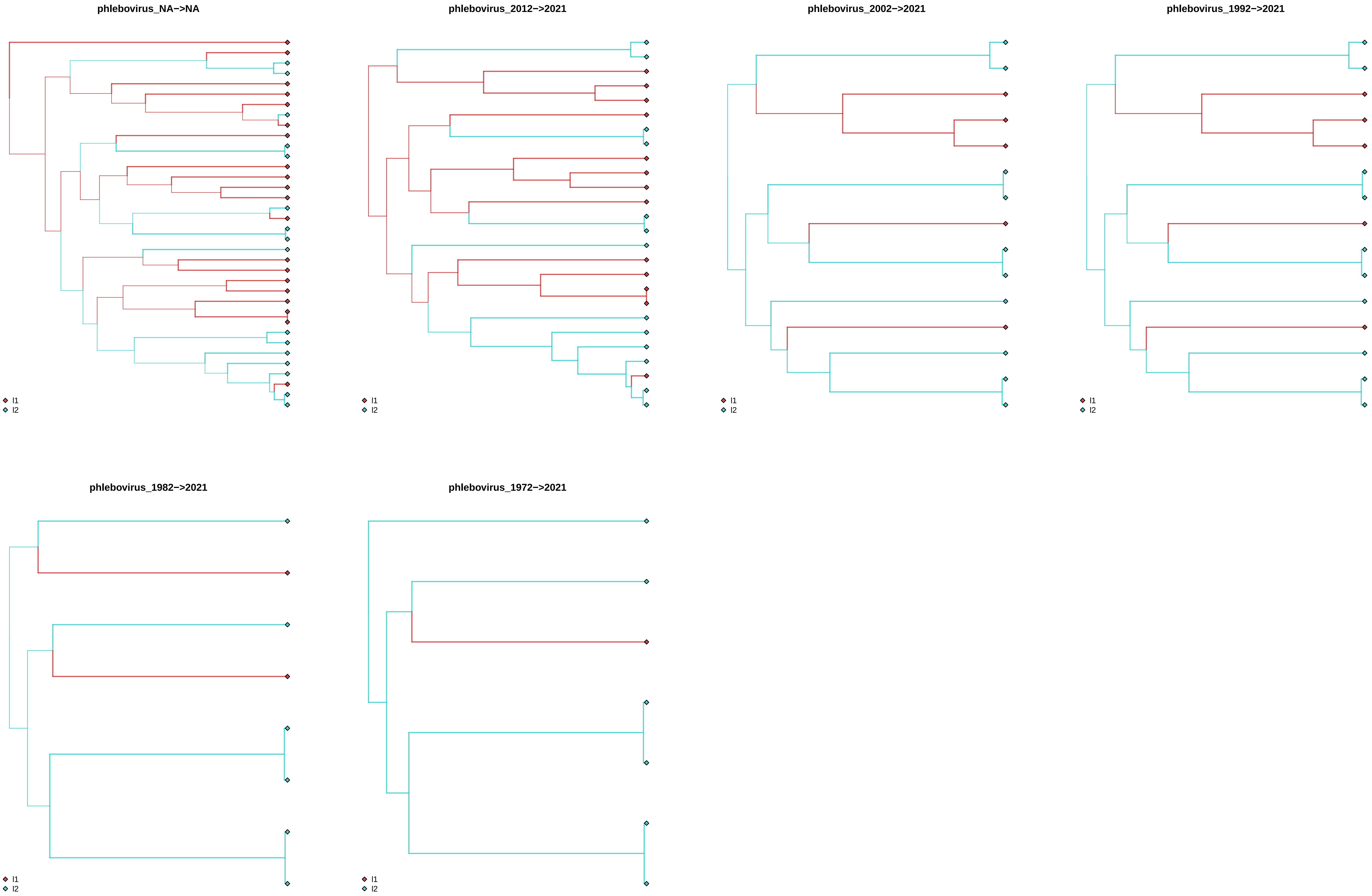
phlebovirus_NA−>NA
phlebovirus_2012−>2021
phlebovirus_2002−>2021
phlebovirus_1992−>2021
phlebovirus_1982−>2021
phlebovirus_1972−>2021
l1
l2

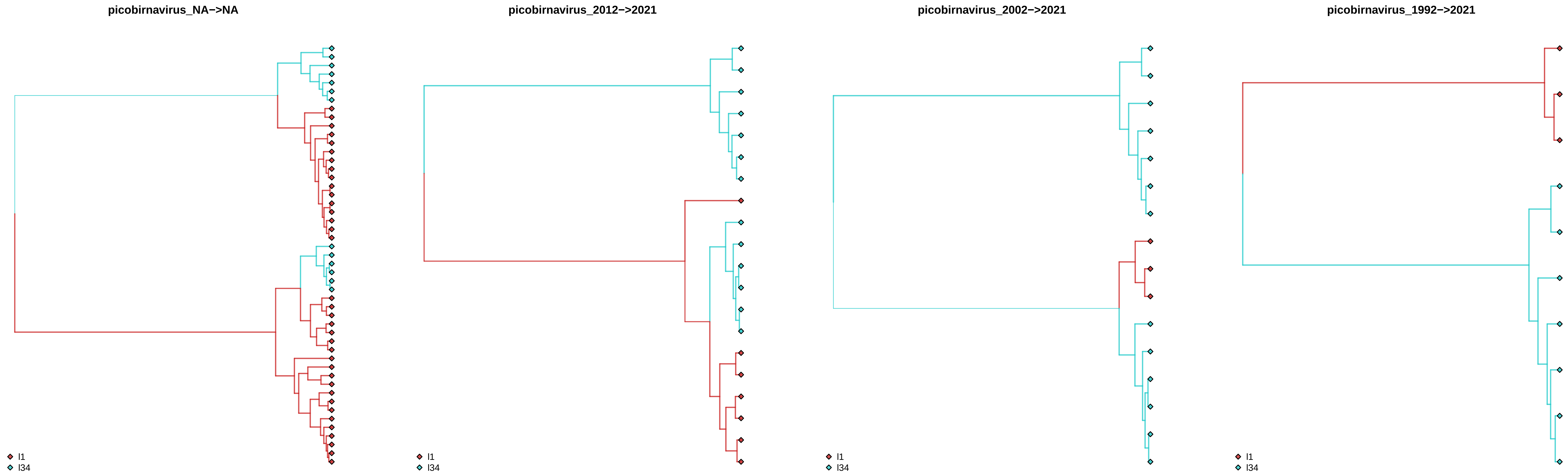
picobirnavirus_NA−>NA
picobirnavirus_2012−>2021
picobirnavirus_2002−>2021
picobirnavirus_1992−>2021
l1
l34

respirovirus_NA−>NA

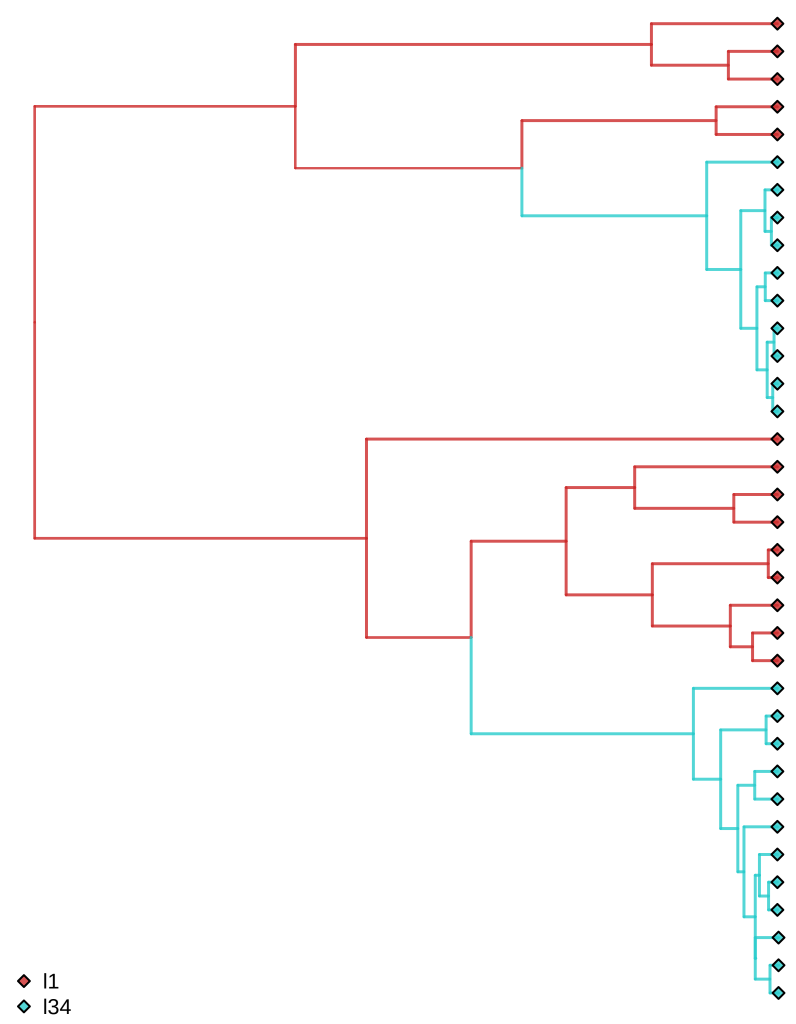

respirovirus_2012−>2021

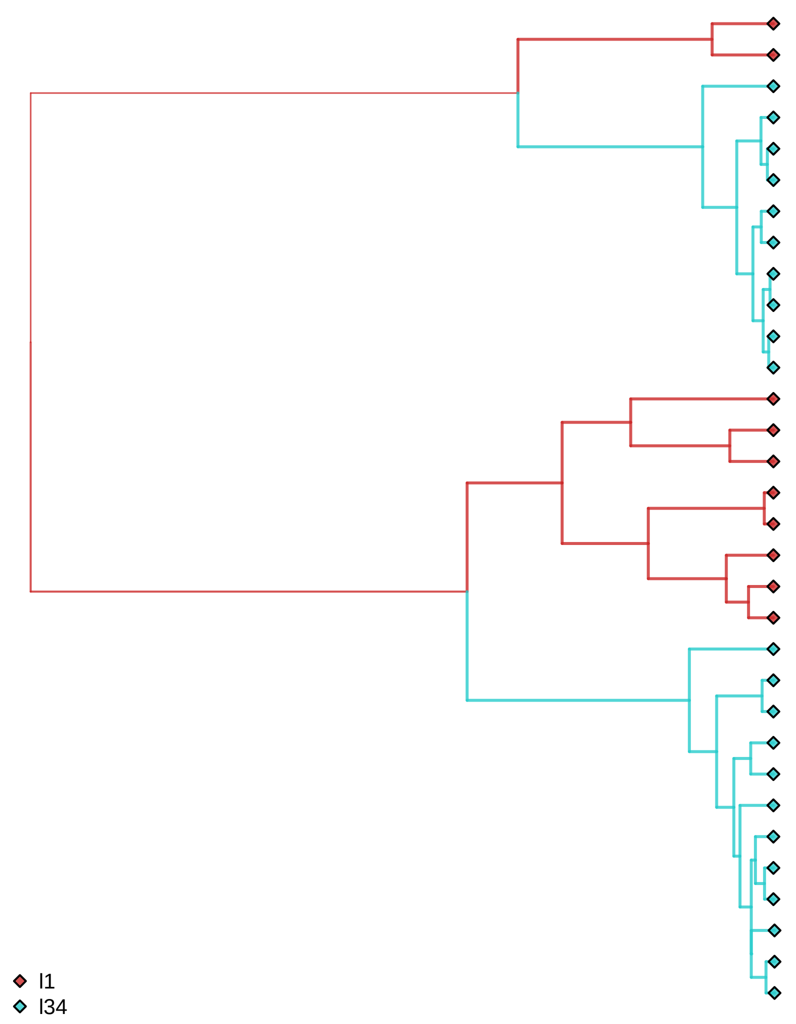

respirovirus_2002−>2021

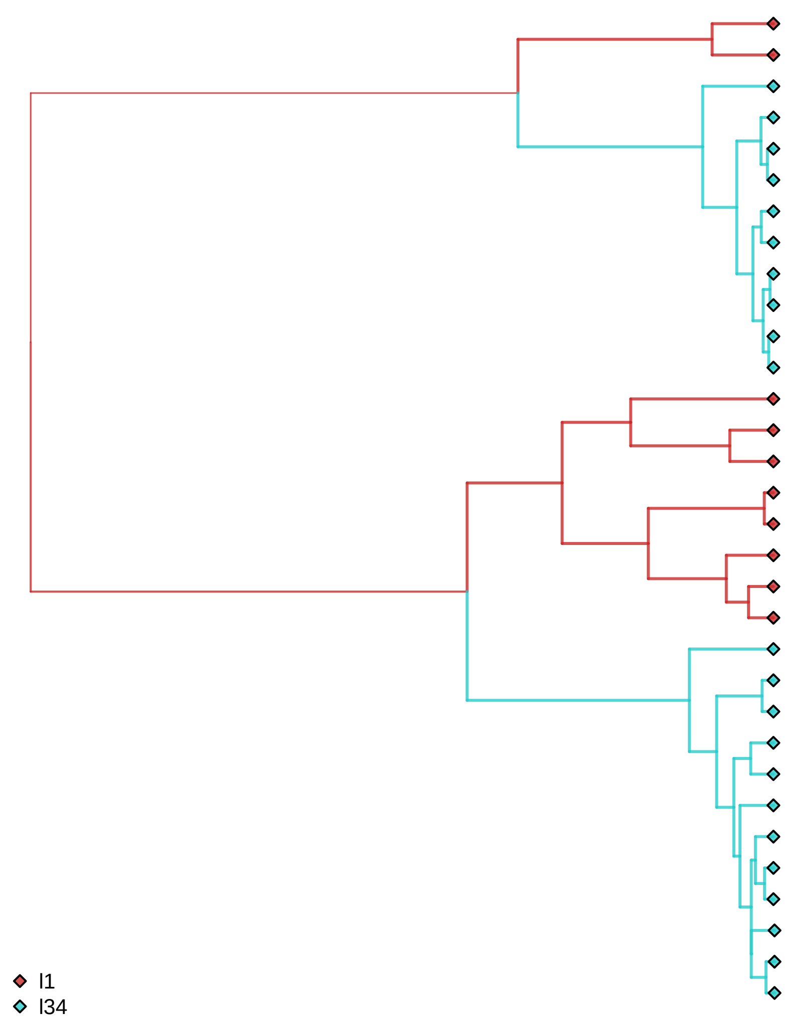

respirovirus_1992−>2021

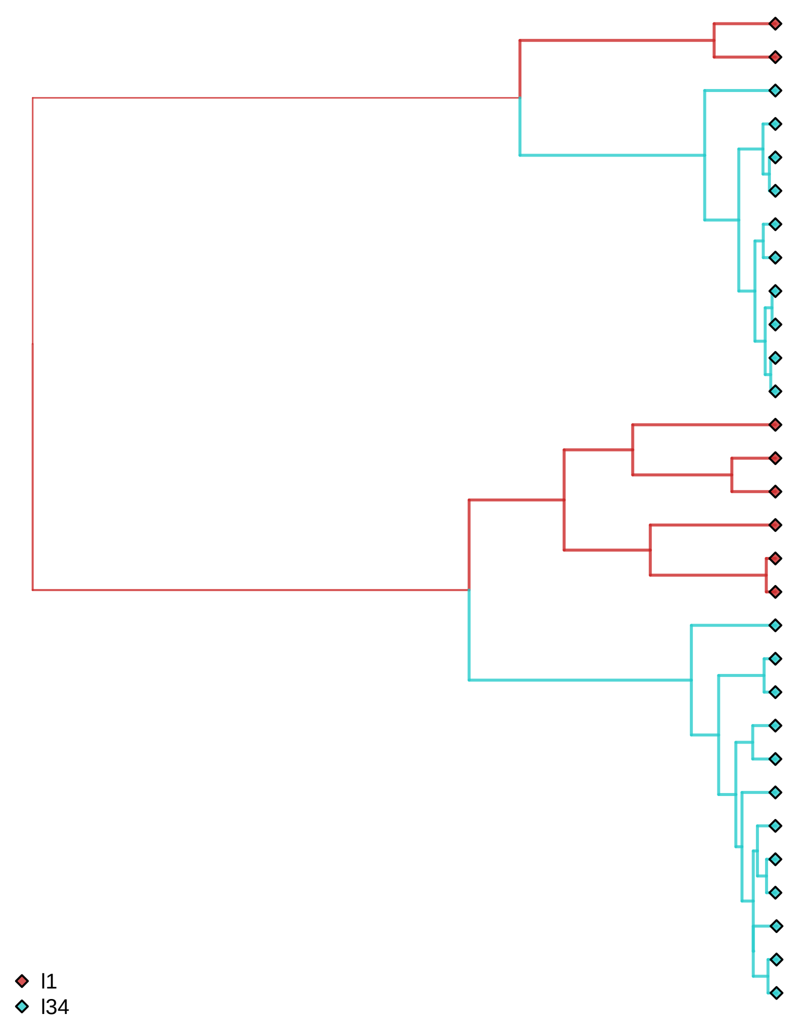

respirovirus_1982−>2021

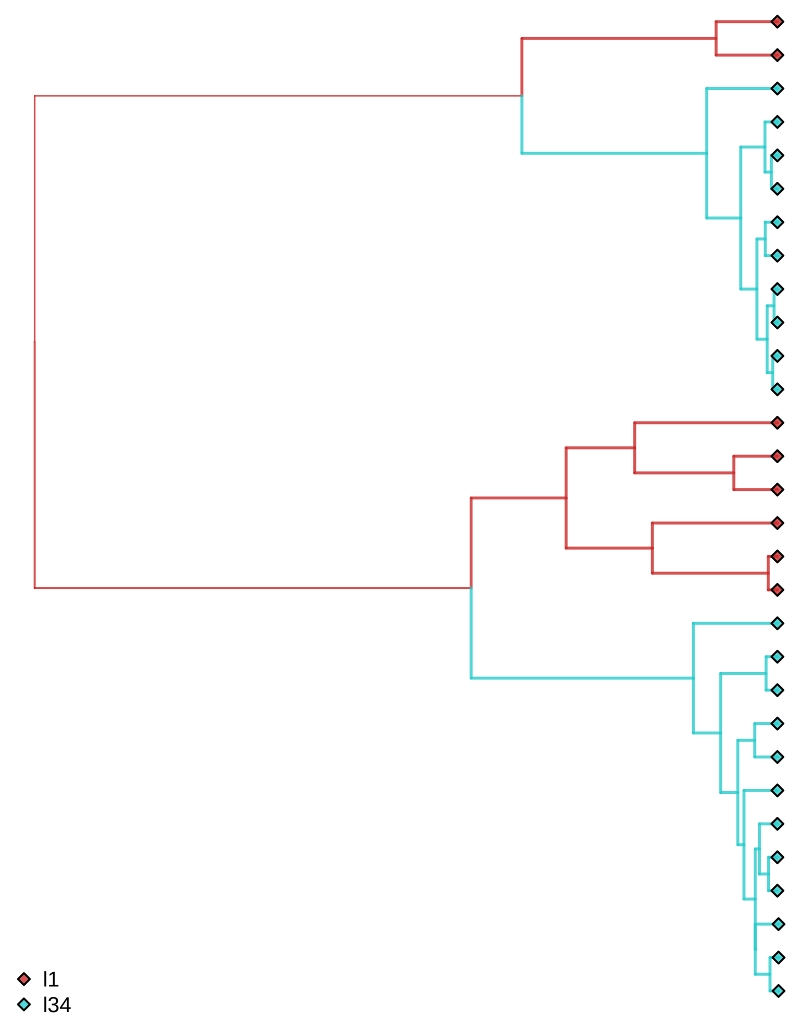

respirovirus_1972−>2021

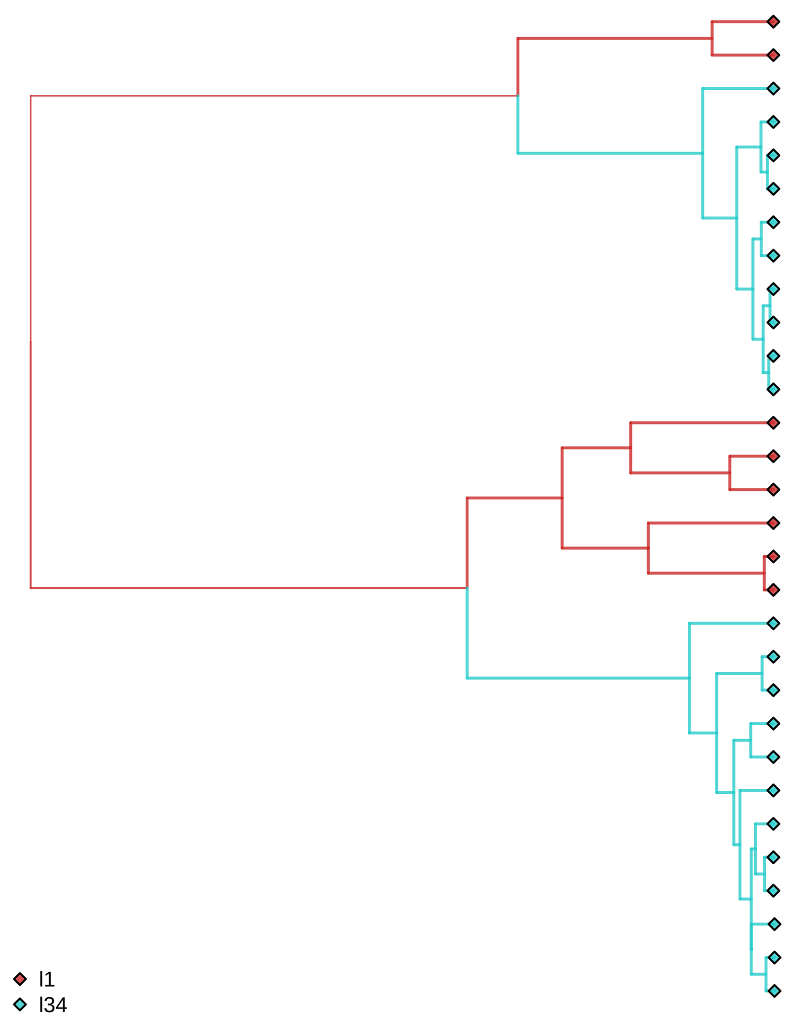

respirovirus_1962−>2021

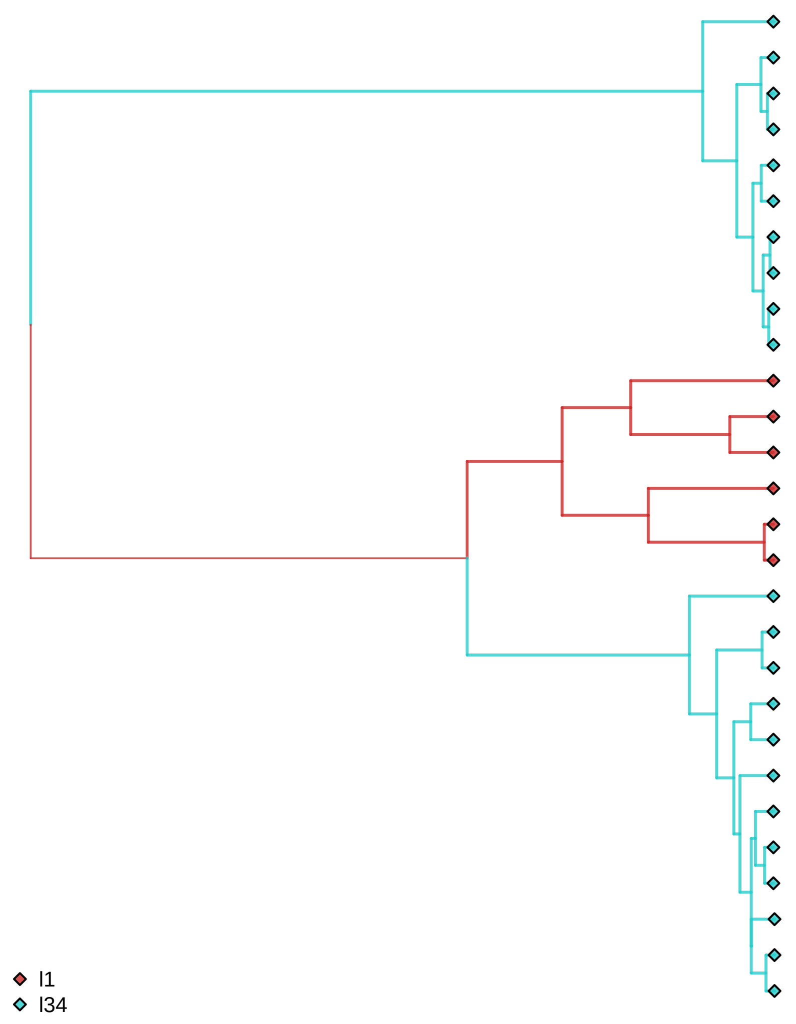

rotavirus_NA−>NA

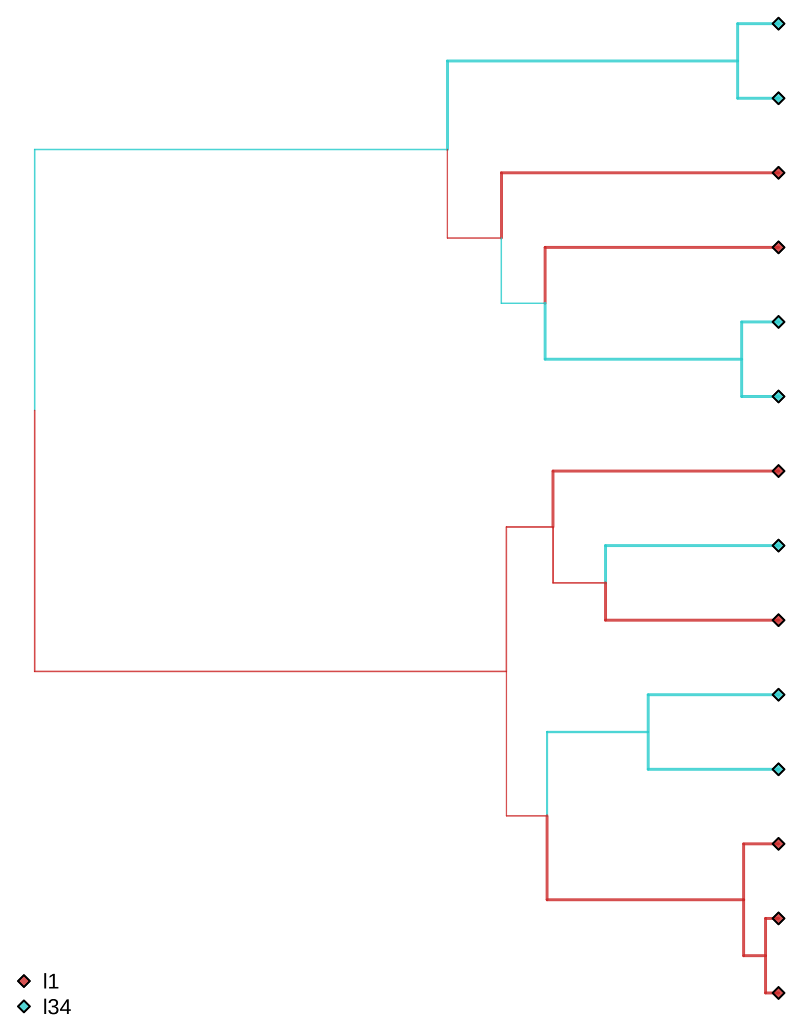

rubivirus_NA−>NA

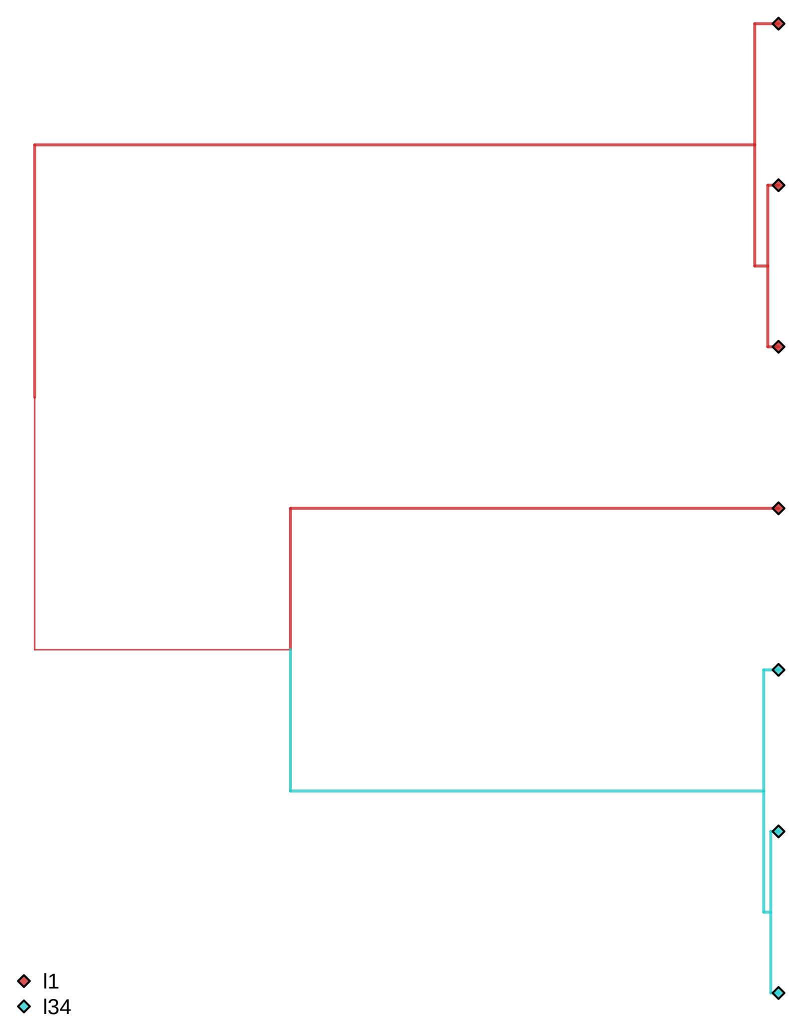

sapovirus_NA−>NA

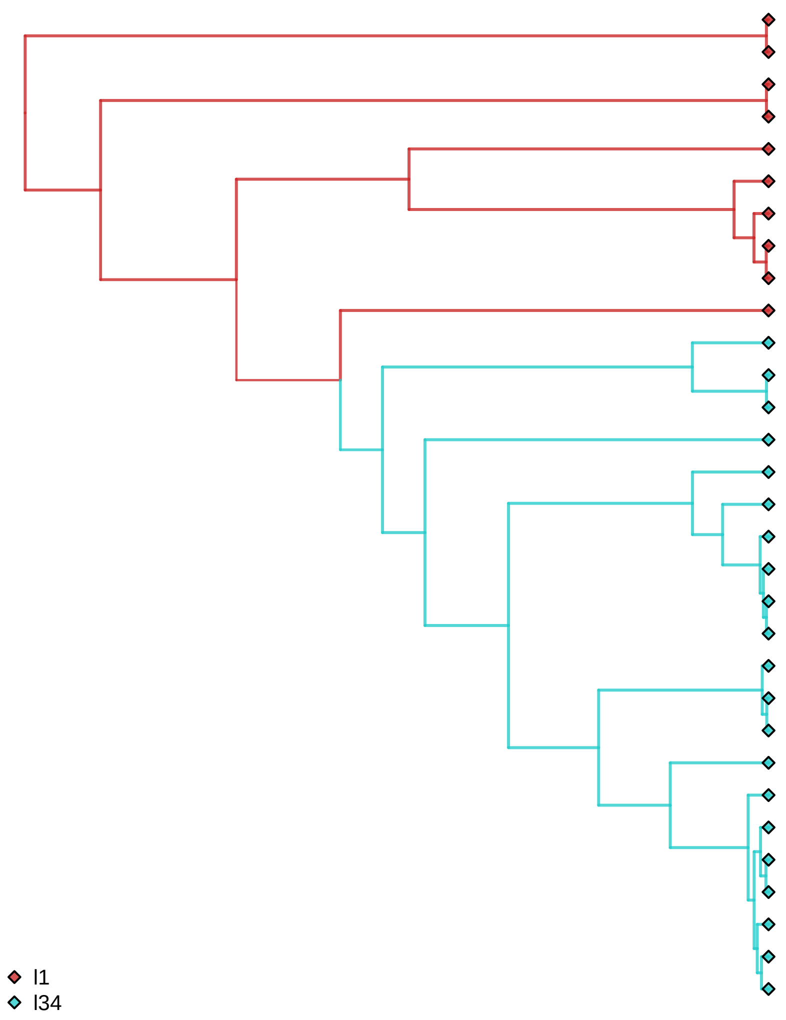

sapovirus_2012−>2021

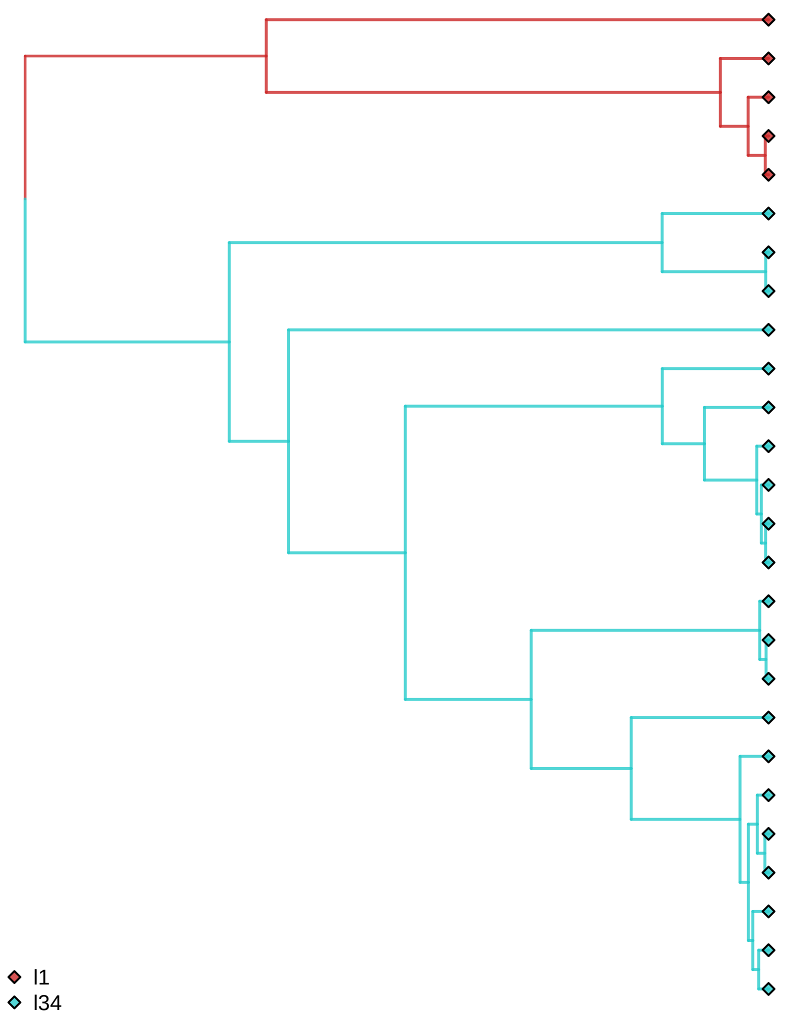

sapovirus_2002−>2021

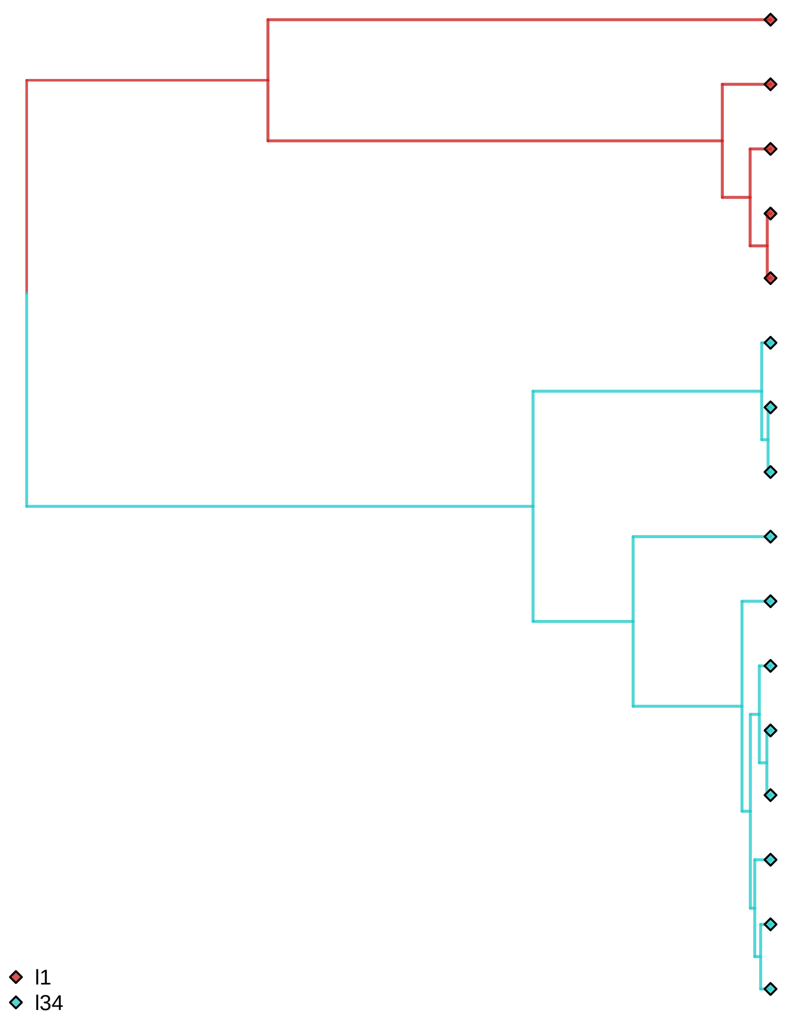

seadornavirus_NA−>NA

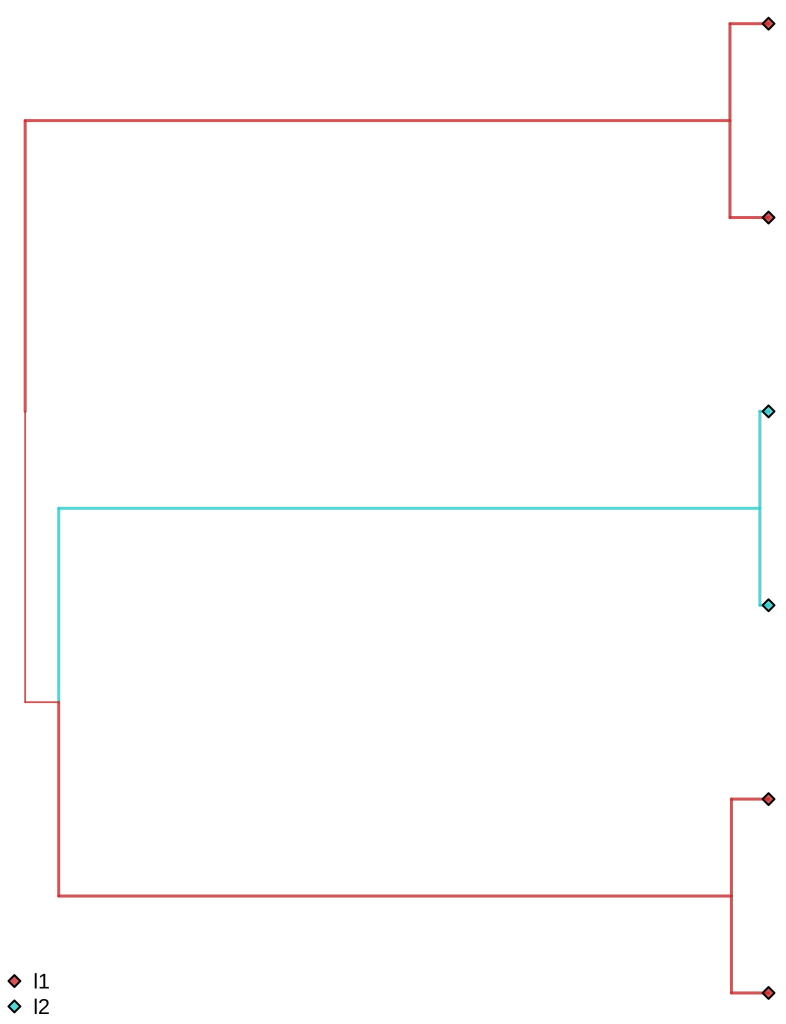

seadornavirus_2012−>2021

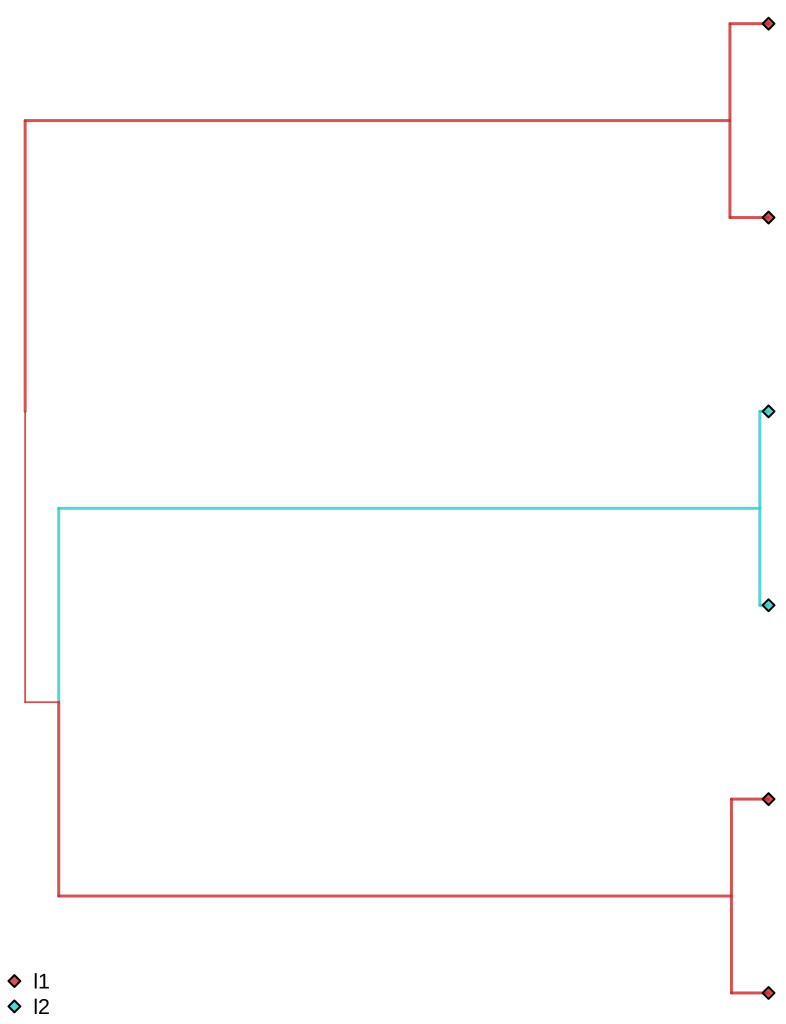

seadornavirus_2002−>2021

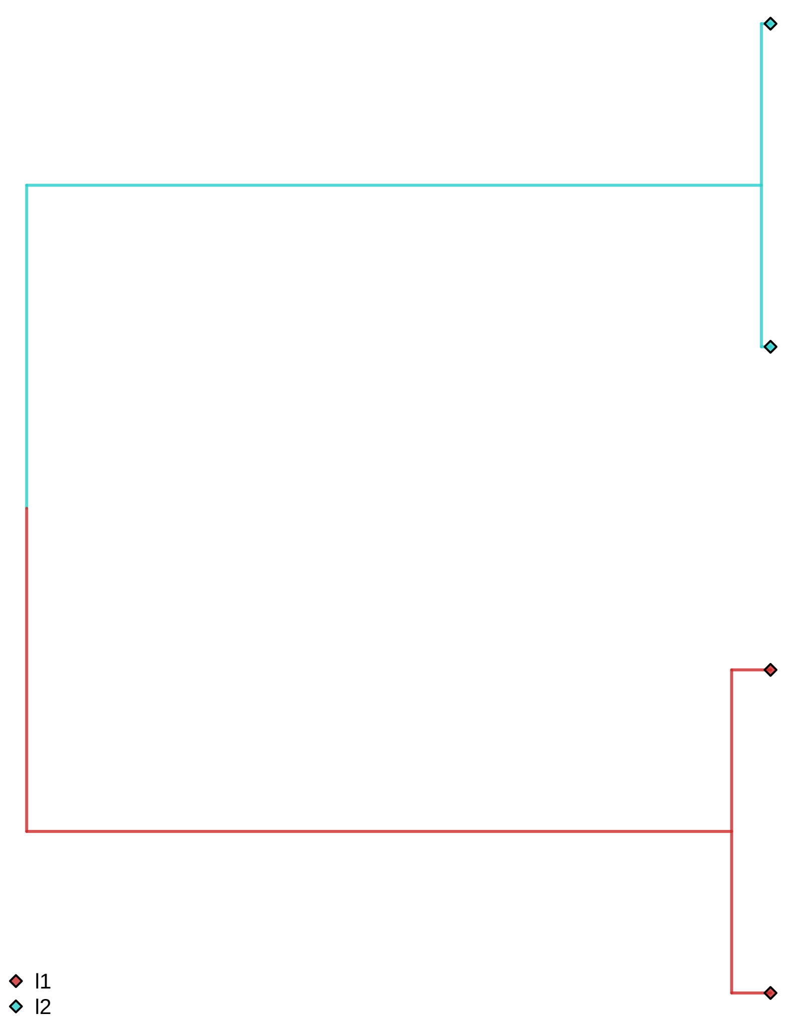

simiispumavirus_NA−>NA

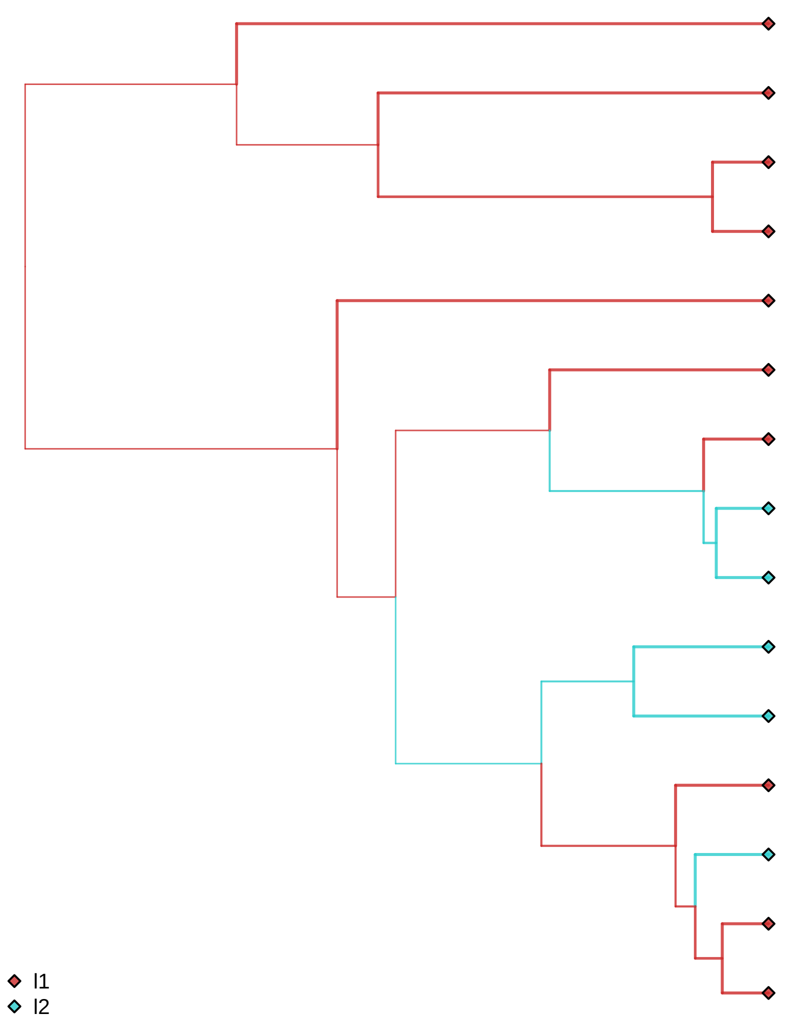

simiispumavirus_2012−>2021

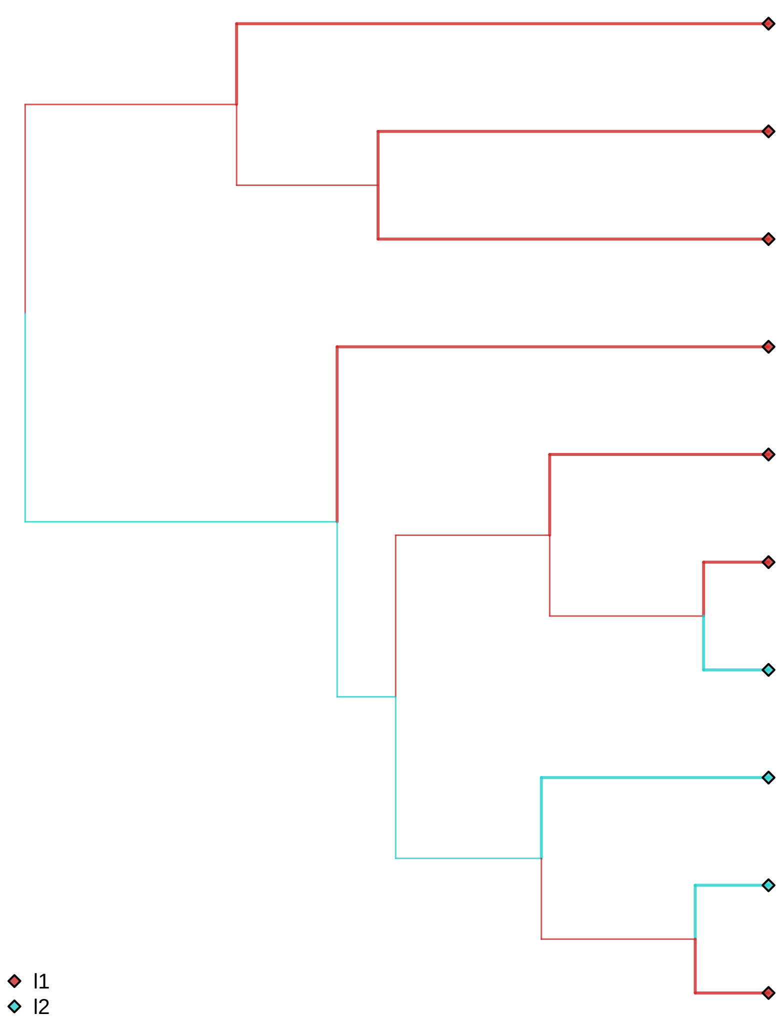

simiispumavirus_2002−>2021

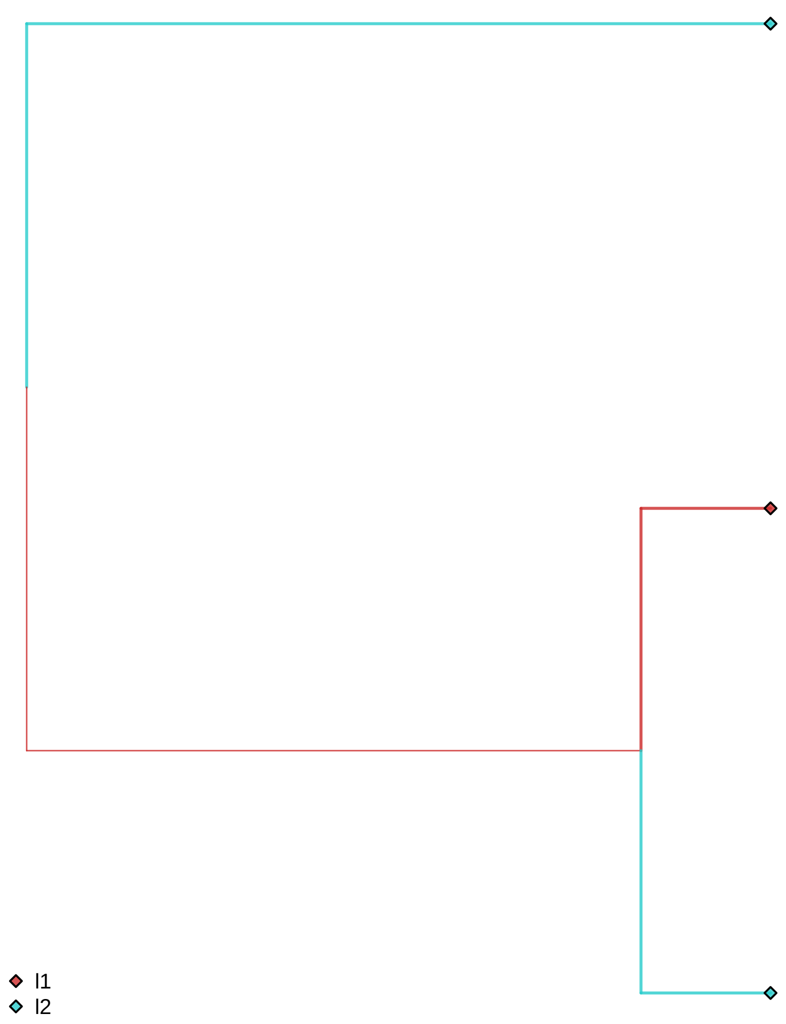

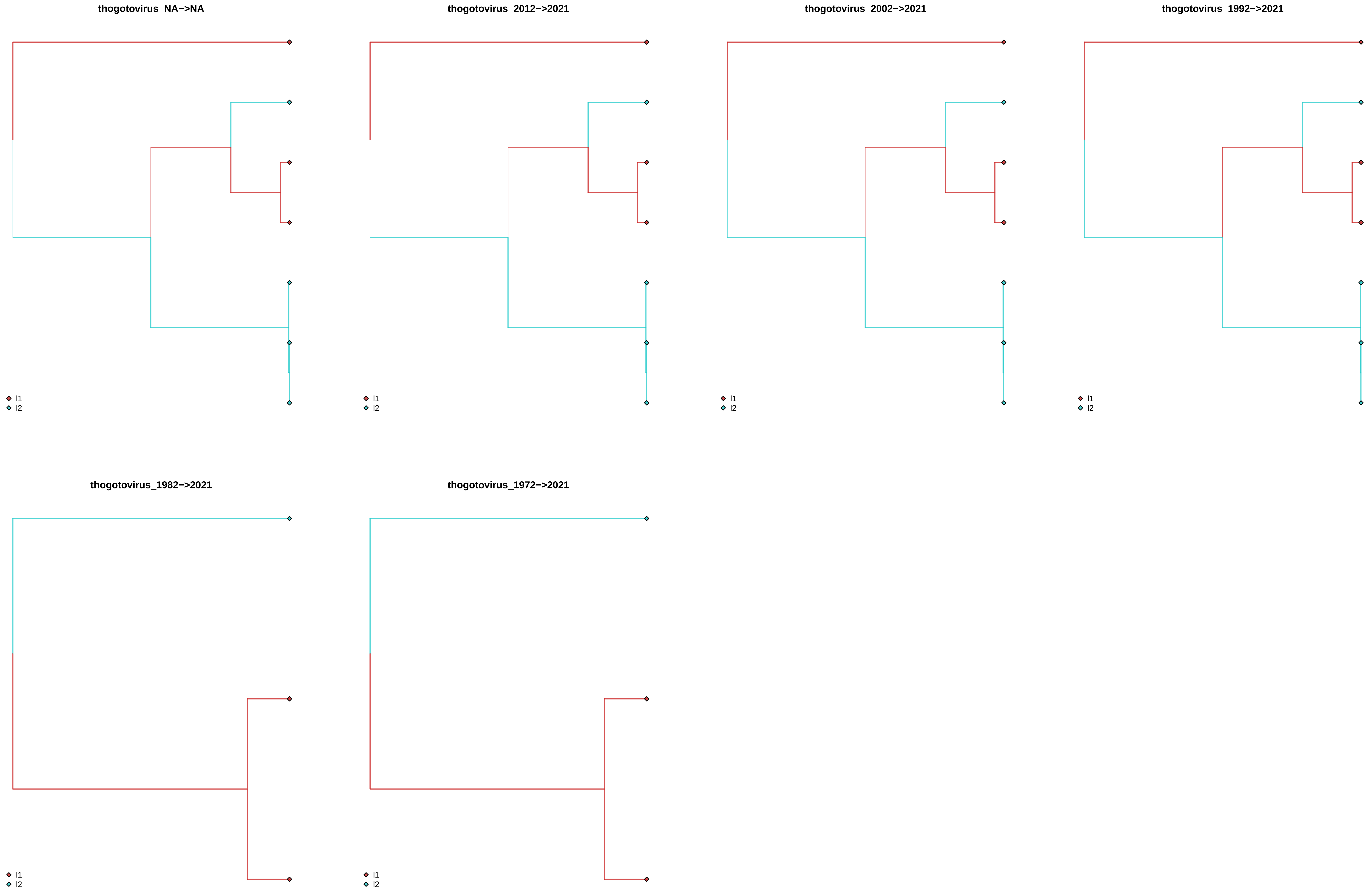
thogotovirus_NA−>NA
thogotovirus_2012−>2021
thogotovirus_2002−>2021
thogotovirus_1992−>2021
thogotovirus_1982−>2021
thogotovirus_1972−>2021
l1
l2

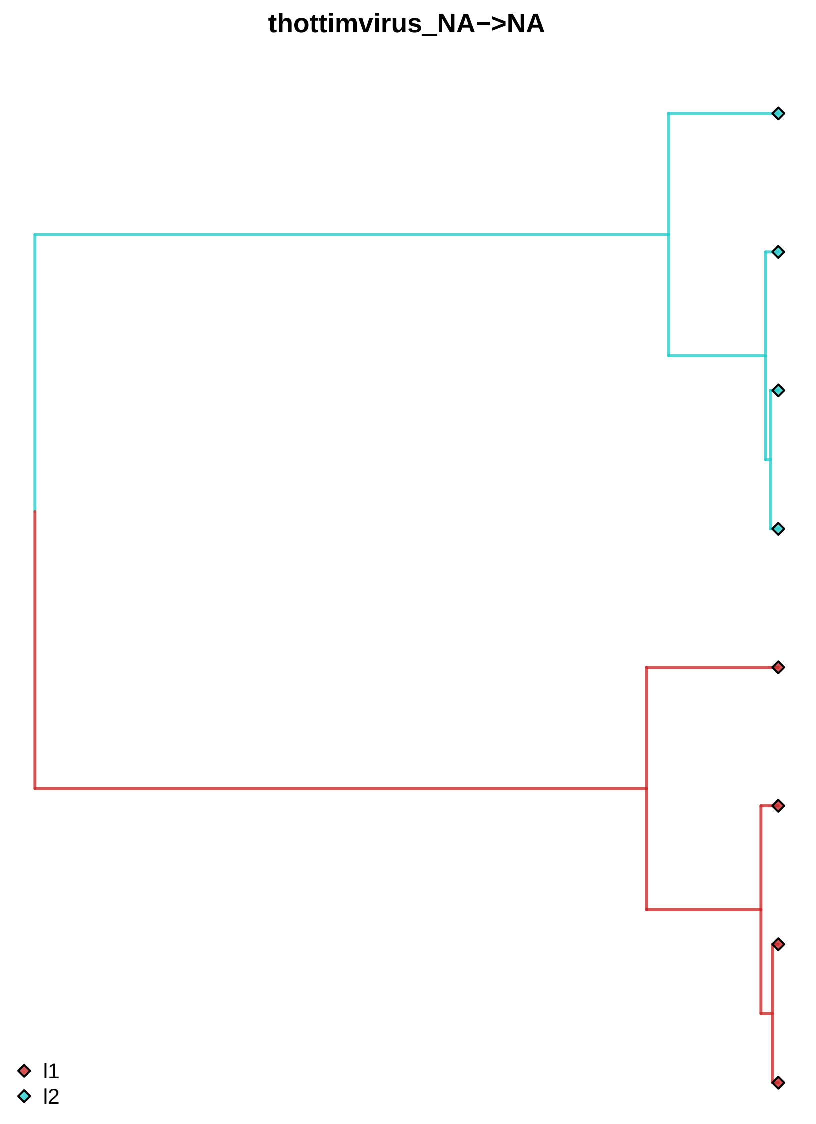
thottimvirus_NA−>NA
l1
l2

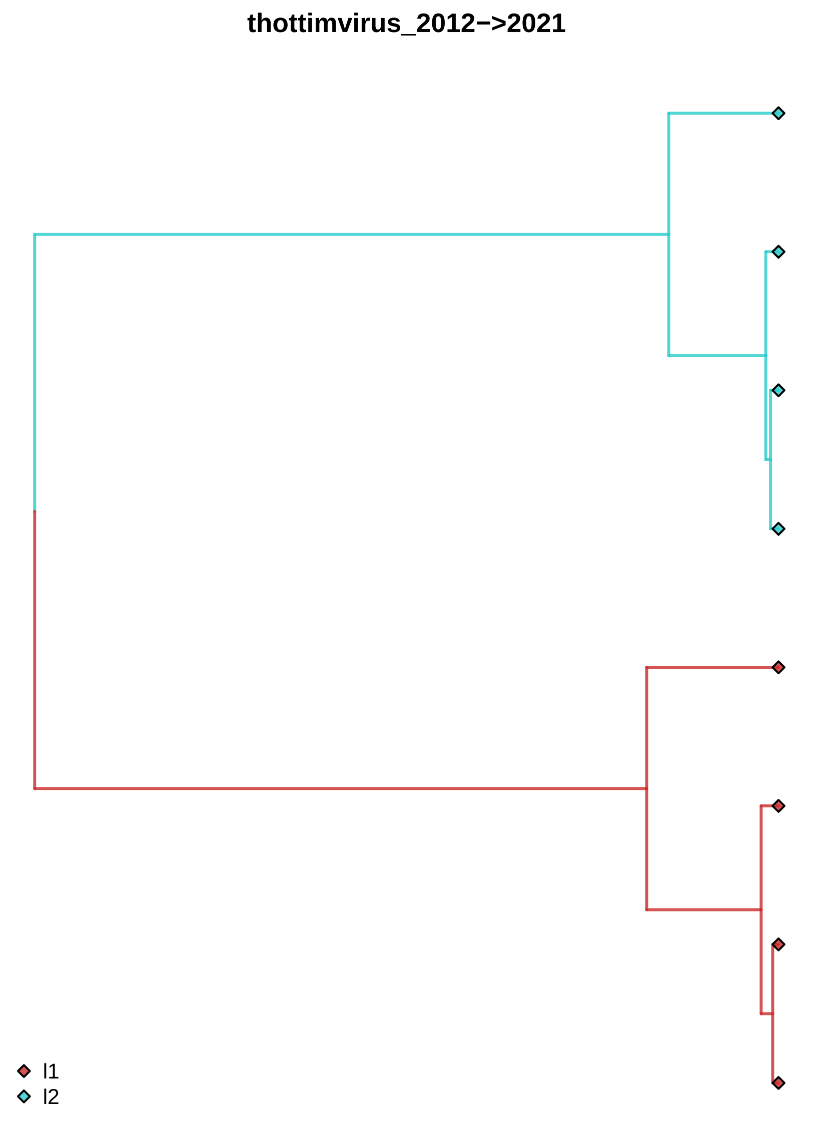
thottimvirus_2012−>2021
l1
l2

tibrovirus_NA−>NA

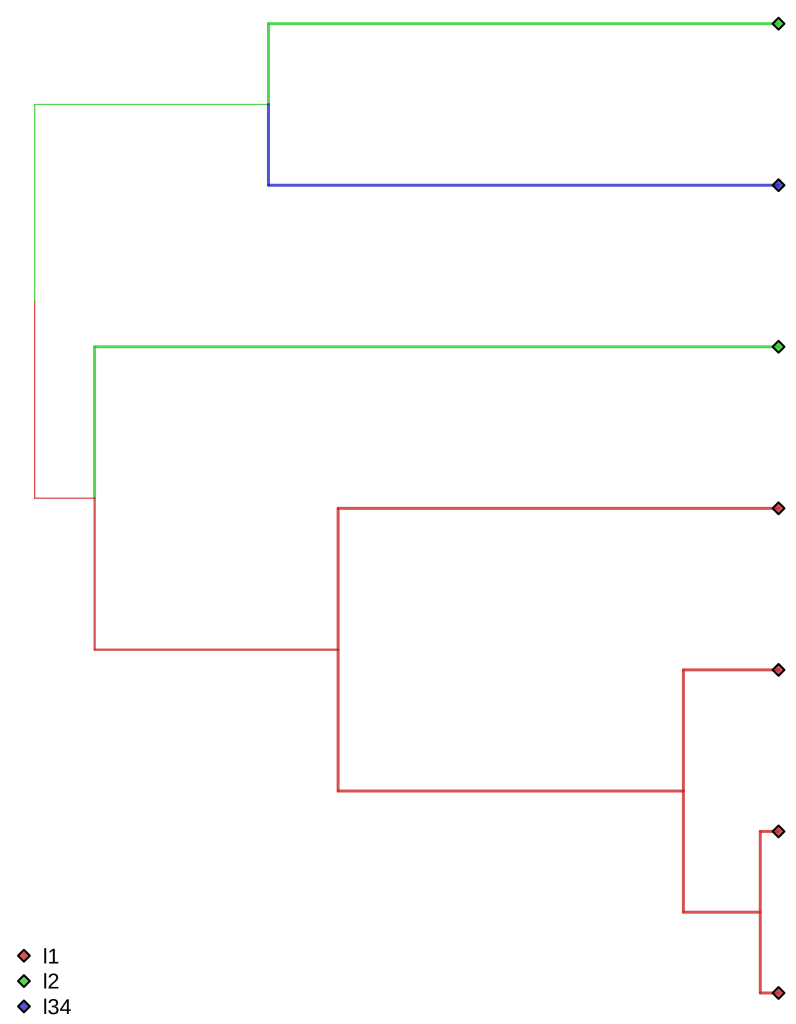

uukuvirus_NA−>NA

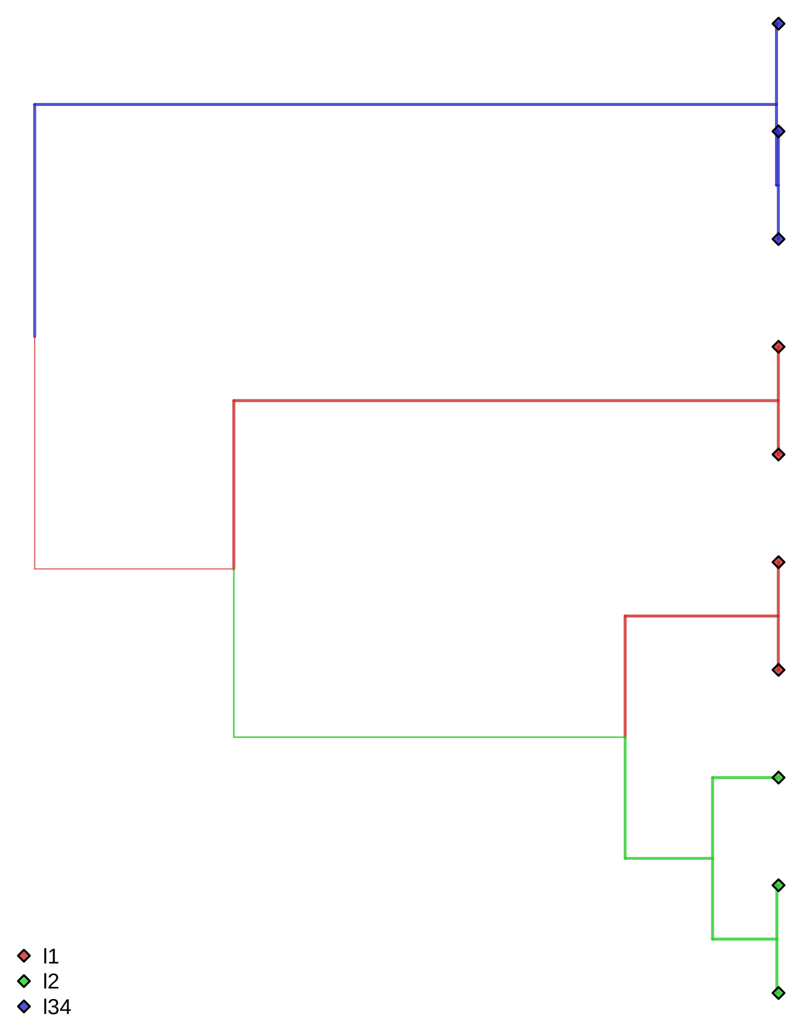

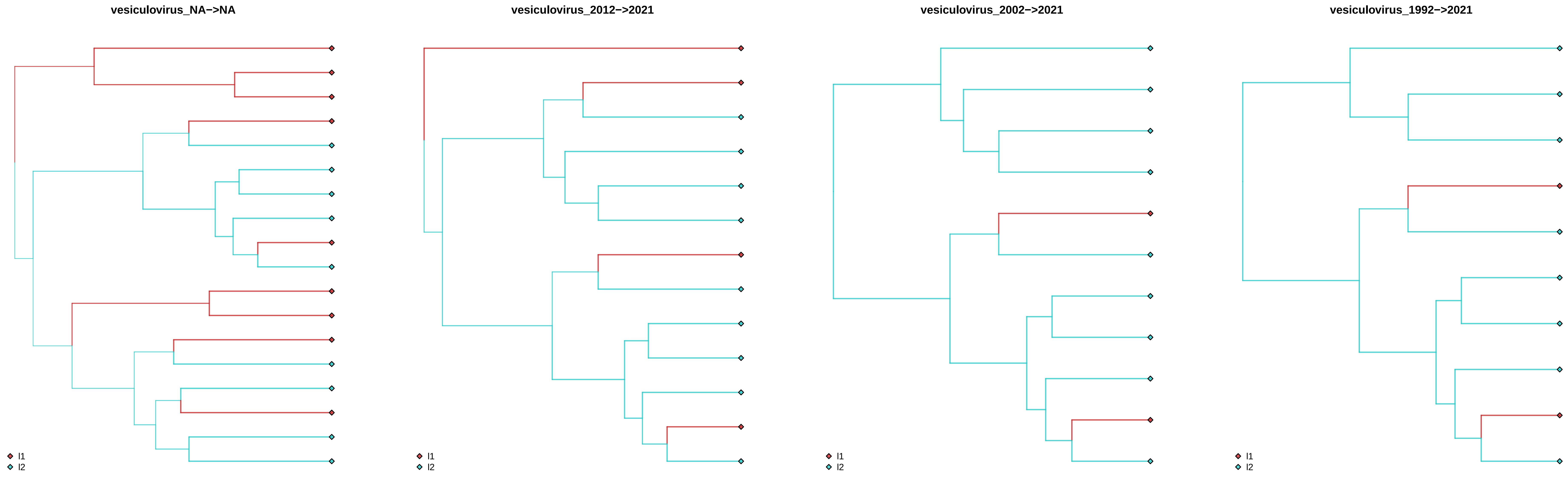

vesiculovirus_NA−>NA
vesiculovirus_2012−>2021
vesiculovirus_2002−>2021
vesiculovirus_1992−>2021
l1
l2

vesivirus_NA−>NA

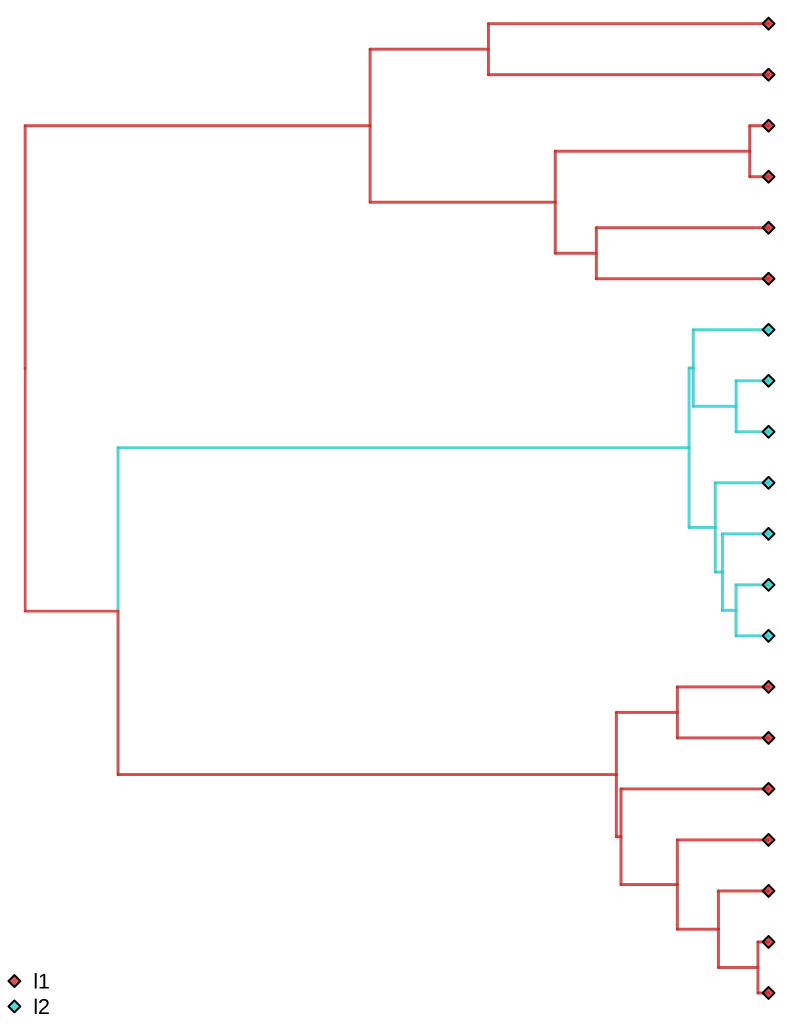

vesivirus_2012−>2021

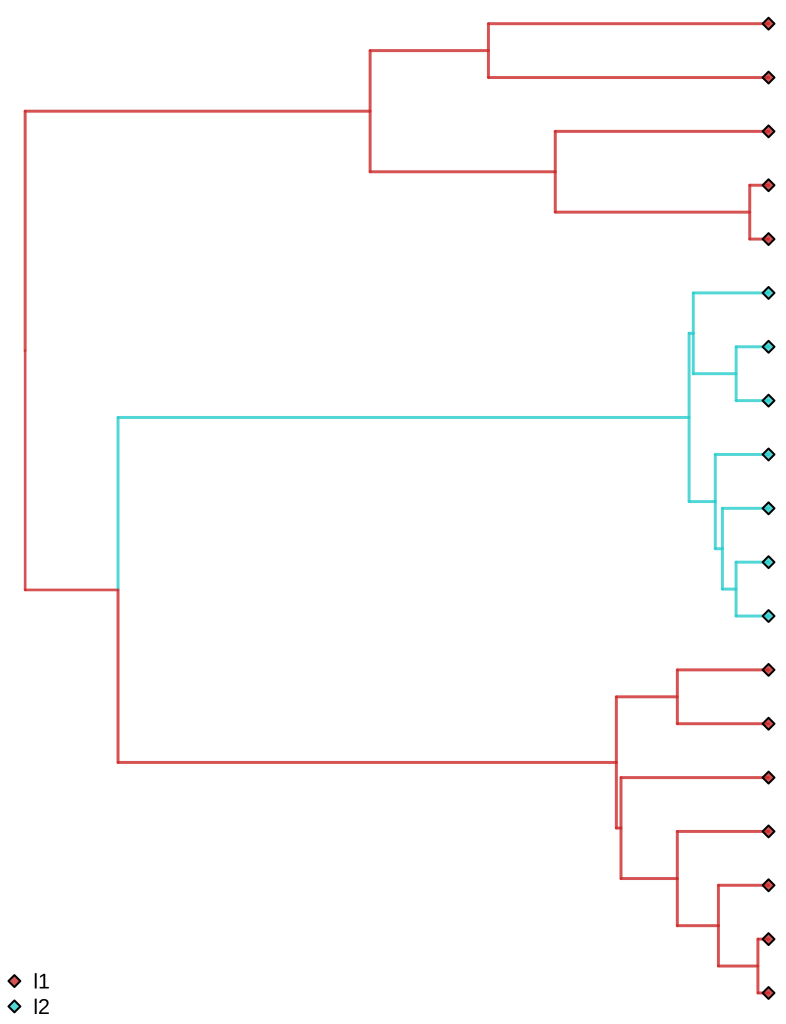

vesivirus_2002−>2021

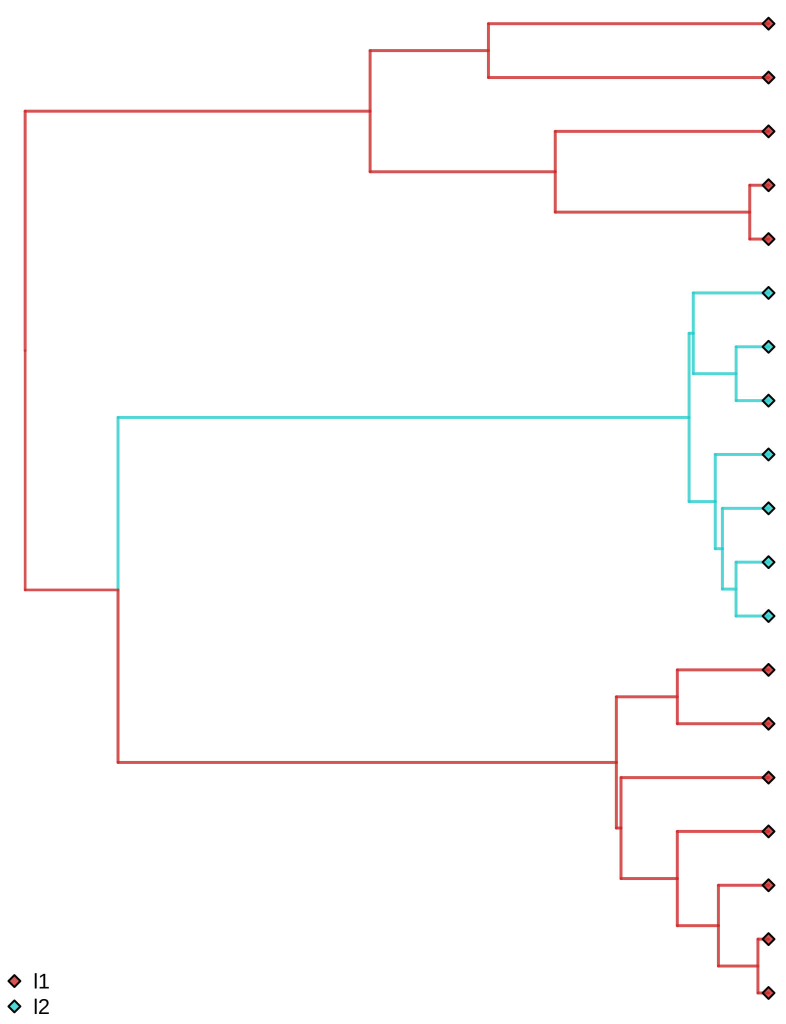

Supplement: msad272_Supplementary_Data [file msad272_supplementary_data.zip › Data file 5.pdf]
